# Positive selection and functional diversification of transcription factor Cmr1 homologs in *Alternaria*

**DOI:** 10.1007/s00253-023-12893-7

**Published:** 2024-01-15

**Authors:** Chaodong Qiu, Zhenyu Liu

**Affiliations:** 1 Department of Plant Pathology, School of Plant Protection, Anhui Agricultural University, Hefei, Anhui 230036, China; 2 Anhui Province Key Laboratory of Integrated Pest Management On Crops, Hefei, Anhui 230036 China

**Keywords:** Cmr1, *Alternaria*, Melanin, Transcription factor, Asmr1, *Alternaria solani*

## Abstract

**Abstract:**

Transcription factor Cmr1 (*Colletotrichum* melanin regulation 1) and its homologs in several plant fungal pathogens are the regulators of the 1,8-dihydroxynaphthalene (DHN)-melanin biosynthesis pathway and have evolved functional diversification in morphology and pathogenicity. The fungal genus *Alternaria* comprises the group of “black fungi” that are rich in DHN-melanin in the primary cell wall and septa of the conidia. Some *Alternaria* species cause many economically important plant diseases worldwide. However, the evolution and function of Cmr1 homologs in *Alternaria* remain poorly understood. Here, we identified a total of forty-two Cmr1 homologs from forty-two *Alternaria* spp. and all contained one additional diverse fungal specific transcription factor motif. Phylogenetic analysis indicated the division of these homologs into five major clades and three branches. Dated phylogeny showed the A and D clades diverged latest and earliest, respectively. Molecular evolutionary analyses revealed that three amino acid sites of Cmr1 homologs in *Alternaria* were the targets of positive selection. Asmr1, the homolog of Cmr1 in the potato early blight pathogen, *Alternaria solani* was amplified and displayed the sequence conservation at the amino acid level in different *A. solani* isolates. Asmr1 was further confirmed to have the transcriptional activation activity and was upregulated during the early stage of potato infection. Deletion of *asmr1* led to the decreased melanin content and pathogenicity, deformed conidial morphology, and responses to cell wall and fungicide stresses in *A. solani*. These results suggest positive selection and functional divergence have played a role in the evolution of Cmr1 homologs in *Alternaria*.

**Key points:**

*• Cmr1 homologs were under positive selection in Alternaria species*

*• Asmr1 is a functional transcription factor, involved in spore development, melanin biosynthesis, pathogenicity, and responses to cell wall and fungicide stresses in A. solani*

*• Cmr1 might be used as a potential taxonomic marker of the genus Alternaria*

**Supplementary Information:**

The online version contains supplementary material available at 10.1007/s00253-023-12893-7.

## Introduction

Melanin, a natural amorphous polymer of high molecular weight, is a ubiquitous pigment found in microorganisms, plants, and animals (Cordero and Casadevall 2020). Many plant pathogenic ascomycete and dothideomycete fungi synthesize melanin following the 1,8-dihydroxynaphthalene (DHN) pathway (Kimura and Tsuge 1993; Tsuji et al. 2000; Eisenman and Casadevall 2012; Schumacher 2016; Wang et al. 2021b; Li et al. 2022). In fungi, melanin confers protection against abiotic stresses, such as ultraviolet radiation, extreme temperatures, reactive oxygen species (ROS), high osmotic pressure, hypertonic and heavy metal exposure, and dehydration (Cordero and Casadevall 2017). Melanin is also involved in fungal cell development of conidia, black appressoria, and sclerotia (Money et al. 1998; Kheder et al. 2012; Yu et al. 2015; Liang et al. 2018; Wang et al. 2021a; Zhou et al. 2022). In addition, DHN-melanin has a function in virulence of some phytopathogenic fungi during their interactions with the host immune response. For those fungi that produce melanized appressoria for host penetration, such as the rice blast pathogen *Magnaporthe oryzae* and the cucumber anthracnose pathogen *Colletotrichum lagenarium*, DHN-melanin is required for appressorial melanogenesis for pathogenicity (Kubo et al. 1982; Howard and Valent 1996). However, similarly, in some fungi like the gray mold pathogen *Botrytis cinerea* that do not form appressoria, DHN-melanin also correlates with their virulence. Mutants of *B. cinerea* defective in three melanin biosynthesis-related genes fail to produce melanin, but they show increased virulence (Zhang et al. 2015; Zhou et al. 2022).

Cmr1 (*Colletotrichum* melanin regulation 1) first described in *Colletotrichum lagenarium*, is an important transcription factor for regulating the expression of key enzymes in fungal DHN-melanin biosynthesis pathway, including polyketide synthase, 1,3,6,8-tetrahydroxynaphthalene reductase, scytalone dehydratase, 1,3,8-trihydroxynaphthalene reductase, and laccase (Tsuji et al. 2000; Eisenman and Casadevall 2012). Cmr1 is characterized by the presence of two types of DNA binding motifs, two Cys_2_His_2_ zinc finger motifs and one GAL4-like Zn(II)_2_Cys_6_ binuclear cluster DNA-binding motif. Until now, Cmr1 homologs that have been identified and confirmed their roles as the regulators in the DHN-melanin biosynthesis pathway of several plant fungal pathogens, include Pig1 in *Magnaporthe grisea* (Tsuji et al. 2000), Cmr1 in *Cochliobolus heterostrophus* (Eliahu et al. 2007), Bmr1 in *Bipolaris oryzae* (Kihara et al. 2008), Amr1 in *Alternaria brassicicola* (Cho et al. 2012), CmrA in *Alternaria alternata* (Fetzner et al. 2014), BcSMR1 in *Botrytis cinerea* (Zhou et al. 2017), Zmr1 in *Zymoseptoria tritici* (Krishnan et al. 2018), CgCmr1 in *Colletotrichum gloeosporioides* (Wang et al. 2021a, b), and StMR1 in *Setosphaeria turcica* (Zhang et al. 2022). Apart from a role in controlling DHN-melanin biosynthesis, Cmr1 homologs also affect the morphology and pathogenicity of some phytopathogenic fungi, suggesting the functional diversification of Cmr1 homologs. It is noteworthy that some Cmr1 homologs including BcSMR1, Zmr1, and CgCmr1 have no role in pathogenicity, while StMR1 positively regulates the pathogenicity and Amr1 negatively affects the pathogenicity. Further investigating the function of other Cmr1 homologs will provide support about the functional difference in pathogenicity of Cmr1 homologs.

The worldwide-distributed fungal genus *Alternaria* is the typical representative of *Dematiaceae* and comprises the group of “black fungi”, which are rich in DHN-melanin in the primary cell wall and septa of their conidia (Carzaniga et al. 2002; Thomma 2003). It is currently classified into 24 sections based on phylogenetic and morphological studies (Woudenberg et al. 2013). Some *Alternaria* species are fungal pathogens of many economically important plant diseases, which result in huge losses in agricultural production and postharvest agricultural products throughout the world (Thomma 2003). *A. brassicicola* is responsible for black spot disease on almost all cultivated *Brassica* species including cabbage, broccoli, canola, mustard, and rapeseed in the world (Neergaard 1945; Sigareva and Earle 1999; Westman et al. 1999). *A. alternata* is a pathogen on over 100 plant hosts including cereal crops, vegetables, and fruits (Rotem 1994). For example, *A. alternata* causes *Alternaria* brown spot of citrus (Gai et al. 2021), leaf blight on *Ophiopogon japonicus* (Wang et al. 2021a, b), and black spot of kiwifruit (Huang et al. 2021). *Alternaria solani* is the causal agent of early blight, one of the most destructive diseases of potato and tomato worldwide (Agrios 2005). In China, early blight ranks number 1 fungal disease on potato (Ma et al. 2020). *A. solani* also causes early blight on other members of the *Solanaceae* family including pepper, eggplant, black nightshade, horse nettle, and Jerusalem cherry (Farr and Rossman 2023). Although potato early blight is a devastating disease, only a few factors involved in pathogenicity have been identified in *A. solani*, such as toxins: alternaric acid and solanapyrones A, B, and C (Brian et al. 1949).

Currently, two Cmr1 homologs including Amr1 in *A. brassicicola* (Cho et al. 2012) and CmrA in *A. alternata* (Fetzner et al. 2014) have been identified and characterized in *Alternaria*. The function of Cmr1 homologs in regulating the DHN-melanin biosynthesis is highly conserved in *A. brassicicola*, *A. alternata*, and other fungi described above (Tsuji et al. 2000; Eliahu et al. 2007; Kihara et al. 2008; Cho et al. 2012; Fetzner et al. 2014; Zhou et al. 2017; Krishnan et al. 2018; Wang et al. 2021a, b; Zhang et al. 2022). Interestingly, based on the amino acid sequence comparison between Amr1 and CmrA, there was one amino acid insertion at site 934 in Amr1 and 31 polymorphic sites (data not shown), suggesting that the amino acid sequences have diverged in Amr1 and CmrA. Furthermore, Amr1 negatively regulates the pathogenicity of *A. brassicicola* (Cho et al. 2012). Cmr1 homologs in phytopathogenic fungi have evolved to differentiate in pathogenicity, which may relate to the interaction with their host plants. As a consequence, given the availability of the genome sequences of 44 *Alternaria* species to date and our laboratory stored 10 *A. solani* isolates from different geographic locations in China, we raised the following questions. How did Cmr1 homologs evolve in *Alternaria* and in *A. solani*? What kind of functions the Cmr1 homolog in *A. solani* may have? Of particular interest, whether there is functional difference in pathogenicity regulated by Cmr1 homologs in *Alternaria*? Here, we exploited *Alternaria* genomes for identifying Cmr1 homologs followed by investigating the diversity and evolutionary relationship of Cmr1 homologs in *Alternaria* as well as those in *A. solani*. We also engineered one *A. solani* isolate deficient in the production of a Cmr1 homolog using a gene knockout technology to test whether Cmr1 homolog acts as a virulence factor and whether DHN-melanin is important for pathogenesis of *A. solani*.

## Materials and methods

### Plants, fungal isolates, and growth conditions

Potato (*Solanum tuberosum* cv. Zaodabai and cv. Favorita) plants were grown and maintained in a growth chamber (23 °C day/15 °C night temperatures with 14 h of light). *A. solani* isolates (Supplemental Table S1) and mutants were maintained as glycerol stocks. For conidial sporulation, the wild type (WT) *A. solani* isolate SH0806 and its mutants were grown on potato carrot agar (PCA) medium at 25 °C.

### Database mining

The protein sequence of Amr1 was used as a query to BlastP searching against all annotated protein databases of *Alternaria* spp. available in the websites of GenBank, National Center for Biotechnology Information (http://www.ncbi.nih.gov), JGI, Joint Genome Institute, US Department of Energy (http://www.jgi.doe.gov), and AGD, *Alternaria* genome database (http://alternaria.vbi.vt.edu) to identify Cmr1 homologs in *Alternaria*. For some *Alternaria* species without genome annotation, first, their corresponding genome sequences were predicted into genes through the AUGUSTUS server (Stanke and Morgenstern 2005). Second, the local protein databases were constructed using the Blast-2.12.0 program (Altschul et al. 1997). Third, BLASTP searching was performed against the generated local protein databases using Amr1 as a query. Finally, all achieved protein sequences were searching against the PFAM database in SMART server (Letunic et al. 2020) to confirm the existence of characteristic motifs of Cmr1.

### Sequence analyses

The amino acid sequences corresponding to individual motifs of Cmr1 homologs were aligned using the ClustalX program (Thompson et al. 1997) and submitted to WebLogo server (Crooks et al. 2004) to calculate the consensus sequences. MEGA7.0.21 (Kumar et al. 2016) was used to make multiple sequence alignments of Cmr1 homologs followed by reconstructing a phylogenetic tree using the maximum likelihood (ML) method with 1000 bootstrapping replications. The Cmr1 (GenBank accession number ABI81496) in *C. heterostrophus* was used as an outgroup. The ML tree was used to estimate divergence times using the method of Reltime implemented in MEGA7.0.21. Two calibration points were used. That is, the node between Cmr1 in *C. lagenarium* and Pig1 in *M. grisea* was constrained to 299 million years ago (MYA) following the estimated divergence time (355 MYA) of *Colletotrichum* and *Alternaria*. Their divergence time estimates were derived from the TimeTree server (Kumar et al. 2022).

The selection pressures were performed using the CODEML program within the Phylogenetic Analysis by Maximum Likelihood (PAML) software package with the ML method (Yang 1997; Nielsen and Yang 1998; Yang et al. 2000; Yang and Bielawski 2000). A series of likelihood ratio tests (LRTs) were carried out to test for positive selection by comparing the null models (M0, M1a, M7) with their corresponding alternative models (M3, M2a, M8). The empirical Bayes theorem implemented in CODEML was used to calculate the posterior probability that a particular amino acid site belongs to a given selection class (neutral, deleterious or advantageous) (Yang 1997). Amino acid sites with a high probability coming from advantageous class of sites are more likely to be under positive selection.

The protein sequences of polyketide synthase, PksA, two α-hydrolases, AygA and AygB, scytalone dehydratase Brm1, two 1,3,6,8-tetrahydroxynaphthalen (T4HN) reductases, Brm2 and Brm3, and seven laccases, LccA, LccB, LccC, LccD, LccE, LccF, and LccG from *A. alternata* (JGI Protein IDs 111952, 115293, 105009, 105968, 111954, 112254, 112523, 114657, 115320, 114031, 111569, 110245, and 116332, respectively) (Gao et al. 2022) were used to search against the *A. solani* BMP0185 genome for identifying for corresponding orthologus genes. Using CmrA (UniProt accession number A0A075QC79) as a template, the structures of Asmr1 and Amr1 were predicted by SWISS-MODEL server (Waterhouse et al. 2018), visualized, and aligned by PyMOL (Schrödinger and DeLano, http://www.pymol.org/pymol).

### DNA manipulations, gene cloning, and DNA sequencing

Genomic DNA was extracted from mycelia using the fungal genomic DNA extraction kit (Solarbio, Beijing, China). Based on the sequence of the Cmr1 homolog (AGD accession number ASL_PT00349) in *A. solani*, a pair of oligonucleotide primers Asmr1F/Asmr1R was designed for the amplification of the entire coding region of *asmr1* from *A. solani* isolates. The amplified fragment was cloned into pTOPO-Blunt vector (Aidlab, Hong Kong, China) and confirmed by DNA sequencing. All primers used in this paper were listed in Supplemental Table S2.

### Transcriptional activation assay

The gene fragment amplified from pTOPO:Asmr1 (carrying the sequence ON962808) using the primers Asmr1-BD-*Eco*RIF/Asmr1-BD-*Sal*IR and a *Eco*RI, *Sal*I-linearized bait vector pGBKT7 (Clontech, Mountain View, CA, USA) were homologously recombined using the ClonExpress® MultiS One Step Cloning Kit (Vazyme, Nanjing, China) to obtain the construct pGBKT7:Asmr1 (intron). To delete the intron sequences, four gene fragments were amplified from pGBKT7:Asmr1 (intron) using the primers Asmr1-BD-*Eco*RIF/Asmr1-IN1-firstR, Asmr1-IN1-secondF/Asmr1-IN2-firstR, Asmr1-IN2-secondF/Asmr1-IN3-firstR, and Asmr1-IN3-secondF/Asmr1-BD-*Sal*IR, respectively. They were homologously recombined with a *Eco*RI, *Sal*I-linearized pGBKT7 vector to achieve the construct pGBKT7:Asmr1, which was confirmed by DNA sequencing. The transcriptional activation assay was performed following the instructions of the Yeast One-hybrid Kit (Clontech, Mountain View, CA, USA). Preparation and transformation of Y2H Gold competent cells were carried out following the instructions of the Yeast Transformation Kit (Coolaber, Beijing, China). The nutrient deficient medium was purchased from Coolaber (Beijing, China).

### RNA manipulations and quantitative real-time RT-PCR

Total RNA extraction from infected leaves of potato 0, 2, 4, 6, 8, 10, 12, 24, 36, 48, 72, and 96 h post-inoculation (hpi) with *A. solani* SH0806 was carried out using the FastePure® Plant Total RNA Isolation Kit (Vazyme, Nanjing, China). cDNA was synthesized using the ChamQ Universal SYBR qPCR Master Mix (Vazyme, Nanjing, China). The primers were designed to anneal to sequences in regions spanning at least one intron to ensure no genomic DNA disturbance except those for *aygB*, *brm3*, and *lccG* due to the lack of introns. Real-time detection was carried out on the CFX ConnectTM Real-Time PCR Detection System (Bio-Rad, Hercules, CA, USA) and real-time PCR data was analyzed using the program GENEX (Bio-Rad, Hercules, CA, USA). The relative expression levels of *asmr1* and thirteen genes including *pksA*, *aygA*, *aygB*, *brm1*, *brm2*, *brm3*, *lccA*, *lccB*, *lccC*, *lccD*, *lccE*, *lccF*, and *lccG* [JGI (https://mycocosm.jgi.doe.gov/Altso1/Altso1.home.html) with transcript IDs 115231, 121832, 121729, 112195, 115171, 120096, 117354, 116243, 119329, 111647, 1181013, 115914, and 114865, respectively] were standardized to those of *A. solani actin1* (GenBank accession number MK388241.1) and *ef1α* (GenBank accession number MN370901.1), respectively.

### Generation of the *asmr1* deletion construct

The deletion construct was made by replacement of the gene with a hygromycin B (HygB) resistance cassette. The HygB phosphotransferase gene (*hph*) was used as a selective marker. The gene replacement construct was produced by a double-joint PCR method (Yu et al. 2004) and consisted of PCR fragments of 5′ and 3′ flanking region sequences of *asmr1* overlapping within *hph*. The 5′ and 3′ flanking regions of *asmr1* were amplified from *A. solani* using Asmr1-5F/Asmr1-5R and Asmr1-3F/Asmr1-3R, respectively. The HygB resistance cassette was amplified from pAg1-H3 (Zhang et al. 2003) with hph1349F/hph1349R. The primer set Asmr1-nestF/Asmr1-nestR was used to amplify the fused knockout construct, which was confirmed by DNA sequencing.

### Generation of the complemented construct of the Δ*asmr1-2*

The Δ*asmr1-2* mutant was complemented with the wild-type (WT) allele under its native promoter. The gene fragment containing a 1497 bp 5′ flanking region and the coding region of *asmr1* without the stop codon was amplified from isolate SH0806 using the primer set Asmr1-CF/Asmr1-CR and verified by DNA sequencing. The confirmed gene fragment and the linearized pYF11-gfp-Gen vector (Chen et al. 2018) were co-transformed into yeast strain XK1-25 using the Yeast Transformation Kit (Coolaber, Beijing, China) to achieve the fusion construct through the yeast gap repair approach. The resulting construct, pYF11:Asmr1 was verified by DNA sequencing.

### Preparation of fungal protoplasts

Preparation of fungal protoplasts was conducted as previously described with a minor modification (Akamatsu et al. 1997). Details are as follows: Agar plugs containing WT *A. solani* (SH0806) or its mutants were cultured on potato dextrose agar (PDA) until spore sporulation. Spores were collected, transferred into potato dextrose broth (PDB), and shaken at 120 rpm at 28 °C for 1–2 days. Mycelia were harvested using miracloth (EMD Biosciences, Rockland, MA, USA), rinsed with sterilized ddH_2_O three times, and re-suspended in 50 ml of filter sterilized solution of enzymes containing 10 mg/ml lysing enzymes (Sigma, St. Louis, MO, USA) and 10 mg/ml Kitalase (Wako Pure Chemical Industries, Ltd., Osaka, Japan), which were dissolved in 0.7 M NaCl. The suspension was shaken at 70 rpm at 30 °C for 3–4 h, filtered by miracloth, and centrifuged at 3000 × g for 5 min. After the removal of the supernatant, the pellets of protoplasts were gently re-suspended with 0.7 M NaCl and centrifuged at 3000 × g for 5 min. Protoplasts were re-suspended with 1 ml of STC solution [1.2 M sorbitol, 10 mM Tris–HCl (pH 7.5), 10 mM CaCl_2_] and centrifuged at 3000 × g for 5 min. Pelleted protoplasts were re-suspended with an appropriate volume of STC solution to obtain a final solution containing 1 × 10^8^ protoplasts. The protoplasts were checked under the light microscope and the number of protoplasts was determined with a hemacytometer.

### Transformation of *A*. *solani* and molecular confirmation of transformants

*A. solani* protoplasts were transformed following the method of Akamatsu et al. (1997) with some modification. One to 5 µg plasmid DNA and 5 µl of heparin sodium (5 mg/ml) were mixed with 1 ml of STC solution containing 1 × 10^8^ protoplasts and incubated on ice for 30 min. One third amount of SPTC solution (STC solution containing 40% PEG 4000) was added into the mixture and incubated at room temperature for 20 min. Subsequently, 50 ml of RM (regeneration medium) (0.1% yeast extract, 0.1% casein enzymatic hydrolysate, 1 M sucrose) was added into the transformation mixture and incubated at 25 °C overnight followed by plating on PDA containing 100 µg/ml Hyg B. After incubating at 28 °C for 7–14 days, putative transformants were selected under two rounds of single-spore isolation. To obtain stable *hph* expressing putative transformants, single-spore isolated putative transformants were sub-cultured on PDA without HygB and PDA with HygB in turn for five generations. Selected transformants were verified by PCR with five primer pairs, H850F/H852R to check the presence of *hph*; Asmr1-InF/Asmr1-InR to check the presence of *asmr1*; Asmr1-nestF/Asmr1-nestR to check whether *asmr1* was replaced by *hph*; Asmr1-nestF/H855R to check the correct integration of HygB resistance cassette into the upstream region of *asmr1*; H856F/nestR to check the correct integration of HygB resistance cassette into the downstream region of *asmr1*. Southern blot hybridization according to the manufacturer’s instructions of the DIG-High Prime DNA Labeling and Detection Kit II (Roche, Indianapolis, IN, USA) followed by DNA sequencing using the primer pair HphCF/HphCR was performed to confirm the presence of one single copy of *hph* and the deletion of *asmr1* in PCR verified transformants. Integration of the WT *asmr1* allele under its native promoter into the Δ*asmr1-2* mutant genome was confirmed with PCR using the *asmr1* internal region primer pair Asmr1-InF/Asmr1-InR.

### Examination of colony growth and conidia

WT *A. solani* and its mutants from glycerol stocks were inoculated on PDA in the dark at 25 °C for 5 days. Agar plugs (5 mm in diameter) containing fresh mycelia on PDA were sub-cultured on PDA, PCA, or potato agar (PA) and PDA containing 0.2 g/L Congo red, 0.02% SDS (sodium dodecyl sulfate), 10 mM H_2_O_2_, 5 ppm mancozeb, 5 ppm chlorothalonil, 5 ppm azoxystrobin, 1 M KCl, 1 M NaCl, 1 M sorbitol, 10 mM FeSO_4_, 1.5 mM CuSO_4_ or 250 mM CaCl_2_ and incubated at 25 °C for 5 days. The diameter of each colony was measured using a Vernier caliper and the inhibitory rate of fungal mycelial growth was calculated. All experiments were conducted three times. Three replicates were used for each experiment. Significant differences in colony diameter and inhibitory rate were determined by one-way analysis of variance (ANOVA). Conidia were collected from PCA with sterilized ddH_2_O and examined for their morphology using a Nikon TE2000 inverted fluorescence microscope (Nikon, Miato, Japan).

### Extraction and detection of fungal melanin content

Extraction of fungal melanin was conducted following the method described by Yang et al. (2012) with modification. A quarter of 1 g of fresh mycelia was collected, transferred into 10 ml of 1 M NaOH solution, incubated at 100 °C water bath for 5 h, and filtered using one layer of filter paper. The filtrate was adjusted to pH to 2.0 using HCl, incubated at 4 °C overnight, and centrifuged at 6000 × g for 15 min. The pellets collecting melanin were dissolved in 5 ml of 1 M NaOH and the absorbance of the solution at 459 nm was measured by UV spectrophotometer. The melanin content (*Y*, mg/g) of fungal mycelia was calculated as the following formula: *Y* = (*X* + 0.0114)/0.0495, where *Y* represents the yield of fungal melanin and *X* indicates the absorbance value at 459 nm. Three replicates were used for the experiment. Significant differences in melanin yield were determined by ANOVA.

### Measurement of ROS

WT *A. solani* and its mutants from glycerol stocks were inoculated on PDA in the dark at 25 °C for 5 days. Agar plugs (5 mm in diameter) containing fresh mycelia were transferred to PDA and incubated at 25 °C for 3 days. Three days-old fungal cultures were stained with nitroblue tetrazolium (NBT) reagent (Beyotime Biotechnology, Shanghai, China) to detect the production of ROS.

### Plant infection assays

A total of 20 µl of suspension containing 1 × 10^5^ spores/ml from WT *A. solani* isolate SH0806 or its mutants was used to inoculate the detached leaves of potato. The inoculated leaves were placed in a plastic tray lined with wet paper towels, tightly fitted with a transparent plastic cover, and kept in a growth chamber at 25 °C under a photoperiod of 12 h. For quantitative real-time RT-PCR, leaves were harvested at 12 time points as described above, individually frozen in liquid nitrogen, and stored at –80 °C prior to RNA extraction. For pathogenicity test, the symptoms and necrotic lesion size were recorded at 14 days post-inoculation (dpi). The necrotic lesion size was calculated based on the square area (cm^2^) of the lesion. The experiment was performed three times. Each time had three technical repeats. Significant differences in lesion size were determined by ANOVA.

## Results

### Cmr1 homologs in *Alternaria* all contain one additional diverse fungal specific transcription factor motif

**Fig. 1:**
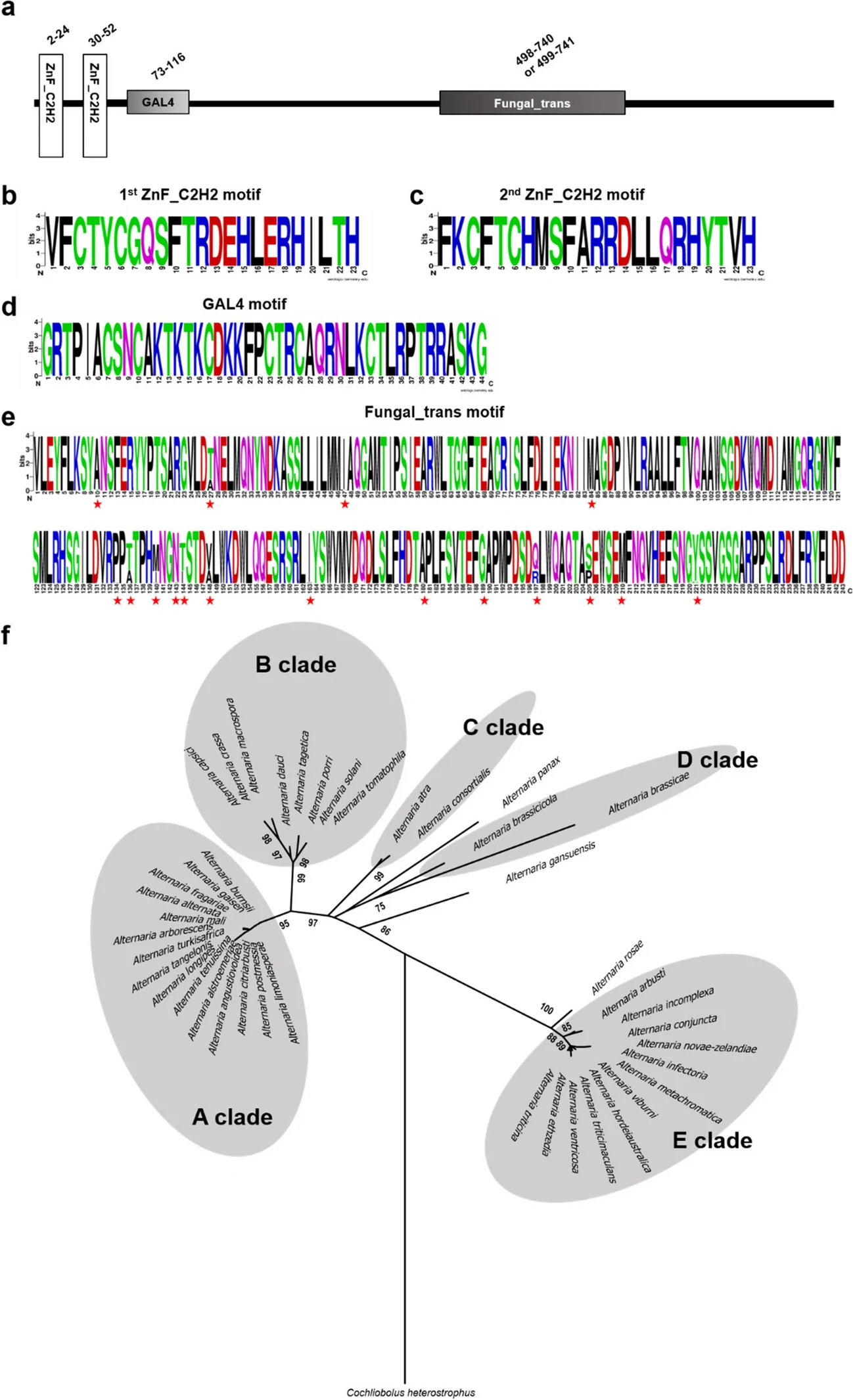
Structure and phylogeny of Cmr1 homologs in *Alternaria*. **a** Motifs of Cmr1 homologs in *Alternaria* are schematically shown. The motifs were predicted by SMART server at http://smart.embl-heidelberg.de. ZnF_C2H2 represents the Cys_2_His_2_ zinc finger motif (SM000355). GAL4 indicates the GAL4-like Zn(II)_2_Cys_6_ binuclear cluster DNA-binding motif (SM00066). Fungal_trans denotes the fungal specific transcription factor motif (PF04082). The numbers above each motif describes the amino acid locations of the motif at the full length protein. **b** Consensus sequence in the first ZnF_C2H2 motif, **c** in the second ZnF_C2H2 motif, **d** in the GAL4 motif, and **e** in the fungal_trans motif were estimated based on multiple sequence alignment of 42 Cmr1 homologs using WebLogo server at http://www.weblogo.berkeley.edu. The diverse amino acid sites are indicated by asterisks in red. The bigger the letter is, the more conserved the amino acid site is. **f** ML tree based on the predicted protein sequences of 42 Cmr1 homologs was constructed using MEGA7.0.21 and rooted with Cmr1 in *C. heterostrophus*. Confidence values higher than 70% shown at the nodes were obtained from 1000 bootstrap replications. The A, B, C, D, and E clades are shaded by gray

To identify Cmr1 homologs in the genus *Alternaria*, we mined publicly available *Alternaria* genomes representing 44 *Alternaria* species (Supplemental Table S3). After verification of the motifs, a total of 44 Cmr1 homologs (one per each species) including previously reported CmrA in *A. alternata* and Amr1 in *A. brassicicola* were identified (Supplemental Table S4 and Supplemental Figs. S1 and S2). Cmr1 homologs from *Alternaria carthami* and *Alternaria destruens* were excluded from further analysis due to the presence of gaps within their coding regions. Excluding three introns including two located in 5′ non-coding region (75/97/98/99 bp and 75/76/83/84/85 bp in length) and one in 3′ non-coding region (55/56/59 bp in length), all remaining 42 Cmr1 homologs ranged from 3027 to 3033 bp in length corresponding to 1009 to 1011 predicted amino acids (Supplemental Table S4). All 42 Cmr1 homologs contained the expected motifs located at N-terminus: two Cys_2_His_2_ zinc finger motifs (ZnF_C2H2, SM000355) at amino acid positions 2 to 24 and 30 to 52, respectively, as well as one GAL4-like Zn(II)_2_Cys_6_ binuclear cluster DNA-binding motif (GAL4, SM00066) at amino acid positions 73 to 116 (Fig. 1a). In addition, they all carried one fungal specific transcription factor motif (fungal_trans, PF04082) at the C-terminus (amino acid positions 498 to 740 or 499 to 741) (Fig. 1a). Weblogo analysis of motifs of Cmr1 homologs revealed that the amino acid sequences within each of two ZnF_C2H2 motifs and the GAL4 motif were completely conserved (Fig. 1b–d). In contrast, the amino acid sequences within the fungal_trans motif were diverse at 17 sites in Cmr1 homologs (Fig. 1e). Our data indicates that Cmr1 homologs in *Alternaria* contain one extra diverse fungal_trans motif except for three fully conserved characteristic motifs of Cmr1.

### Five clades of Cmr1 homologs emerge in *Alternaria*

To determine the evolutionary relationship of Cmr1 homologs in *Alternaria*, a phylogeny (phylogenetic tree) was generated based on alignment of their amino acid sequences using the ML method implemented in the program MEGA7.0.21 (Kumar et al. 2016). Forty-two Cmr1 homologs were classified into five closely related genetic groups, clades A, B, C, D, and E supported with high bootstrap values of 95, 99, 99, 75, and 88, respectively, and three individual branches (Fig. 1f). The clade A comprised 13 species including members of the *Alternaria* section *Alternata* and 2 species belonging to an unclassified *Alternaria* section, *Alternaria alstroemeriae* and *Alternaria fragariae*. The B clade was composed of 8 species, which were included in the *Alternaria* section *Porri*, such as *A. solani*. The C clade consisted of *Alternaria atra* and *Alternaria consortialis*, belonging to the *Alternaria* section *Ulocladioides*. The D clade also contained 2 species, *A. brasssicicola* from the *Alternaria* section *Brassicicola* and *Alternaria brassicae* from an unclassified *Alternaria* section. The E clade included 12 species, all from the *Alternaria* section *Infectoriae.* The results showed that the A, B, C, and D clades were more closely related to each other than to the E clade. In addition, the A clade was the most divergent one. Three species including *Alternaria panax*, *Alternaria gansuensis*, and *Alternaria rosae* formed individually three branches. Note that, phylogenetic analysis placed CmrA in *A. alternata*, the Cmr1 homolog in *A. solani*, and Amr1 in *A. brassicicola* in A, B, and D distinct clades, respectively. When coupled with the divergence in host range of *A. alternata*, *A. solani*, and *A. brassicicola*, it appears that these Cmr1 homologs have been under selection for functional divergence during the interaction with their hosts or living environments. Most surprising, the phylogeny of Cmr1 homologs followed the authoritative classification system of the genus *Alternaria* established by Woudenberg et al. (2013). It implies that Cmr1 can be used as a potential taxonomic marker of the genus *Alternaria*, which can distinguish at least five groups of *Alternaria* species.

### The A and D clades diverge latest and earliest, respectively

**Fig. 2:**
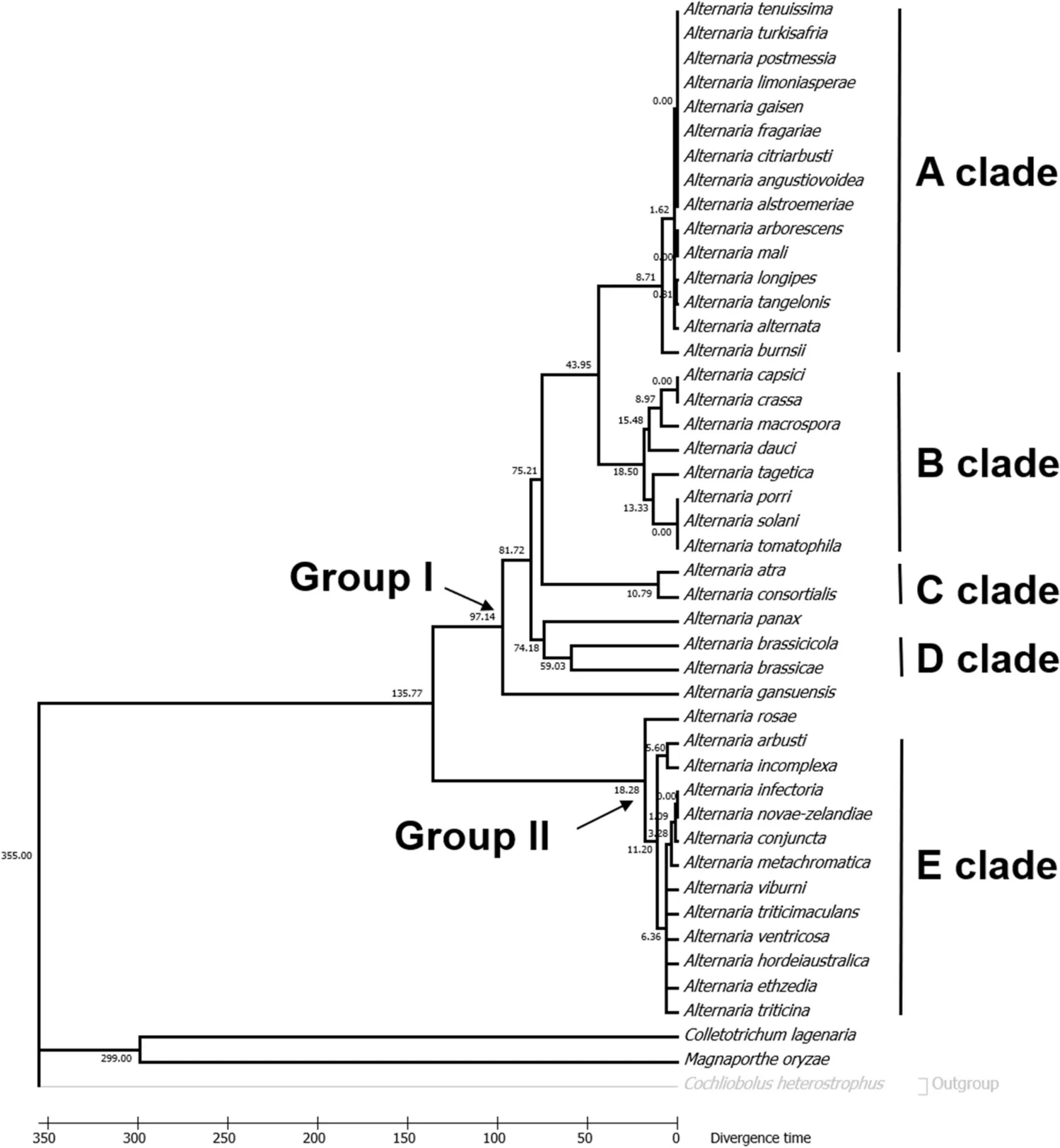
Dated phylogeny of Cmr1 homologs in *Alternaria*. The topology was based on ML analysis of the amino acid sequences of 42 Cmr1 homologs. The Cmr1 in *C. heterostrophus* was used as an outgroup. Molecular-clock estimates of median divergence times using the Reltime ML method implemented in MEGA7.0.21 and two calibration points (see “Materials and methods” for details) are given on each node in MYA (million years ago). The scale is in MYA

To investigate how divergent Cmr1 homologs appeared in *Alternaria*, we estimated the divergence times for all branching points in the phylogeny of 42 Cmr1 homologs using the Reltime ML method implemented in the Timetree Wizard window of the program MEGA7.0.21 (Kumar et al. 2016) and two calibration points (see “Materials and methods” for details). As seen in Fig. 2, the last common ancestor of group I and group II of the 42 Cmr1 homologs had an estimated age of 135.77 MYA (million years ago). The origin of group I including the A, B, C, and D clades as well as two individual branches, *A. panax* and *A. gansuensis*, was estimated to be 97.14 MYA. The origin of group II was inferred to be 18.28 MYA, suggesting that group II emerged later than group I. In addition, among the five clades, the D clade diverged earliest (59.93 MYA), followed by the B clade (18.5 MYA), the E clade (11.2 MYA), and the C clade (10.79 MYA), and the A clade diverged latest (8.71 MYA).

### Cmr1 homologs in *Alternaria* are under positive selection

The value of the ratios of nonsynonymous to synonymous nucleotide substitution rates in protein-coding DNA sequences (*ω* = *d*_N_/*d*_S_) is the indicator of the type of selection pressures (Yang and Bielawski 2000). The *ω* values of 1, < 1, and > 1 represent neutral, positive, and negative selection, respectively. To examine the selection pressure underlying sequence diversification in Cmr1 homologs in *Alternaria*, we analyzed the 42 Cmr1 homologs using the ML method (a statistical distribution to model *ω* variations among sites along the protein) implemented in the program CODEML in the PAML software package (Yang 1997). First, LRTs were performed to test for selection. In details, three pairs of ML models of codon substitution such as M3/M0, M8/M7, and M2a/M1a were used (Yang et al. 2000) (Table 1). Both the discrete model M3 and the selection model M2a did not identify any amino acid sites under positive selection. In contrast, the model M8 suggested that 0.2% of sites were under positive selection with *ω* = 1.659. The LRT for comparing M8 with M7 was 2△L = 2 × [–13151.053–(–13,156.009)] = 9.912, which is greater than the χ^2^ critical value (9.21 at the 1% significance level, with degrees of freedom (df) = 2) (Table 1). Thus, the null model M7 which does not allow for the presence of positive selection sites with *ω* > 1 was rejected, indicating that the detected positive selection under the discrete M8 was statistically significant at *P* value < 0.05. Second, the amino acid sites under positive selection when LRT tested the presence of sites with ω > 1 were identified. This was achieved by using the empirical Bayes theorem to calculate the (posterior) probability. We identified three sites including 171 S, 927 G, and 966 S implicated as being under positive selection with the confidence level of 98%, 78.6%, and 69.5%, respectively, under model M8. Interestingly, none of the three identified positive selection sites were located in any of the four identified protein motifs of Cmr1 homologs (Fig. 1a and Supplemental Fig. S3). The results suggest that positive selection has affected the evolution of Cmr1 homologs in *Alternaria*.

**Table 1:** Likelihood ratio test results

Model	Estimates of parameters	InL^a^	Positive selection sites^b^	Model comparison	LRT statistic (2△L^c^; df^d^; *P* value)
M0	ω = 0.040	− 13,233.007	Not allowed		
M3	*P*_0_ = 0.898, *P*_1_ = 0.100, *P*_2_ = 0.002, ω_0_ = 0.014, ω_1_ = 0.275, ω_2_ = 1.868	− 13,150.160	None	M0 vs. M3	82.847; 4; 0
M1a	*P*_0_ = 0.973, *P*_1_ = 0.027, ω_0_ = 0.027, ω_1_ = 1.000	− 13,173.877	Not allowed		
M2a	*P*_0_ = 0.973, *P*_1_ = 0.027, *P*_2_ = 0.000, ω_0_ = 0.027, ω_1_ = 1.000, ω_2_ = 34.926	− 13,173.664	None	M1a vs. M2a	0.213; 2; 0.90
M7	*P* = 0.146, *q* = 2.908	− 13,156.009	Not allowed		
M8	*P*_0_ = 0.998, *P* = 0.164, *q* = 3.556, *P*_1_ = 0.002, ω = 1.659	− 13,151.053	171 S (98%); 927 G (78.6%); 966 S (69.5%)	M7 vs. M8	9.912; 2; 0.007

The χ^2^ critical value = 9.21 and 13.28 at 1% significance level with degrees of freedom 2 and 4, respectively

^a^*InL*, log likelihood value

^b^Amino acid sites inferred to be under positive selection with a posterior probability in brackets predicted with Empirical Bayes statistics (Yang et al. 1997)

^c^2*△*L, 2 (InL alternative hypothesis-InL null hypothesis)

^d^df, degrees of freedom

### The Cmr1 homolog is fully conserved at the amino acid level in *A. solani* isolates

To study whether there is sequence polymorphism in Cmr1 homologs in *A. solani*, we amplified, cloned, and sequenced Cmr1 homologs from ten *A. solani* isolates from different potato or tomato producing areas in China including the northeast (Suihua, Keshan, Daqing, Jiagedaqi District, and Benxi), the northwest (Dingxi), the north (Bejing and Shandong), and the south (Jiansu) (Supplemental Table S1). Multiple alignments of the nucleotide sequences of these ten Cmr1 homologs and two additional Cmr1 homologs from isolates BMP 0185 and altNL03003 (The Netherlands) revealed that polymorphisms were detected at 10 of 3277 nucleotides (data not shown). All polymorphic sites were located in the exon sequences," however, they could not cause amino acid changes. The results imply that Cmr1 homologs at amino acid levels are completely conserved and appear to undergo negative selection in *A. solani* isolates from different geographic locations.

### Asmr1 exhibits transcriptional activity in yeast

**Fig. 3:**
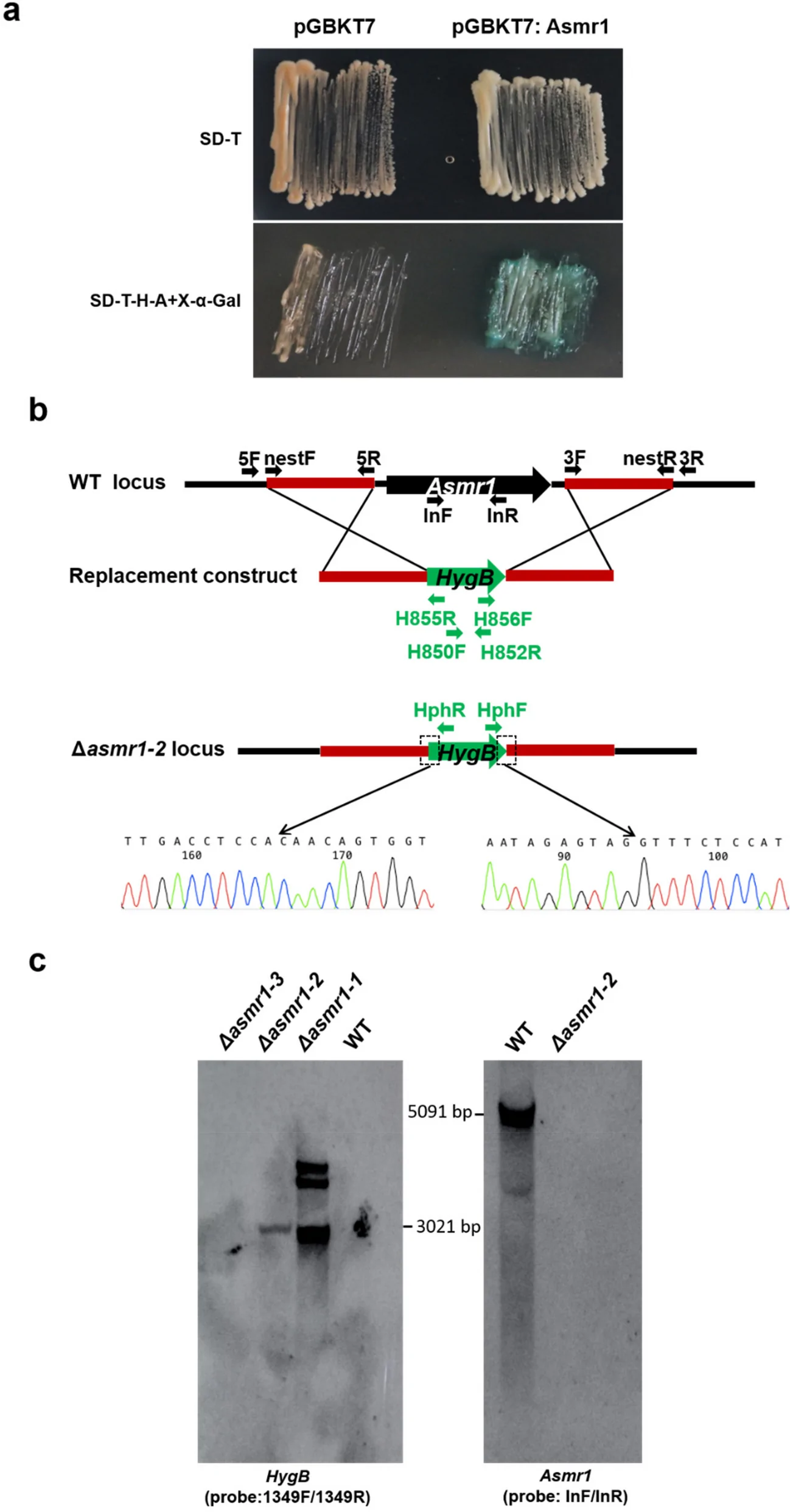
Transcriptional activation analysis of Asmr1 in yeast and verification of *asmr1* deletion and complementation mutants. **a** Yeast Y2H GOLD strains carrying the pGBKT7 vector (negative control) and pGBKT7:Asmr1 construct grew on SD-T and SD-T-H-A + X-α-Gal medium. **b** Schematic diagram of replacement of the *asmr1* coding region with a HygB resistance cassette. The lowest panel shows the sequencing results of the Δ*asmr1-2* mutant with the primer pair, HphF/HphR. **c** Southern blot analysis of the *asmr1* deletion mutants with the *hph* probe amplified with the primer set Hph1349F/Hph1349R and the *asmr1* probe amplified with the primer pair Asmr1-InF/Asmr1-InR. Abbreviations: WT = wild-type *A. solani*; Δ*asmr1-1*, Δ*asmr1-2*, Δ*asmr1-3*, *asmr1* deletion mutants; Δ*asmr1-2C*, complementation mutant Δ*asmr1-2*:*asmr1*

To link the sequence analysis and the function of the Cmr1 homolog in *A. solani*, we validated the transcription factor activity of the Cmr1 homolog in *A. solani* using the transcription activation assay. The construct pGBKT7 carrying the open reading frame of Asmr1 (we designated the Cmr1 homolog amplified from isolate SH0806 *A. solani* melanin regulation transcription factor 1 or Asmr1) was first achieved. After transforming into the yeast strain Y2H GOLD, both empty pGBKT7 and pGBKT7:Asmr1 constructs enabled Y2H GOLD to grow on selective dropout medium without tryptophan (SD-T). However, only the strain Y2H GOLD carrying pGBKT7:Asmr1 construct could grow on SD medium without tryptophan, histidine, and adenine (SD-T-H-A). In addition, transformed colonies of pGBKT7:Asmr1 exhibited a blue color on SD-T-H-A medium supplemented with 5-bromo-4-chloro-3-indolyl-β-D-galactopyranoside (X-α-Gal) (Fig. 3a). The results indicate that Asmr1 is able to activate the transcription of reporter genes including *His3*, *Ade2*, and *Mel1*, supporting that Asmr1 is a functional transcription factor.

### Asmr1 is involved in spore development and melanin biosynthesis

To further investigate the function of Asmr1, a deletion mutant of the *asmr1* gene was constructed by replacing the coding region with a HygB resistance cassette (Fig. 3b). Three HygB resistant transformants, namely, Δ*asmr1-1*, Δ*asmr1-2*, and Δ*asmr1-3* were obtained. The results of PCR analysis followed by Southern hybridization and DNA sequencing showed that Δ*asmr1-1* was an ectopic insertion mutant, whereas Δ*asmr1-2* and Δ*asmr1-3* were correct *asmr1* replacement mutants (Supplemental Fig. S4a, Fig. 3b, c). We complemented the Δ*asmr1-2* mutant with the WT allele. PCR and RT-PCR analyses confirmed that Δ*asmr1-2C* was a correctly *asmr1* complemented transformant of mutant Δ*asmr1-2* (Supplemental Fig. S4b).

**Fig. 4:**
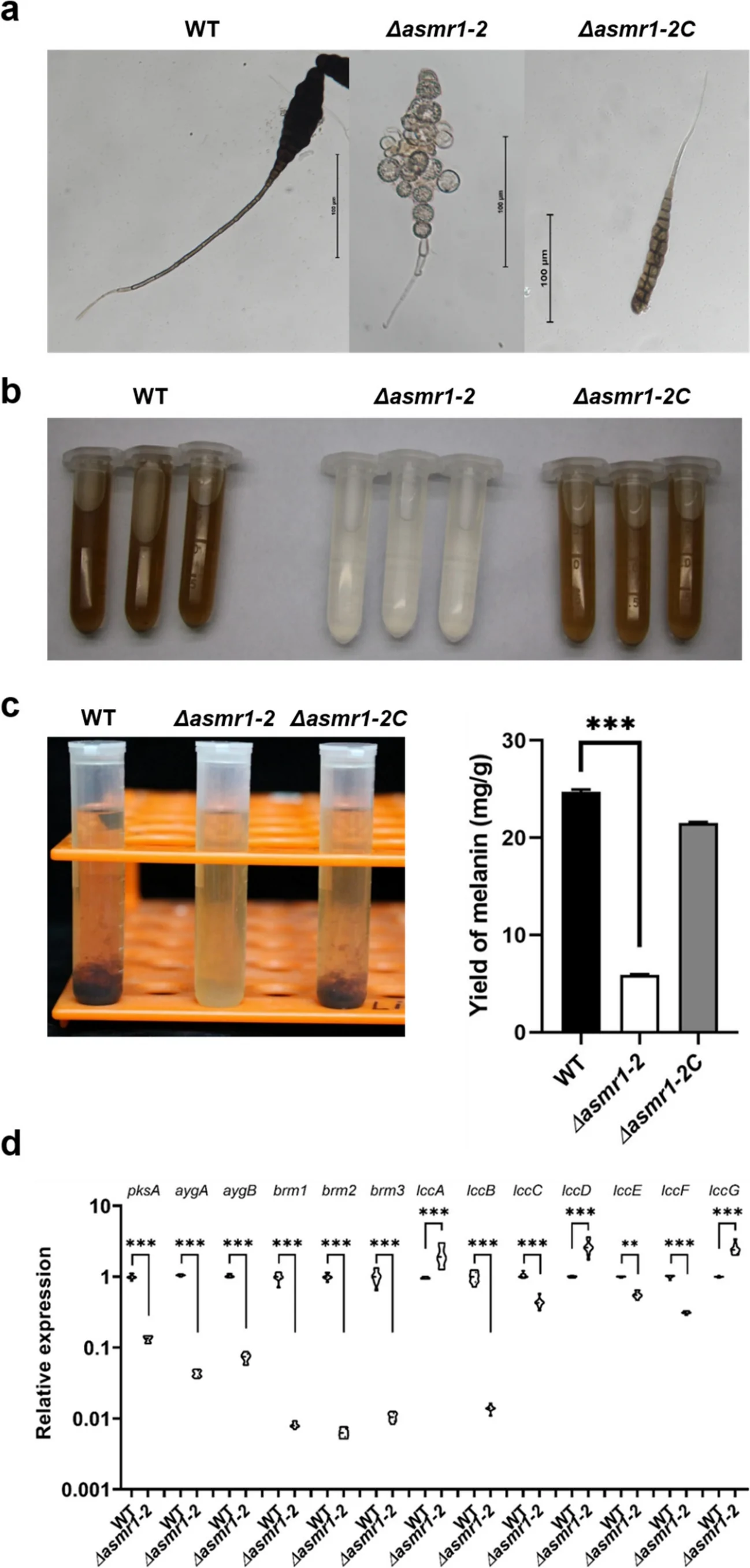
Effect of *asmr1* deletion on conidial morphology and melanin production in *A. solani*. **a** Comparison of conidial morphology between mutants and wild-type on PCA. **b** Crude extracts of melanin of wild-type and its mutants. **c** Precipitation (left panel) and yield (right panel) of crude extracts of melanin of wild-type and its mutants. The melanin content (*Y*, mg/g) of fungal mycelia was calculated as the following formula: *Y* = (*X* + 0.0114)/0.0495, where *Y* represents the yield of fungal melanin, and *X* indicates the absorbance value at 459 nm. **d** Expression levels of *A. solani* orthologs of genes involved in DHN-melanin biosynthesis in wild-type and the Δ*asmr1-2* mutant and normalized to *ef1α* expression levels. Error bars present standard deviations of three biological replicates. The significant difference in yield of melanin and relative expression was determined by one-way analysis of variance (ANOVA). **P < 0.01; ***P < 0.001 (comparison with wild-type). Abbreviations: WT, wild-type *A. solani*; Δ*asmr1-2*, *asmr1* deletion mutant; Δ*asmr1-2C*, complementation mutant Δ*asmr1-2*:*asmr1*

We determined the effect of *asmr1-*deficiency on conidia in *A. solani*. The WT and the Δ*asmr1-2* and Δ*asmr1-2C* mutants all produced conidia on PCA and showed little difference in conidia production (data not shown). However, the conidia of the Δ*asmr1-2* mutant were pale, with non-black color, and deformed, in comparison with the dark brown to black color and intact conidia of the WT and the Δ*asmr1-2C* mutant (Fig. 4a and Supplemental Fig. S5). In certain individual conidia of the Δ*asmr1-2* mutant, some part was colorless or white while another part looked brownish. It is worth noting that the beak of the conidia of the Δ*asmr1-2* mutant became shorter compared with the WT and the Δ*asmr1-2C* mutant (Fig. 4a and Supplemental Fig. S5). We subsequently measured the melanin content in *A. solani*. The color of the crude melanin extract from the Δ*asmr1-2* mutant looked very light and was close to transparent. In contrast, that from the WT and the Δ*asmr1-2C* mutant displayed brown or dark brown color (Fig. 4b, c). Compared to WT, the melanin yield of the Δ*asmr1-2* mutant significantly decreased (Fig. 4c). Consequently, we quantified the transcript levels of *A. solani* orthologs of 13 DHN-melanin biosynthesis-related genes in fungi. Compared to WT, ten genes including *pksA*, *aygA*, *aygB*, *brm1*, *brm2*, *brm3*, *lccB*, *lccC*, *lccE*, and *lccF* were all significantly less expressed in the Δ*asmr1-2* mutant, whereas the three laccase genes *lccA*, *lccD*, and *lccG* were all significantly upregulated in the Δ*asmr1-2* mutant (Fig. 4d), suggesting that the expression of the former genes was positively dependent on Asmr1 and that of the latter negatively controlled by Asmr1. Altogether, the results indicate that Asmr1 is involved in conidial morphogenesis and the regulation of melanin biosynthesis in *A. solani*.

### Decreased pathogenicity of the Δ*asmr1*-*2* mutant in potato

**Fig. 5:**
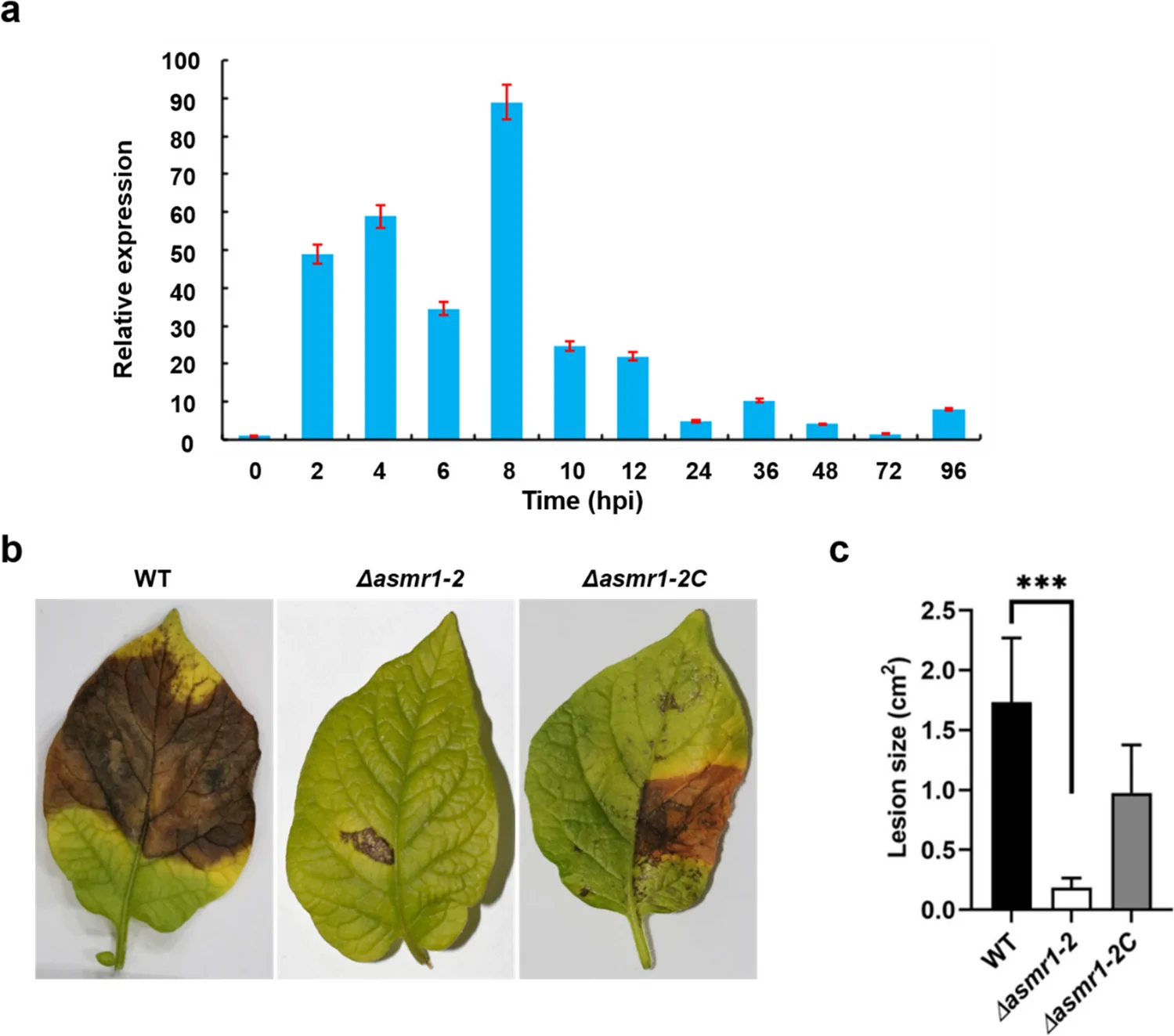
Time course expression of *asmr1* during infection of potato by *A. solani* and pathogenicity assay of the *asmr1* deletion mutant on potato. **a** Expression levels of *asmr1* at 0, 2, 4, 6, 8, 10, 12, 24, 36, 48, 72, and 96 hpi of susceptible potato cv. Zaodabai infection with *A. solani* isolate SH0806 and normalized to *A. solani actin1* expression levels. Error bars present standard deviations of three biological replicates. **b** Inoculated leaves of susceptible potato cv. Favorita with wild-type, *asmr1* deletion mutant, and complementation mutant. **c** The area size (cm.^2^) of necrotic lesion caused by inoculation at 14 dpi. Values represent means from three independent experiments with three replicates each. The significant difference in lesion size was determined by ANOVA. ***P < 0.001 (comparison with wild-type). Abbreviations: WT, wild-type *A. solani*; Δ*asmr1-2*, *asmr1* deletion mutant; Δ*asmr1-2C*, complementation mutant Δ*asmr1-2*:*asmr1*

To test whether the Asmr1 is involved in the infection process of *A. solani* in potato, we determined the expression of *asmr1* during infection using quantitative real-time RT-PCR. The *asmr1* gene showed elevated levels of expression throughout early infection stage from 0 to 12 hpi (Fig. 5a), suggesting that Asmr1 may involve in the infection process of *A. solani* in potato. This finding promoted us to perform a pathogenicity test. Two weeks after inoculation, the necrotic lesions caused by the Δ*asmr1-2* mutant were significantly smaller than those caused by the WT (Fig. 5b, c). The results suggest that the pathogenicity of the Δ*asmr1-2* mutant decreases in potato.

### Responses of the Δ*asmr1*-*2* mutant to cell wall and fungicide stresses

**Fig. 6:**
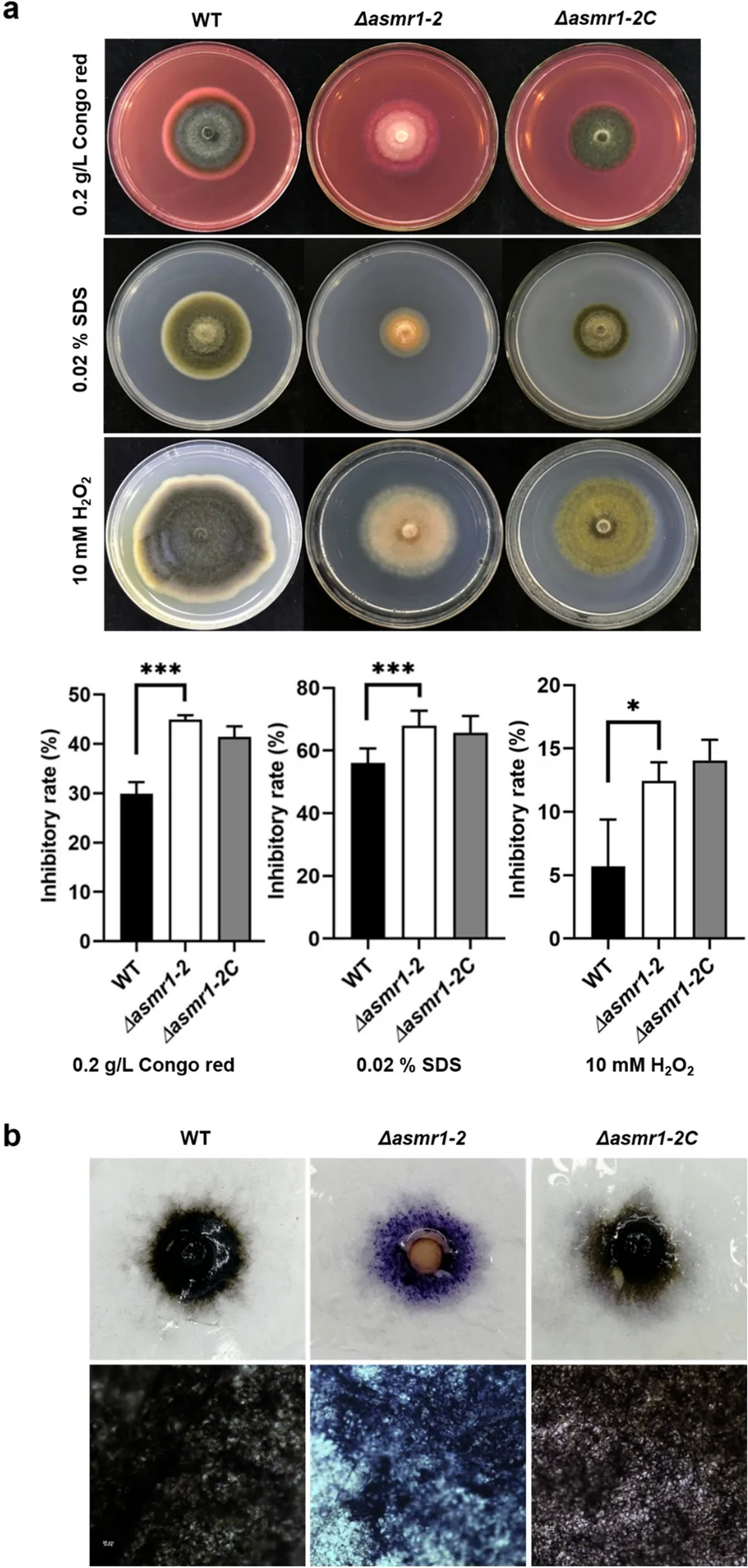
Effects of *asmr1* deletion on *A. solani* responses to cell wall and oxygen stressors and determination of ROS contents. **a** Comparison of mycelial growth inhibitory rate between mutants and wild-type on PDA in the presence of indicated chemicals. Values represent means from three independent experiments with three replicates each. The significant difference in growth inhibitory rate was determined by ANOVA. *P < 0.05; ***P < 0.001 (comparison with wild-type). **b** Colony (upper panel) and hyphae (lower panel) of 3-day-old WT, *asmr1* deletion mutant, and complementation mutant on PDA stained with NBT reagent. Abbreviations: WT, wild-type *A. solani*; Δ*asmr1-2*, *asmr1* deletion mutant; Δ*asmr1-2C*, complementation mutant Δ*asmr1-2*:*asmr1*

Melanin plays a role in protecting fungi against a diverse array of stresses (Cordero and Casadevall 2017). To this end, we examined the effect of stressors including cell wall inhibitors, oxygen stressor, fungicides, osmotic stressors, and metal ions on the Δ*asmr1-2* mutant. The mycelial growth of the Δ*asmr1-2* mutant was significantly inhibited in the presence of Congo red or SDS (Fig. 6a), suggesting that Asmr1 may be involved in the regulation of the cell-wall-related pathway in *A. solani*. Similarly, the mycelial growth of the Δ*asmr1-2* mutant was also significantly reduced on the medium containing 10 mM H_2_O_2_ (Fig. 6a)_._ This is likely due to the increase of ROS in the Δ*asmr1-2* mutant, which results in a counterbalance between ROS and H_2_O_2_. Thus, we decided to examine the ROS content in the Δ*asmr1-2* mutant through staining 3-day-old fungal cultures with NBT reagent. NBT reacts with superoxide anion to form blue or blue purple formazan. The higher the content of superoxide anion, the darker the color (Halliwell and Gutteridge 1985). As seen in Fig. 6b, the entire colony and hyphae of the Δ*asmr1-2* mutant exhibited a blue purple color, whereas both WT and the Δ*asmr1-2C* mutant changed not in color. The results indicate that the ROS content in the Δ*asmr1-2* mutant is much higher than that of WT. Perhaps, without protection from melanin, *asmr1*-deficient *A. solani* increases the ROS content to combat against external adverse environmental conditions.

**Fig. 7:**
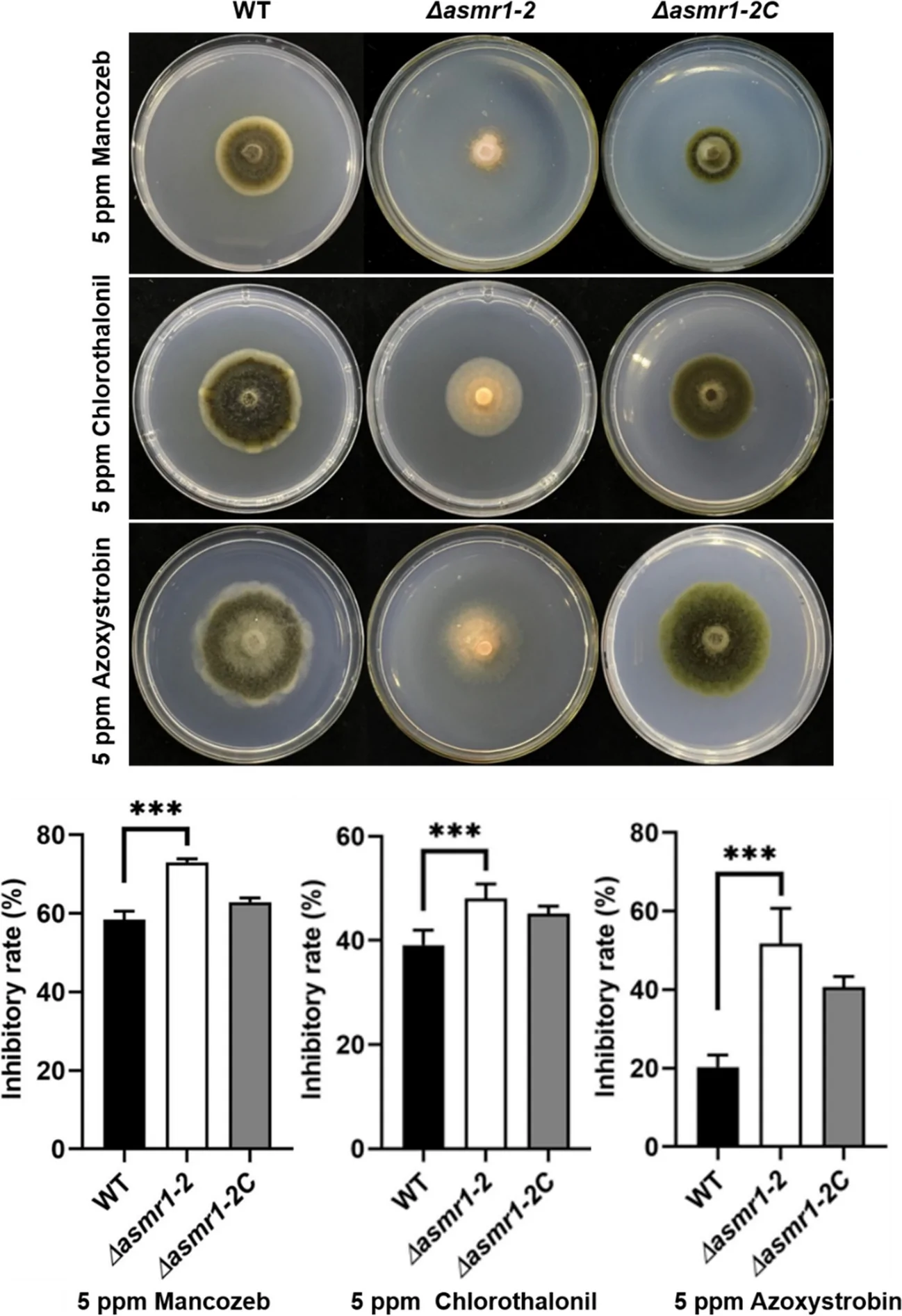
Effects of the *asmr1* deletion on *A. solani* responses to three fungicides. Comparison of mycelial growth inhibitory rates between mutants and wild-type on PDA in the presence of indicated fungicides. Values represent means from three independent experiments with three replicates each. Significant differences in growth inhibitory rates were determined by ANOVA. ***P < 0.001 (comparison with wild-type). Abbreviations: WT, wild-type *A. solani*; Δ*asmr1-2*, *asmr1* deletion mutant; Δ*asmr1-2C*, complementation mutant Δ*asmr1-2*:*asmr1*

Three economic and practical fungicides including mancozeb, chlorothalonil, and azoxystrobin for the prevention and control of potato early blight were used in this study. Compared to WT, the Δ*asmr1-2* mutant showed a significant decrease in the mycelial growth on the medium supplemented with 5 ppm of mancozeb, chlorothalonil, or azoxystrobin (Fig. 7). These results indicate that the deletion of *asmr1* results in the reduced tolerance of *A. solani* to these three fungicides. It also indirectly suggests that the effect of mancozeb on preventing and controlling potato early blight is better than that of chlorothalonil and azoxystrobin.

The mycelial growth of the Δ*asmr1-2* mutant was comparable to that of the WT and Δ*asmr1-2C* mutant on the medium containing KCl, NaCl or sorbitol (Supplemental Fig. S6a). Since KCl, NaCl, and sorbitol are related to fungal osmotic stress, we speculate that Asmr1 may not be involved in the regulation of the osmotic stress pathway in *A. solani*. In addition, the mycelial growth of the WT and of the Δ*asmr1-2* and Δ*asmr1-2C* mutants were completely inhibited on the medium supplemented with Fe^2+^ (Supplemental Fig. S6b). The colony size was not significantly different between the WT and the Δ*asmr1-2* mutant under Cu^2+^ stress. However, the Δ*asmr1-2* mutant showed a significant reduction in the mycelial growth on the medium containing Ca^2+^. Altogether, it suggests that Asmr1 may be involved in the regulation of the *A. solani* response to Ca^2+^, but not to Fe^2+^ and Cu^2+^.

We also determined the effect of *asmr1*-deficiency on vegetative growth of the WT and the Δ*asmr1-2*, and Δ*asmr1-2C* mutants on PDA, PCA, or PA (Supplemental Fig. S7). The Δ*asmr1-2* mutant displayed orange or near pink and light orange color (with less dense aerial hyphae) on PDA and PCA, respectively, compared to the black-colored WT. On PA lacking sugar, WT changed from black on the medium containing sugar to gray, while the Δ*asmr1-2* mutant remained little changed. Compared to the WT, the colony size in the Δ*asmr1-2* mutant was significantly smaller on PDA, PCA, or PA. The phenotype was comparable for both the Δ*asmr1-2C* mutant and the WT. The results suggest that Asmr1 affects the vegetative growth in *A. solani*.

### Both Asmr1 and Amr1 share highly similar structures with CmrA

**Fig. 8:**
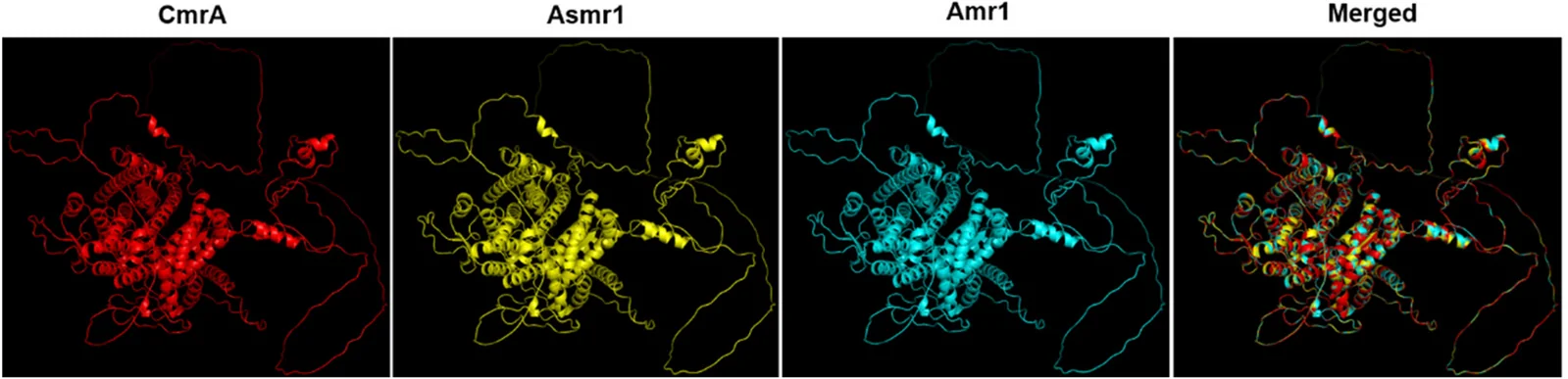
The structures of CmrA, Asmr1, and Amr1. The protein structures of Asmr1 and Amr1 were predicted by SWISS-MODEL server at http://swissmodel.expasy.org using CmrA (UniProt accession number A0A075QC79) as a template. The structures were visualized and aligned using the PyMOL software

Through multiple sequence alignment of Asmr1, Amr1, and CmrA, a total of 39 polymorphic amino acid sites were identified (data not shown). Therefore, we predicted the protein structures of Asmr1 and Amr1 through the SWISS-MODEL server (Waterhouse et al. 2018) using the public available protein structure of CmrA as a template. As seen in Fig. 8, both Asmr1 and Amr1 folded into a structure highly similar to CmrA. The alpha-helix, beta-sheet, and random coil were accounted for 34.4%, 3%, and 53.1% of the entire protein structure, respectively. Six and two out of 39 polymorphic sites were located in an alpha-helix or a beta-sheet while the remaining 31 sites were in random coils. Furthermore, two of six polymorphic sites in alpha-helixes were located in the fungal_trans motif. Taken together, the results suggest that structural conservation occurs in Asmr1, Amr1, and CmrA.

## Discussion

In this study, we found that Cmr1 homologs were present in all examined *Alternaria* species and defined their gene structures with regard to consensus sequences at three intron junctions. This will eventually contribute to future prediction, identification, and annotation of Cmr1 homologs in other *Alternaria* species. Furthermore, identification of three completely conserved motifs suggests that the functional conservation of these motifs occurs in Cmr1 homologs. Most notably, one extra diverse fungal_trans motif was identified in Cmr1 homologs in *Alternaria*. However, until now, not all identified Cmr1s and their homologs in other fungi carry this motif. The motif is present in Cmr1 of *C. heterostrophus*, Bmr1, and StMR1, but not in Cmr1 of *C. lagenaria*, Pig1, BcSMR1, Zmr1, and CgCmr1 (Supplemental Fig. S8). It is not clear whether the fungal_trans motif originally existed in Cmr1 or was obtained during evolution. The phylogeny of eleven described Cmr1 homologs including those from other fungi and Asmr1, Amr1, and CmrA revealed that the six Cmr1 homologs with fungal_trans motif from six different fungal species belonging to four distinct genera clustered together to form one clade (Supplemental Fig. S8b). Given the fungal_trans motif sequences were polymorphic, therefore, we compared the motif structures of the six Cmr1 homologs. All fungal_trans motifs of the six Cmr1 homologs folded into a similar structure (Supplemental Fig. S9a, b, c). Despite the different morphologies of these six species, as well as the taxonomic status of four different genera, Cmr1 homologs still share similarities among the six species, such as the close phylogenetic relationship, the similar gene structure, that all carry the four identified protein motifs, and similar folded structures of the fungal_trans motif. We speculate that Cmr1 homologs may have undergone divergent evolution in different fungi. This is likely due to the long period of selection pressure from their ecological niches and hosts.

Due to the large differences in the morphology, identification of *Alternaria* spp. is usually not correct only based on one single gene. However, the reliability in *Alternaria* spp. identification using four to five genes is high (Woudenberg et al. 2013). We found here that Cmr1 could distinguish different groups of *Alternaria* spp., indicating that Cmr1 has potential application values in taxonomy. It therefore not only augments the known function of melanin, but also expands the identification methods of *Alternaria* spp., that is, Cmr1 might be used as a novel taxonomic marker, just like the secondary metabolite toxin (Andersen et al. 2008; Patriarca et al. 2019).

Here, we show that positive selection has promoted divergence of Cmr1 homologs in *Alternaria* at three amino acid sites. These three positive selection sites were found to be located in random coils, which may alter the secondary structure of Cmr1. To rapidly adapt to novel and changing environments for increasing the survival probability, biological populations need to maintain new beneficial mutations or a standing genetic variation of adaptive traits (Messer and Petrov 2013). Melanization is such an adaptive trait. It is known that transcription factors that regulate expression of effectors in plant-pathogen interactions, secreted enzymes, and secondary metabolites, are required for the adaption of plant pathogenic fungi to the host environment (van der Does and Rep 2017). Thus, adaptive changes in the coding region of Cmr1 homologs in *Alternaria* might be responsible for the regulation of the variation in melanin accumulation. It would allow fungal populations to adapt and specialize on new hosts, or to adapt/tolerate to stress conditions. Future functional analyses, such as site-directed mutagenesis and identification of host targets of Cmr1 could be explored to help understanding the nature of adaptive changes in Cmr1 homologs in *Alternaria*.

Asmr1 was confirmed to have transcriptional activation activity and be involved in the melanin biosynthesis pathway. Noticeably, the deletion of *asmr1* did not completely abolish melanin production, suggesting the presence of a crosstalk between melanin biosynthesis and other signaling pathways regulating melanin. The MAP kinase (MAPK) pathways are critical for fungi to regulate responses to environmental stimuli (Zhang et al. 2021). Three conserved MAPK pathways including the cell wall integrity (CWI), invasive growth (IG), and high osmolarity growth (HOG) pathways have been well characterized in plant fungal pathogens (Zhang et al. 2021). Indeed, one IG pathway gene, *AbSte7* in *A. brassicicola* and one CWI pathway gene, *AaSLT2* in *A. alternata* are required for melanin biosynthesis (Yago et al. 2021; Xu et al. 2016). In addition, *AaSet2* encoding a histone methyltransferase is involved in melanin accumulation (Meng et al. 2022). Histone methylation is known to be vital for transcriptional regulation and diverse biological processes. We predict that the incomplete decrease of melanin accumulation in the Δ*asmr1-2* mutant could be due to the regulation role from other melanin-related signaling pathways, such as the MAPK pathway.

Asmr1 also affected other biological processes of *A. solani*. After the deletion of *asmr1*, the structure of certain conidia of *A. solani* became abnormal and the numbers of transverse and longitudinal septa of conidia also changed. This finding is similar to results presented in previous reports about the involvement of DHN-melanin in regulating longitudinal septal number and cell wall integrity of *A. alternata* conidia (Yago et al. 2021; Kheder et al. 2012) and that of CmrA in controlling the spore morphology and spore formation of *A. alternata* (Fetzner et al. 2014). However, the deletion of *amr1* in *A. brassicicola* has no effect on the conidial morphology (Cho et al. 2012). It suggests that the functional divergence of spore morphology occurs in Cmr1 homologs in *Alternaria*. In addition, we found that there were some pigments remaining in conidia, although the melanized degree of conidia and hyphae decreased after the deletion of *asmr1*. A recent study reported that many proteins including the CmrA transcription factor, which are required for the DHN-melanin biosynthesis pathway, are also involved in fungal toxin production via the perylene quinones biosynthesis pathway in *A. alternata* (Gao et al. 2022). Therefore, we speculate that Asmr1 may be a global transcription factor participating in multiple pathways.

Except for controlling melanin biosynthesis and morphogenesis, Asmr1 was required for the resistance to Congo red or SDS. It is critical for plant fungal pathogens to maintain the cell wall integrity and stability for disease establishment within the host (Zhang et al. 2021). Melanin is located within the fungal cell wall (Thomma 2003). The absence of Asmr1 might affect the cell wall integrity and stability of *A. solani*, further influencing the tolerance of *A. solani* to these cell wall stressors. Plant pathogenic fungi secrete a series of cell wall-degrading enzymes (CWDEs) during infection (Kubicek et al. 2014). Some researches indicate that the type and activity of fungal CWDEs is associated with metal ions, such as Ca^2+^, Zn^2+^, and Mg^2+^ etc. (Piero and Pascholati 2000; Martino et al. 2000; Sucheta et al. 2001). For instance, Ca^2+^ inhibits the activity of polygalacturonase in *Fusarium oxysporum* f. sp. *vasinfectum* infecting cotton (Piero and Pascholati 2000). One possible explanation for the observed phenotype of the decreased tolerance to Ca^2+^ in the Δ*asmr1-2* mutant could be that the deletion of* asmr1* might affect the cell wall integrity of *A. solani*, leading to a change of cell wall permeability, facilitating the easier attack of certain CWDEs by Ca^2+^. Therefore, this would influence the tolerance of *A. solani* to Ca^2+^.

Similar to the silenced mutant of CmrA in *A. alternata* (Fetzner et al. 2014), the Δ*asmr1-2* mutant of *A. solani* reduced tolerance to H_2_O_2_. In contrast, a deletion mutant of Amr1 in *A. brassicicola* remained unchanged in the sensitivity against H_2_O_2_ (Cho et al. 2012). This implies the diversification of responding to oxidative stress in Cmr1 homologs in *Alternaria*. Plants generate toxic ROS, primarily H_2_O_2_ and superoxide as a defense response against pathogens. For a plant fungal pathogen, its sensitivity to ROS likely depends on the effectiveness of its own ROS detoxification ability. In *A. alternata*, the detoxification of ROS is regulated by the redox-responsive transcription regulators YAP1 and SKN7 or by a nonribosomal peptide synthetase (NPS) mediated pathway (Lin et al. 2009; Yang et al. 2009; Chen et al. 2012, 2013). The ability of *A. alternata* to detoxify ROS is also required for virulence on citrus (Lin et al. 2009; Yang et al. 2009). Hence, the lack of Asmr1 might likely affect the ability of *A. solani* to effectively detoxify H_2_O_2_ through impairing ROS detoxification pathway, further influencing the tolerance of *A. solani* to external H_2_O_2_ stress.

One of the interesting findings of this study is the observation on the involvement of a melanin-regulating gene in fungicide sensitivity. Asmr1 was found to be required for cellular resistance to mancozeb, chlorothalonil, and azoxystrobin. Mancozeb is shown to be an inhibitor of metal- and/or sulfhydryl-containing enzymes related to lipid metabolism, respiration, and ATP production, resulting in the inhibition of fungal spore germination (Dias et al. 2010; Gullino et al. 2010). Chlorothalonil has a multi-site mode of action that inhibits specific NAD thiol-dependent glycolytic and respiratory enzymes (Tillman et al. 1973). Azoxystrobin is a quinone outside inhibitor (QoI) fungicide with a site-specific mode of action. It inhibits mitochondrial respiration by binding to Qo site of the cytochrome b, blocking electron transfer, and thus halting ATP synthesis (Bartlett et al. 2002). Based on the mode of action of these tested fungicides, we predict that the reduced level of melanin due to the deletion of *asmr1* might lead to the decreased detoxification ability of *A. solani* on substances affecting fungal spore germination (mancozeb/chlorothalonil), glycolysis (chlorothalonil), and respiration (azoxystrobin/chlorothalonil). Genome-wide expression analysis between the Δ*asmr1-2* mutant and the WT under appropriate conditions are worthy to do to further characterize the function of Asmr1, and to understand its role in abiotic stress response.

Asmr1 was found to be related to the pathogenicity in *A. solani*. Given the involvement of the Δ*asmr1-2* mutant in the sensitivity to cell wall stressors, it seems likely that the ability of the Δ*asmr1-2* mutant to respond to cell wall stress was associated with its decreased pathogenicity. Asmr1 might regulate the expression of a set of genes encoding CWDEs that are important for decomposing and utilizing plant material during infection. In this context, it is worth noting that many glycoside hydrolases 61 family genes are highly expressed in the deletion mutant of *Amr1* in *A. brassicicola* during pathogenesis (Cho et al 2012). Thus, *A. brassicicola* can quickly and efficiently utilize pectin and other cell wall components of green cabbage plant cells, causing the host cells to be more easily damaged. Another explanation for the decreased pathogenicity may be a connection between melanin and spore germination or the ROS detoxification ability. Although the deletion of *asmr1* did not affect the yield of conidia, the deformed conidia in the Δ*asmr1-2* mutant might influence spore germination. The possibility of a positive relationship between Asmr1 and CWDEs/spore germination/ROS detoxification could be explored further through experimental investigation of expression profiles between the Δ*asmr1-2* mutant and WT during the infection on potato using the RNA-seq approach followed by functional assays.

While in the same genus, Amr1 can negatively regulate the pathogenicity in *A. brassicicola* (Cho et al. 2012). Asmr1 positively regulated the pathogenicity in *A. solani*. It suggests there is the pathogenicity diversification regulated by Cmr1 homologs in *Alternaria*. Significantly, their predicted secondary structures were almost identical, despite the sequence variation between Asmr1, Amr1, and CmrA. This is possibly due to the fact that the amino acid substitutions among them include a high proportion of the same physico-chemical groups, such as nonpolar and non-charged amino acids. Considering the conservation of the protein structure and diversification in pathogenicity, we speculate that the structural architecture is under high selective constraint in the function of Asmr1 and Amr1, which is driven by the co-evolution between pathogens *A. solani* and *A. brassicicola* and their corresponding hosts. With the increase in the genomic sequences available of *Alternaria* spp., this will contribute to improve our understanding the evolutionary relationships among Cmr1 homologs in *Alternaria*. This study provides an experimental framework to identify Cmr1 homologs in *Alternaria* spp. and explore their evolution and function. In combination with the identification of targets of Cmr1 homologs, this will ultimately help to make a link between the evolution of Cmr1 homologs and their functional roles in the biology of *Alternaria* spp.

## Supplementary Information

Below is the link to the electronic supplementary material.

Supplementary file1 (PDF 2659 KB)

## Data Availability

The sequences of Cmr1 homologs from *A. solani* isolates have been deposited in GenBank under accession numbers ON962808 (isolate SH0806) and OP302825 to OP302833 (isolates DX0917, DQ0803, JG0805, KS0809, BX0917, BJPO2207, BJTO2207, JSTO2207, and SDTO2207, respectively).
