# Supplementary material for: Positive selection and functional diversification of transcription factor Cmr1 homologs in Alternaria
Source: Appl Microbiol Biotechnol. 2024 Jan 15;108:133. doi: 10.1007/s00253-023-12893-7 (PMC10789848; doi:10.1007/s00253-023-12893-7)
Supplement: Supplementary file 1 [file 253_2023_12893_MOESM1_ESM.pdf]

Applied Microbiology and Biotechnology Supplemental Data

Article title: **Positive selection and functional diversification of transcription factor Cmr1 homologs in *Alternaria***

Chaodong Qiu[1], Zhenyu Liu[1,2]*

[1]Department of Plant Pathology, School of Plant Protection, Anhui Agricultural University, Hefei, China

[2]Anhui Province Key Laboratory of Integrated Pest Management on Crops, Hefei, 230036, China

*** Correspondence:**

Zhenyu Liu, zliu@ahau.edu.cn; Tel: 0551-65786312; Fax number: 0551-65785932

> *Alternaria alstroemeriae* (NA）

ATGGTCTTCTGCACATATTGTGGACAGTCGTTCACCAGGGACGAACATCTCGAACGACATATCCTAACTCGTAAGTCAACTCTTGCTCTGTCCATGAGGGTGGATTTCC

GCAATGTCGAGTTCAATCCGATCGTTCCGTAGCATCTCCGCTGACCGTCAATGCCAAACAGACACCAATGTCAAGCCATTCAAGTGCTTTACATGTCATATGAGCTTTG

CTCGACGGTAAGTCCCACCCTGCGTGCGCTCCTTCCTCTTCCTGTTTGGTCATAATTTCCGTACCGAACGAAACGCTGACAATCTTTCCAGAGATCTTTTGCAAAGACA

TTACACCGTACACGGCCGCAACCAGAACAACGAAGAAGGCCTCCCACCGCAAGGCATCATACCTAAGTCCGCCGGCCGCACACCCATCGCCTGCAGTAATTGTGCAA

AGACGAAGACGAAATGCGACAAGAAGTTTCCTTGCACCCGGTGCGCTCAAAGGAACCTGAAGTGTACTCTACGTCCTACACGGCGAGCATCGAAAGGTGTCCAACGA

GTAGGAGGTCCTCCTGAAACAGGCAGTGGTGATTCTAGCGAGAGTGGTAGCAACGCGGATAACCAGAACACATCCGGCAATGTCTCCCCAAACCACGATGGCCAGTC

GAAACAAGCAACCGCTCAGTCGTCTCCATCTCAAATTCAGCAGCCTAGCCAGATTTCTGACAGCCAACCTCCATCGCAACGGCAATCGCAATCTCCCTCAGCGCCCCA

CTCGCGGAATCCTTCCATCTCACATCCAGTGCAACCACCACCGCACATGAACCCCGAGGAGAAGTCGCTCCACATACACATGCCTATCTCGAATACTCCCCCGTTCTT

CGATCAGTCACCCTCTAACGGACTGTCTCATTCCTCATCATTACTTTCACCTCTTCCCACACCAGTTACGGGCATGAATAGTTTTGTTTCTTCGACACCCATGTCTGGGT

ACGACGACTTCGTCAGGACAGTGCGCGATCAACCGGAAAATGAGAGCCCGCAATTCATGATGGATCCTTGGCACTCGATGCCCATGGATACGAGTTACGATCCTATGC

GGATTGACCCCGCTCTGATGATGACCATGGGCATGGACATGACGATGGGACCGCCCCAAGACGGTATCCTTAGTATGATACCCGAAATGTCACCTGCGCAAACATTCG

GACAGCCTGTTCAGACTCCTCGTATGGTGGACGAATCGTTCTCGGACTTGCAAATCGGCAGTTCGGCTTCGATGTTCTACCCCTCTAACAGACATTCTTCAATAGCGGA

CGCCGGCATCCCTGATCTTGGCGCCATCATCGCAGCCCAAGACGGCTGGACGGTCTTCCGTTGCACTCCTTCTATCGCTTCGAGCTCATGTCCGCGGACTGCAAGGC

TGAATCTTGAGAGATTAGAGTTGAGCCTGAAGAACCATGAGGGCTGGGCTAACTGGACCCCATCGTGGGACGAATCTGATTTCCAACAAGGCGGACAGATCGCCGTG

TCAAAACTCCAGGAAGCCACACGAGACAAACTCCTCGCGATCACACAGAGCTTCCTTCACAAGGCTCTAGATATCCACAAAGATGGAAGCAGCTCGTCTAGTAGTGGC

GAGTCTCCAGGATCTACGGCTTCACATTCCAACTTCGTTCTTCTTCCGCCTGCTCGTGTCCTGGAGTACTTCCTCAAGTCGTACGCCAACTCTTTCGAACGCTATTATCC

TACCTCGGCCAGAGGAGTCCTCGACACCAACGAGTTGATGCAGAACTACAATGACAAAGCTTCGAGCTTGCTCATTCTCATGATGATTGCGCAGGGCGCTATGACCAT

TCCGTCCATAGAAGCGAGATGGCTGACTGGCGGCTTTACTGAGGCATGTCGGATATCCCTGTTCGACCTCATCGAGAAGAACATCATTATGGCTGGCGATCCTATTGT

CCTGAGGGCGGCCCTTCTCTTCACTGTTCAAGCGGCGTGGAGTGGCGACAAGTGGCAGATGGACATCGCCATGGGTCAGCGCGGCATGTATTTTTCCATGTTACGAC

ATTCCGGTATTCTCGATGTTCGACCGCCTACCACGCCTCACATGAATGGAAACACTTCGACGGATGTGTTGTGGAAGGACTGGCTTCAGCAAGAAAGCCGCAGCCGGT

AAGTGTTGAAAACCTCGGTACAGTCACGTGCACGCACTTACTAATATCGTGTAGCCTCATCTACTCTTGGGTCATGGTAGACCAAGACCTATCGCTATTCCACGATACC

GCTCCCCTGTTTTCGGTAACAGAATTTGGCGCGCCAATGCCTGATTCAGATCAACTCTGGCAGGCGCAGACAGCTCCAGAGTGGTCGGAGATGTTCAACCAGGTCCAT

GAATTCTCGAATGGATACTCCTCAGTAGGCTCTGGAGCAAGACCACCGAGTCTCAGAGATCTCTTCCGCTACTTCCTAGATGACGAGATAGTCGTCCAGGATGTTCAG

GAGATTCATCTGACCCCGATGCACCTGCGACTACTACTGCACCCTCTGCAGACCCTTGTGTGCCAATATTGCCAACTCCTCAGTTGCTTTTCCGACAGTGTAGCCTCTC

GATCGCGGAATCGGGCATTGACAGCTGCTTCGACTCGCGTACGCCTAGAAGAGGTGCAGGCGTTATTGGGGCGGTGGTTCGACTTGGCGCAACGGTACATGAAGGC

GAACCCAGTATGTCCGATGATGCAAGCAAATCTCGTCATGTTCCATCTTATCAGCATGAATGCTGTGTCAAATTTCCCAGAGATTGAGCGCCTCGCCCGACGGGAGGG

TGTGGACGGCTCGTATCAACAGATGCTGTGGATGCACAAGAAGTGCTTGTCGGATGTGCAGGAGGCCATCACTCACGCTGGCCAAGTTCTTCGCCTCATTCGTGAGAT

GCCACGAGGCATTCGCCCGCCGTGGTGGGCTGGTGCTGTCTATCGGGCAGGGTTGGTCTTCTGGGCTGACTCGCTCGCTCACAACGAGTCCTTGAGTCCCAACCAT

CAAGGGACATATCAGCCATCTAATACTGCTTTCTCTGTGGATCGCCTCTCTGCTGACCACCCAACCATACTGAGGTACATGTCACGGAGGGAGGGTATTCCCACCTTG

ACCAAGCGCGATGGTAGCCCTGTTCCACTGGATAACGGCTTTGCCGTCCTGTCGCATTGTATCGATGTCCTTGACGAGGGTGTCGCCACCAGGTTTTCCGATGGCATT

CGTAGTAAACTCGAGAAACTTGCGCGCGGTTAA

>*Alternaria alternata* (KJ939356[a])

ATGGTCTTCTGCACATATTGTGGACAGTCGTTCACCAGGGACGAACATCTCGAACGACATATCCTAACTCGTAAGTCAACTCTTGCTCTGTCCATGAGGGTGGATTTCC

GCAATGTCGAGTTCAATCCGATCGTTCCGTAGCATCTCCGCTGACCGTCAATGCCAAACAGACACCAATGTCAAGCCATTCAAGTGCTTTACATGTCATATGAGCTTTG

CTCGACGGTAAGTCCCACCCTGCGTGCGCTCCTTCCTCTTCCTGTTTGGTCATAATTTCCGTACCGAACGAAACGCTGACAATCTTTCCAGAGATCTTTTGCAAAGACA

TTACACCGTACACGGCCGCAACCAGAACAACGAAGAAGGCCTCCCACCGCAAGGCATCATACCTAAGTCCGCCGGCCGCACACCCATCGCCTGCAGTAATTGTGCAA

AGACGAAGACGAAATGCGACAAGAAGTTTCCTTGCACCCGGTGCGCTCAAAGGAACCTGAAGTGTACTCTACGTCCTACACGGCGAGCATCGAAAGGTGTCCAACGA

GTAGGAGGTCCTCCTGAAACAGGCAGTGGTGATTCTAGCGAGAGTGGTAGCAACGCGGATAACCAGAACACATCCGGCAATGTCTCTCCAAACCACGATGGCCAGTC

GAAACAAGCAACCGCTCAGTCGTCTCCATCTCAAATTCAGCAGCCTAGCCAGATTTCTGACAGCCAACCTCCATCGCAACGGCAATCGCAATCTCCCTCAGCGCCCCA

CTCGCGGAATCCTTCCATCTCACATCCAGTGCAACCACCACCGCACATGAACCCCGAGGAGAAGTCGCTCCACATACACATGCCTATCTCGAATACTCCCCCGTTCTT

CGATCAGTCACCCTCTAACGGACTGTCTCATTCCTCATCATTACTTTCACCTCTTCCCACACCAGTTACGGGCATGAATAGTTTTGTTTCTTCGACACCCATGTCTGGGT

ACGACGACTTCGTCAGGACAGTGCGCGATCAACCGGAAAATGAGAGCCCGCAATTCATGATGGATCCTTGGCACTCGATGCCCATGGATACGAGTTACGATCCTATGC

GGATTGACCCCGCTCTGATGATGACCATGGGCATGGACATGACGATGGGACCGCCCCAAGACGGTATCCTTAGTATGATACCCGAAATGTCACCTGCGCAAACATTCG

GACAGCCTGTTCAGACTCCTCGTATGGTGGACGAATCGTTCTCGGACTTGCAAATCGGCAGTTCGGCTTCGATGTTCTACCCCTCTAACAGACATTCCTCAATAGCGGA

CGCCGGCATCCCTGATCTTGGCGCCATTATCGCAGCCCAAGACGGCTGGACGGTCTTCCGTTGCACTCCTTCTATCGCTTCGAGCTCATGTCCGCGGACCGCAAGGC

TGAATCTTGAGAGATTAGAGTTGAGCCTGAAGAACCATGAGGGCTGGGCTAACTGGACCCCATCGTGGGACGAATCTGATTTCCAACAAGGCGGACAGATCGCCGTG

TCAAAACTCCAGGAAGCCACACGAGACAAACTTCTCGCGATCACACAGAGCTTCCTTCACAAGGCTCTAGATATCCACAAAGATGGGAGCAGCTCGTCTAGTAGTGGC

GAGTCTCCAGGATCTACGGCTTCACATTCCAACTTCGTTCTTCTTCCGCCTGCTCGTGTCCTGGAGTACTTCCTCAAGTCGTACGCCAACTCTTTCGAACGCTATTATCC

TACCTCGGCCAGAGGAGTCCTCGACACCAACGAGTTGATGCAGAACTACAATGACAAAGCTTCGAGCTTGCTCATTCTCATGATGATTGCGCAGGGCGCTATGACCAT

TCCGTCCATAGAAGCGAGATGGCTGACTGGCGGCTTTACTGAGGCATGTCGGATATCCCTGTTCGACCTCATCGAGAAGAACATCATTATGGCTGGCGATCCTATTGT

CCTGAGGGCGGCCCTTCTCTTCACTGTTCAAGCGGCGTGGAGTGGCGACAAGTGGCAGATGGACATCGCCATGGGCCAGCGCGGCATGTATTTTTCCATGTTACGAC

ATTCCGGTATTCTCGATGTTCGACCGCCTACCACGCCTCACATGAATGGAAACACTTCGACGGATGTGTTGTGGAAGGACTGGCTTCAGCAAGAAAGCCGCAGCCGGT

AAGTGTTGAAAACCTCGGTACAGTCACGTGCGCGCACTTACTAATATCGTGTAGCCTCATCTACTCTTGGGTCATGGTAGACCAAGACCTATCGCTATTCCACGATACC

GCTCCCCTGTTTTCGGTAACAGAATTTGGCGCGCCAATGCCTGATTCAGATCAACTCTGGCAGGCGCAGACAGCTCCAGAGTGGTCGGAGATGTTCAACCAGGTCCAT

GAATTCTCGAATGGATACTCCTCAGTAGGCTCTGGAGCAAGACCACCGAGTCTCAGAGATCTCTTCCGCTACTTCCTAGATGACGAGATAGTCGTCCAGGATGTTCAG

GAGATTCATCTGACCCCGATGCACCTGCGACTACTACTGCACCCTCTGCAGACCCTTGTGTGCCAATATTGCCAACTCCTCAGTTGCTTCTCCGACAGTGTAGCCTCTC

GATCGCGGAATCGGGCATTGACAGCTGCTTCGACTCGCGTACGCCTAGAAGAGGTACAGGCGTTATTGGGGCGGTGGTTCGACTTGGCGCAACGGTACATGAAGGC

GAACCCAGTATGTCCGATGATGCAAGCAAATCTCGTCATGTTCCATCTTATCAGCATGAATGCTGTGTCAAATTTCCCAGAGATTGAGCGCCTCGCCCGACGGGAGGG

TGTGGACGGCTCGTATCAACAGATGCTGTGGATGCACAAGAAGTGCTTGTCGGATGTGCAGGAGGCCATCACTCACGCTGGCCAAGTTCTTCGCCTCATTCGTGAGAT

GCCACGAGGCATTCGCCCGCCGTGGTGGGCTGGTGCTGTCTATCGGGCAGGGTTGGTCTTCTGGGCTGACTCGCTCGCTCACAACGAGTCCTTGAGTCCCAACCAT

CAAGGGACATATCAGCCATCTAATACTGCTTTCTCTGTGGATCGCCTCTCTGCTGACCACCCAACCATACTGAGGTACATGTCACGGAGGGAGGGTATTCCCACCTTG

ACCAAGCGCGATGGTAACCCTGTTCCACTGGATAACGGCTTTGCCGTCCTGTCGCATTGTATCGATGTCCTTGACGAGGGTGTCGCCACCAGGTTTTCCGATGGCATT

CGTAGTAAACTCGAGAAACTTGCGCGCGGTTAA

>*Alternaria angustiovoidea* (NA)

ATGGTCTTCTGCACATATTGTGGACAGTCGTTCACCAGGGACGAACATCTCGAACGACATATCCTAACTCGTAAGTCAACTCTTGCTCTGTCCATGAGGGTGGATTTCC

GCAATGTCGAGTTCAATCCGATCGTTCCGTAGCATCTCCGCTGACCGTCAATGCCAAACAGACACCAATGTCAAGCCATTCAAGTGCTTTACATGTCATATGAGCTTTG

CTCGACGGTAAGTCCCACCCTGCGTGCGCTCCTTCCTCTTCCTGTTTGATCATAATTTCCGTACCGAACGAAACGCTGACAATCTTTCCAGAGATCTTTTGCAAAGACAT

TACACCGTACACGGCCGCAACCAGAACAACGAAGAAGGCCTCCCACCGCAAGGCATCATACCTAAGTCCGCCGGCCGCACACCCATCGCCTGCAGTAATTGTGCAAA

GACGAAGACGAAATGCGACAAGAAGTTTCCTTGCACCCGGTGCGCTCAAAGGAACCTGAAGTGTACTCTACGTCCTACACGGCGAGCATCGAAAGGTGTCCAACGAG

TAGGAGGTCCTCCTGAAACAGGCAGTGGTGATTCTAGCGAGAGTGGTAGCAACGCGGATAACCAGAACACATCCGGCAATGTCTCTCCAAACCACGATGGCCAGTCG

AAACAAGCAACCGCTCAGTCGTCTCCATCTCAAATTCAGCAGCCTAGCCAGATTTCTGACAGCCAACCTCCATCGCAACGGCAATCGCAATCTCCCTCAGCGCCCCAC

TCGCGGAATCCTTCCATCTCACATCCAGTGCAACCACCACCGCACATGAACCCCGAGGAGAAGTCGCTCCACATACACATGCCTATCTCGAATACTCCCCCGTTCTTC

GATCAGTCACCCTCTAACGGACTGTCTCATTCCTCATCATTACTTTCACCTCTTCCCACACCAGTTACGGGCATGAATAGTTTTGTTTCTTCGACACCCATGTCTGGGTA

CGACGACTTCGTCAGGACAGTGCGCGATCAACCGGAAAATGAGAGCCCGCAATTCATGATGGATCCTTGGCACTCGATGCCCATGGATACGAGTTACGATCCTATGC

GGATTGACCCCGCTCTGATGATGACCATGGGCATGGACATGACGATGGGACCGCCCCAAGACGGTATCCTTAGTATGATACCCGAAATGTCACCTGCGCAAACATTCG

GACAGCCTGTTCAGACTCCTCGTATGGTGGACGAATCGTTCTCGGACTTGCAAATCGGCAGTTCGGCTTCGATGTTCTACCCCTCTAACAGACATTCCTCAATAGCGGA

CGCCGGCATCCCTGATCTTGGCGCCATTATCGCAGCCCAAGACGGCTGGACGGTCTTCCGTTGCACTCCATCTATCGCTTCGAGCTCATGTCCGCGGACCGCAAGGC

TGAATCTTGAGAGATTAGAGTTGAGCCTGAAGAACCATGAGGGCTGGGCTAACTGGACCCCATCGTGGGACGAATCTGATTTCCAACAAGGCGGACAGATCGCCGTG

TCAAAACTCCAGGAAGCCACACGAGACAAACTCCTCGCGATCACACAGAGCTTCCTTCACAAGGCTCTAGATATCCACAAAGATGGAAGCAGCTCGTCTAGTAGTGGC

GAGTCTCCAGGATCTACGGCTTCACATTCCAACTTCGTTCTTCTTCCGCCTGCTCGTGTCCTGGAGTACTTCCTCAAGTCGTACGCCAACTCTTTCGAACGCTATTATCC

TACCTCGGCCAGAGGAGTCCTCGACACCAACGAGTTGATGCAGAACTACAATGACAAAGCTTCGAGCTTGCTCATTCTCATGATGATTGCGCAGGGCGCTATGACCAT

TCCGTCCATAGAAGCGAGATGGCTGACTGGCGGCTTTACTGAGGCATGTCGGATATCCCTGTTCGACCTCATCGAGAAGAACATCATTATGGCTGGCGATCCTATTGT

CCTGAGGGCGGCCCTTCTCTTCACTGTTCAAGCGGCGTGGAGTGGCGACAAGTGGCAGATGGACATCGCCATGGGTCAGCGCGGCATGTATTTTTCCATGTTACGAC

ATTCCGGTATTCTCGATGTTCGACCGCCTACCACGCCTCACATGAATGGAAACACTTCGACGGATGTGTTGTGGAAGGACTGGCTTCAGCAAGAAAGCCGCAGCCGGT

AAGTGTTGAAAACCTCGGTACAGTCACGTGCGCGCACTTACTAATATCGTGTAGCCTCATCTACTCTTGGGTCATGGTAGACCAAGACCTATCGCTATTCCACGATACC

GCTCCCCTGTTTTCGGTAACAGAATTTGGCGCGCCAATGCCTGATTCAGATCAACTCTGGCAGGCGCAGACAGCTCCAGAGTGGTCGGAGATGTTCAACCAGGTCCAT

GAATTCTCGAATGGATACTCCTCAGTAGGCTCTGGAGCAAGACCACCGAGTCTCAGAGATCTCTTCCGCTACTTCCTAGATGACGAGATAGTCGTCCAGGATGTTCAG

GAGATTCATCTGACCCCGATGCACCTGCGACTACTACTGCACCCTCTGCAGACCCTTGTGTGCCAATATTGCCAACTCCTCAGTTGCTTTTCCGACAGTGTAGCTTCTC

GATCGCGGAATCGGGCATTGACAGCTGCTTCGACTCGCGTACGCCTAGAAGAGGTGCAGGCGTTATTGGGGCGGTGGTTCGACTTGGCGCAACGGTACATGAAGGC

GAACCCAGTATGTCCGATGATGCAAGCAAATCTCGTCATGTTCCATCTTATCAGCATGAATGCTGTGTCAAATTTCCCAGAGATTGAGCGCCTCGCCCGACGGGAGGG

TGTGGACGGCTCGTATCAACAGATGCTGTGGATGCACAAGAAGTGCTTGTCGGACGTGCAGGAGGCCATCACTCACGCTGGCCAAGTTCTTCGCCTCATTCGTGAGA

TGCCACGAGGCATTCGCCCGCCGTGGTGGGCCGGTGCTGTCTATCGGGCAGGGTTGGTCTTCTGGGCTGACTCGCTCGCTCACAACGAGTCCTTGAGTCCCAACCA

TCAAGGGACATATCAGCCATCTAATACTGCTTTCTCTGTGGATCGCCTCTCTGCTGACCACCCAACCATACTGAGGTACATGTCACGGAGGGAGGGTATTCCCACCTTG

ACCAAGCGCGATGGTAGCCCTGTTCCACTGGATAACGGCTTTGCCGTCCTGTCGCATTGTATCGATGTCCTTGACGAGGGTGTCGCCACCAGGTTTTCCGATGGCATT

CGTAGTAAACTCGAGAAACTTGCGCGCGGTTAA

>*Alternaria arborescens* (XM_028649146[a])

ATGGTCTTCTGCACATATTGCGGACAGTCGTTCACCAGGGACGAACATCTCGAACGACATATCCTAACTCGTAAGTCAACTCTTGCTCTGTCCATGAGGGTGGATTTCC

GCAACGTCGAGTTCAATCCGATCGTTCCGTAGCATCTCCGCTGACCGTCAATGCCAAACAGACACCAATGTCAAGCCATTCAAGTGCTTTACATGTCATATGAGCTTTG

CTCGACGGTAAGTCCCACCCTGCGTGCGCTCCTTCCTCTTCTTGTTTGGTCATAATTTCCGTACCGAACGAAACGCTGACAATCTTTCCAGAGATCTTTTGCAAAGACAT

TACACCGTACACGGCCGCAACCAGAACAACGAAGAAGGCCTCCCACCGCAAGGCATCATACCTAAGTCCGCCGGCCGCACACCCATCGCCTGCAGTAATTGTGCAAA

GACGAAGACGAAATGCGACAAGAAGTTTCCTTGCACCCGGTGCGCTCAAAGGAACCTGAAGTGTACTCTACGTCCTACACGGCGAGCATCGAAAGGTGTCCAGCGAG

TAGGAGGTCCTCCTGAAACAGGCAGTGGTGATTCTAGCGAGAGTGGTAGCAACGCGGATAACCAGAACACATCCGGCAATGTCTCTCCAAACCACGATGGCCAGTCG

AAACAAGCAACCGCTCAGTCGTCTCCATCTCAAATTCAGCAGCCTAGCCAGATTTCTGACAGCCAACCTCCATCGCAACGGCAATCGCAATCTCCCTCAGCGCCCCAC

TCGCGAAATCCTTCCATCTCACATCCAGTGCAACCACCACCGCACATGAACCCCGAGGAGAAGTCGCTCCACATACACATGCCTATCTCGAATACTCCCCCGTTCTTC

GATCAGTCACCCTCTAACGGACTGTCTCATTCCTCATCATTACTTTCACCTCTTCCCACACCAGTGACGGGCATGAATAGTTTTGTTTCTTCGACACCCATGTCTGGATA

CGACGACTTCGTCAGGACAGTGCGCGATCAACCGGAAAATGAGAGCCCACAATTCATGATGGATCCTTGGCACTCGATGCCCATGGATACGAGTTACGATCCTATGCG

GATTGACCCCGCTCTGATGATGACCATGGGCATGGACATGACGATGGGACCGCCCCAAGACGGTATCCTTAGTATGATACCCGAAATGTCACCTGCGCAAACATTCGG

ACAGCCTGTTCAGACTCCTCGTATGGTGGACGAATCGTTCTCGGACTTGCAAATCGGCAGTTCGGCTTCGATGTTCTACCCCTCTAACAGACATTCCTCAATAGCGGAC

GCCGGCATCCCTGATCTTGGCGCCATTATCGCAGCCCAAGACGGCTGGACGGTCTTCCGTTGCACTCCTTCTATCGCTTCGAGCTCATGTCCGCGGACTGCAAGGCT

GAATCTTGAGAGATTAGAGTTGAGCCTGAAGAACCATGAGGGCTGGGCTAACTGGACCCCATCGTGGGACGAGTCTGATTTCCAACAAGGCGGACAGATCGCCGTGT

CAAAACTCCAAGAAGCCACACGAGACAAACTCCTCGCGATCACACAGAGCTTCCTTCACAAGGCTCTAGATATCCACAAAGATGGAAGCAGCTCGTCTAGTAGTGGTG

AGTCTCCAGGATCTACGGCTTCACATTCCAACTTCGTTCTTCTTCCGCCTGCTCGTGTCCTGGAGTACTTCCTCAAGTCGTACGCCAACTCTTTCGAACGCTATTATCCT

ACCTCGGCCAGAGGAGTCCTCGACACCAACGAGTTGATGCAGAACTACAATGACAAAGCTTCGAGCTTGCTCATTCTCATGATGATTGCGCAGGGCGCTATGACCATT

CCGTCCATAGAAGCGAGATGGCTGACTGGTGGCTTTACTGAGGCATGTCGGATATCCCTGTTCGACCTCATCGAGAAGAACATCATTATGGCTGGCGATCCTATTGTC

CTGAGGGCGGCCCTTCTCTTCACTGTTCAAGCGGCGTGGAGTGGCGACAAGTGGCAGATGGACATCGCCATGGGTCAGCGCGGCATGTATTTTTCCATGTTACGACA

CTCCGGTATTCTCGATGTTCGACCGCCTACCACGCCTCACATGAATGGAAACACTTCGACGGATGTGTTGTGGAAGGACTGGCTTCAGCAAGAAAGCCGCAGCCGGT

AAGTGTTGAAAACCTCAGTACAGGCACGTGCGCGCACTTACTAATATCGTGTAGCCTCATCTACTCTTGGGTCATGGTAGACCAAGACCTATCGCTATTCCACGATACT

GCTCCCCTATTTTCGGTAACAGAATTTGGCGCGCCGATGCCTGATTCAGATCAACTCTGGCAGGCGCAGACAGCTCCAGAGTGGTCGGAGATGTTCAACCAGGTCCAT

GAATTCTCGAATGGATACTCCTCAGTAGGCTCTGGAGCAAGACCACCGAGTCTCAGAGATCTCTTCCGCTACTTCCTAGATGACGAGATAGTTGTCCAGGATGTTCAG

GAGATTCATCTGACCCCGATGCACCTGCGACTACTACTGCACCCTCTGCAGACCCTTGTGTGCCAATATTGCCAACTCCTCAGTTGCTTTTCCGACAGTGTAGCCTCTC

GATCGCGGAATCGGGCATTGACAGCTGCTTCGACTCGCGTACGCCTAGAAGAGGTGCAGGCGTTGTTGGGGCGGTGGTTCGACTTGGCGCAACGGTACATGAAGGC

GAACCCAGTATGTCCGATGATGCAGGCAAATCTCGTCATGTTCCATCTTATCAGCATGAATGCTGTGTCAAATTTCCCAGAGATTGAGCGCCTCGCCCGACGGGAGGG

TGTGGACGGCTCGTATCAACAGATGCTGTGGATGCACAAGAAGTGCTTGTCGGATGTGCAGGAGGCCATCACTCACGCTGGCCAAGTTCTTCGCCTCATTCGTGAGAT

GCCACGAGGCATTCGCCCGCCGTGGTGGGCTGGTGCTGTCTATCGGGCAGGGTTGGTCTTCTGGGCTGACTCACTCGCTCACAACGAGTCCTTGAGTCCCAACCATC

AAGGGACATATCAGCCATCAAATACTGCTTTCTCTGTGGATCGCCTCTCTGCTGACCACCCAACCATACTGCGGTACATGTCACGGAGGGAGGGTATTCCCACCTTGA

CCAAGCGCGATGGTAGCCCTGTTCCACTGGATAACGGCTTTGCCGTCCTGACGCATTGTATCGATGTCCTCGACGAGGGTGTCGCCACCAGGTTTTCCGATGGCATTC

GTAGTAAACTCGAGAAACTTGCACGCGGTTAA

>*Alternaria arbusti* (NA)

ATGGTTTTTTGCACATATTGTGGACAGTCGTTCACCAGAGACGAGCATCTCGAACGACATATCTTGACTCGTATGTTGACCCCTCGTCTTTCGCTCTTTCATTCTTCCTC

GCACCAGCTACTATCCAATCTTTCTTCTGGGATATCTGCTAATCACTATGGACGAGCAGATACCAACGTCAAGCCTTTCAAATGCTTCACATGTCATATGAGCTTTGCTC

GACGGTATGTGGCACCCTTCCTTGCTCTTCCTCATCCTGTGGCACCGACCGTTCGGAACAAAGTACTGACAGACTCTTCAGAGATCTTTTGCAAAGACACTACACCGTA

CACGGCCGCAACCAGAACAACGAAGAGGGCCTCCCTCCGCAAGGCATAATACCAAAGTCCGCCGGCCGCACGCCCATCGCCTGCAGTAATTGTGCGAAGACAAAGA

CCAAGTGTGACAAGAAGTTTCCGTGCACTAGGTGCGCTCAGAGGAATCTGAAATGCACATTGCGTCCTACAAGGCGGGCATCGAAAGGTGTCCAGCGGGTTGGCGGT

CCTCCTGAGGCTGGGAGCGGGGATTCGAGCGAGAGTGGCAGCAACGCGGATAACCACAACACATCCGGCAATGTCTCTCCAAACCACGATAGCCAACCAAAACAAGC

GGTGTCGAAGTCCTCTCCCGCTCAGATCCACCAGACTTCCCATATTCCGGACAGCCAGCCACCGTCGCAAAGACAATCGCAGTCGCCCTCGGCCCCTCATTCACGAA

ACCCTTCCATCTCGCAATCGGTACAGCCACCAACACACATAAATCCCGAGGAGAAGTCGTTGCATCTTCATATGCCGATCTCTGATACGCCGCCCTTTTTCGACCAGTC

TCCTTCCAACGGACTTGCTCAATCATCGTCGCTTCTTTCGCCACTCCCCACCCCGGTGACCGGCATGAATGGCTTCGTCACCTCCACACCTATGTCAGGATATGACGA

CTTCGTTAGAACGGTACGGGACCAATCCGATAATGAGAGCCCTCAATTCATGATGGACCCTTGGAACGGCATGCCCATGGACACAACCTTTGATCCCATGCGGATAGA

CCCCGCGTTGATGATGAGCATGAGTATGGACATGAGTATGGGACCGCCTCAGGATGGCATCCTTGGCATGATACCAGAAATGTCGCCTGCTCAAACATTTGGACAGCC

TGTTCAGACACCTCGCATGGACGAGTCCTTTTCGGATCTGCAAATTGGTAGTTCGGCTTCGATGTTCTACCCCTCCAACAGGCACTCTTCGATAGCAGATGCCGGCATA

CCTGATCTTGGTGCCATTATCGCGGCTCAAGATGGCTGGACTGTTTTCCGTTGCACTCCTTCTATTGCTTCAAGCTCGTGTCCACGCACAGCGAGACTCAACCTCGAG

CGGTTAGAGCTGAGCCTCAAGAACCACGAGGGCTGGGCTAACTGGTCCCCATCGTGGGATGAATCCGACTTTCAACAAGGCGGACAAATCGCGGTATCGAAGTTGCA

GGAAGCCACGCGAGACAAACTTCTCGCCATCACCCAAAGCTTCCTCCACAAGGCTTTGGACATCCACAAAGACGGCAGTAGCTCGTCCAGCAGTGGCGAGTCTCCAG

GATCCACGGCCTCTCACTCCAACTTCGTTCTTCTTCCACCTGCTCGCGTCCTGGAGTACTTTCTCAAATCATACGCCAACTCTTTCGAACGCTATTATCCCACCTCCGCC

AGAGGTGTACTCGATACTAATGAACTGATGCAAAACTACAACGATAAAGCATCGAGTTTGCTCATTCTCATGATGATTGCGCAGGGCGCCATGACCATTCCATCCATAG

AGGCCAGGTGGCTAACTGGTGGCTTCACCGAAGCCTGCCGGATATCGCTCTTCGACCTTATCGAGAAGAACATCATCATGGCTGGTGATCCTATCGTTCTGCGTGCAG

CCCTCCTCTTCACTGTCCAGGCGGCGTGGAGTGGCGACAAATGGCAGATGGACATCGCTATGGGCCAGCGTGGCATGTATTTTTCCATGTTGCGACATTCCGGTATTC

TGGATGTGCGACCGCCAGCAACGCCTCATATAAACGGAAACATTTCGACGGATGCGTTGTGGAAGGACTGGCTTCAGCAAGAAAGCCGCAGTCGGTAAGTGTTGAAA

ACTTCGGTACCGTCACGTGAACGCACTTACTAATATTGGCGCAGCCTCATCTACTCCTGGGTCATGGTAGACCAAGACTTATCGCTGTTCCACGATACCGCTCCACTGT

TTTCAGTAACAGAGTTTGGCGCGCCGATGCCTGATTCGGATCGACTTTGGCAAGCGCAGACCGCCTCGGAGTGGTCTGAAATGTTCAACCAAGTCCATGAGTTCTCGA

ACGGGTACTCTTCAGTAGGCTCTGGGGCCAGACCACCCAGTCTCAGAGATCTCTTCCGTTACTTCCTAGATGACGAAATCGTCGTACAGGATGTACAGGAGATTCATC

TGACACCTATGCACCTGCGTCTGCTCCTCCACCCCCTGCAGACTTTGGTCTGCCAGTATTGCCAGCTTCTCAGCTGCTTTTCCGATAGCGTCGCTTCACGATCGCGGA

ATCGCGCACTGACAGCAGCGTCGACCCGCGTGCGTCTCGAGGAGGTGCAAGCGTTGCTAGGGCGTTGGTTCGATTTGGCGCAACGGTATATGAAGTCCAACCCAGT

GTGTCCGATGATGCAGGCCAACCTTGTCATGTTCCATCTTATCAGCTTGAACGCTGTGTCCAACTTTCCAGAGATAGAGCGCCTTGCGCGTCGAGAGGGCGTGGACG

GCTCATACCAGCAGATGCTGTGGATGCACAAGAAGTGTCTGTCCGATGTGCAGGAGGCCATCACCCACGCCGGTCAGGTCCTTCGACTCATCCGTGAGATGCCAAGA

GGTATCCGGCCGCCATGGTGGGCTGGCGCCGTCTACCGTGCGGGTTTGGTCTTCTGGACTGATTCGCTGGCACATGGCGAGTCCTTGAGTCCCAACCACCAAGGAT

CGTATCAGCCATCGAACTCCGCGTTCTCTGTGGATCGCCTTCCTGCTGACCACCCAACCATACTGAGGTACATGTCTCGCAGAGAGGGCATCCCCACTCTGACCAAGC

GAGACGGTACACACGTCCCCTTGGATAACGGCTTCGCGATCTTGTCGCATTGTATCGACGTCCTTGATGAGGGTGTTGCCACCAGGTTCTCCGATGGCATTCGTAGTA

AACTCGAGAAACTCGCGCGAGGATAA

>*Alternaria atra* (NA)

ATGGTTTTTTGCACATACTGTGGACAGTCGTTCACCAGAGACGAGCATCTCGAACGACATATCCTAACTCGTAAGTCGACCTTCGTTTGTCCTTCCAAATTGGTTGTCGC

AGTGCCAACTACGAACCGATCGTTCCGTAGGTTCCCCACTGACCACCACTGCCAAACAGACACCAACGTCAAGCCATTCAAATGCTTTACATGCCATATGAGCTTCGCT

CGACGGTAAGTCCTATCCTTCTTGCTCTTTCTCGCCTGGTGGTACCTCTCATATGGAACGAAATGCTGACGACCCTTCTAGAGATCTTTTGCAAAGACATTATACCGTAC

ACGGCCGCAACCAGAACAACGAAGAGGGTCTCCCACCGCAAGGGATAATACCCAAGTCCGCCGGCCGCACACCCATCGCCTGCAGTAATTGTGCAAAGACAAAGAC

GAAATGCGACAAGAAGTTTCCGTGCACCCGGTGCGCTCAAAGGAATCTGAAGTGTACTCTACGTCCTACACGGCGAGCATCCAAAGGCGTCCAGAGAGTGGGAGGTC

CTCCTGAGGCAGGGAGTGGTGACTCGAGCGAGAGTGGTAGCAACGCTGATAACCAGAATACATCCGGGAATGTCTCCCCAAACCACGATGGCCAACCGAAACAAGCA

ACCGCACAGTCGTCACCGTCTCAAATCCAGCAGCCTGCCCAGGTGTCCGACAGCCAACCTCCGTCACAAAGACAATCGCAATCGCCCTCAGCGCCCCACTCGCGGAA

TCCTTCCATCTCACACCCAGTACAACCACCACCACACATAAATCCCGAGGAAAAGTCGCTTCACATCCACATGCCCATCTCGAATACACCACCGTTTTTCGATCAGTCC

CCTTCTAACGGGCTTGCTCATTCGTCATCGTTACTCTCACCACTTCCTACACCGGTTACGGGCATGAATAGCTTTGTCTCCTCAACACCCATGTCCGGATACGACGACT

TTGTAAGGACAGTGCGCGATCAGTCAGACAATGAGAGCCCGCAATTCATCATGGATCCATGGCACTCGATGCCTATGGATACGAGTTTTGATCCCATGCGAATTGACC

CCTCGCTGATAATGACCATGGGCATGGATATGACTATGGGACCACCCCAGGACGGCATTCTCGGTATGATACCCGAAATGTCACCTGCGCAGACATTTGGACAGCCTG

TCCAGACTCCTCGCATGGTGGATGAATCTTTCTCAGACCTGCAGATTGGCAGTTCGGCTTCGATGTTCTACCCCTCCAACAGACACTCTTCAATAGCCGACGCCGGCA

TCCCCGATCTTGGCGCCATCATTGCGGCTCAAGATGGCTGGACGGTTTTCCGTTGCACTCCTTCCATCGCTTCGAGCTCATGCCCGCGAACTGCAAGGCTGAATCTTG

AGCGACTAGAGTTGAGTCTAAAGAACCACGAGGGCTGGGCTAACTGGACCCCATCATGGGACGAATCCGACTTTCAGCAAGGTGGTCAAATAGCCGTATCGAAACTAC

ATGAAGCCACACGAGACAAACTCCTCGCCATCACCCAAAGCTTTCTTCACAAGGCTCTGGACATCCATAAGGATGGAAGCAGTTCATCTAGCAGTGGCGAGTCTCCGG

GATCCACGGCCTCGCACTCTAACTTCGTTCTTCTTCCGCCTGCTCGCGTACTGGAGTACTTCCTCAAGTCGTATGCCAACTCTTTCGAAAGGTATTATCCTACCTCCGC

TAGAGGTGTTCTCGACGCCAACGAACTGATGCAAAACTACAATGACAAGGCATCAAGTTTGCTCATTCTCATGATGATTGCGCAGGGCGCCATGACTATTCCGTCCATA

GAAGCGAGATGGCTGACTGGTGGCTTCACTGAGGCATGTCGGATATCGCTCTTCGACCTCATCGAGAAGAACATTATCATGGCTGGCGATCCTATCGTCCTGCGGGC

AGCCCTTCTCTTCACTGTCCAAGCGGCGTGGAGTGGCGACAAGTGGCAGATGGACATTGCCATGGGTCAGCGCGGCATGTATTTTTCCATGTTACGACACTCCGGTAT

CCTAGATGTTCGGCCGCCGACCACACCTCATATGAATGGAAACACGTCGACGGATGTGTTGTGGAAGGACTGGCTTCAGCAAGAAAGCCGCAGTCGGTAAGTGTTGA

AAACCTCGGTACAGTCACGTGTGCACACTTACTAATATTGTGCAGCCTCATCTACTCCTGGGTTATGGTAGACCAAGACCTGTCTCTCTTCCACGATACCGCTCCTCTAT

TCTCGGTAACAGAGTTTGGTGCGCCAATGCCTGATTCAGATCAACTCTGGCAGGCGCAGACCGCTTCAGAGTGGTCGGAGATGTTCAACCAAGTCCATGAATTCTCGA

ATGGATACTCCTCGGTAGGGTCTGGAGCAAGGCCACCGAGTCTGAGAGATCTCTTCCGTTACTTCCTAGATGACGAGATAGTCGTTCAGGATGTCCAAGAGATTCATC

TGACCCCAATGCACCTGCGGCTGCTGCTTCATCCTTTGCAGACTCTGGTGTGCCAGTATTGCCAACTCCTCAGTTGCTTTTCCGATAGCGTGGCCTCTCGATCGCGAA

ATCGGGCACTGACAGCTGCTTCGACTCGCGTACGTCTAGAAGAGGTGCAGGCGTTACTGGGGCGGTGGTTCGACTTAGCGCAACGGTACATGAAGGCGAACCCAGT

GTGTCCAATGATGCAAGCCAACCTCGTCATGTTCCATCTTATCAGCATGAATGCTGTATCAAACTTCCCAGAGATTGAACGTCTCGCGCGACGAGAGGGCGTGGACGG

CTCATACCAGCAGATGATGTGGATGCACAAGAAGTGCTTGTCGGATGTGCAGGAGGCTATCACCCACGCTGGCCAAGTACTTCGTCTCATCCGTGAGATGCCGCAAG

GCATTCGCCCGCCGTGGTGGGCTGGTGCTGTCTATCGGGCAGGGTTGGTCTTCTGGACTGATTCGCTCGCTCATAGCGAGTCCTTGAGCCCCAACAACCACCAAGGA

ACGTATCAAACGTCTAATGCTGCTTTCTCTGTGGACCGCCTCCCTGCTGACCACCCAACGATACTGAGGTACATGTCACGAAGGGATGGTATCCCTACTTTGACCAAGC

GCGACGGAAACCCTGTCCCACTGGATAATGGTTTCGCTGTCCTGTCACATTGTATCGATGTACTTGACGATGGTGTCGCTACCCGATTTTCCGATGGCATTCGTAGTAA

ACTCGAGAAGCTTGCGCGGGGTTAA

>*Alternaria brassicicola* (NA)

ATGGTCTTCTGCACATATTGTGGACAGTCGTTCACCAGAGACGAGCATCTCGAACGACATATCCTAACTCGTAAGTTGGCTCTTCGTCTGTCCTTGCGAATTGGTTTTC

GCAGTGCCGACTGTGAACCGATCGTTCCGTAGCAACTACGCTGACCACCATGGCCAAACAGACACCAACGTCAAGCCATTCAAGTGCTTCACATGCCATATGAGCTTT

GCTCGACGGTACGTTCCTTCCCTCGTCTGCTTCCTTGCTCTTTCCCGTCTGGTGGCATCTCTCGTACGGAACCAAGCGCTGACGACCCTGCTAGAGACCTTTTGCAAA

GACATTATACCGTACACGGCCGCAACCAGAACAACGAAGAGGGTCTCCCACCCCAAGGGATAATCCCCAAGTCCGCCGGCCGCACGCCCATCGCCTGCAGTAATTGT

GCAAAGACCAAGACGAAATGCGATAAGAAGTTTCCGTGCACCCGGTGCGCTCAGAGGAACCTGAAGTGTACTCTACGTCCTACACGGCGAGCATCGAAAGGTATCCA

GCGAGTCGGAGTTCCTCCTGAAACAGGGAGCGGTGACTCGAGCGAGAGTGGTAGCAACGCGGATAACCAGAACACATCCGGGAATGTCTCCCCAAATCACGATGGC

CAATCGAAACAGGCAACCGCACAGTCGTCTCCATCTCAAATCCAAAAGCCTGCCCAGGTATCTGACAGCCAACCTCCGTCGCAACGACAATCGCAATCGCCCTCGGC

GCCCCACTCACGGAATCCTTCCATCTCGCATCCAGTACAACCACCATCACACATAAATCCCGAGGAAAAGTCGCTTCACATCCATATGCCGATTTCGAACACGCCCCCA

TTCTTTGACCATTCCCCTTCCAATGGACTTGCTCATTCGTCATCACTACTCTCGCCGCTTCCTACACCCGTTACGGGCATGAATAGCTTTGTCTCCTCAACACCCATGTC

CGGATACGACGACTTTGTAAGGACAGTGCGCGATCAATCGGACAATGAGAGCCCGCAATTCATGATGGATCCGTGGCACTCGATGCCCATGGACACGAGCTTCGATT

CCATGCGAATTGATCCCACGCTGATCATGAACATGGGCATGGATATGACCATGGGACCGCCCCAGGACGGCATCCTTAGTATGATCCCCGAGATGTCGCCTGCGCAG

ACATTTGGACAGCCTGTCCAGACTCCTCACATGGCGGACGAATCGTTCTCAGACCTGCACATGGGCAGTTCGGCTTCAATGTACTACCCCTCCAACAGACACTCTTCA

ATAGCGGATGCCGGCATTCCTGATCTTGGCGCCATCATTGCGGCTCAAGACGGCTGGACGGTGTTCCGTTGCACTCCTTCCATCGCTTCGAGCTCATGTCCGCGAACT

GCAAGGCTGAATCTTGAGCGACTAGAGTTGAGCCTGAAGAACCACGAAGGATGGGCCAACTGGACCCCGTCGTGGGACGAATCCGACTTCCAACAAGGCGGACAAAT

AGCAGTGTCGAAATTGCAGGAGGCAACACGAGACAAACTCCTTGCCATCACCCAAAGCTTCCTCCATAAGGCCCTGGACATTCACAAGGACGGAAACAGCTCATCTAG

CAGTGGTGAATCTCCGGGGTCCACGGCTTCTCATTCCAACTTTGTTCTTCTTCCGCCTGCTCGCGTCCTGGAGTACTTCCTCAAGTCGTATGCAAACTCTTTCGAACGG

TATTATCCTACCTCCGCTAGAGGTGTTCTCGACACCAACGAGCTGATGCAAAACTACAATGACAAGGCATCAAGCTTGCTCATCCTCATGATGATTGCGCAGGGCGCCA

TGACTATTCCGTCCATAGAAGCGAGATGGCTGACTGGTGGCTTTACCGAAGCATGTCGGATATCGCTCTTCGACCTTATTGAGAAGAATATCATCATGGCTGGTGATCC

TATCGTCTTGCGTGCCGCCCTTCTCTTCACTGTCCAAGCGGCGTGGAGTGGCGACAAGTGGCAGATGGACATTGCCATGGGTCAGCGCGGCATGTATTTCTCCATGTT

ACGACACTCTGGTATACTTGATGTTCGGCCGCCGACCACGCCTCATATGAATGGAAATACGTCGACGGATGTGTTGTGGAAGGACTGGCTTCAGCAAGAAAGCCGCA

GTCGGTAAGCGTTAAAAAAAAATCTCGGCACAGTCACGTGCGCACAGTTACTAACATCGTGCAGCCTCATCTACTCTTGGGTCATGGTAGACCAAGACCTATCGCTCTT

CCACGATACCGCTCCACTGTTCTCGGTAACAGAATTTGGCGCGCCGATGCCTGATTCAGATCAACTCTGGCAGGCACAGACCGCTTCAGAGTGGTCCGAGATGTTCAA

CCAAGTCCATGAGTTTTCGAATGGGTACTCCTCAGTAGGCTCTGGAGCAAGGCCACCGAGTCTGAGGGATCTCTTCCGTTACTTCCTAGATGACGAGATAGTCGTTCA

GGATGTTCAGGAGATTCATCTGACCCCAATGCACCTGCGGCTGCTGCTCCACCCTTTGCAGACCCTCGTGTGCCAATATTGCCAACTCCTCAGTTGCTTTTCCGACAGT

GTAGCCTCTCGATCGCGAAATCGGGCACTGACGGCCGCTTCGACTCGCGTACGTCTAGAAGAGGTGCAGGCGTTGCTGGGGCGGTGGTTCGACTTGGCGCAACGGT

ATATGAAGGCCAATCCAGTGTGTCCAATGATGCAAGCAAACCTTGTCATGTTCCATCTTATCAGCATGAATGCTGTGTCGAATTTCCCAGAGATTGAGCGCCTCGCGCG

GCGAGAGGGCGTGGACGGCTCGTACCAGCAGATGCTGTGGATGCACAAGAAGTGCTTGTCGGACGTGCAGGAGGCCATCACCCACGCTGGCCAAGTCCTTCGACTC

ATACGTGAGATGCCACGAGGCATTCGCCCGCCGTGGTGGGCTGGCGCTGTGTATCGGGCAGGGTTAGTCTTATGGACTGATTCGCTCGCCCATAACGAGTCGTTGAG

CCCCAACCACCAAGGAACATACCAGCAGCCATCTAATGCTGCTTTTTCTGTGGACCGCCTCCCTGCTGACCACCCAACCATACTGAGGTACATGTCACGAAGAGATGG

TACTCCCACTTTGACCAAGCGCGACGGTACATCCGTCCCACTCGACAACGGCTTCGCTGTCCTGTCGCATTGTATTGACGTCCTTGACGAGGGTGTCGCTACCAGATT

CTCCGATGGCATTCGTAGTAAACTCGAGAAACTTGCGCGGGGTTAA

>*Alternaria brassicae* (NA)

ATGGTCTTCTGCACATACTGTGGACAGTCGTTCACCAGAGACGAGCATCTCGAACGACACATCCTAACTCGTAAGCTGACCCTTCGTCAATCGTTCCGAACCGATTGTT

CTGTAAGGATCCCCCACTGATCACCATTGCCAAATAGACACCAACGTCAAGCCATTCAAGTGCTTTACATGCCATATGAGCTTTGCTCGACGGTACGTTCTATCCTTCAT

GCGCTTCCTTGCTCTTTCCCGCCTGGTCATGTCTCTCTTCCACAACAAATTGCTGACGATCCTTCCAGAGACCTCCTGCAAAGACATTATACCGTACACGGTCGCAATC

AGAACAACGAAGAGGGTCTTCCACCACAAGGGATAATACCCAAGTCCGCCGGCCGCACACCCATCGCCTGCAGTAACTGTGCCAAGACAAAGACCAAATGCGATAAG

AAGTTCCCGTGCACTAGGTGCGCTCAAAGGAACCTGAAGTGCACTCTACGTCCTACACGGCGAGCATCGAAAGGCGTCCAGCGAGTGGGAGGTCCTCCGGAAACTG

GGAGTGGTGACTCGAGCGAGAGTGGTAGCAACGCGGATAACCAGAACACATCCGGGAATGTCTCCCCAAACCACGATGGCCAACCGAAACAAGGAACCGCACATTC

GTCTCCTTCCCATATCCAGCAGCCTGTCCAGGTATCTGACAGCCAACCTCCGTCACAACGCCCATCGCAATCGCCGTCAGCGCCCCACTCACGGAATACGTCCATCTC

ACGTCCAGTACAACCACCACCACACATCAATCCGGAGGAAAAGTCTCTTCACATCCATATGCCGATTTCGAATACGCCACCGTTTTTCGACCAATCCCCTTCCAGCGGA

CTCGCTCATTCGTCGTCACTACTCTCACCACTCCCTACACCGGTTACGGGCATGAACAGCTTCGTCTCCTCGACACCCATGTCCGGATACGATGACTTTGTAAGGACA

GTGCGCGATCAACCAGATAACGAGAGCCCGCAGTTCATGATGGATCCTTGGCACACGATGCCCATGGACACAAACTTTGATGCCATGCGGATTGACCCCGCGCTGAT

CCTGAATATGGGCATGGATATGACCATGGGACCACCTCAGGACGGCATCCTTGGTATGATACCTGAAATGTCACCTGCGCAAACGTTTGGACAGCCTGTCCAGACTCC

TCGTATGGTAGACGAATCGTTTTCAGACTTGCATCTTGGCAGTTCGGCTTCGGTGTACTACACTTCCAATAGACACTCTTCGATAGCGGATGCCGGTATCCCTGATCTT

GGCGCCATCATTGCGGCCCAAGACGGCTGGACGGTCTTCCGTTGCACTCCCTCTATCGCGTCGAGCTCTTGTCCGCGAACTGCAAGGCTTAACCTCGAGAGACTAGA

GCTGAGCCTGAAGAACCACGAAGGATGGGCCAACTGGACTCCATCGTGGGACGAAACCGATTTCCAACAAGGCGGGCAAATAGCCGTGTCCAAGCTGCAGGAAGCC

ACCCGAGACAAACTTCTCGCCATAACCCAAAGCTTCCTCCACAAGGCTCTGGACATCCACAAGGACGGGAATAGCTCATCCAGCAGTGGTGAGTCCCCAGGATCCACA

GCTTCTAATTCCAATTTTGTTCTTCTTCCGCCTGCCCGCGTCCTAGAGTACTTCCTCAAGTCGTATGCCAACTCTTTCGAACGGTATTATCCCACCTCTGCCAGAGGTGT

TCTTGACACCAACGAGTTGATGCAAAACTATAATGACAAGGCATCAAGCTTACTCATCCTCATGATGATTGCGCAGGGCGCCATGACCATACCGTCCATAGAAGCTAGA

TGGCTCACCGGTGGCTTTACCGAAGCTTGTCGGATATCGCTCTTCGACCTCATCGAGAAGAACATCATCATGGCTGGTGATCCTATCGTTCTGCGTGCAGCCCTTCTCT

TTACTGTTCAAGCGGCATGGAGTGGCGACAAGTGGCAGATGGACATCGCCATGGGTCAACGCGGCATGTATTTTTCCATGTTGCGACACTCCGGTATCCTTGATGTTC

GGCAACCGGCGACGCCTCATATGAATGGGAATACTTCGACGGATGTGTTGTGGAAGGACTGGCTCCAGCAGGAAAGCCGCAGTCGGTAAGCGATGAAAACCTCGGTA

CAATCACGTGCGCCAAGTTACTAACATGGTGCAGCCTCATCTACTCCTGGGTCATGGTAGACCAAGACCTATCGCTGTTCCACGATACCGCTCCTCTGTTCTCGGTAAC

AGAGTTTGGTGCGCCGATGCCTGATTCAGATCAACTCTGGCAGGCGCAAACCGCTTCAGAGTGGTCGGAGATGTTCAACCAAGTCCATGAGTTCTCGAATGGTTACTC

CTCAGTAGGCTCTGGAGCAAGGCCACCAAGTCTCAGAGATCTATTCCGTTACTTCCTAGATGACGAAATAGTCGTTCAGGATGTTCAGGAGATCCATCTGACCCCAATG

CACCTGCGCCTGCTGCTACACCCTTTGCAGACCCTTGTGTGCCAATATTGCCAACTCCTCAGTTGCTTTTCCGACAGCGTAGCCTCTCGATCGCGAAATCGGGCACTG

ACGGCCGCTTCGACTCGCGTACGCCTAGAAGAGGTGCAGGCGCTGCTGGGTCGGTGGTTCGACTTGGCGCAACGGTACATGAAGGCAAATCCACCGTGCCCAATGA

TGCAAGCAAATCTCGTCATGTTCCATCTGATCAGCATGAACGCTGTATCGAATTTTCCAGAAATCGAGCGCCTCGCGCGGCGGGAGAGTGTGGACGGCTCGTACCAG

CAGATGCTGTGGATGCACAAGAAGTGCCTGTCGGATGTACAGGAGGCCATCACTCACGCTGGCCAAGTCCTTCGCCTTATCCGTGAGATGCCACGAGGCATTCGCCC

TCCGTGGTGGCCTGGTGCGGTCTATCGGGCAGGATTGGTCTTCTGGACTGAATCGCTCGCTCATAACGAATCGTTAAGCCCCAACCACCAAGGGACATATCAGCAGC

CATCTAATGCTGCTTTGGCTGTGGATCGTCTTCCTGCTGACCACCCAACCATACTGAGGTACATGTCACGAAGGGAGGGTATCCCCACTTTGACCAAGCGCGACGGTA

CATCCGTCTCACTCGACAACAGCTACGCTGTCCTGTCGCATTGTCTTGATCTCCTTGACGAGGGTGTCGCTACCAGATTTTCCGACGGCATTCGTAGTAAACTTGACAA

ACTTGCGCGGGGTTAA

>*Alternaria burnsii* (XM_038925615[a])

ATGGTCTTCTGCACATATTGTGGACAGTCGTTCACCAGGGACGAACATCTCGAACGACATATCCTAACTCGTAAGCCAACTCTTGCTCTGTCCATGAGGGTGGATTTTC

GCAATGTCGAGTTCAATCCGATCGTTCCGTAGCATCTCCGCTGACCATCAATGCCAAACAGACACCAATGTCAAGCCTTTCAAGTGCTTTACATGTCATATGAGCTTTG

CTCGACGGTAAGTCCCACCCTGCGTGCGCTCCCTCCTCTTCTTGTTTGGTCATAATCTCCGTATTGAACGAAACGCTGACAATCTTTCCAGAGATCTTTTGCAAAGACAT

TACACCGTACACGGCCGTAACCAGAACAACGAAGAAGGCCTCCCACCGCAAGGCATCATACCTAAGTCCGCCGGTCGCACACCCATCGCCTGCAGTAATTGTGCAAA

GACGAAGACGAAATGCGACAAGAAGTTTCCTTGCACCCGGTGCGCTCAAAGGAACCTGAAGTGTACTCTACGTCCTACACGGCGAGCATCGAAAGGTGTCCAACGAG

TAGGAGGTCCTCCTGAAACAGGCAGTGGTGATTCGAGCGAGAGTGGTAGCAACGCGGATAACCAGAACACATCCGGCAATGTCTCTCCAAACCACGATGGCCAGCCG

AAACAAGCAACCGCTCAGTCGTCTCCATCTCAAATTCAGCAGCCTAGCCAGATTTCCGACAGCCAACCTCCATCGCAACGGCAATCGCAATCTCCCTCAGCGCCCCAC

TCGCGGAATCCTTCCATCTCACATCCAGTGCAACCACCACCGCACATGAACCCCGAGGAGAAGTCGCTCCACATACACATGCCTATCTCGAATACTCCCCCGTTCTTC

GATCAGTCACCCTCTAATGGACTGTCTCATTCCTCATCATTGCTCTCACCTCTTCCCACACCGGTTACGGGCATGAATAGTTTTGTTTCTTCGACACCCATGTCTGGGTA

CGACGACTTCGTTAGGACAGTGCGCGATCAACCGGAAAATGAGAGCCCGCAATTCATGATGGATCCTTGGCACTCGATGCCCATGGATACGAGTTACGATCCTATGCG

GATTGACCCCGCTCTGATGATGACCATGGGCATGGACATGACGATGGGACCGCCCCAAGACGGTATCCTTAGTATGATACCCGAAATGTCGCCTGCACAAACATTCGG

ACAGCCTGTTCAGACTCCTCGTATGGTGGACGAATCGTTCTCGGACTTGCAAATCGGCAGTTCGGCTTCGATGTTCTATCCCTCTAACAGACATTCTTCAATAGCGGAC

GCCGGCATCCCTGATCTTGGCGCCATTATCGCAGCCCAAGACGGCTGGACGGTCTTCCGTTGCACTCCTTCTATCGCTTCAAGCTCATGTCCGCGGACTGCAAGGCT

GAATCTTGAGCGATTAGAGTTGAGCCTGAAGAACCATGAGGGCTGGGCTAACTGGACCCCATCGTGGGATGAATCTGATTTCCAACAAGGCGGACAGATCGCCGTGT

CAAAACTCCAGGAAGCCACACGAGACAAACTCCTCGCGATCACACAGAGCTTCCTTCACAAGGCTCTAGATATCCACAAAGACGGAAGCAGCTCGTCCAGCAGTGGC

GAGTCTCCAGGATCTACGGCTTCACATTCCAACTTCGTTCTGCTTCCGCCTGCTCGTGTCCTGGAGTACTTCCTCAAGTCGTACGCCAACTCTTTTGAACGCTATTATC

CTACCTCGGCCAGAGGAGTCCTCGACACCAACGAGTTGATGCAGAACTACAATGACAAAGCTTCGAGCTTGCTCATTCTCATGATGATTGCGCAGGGCGCCATGACCA

TTCCGTCCATCGAAGCGAGATGGCTGACTGGTGGCTTTACTGAGGCATGTCGGATATCGCTGTTCGACCTCATCGAGAAGAACATCATTATGGCTGGCGATCCTATTG

TCCTGAGGGCGGCCCTTCTCTTTACTGTTCAAGCGGCGTGGAGTGGCGACAAGTGGCAGATGGACATCGCCATGGGTCAGCGCGGCATGTATTTTTCCATGTTACGA

CATTCCGGCATCCTCGATGTTCGGCCGCCTACCACGCCTCACATGAATGGAAACACTTCGACGGATGTGCTGTGGAAGGACTGGCTTCAGCAAGAAAGCCGCAGCCG

GTAAGTGTAGAAAACCTCGGTACAGTCACGTGCGCGCACTCACTAATATCGTGTAGCCTCATCTACTCATGGGTCATGGTAGACCAAGACCTATCGCTATTCCACGATA

CTGCTCCCCTGTTTTCGGTAACAGAATTTGGTGCGCCAATGCCTGATTCAGATCAACTCTGGCAGGCGCAGACAGCTTCAGAGTGGTCGGAGATGTTCAACCAGGTCC

ATGAATTCTCGAATGGATACTCCTCAGTAGGCTCTGGAGCAAGACCACCGAGTCTCAGAGATCTCTTCCGCTACTTCCTAGATGACGAGATAGTCGTCCAGGATGTTCA

GGAGATTCATCTGACCCCGATGCACCTGCGACTACTACTGCACCCTCTGCAGACCCTTGTGTGCCAATATTGCCAACTCCTCAGTTGCTTTTCCGACAGTGTAGCCTCT

CGATCGCGGAATCGGGCATTGACAGCTGCTTCGACTCGCGTACGCCTAGAAGAGGTGCAGGCGTTATTGGGGCGGTGGTTCGACTTGGCGCAACGGTACATGAAGG

CGAACCCAGTATGTCCGATGATGCAAGCAAATCTCGTCATGTTCCATCTTATCAGCATGAATGCTGTGTCAAATTTCCCAGAGATTGAGCGCCTCGCCCGACGGGAGG

GCGTGGACGGCTCGTATCAACAGATGCTGTGGATGCACAAGAAGTGCTTGTCGGATGTGCAGGAGGCCATCACTCACGCTGGCCAAGTTCTTCGCCTCATTCGTGAG

ATGCCACGAGGCATTCGCCCGCCGTGGTGGGCTGGTGCTGTCTATCGGGCAGGGTTGGTCTTCTGGGCTGACTCGCTCGCTCACAACGAGTCCTTGAGTCCCAACCA

TCAAGGGACATATCAGCCATCTAATACTGCTTTCTCTGTGGATCGCCTCTCTGCTGACCACCCAACCATACTGAGGTACATGTCACGGAGGGAGGGTATTCCCACCTTG

ACTAAGCGCGATGGTAGCCCCGTTCCATTGGATAACGGCTTTGCCGTCCTGTCGCATTGTATCGATGTCCTTGACGAGGGTGTCGCCACCAGGTTTTCCGATGGCATT

CGTAGTAAACTCGAGAAACTTGCGCGCGGTTAA

>*Alternaria capsici* (NA)

ATGGTCTTCTGCACATATTGTGGACAGTCGTTCACCAGGGACGAGCATCTCGAACGACATATTCTAACTCGTAAGTTGGCCCTTGCTCCGTCCTTGAGGCTTGATTTCC

GCAGTGACGAGTACAGTCCGGTCCTTCCGTGGGATCTTCGCTAACCGCCATTGCCAAACAGACACAAATGTCAAGCCCTTCAAGTGCTTTACATGCCATATGAGTTTCG

CTCGACGGTCAGTCTTACCCTCCATGCACTCCTTGCTCTTTCTCGGCCGATGGCAATCCTCGTAGGGAACGAAACGCTGATGATCCTCCCAGAGATCTTTTGCAAAGA

CATTATACCGTCCACGGCCGCAACCAGAACAACGAGGAAGGTCTGCCACCGCAAGGGATCATACCTAAATCCGCTGGTCGCACACCCATCGCCTGCAGTAATTGTGC

AAAGACGAAGACGAAATGCGACAAGAAGTTTCCCTGCACCCGGTGCGCTCAGAGGAACCTGAAATGTACTCTGCGTCCTACACGTCGAGCATCGAAAGGTGTCCAGC

GAGTGGGGGGTCCTCCTGAAACAGGGAGTGGTGATTCGAGCGAGAGTGGTAGCAATGCGGACAACCAGATCACATCCGGCAATGTCTCTCCAAACCACGACGGTCAA

TCGAAACAAGCAACCGCACAGTCATCTCCATCTCAGATTCAGCAGCCTGCCCTGATGTCTGACAGCCAACCTCCGTCACAACGACAATCGCAGTCGCCTTCAGCGCCC

CACTCGCGGAACCCTTCCATCTCGCATCCAGTGCAACCACCACCGCACATGAACCCCGAGGAGAAGTCGCTCCATATACACATGCCGATTTCAAATACTCCCCCATTC

TTCGACCAGTCACCGTCCAATGGCCTAGCTCATTCCTCGTCATTACTTTCGCCTCTTCCCACACCGGTTACGGGCATGAATAGCTTTGTCTCCTCGACACCCATGTCTG

GATACGACGATTTTGTCAGAACAGTGCGCGATCAGCCGGAGAATGAGAGTCCGCAATTCATGATGGATCCTTGGCATTCGATGCCTATGGACACGAACTTCGATCCTA

TGCGGATTGACCCCACTCTGATGATGACCATGGGCATGGATATGACCATGGGACCACCCCAAGACGGTATTCTTAGTATGATACCCGAAATGTCACCTGCGCAGACGT

TCGGACAACCTGTTCAGACTCCTCGCATGGTGGACGAATCATTCTCAGACTTGCAGATTGGCAGTTCGGCTTCGATGTTCTACCCTTCCAACAGACATTCTTCGATAGC

GGATGCCGGCATCCCTGATCTTGGAGCTATCATCGCGGCTCAAGACGGCTGGACTGTTTTCCGCTGCACTCCTTCTATTGCTTCCAGCTCATGTCCGCGGACTGCAAG

GCTGAATCTTGAGCGCCTAGAGTTGAGCCTGAAGAACCATGAGGGTTGGGCTAACTGGGCTCCGTCCTGGGATGAATCCGATTTCCAACAAGGCGGGCAAATAGCCG

TGTCGAAATTACAGGAAGCCACACGAGACAAACTCCTCGCCATCACACAGAGCTTTCTTCACAAAGCCCTAGACATCCACAAAGACGGAAACAGCACGTCCAGTAGTG

GTGAATCTCCAGGGTCCACGGCTTCGCATTCCAACTTTGTCCTTCTTCCGCCTGCTCGCGTCCTGGAGTACTTCCTCAAGTCGTACGCCAACTCTTTCGAACGCTATTA

TCCTACCTCCGCCAGAGGAGTCCTTGACGCCAACGAGTTGATGCAGAACTATAATGACAAGGCATCAAGCTTGCTCATTCTCATGATGATCGCGCAGGGCGCCATGAC

TATTCCGTCCATAGAAGCGAGATGGCTGACCGGTGGGTTTACCGAGGCATGTCGGATATCGCTGTTCGACCTGATCGAGAAGAACATTATCATGGCTGGCGATCCTAT

CGTCCTAAGAGCGGCCCTTCTCTTTACTGTCCAAGCGGCGTGGAGTGGCGACAAGTGGCAGATGGACATCGCCATGGGTCAGCGCGGCATGTATTTTTCCATGTTAC

GGCACTCCGGTATTCTTGATGTCCGACCGCCTACCACGCCTCACATGAACGGAAACACGTCGACGGATGTGTTGTGGAAGGACTGGCTCCAGCAAGAAAGCCGCAGC

CGGTAAGTGATGAAAACCTCGGTACAGTCACGTGCGGACACTTACTAATATCATGTAGCCTCATCTACTCTTGGGTCATGGTAGACCAGGACCTATCGCTGTTCCATGA

TACCGCCCCGCTGTTCTCGGTAACAGAATTCGGCGCGCCAATGCCTGATTCAGACCAGCTCTGGCAGGCGCAGACTGCTTCAGAGTGGTCGGAGATGTTCAACCAAG

TGCATGAATTCTCCAATGGGTGCTCCTCAGTAGGCTCTGGAGCAAGACCACCGAGTCTCAGAGATCTCTTCCGGTACTTCCTAGATGACGAGATAGTCGTCCAGGATG

TCCAGGAGATTCATCTGACCCCAATGCACCTGCGGCTGCTGCTCCACCCTCTGCAGACCCTTGTGTGCCAGTATTGTCAACTCCTCAGCTGCTTTTCAGATAGCGTAG

CCTCTCGATCGAGGAATCGGGCACTGACTGCCGCTTCGACTCGCGTGCGCTTAGAAGAGGTACAGGCCCTACTGGGGCGGTGGTTCGACTTGGCGCAAAGATACAT

GAAGGCGAACCCAGCATGTCCAATGATGCAGGCAAACCTCGTCATGTTCCATCTGATCAGCATGAATGCTGTGTCGAACTTCCCAGAGATCGAGCGCCTCGCGCGAC

GAGAGGGCGTGGACGGCTCGTACCAGCAGATGTTGAGGATGCACAAGAAGTGCTTGTCGGATGTGCAGGAGGCCATAACCCATGCTGGTCAAGTTCTTCGCCTCATT

CGTGAGATGCCACGAGGCATCCGCCCGCCGTGGTGGGCTGGTGCTGTCTATCGGGCAGGGTTGGTGTTTTGGACTGATTCACTCGCCCGTAACGAGTCTCTGAGCC

CCAATCACCAAGGAGCATATCAGCAGCCATCTAATACTGCTTTCTCTGTGGATCGCCTTTCTGCTGACCACCCTACCATACTGAGGTACATGTCACGAAGGGAGGGCAT

CCCCACCTTGACCAAGCGTGACGGAAACCCCGTTCCACTGGATAACGGCTTCGCTATCCTGTCGCATTGTATCGATGTCCTTGACGAGGGCGTCGCCACCAGATTTTC

CGATGGCATTCGTAGTAAACTCGAGAAACTTGCGCGTGGTTGA

>*Alternaria carthami* (ACM_PT10841[b])

ATGGTCTTCTGCACATATTGTGGACAGTCGTTCACCAGGGACGAGCATCTCGAACGACATATCCTGACTCGTAAGTTGGCTCTTGCTCCGTCCTTGAGGATTGATTTCC

GCCGTGACGAGTACAGTCCGGTCGTTCCGTGGGACCTTCGCTAACCGCCGTTGCCAAACAGACACAAATGTCAAGCCCTTCAAGTGCTTTACATGCCATATGAGCTTT

GCTCGACGGTAAGTCTTACCCTTCATGTGCTCCTTGCTCTTTCTCCGCCGGTGGCAACCCTTGTAGCGAACGAAACGCTGACGATCCTCCCAGAGATCTTTTGCAAAG

ACATTACACCGTCCACGGCCGCAACCAGAACAACGAGGAAGGTCTACCACCACAAGGGATCATACCTAAGTCCGCTGGCCGCACACCCATCGCCTGCAGCAATTGTG

CAAAGACGAAGACGAAATGCGACAAGAAGTTTCCTTGCACCCGGTGCGCTCAGAGGAACCTGAAATGTACTCTGCGTCCTACACGTCGAGCATCGAAAGGTGTCCAG

CGAGTGGGGGGTCCTCCTGAAACAGGGAGTGGTGATTCGAGCGAGAGTGGTAGCAATGCGGACAACCAGATCACATCCGGCAATGTCTCTCCAAACCACGATGGCC

AATCGAAACAAGCAACCGCACAGTCATCTCCATCTCAGATTCAGCAGACTGCCTTGATGACTGACAGCCAACCTCCGTCACAACGACAATCGCAATCGCCTTCAGCGT

CCCACTCGCGGAATCCCTCCATCTCGCATCCAGTGCAACCACCACCGCACATGAATCCCGAGGAGAAGTCGCTCCACATACACATGCCGATCTCAAATACTCCCCCGT

TCTTCGACCAGTCACCGTCCAATGGCCTAGCTCATTCCTCGTCATTACTTTCGCCTCTTCCCACTCCGGTTACGGGCATGAATAGCTTTGTCTCCTCGACACCAATGTC

TGGATACGACGATTTCGTCAGGACAGTGCGCGATCAGCCGGAGAATGAGAGCCCGCAGTTCATGATGGATCCTTGGCATTCGATGCCTATGGATACGAGCTTCGATC

CTATGCGGATTGACCCCGCTCTGATGATGACCATGGGCATGGATATGACTATGGGACCACCCCAAGACGGCATTCTCAGTATGATACCCGAAATGTCACCTGCGCAGA

CATTCGGGCAGCCTGTCCAGACTCCTCGCATGGTGGACGAATCTTTCTCGGACTTGCAGATTGGCAGTTCGGCTTCGATGTTCTACCCTTCCAACAGACATTCTTCAAT

AGCGGACGCCGGCATCCCTGATCTCGGAGCCATTATCGCGGCTCAAGACGGCTGGACGGTTTTTCGTTGCACTCCTTCTATTGCTTCCAGCTCATGTCCGCGGACAG

CGAGGCTGAATCTTGAGCGACTAGAGTTGAGCCTGAAGAACCATGAAGGGTGGGCCAACTGGTCACCATCCTGGGATGAATCCGATTTTCAACAAGGCGGGCAAATA

GCCGTGTCGAAATTACAGGAAGCCACACGAGACAAACTCCTCGCCATCACACAGAGCTTTCTTCACAAAGCTCTAGACATCCACAAAGACGGAAGCAGCACGTCCAGT

AGTGGTGAATCTCCAGGGTCCACGGCTTCGCATTCCAATTTCGTCCTTCTTCCGCCTGCTCGCGTCCTGGAGTACTTCCTCAAGTCGTACGCCAACTCTTTCGAACGCT

ACTACCCTACCTCCGCCAGAGGAGTCCTCGACGCCAACGAGTTGATGCAGAACTACAATGACAAGGCATCAAGCTTGCTCATTCTCATGATGATTGCGCAGGGCGCCA

TGACCATTCCGTCCATAGAAGCGAGATGGCTGACTGGTGGGTTTACCGAGGCATGTCGGATATCACTGTTCGACCTGATCGAGAAGAACATCATCATGGCTGGCGATC

CTATCGTCCTGAGAGCGGCCCTTCTCTTCACTGTCCAAGCGGCGTGGAGTGGCGACAAGTGGCAGATGGACATCGCCATGGGTCAGCGCGGCATGTATTTTTCCATG

TTACGGCACTCCGGTATTCTTGATGTTCGGCCACCTACCACGCCTCACATGAACGGAAACACGTCGACGGATGTGTTGTGGAAGGACTGGCTTCAGCAAGAAAGCCG

CAGCCGGTAAGTGTTGAAAACCTCGGTACAGTCACGTGCGCACACTTACTAATATGATGTAGCCTCATCTACTCTTGGGTCATGGTGGACCAAGATCTATCGCTCTTCC

ACGATACTGCCCCACTGTTCTCGGTAACAGAATTCGGCGCGCCAATGCCGGATTCAGATCAGCTCTGGCAGGCACAGACCGCTTCAGAGTGGTCGGAGATGTTCAAC

CAAGTGCATGAATTCTCCAATGGGTACTCCTCAGTAGGCTCTGGAGCAAGACCACCGAGTCTCAGAGATCTCTTCCGTTACTTCCTAGATGACGAGATAGTCGTCCAG

GATGTCCAGGAGATTCACTTGACCCCGATGCACCTGCGGCTGCTGCTCCACCCTCTGCAGACCCTTGTGTGCCAGTATTGTCAACTCCTCAGCTGCTTTTCAGATAGC

GTAGCCTCTCGATCGAGGAATCGGGCACTGACTGCCGCTTCGACTCGCGTGCGCCTAGAAGAGGTACAGGCCTTGCTGGGGCGGTGGTTCGACTTGGCGCAACGAT

ACATGAAGGCGAACCCAGTATGTCCGATGATGCAGGCAAACCTCGTCATGTTCCATCTGATCAGCATGAATGCTGTGTCGAACTTCCCAGAGATTGAGCGCCTCGCGC

GACGAGAGGGCGTGGACGGCTCGTACCAGCAGATGCTGTGGATGCACAAGAAGTGCTTGTCGGATGTGCAGGAGGCCATAACACATGCTGGTCAAGTTCTTCGCCTC

ATCCGTGAGATGCCACGNNNNNNNNNNATCCGCCCGCCGTGGTGGGCTGGTGCTGTCTATCGTGCAGGATTGGTGTTCTGGACTGATTCGCTCGCTCATAACGAGTC

TCTGAGCCCCAACCACCAAGGAACATATCAGCAGCCATCGAATACTGCTTTCTCTGTGGATCGCCTTTCTTCTGACCACCCTACCATACTGAGGTACATGTCACGAAGA

GAGGGTATCCCCACCTTGACCAAGCGTGACGGAAACCCCGTTCCACTGGATAACGGCTTCGCTATCCTGTCGCATTGTATCGATGTCCTTGACGAGGGCGTCGCCAC

CAGATTTTCCGATGGCATTCGTAGTAAACTCGAGAAACTTGCGCGTGGTTGA

>*Alternaria citriarbusti* (NA)

ATGGTCTTCTGCACATATTGTGGACAGTCGTTCACCAGGGACGAACATCTCGAACGACATATCCTAACTCGTAAGTCAACTCTTGCTCTGTCCATGAGGGTGGATTTCC

GCAATGTCGAGTTCAATCCGATCGTTCCGTAGCATCTCCGCTGACCGTCAATGCCAAACAGACACCAATGTCAAGCCATTCAAGTGCTTTACATGTCATATGAGCTTTG

CTCGACGGTAAGTCCCACCCTGCGTGCGCTCCTTCCTCTTCCTGTTTGGTCATAATTTCCGTACCGAACGAAACGCTGACAATCTTTCCAGAGATCTTTTGCAAAGACA

TTACACCGTACACGGCCGCAACCAGAACAACGAAGAAGGCCTCCCACCGCAAGGCATCATACCTAAGTCCGCCGGCCGCACACCCATCGCCTGCAGTAATTGTGCAA

AGACGAAGACGAAATGCGACAAGAAGTTTCCTTGCACCCGGTGCGCTCAAAGGAACCTGAAGTGTACTCTACGTCCTACACGGCGAGCATCGAAAGGTGTCCAACGA

GTAGGAGGTCCTCCTGAAACAGGCAGTGGTGATTCTAGCGAGAGTGGTAGCAACGCGGATAACCAGAACACATCCGGCAATGTCTCTCCAAACCACGATGGCCAGTC

GAAACAAGCAACCGCTCAGTCGTCTCCATCTCAAATTCAGCAGCCTAGCCAGATTTCTGACAGCCAACCTCCATCGCAACGGCAATCGCAATCTCCCTCAGCGCCCCA

CTCGCGGAATCCTTCCATCTCACATCCAGTGCAACCACCACCGCACATGAACCCCGAGGAGAAGTCGCTCCACATACACATGCCTATCTCGAATACTCCCCCGTTCTT

CGATCAGTCACCCTCTAACGGACTGTCTCATTCCTCATCATTACTTTCACCTCTTCCCACACCAGTTACGGGCATGAATAGTTTTGTTTCTTCGACACCCATGTCTGGGT

ACGACGACTTCGTCAGGACAGTGCGCGATCAACCAGAAAATGAGAGCCCGCAATTCATGATGGATCCTTGGCACTCGATGCCCATGGATACGAGTTACGATCCTATGC

GGATTGACCCCGCTCTGATGATGACCATGGGCATGGACATGACGATGGGACCGCCCCAAGACGGTATCCTTAGTATGATACCCGAAATGTCACCTGCGCAAACATTCG

GACAGCCTGTTCAGACTCCTCGTATGGTGGACGAATCGTTCTCGGACTTGCAAATCGGCAGTTCGGCTTCGATGTTCTACCCCTCTAACAGACATTCCTCAATAGCGGA

CGCCGGCATCCCTGATCTTGGCGCCATTATCGCAGCCCAAGACGGCTGGACGGTCTTCCGTTGCACTCCTTCTATCGCTTCGAGCTCATGTCCGCGGACCGCAAGGC

TGAATCTTGAGAGATTAGAGTTGAGCCTGAAGAACCATGAGGGCTGGGCTAACTGGACCCCATCGTGGGACGAATCTGATTTCCAACAAGGCGGACAGATCGCCGTG

TCAAAACTCCAGGAAGCCACACGAGACAAACTTCTCGCGATCACACAGAGCTTCCTTCACAAGGCTCTAGATATCCACAAAGATGGGAGCAGCTCGTCTAGTAGTGGC

GAGTCTCCAGGATCTACGGCTTCACATTCCAACTTCGTTCTTCTTCCGCCTGCTCGTGTCCTGGAGTACTTCCTCAAGTCGTACGCCAACTCTTTCGAACGCTATTATCC

TACATCGGCCAGAGGAGTCCTCGACACCAACGAGTTGATGCAGAACTACAATGACAAAGCTTCGAGCTTGCTCATTCTCATGATGATTGCGCAGGGCGCTATGACCAT

TCCGTCCATAGAAGCGAGATGGCTGACTGGCGGCTTTACTGAGGCATGTCGGATATCCCTGTTCGACCTCATCGAGAAGAACATCATTATGGCTGGCGATCCTATTGT

CCTGAGGGCGGCCCTTCTCTTCACTGTTCAAGCGGCGTGGAGTGGCGACAAGTGGCAGATGGACATCGCCATGGGTCAGCGCGGCATGTATTTTTCCATGTTACGAC

ATTCCGGTATTCTCGATGTTCGACCGCCTACCACGCCTCACATGAATGGAAACACTTCGACGGATGTGTTGTGGAAGGACTGGCTTCAGCAAGAAAGCCGCAGCCGGT

AAGTGTTGAAAACCTCGGTACAGTCACGTGCGCGCACTTACTAATATCGTGTAGCCTCATCTACTCTTGGGTCATGGTAGACCAAGACCTATCGCTATTCCACGATACC

GCTCCCCTGTTTTCGGTAACAGAATTTGGCGCGCCAATGCCTGATTCAGATCAACTCTGGCAGGCGCAGACAGCTCCAGAGTGGTCGGAGATGTTCAACCAGGTCCAT

GAATTCTCGAATGGATACTCCTCAGTAGGCTCTGGAGCAAGACCACCGAGTCTCAGAGATCTCTTCCGCTACTTCCTAGATGACGAGATAGTCGTCCAGGATGTTCAG

GAGATTCATCTGACCCCGATGCACCTGCGACTACTACTGCACCCTCTGCAGACCCTTGTGTGCCAATATTGCCAACTCCTCAGTTGTTTCTCCGACAGTGTAGCCTCTC

GATCGCGGAATCGGGCATTGACAGCTGCTTCGACTCGCGTACGCCTAGAAGAGGTACAGGCGTTATTGGGGCGGTGGTTCGACTTGGCGCAACGGTACATGAAGGC

GAACCCAGTATGTCCGATGATGCAAGCAAATCTCGTCATGTTCCATCTTATCAGCATGAATGCTGTGTCAAATTTCCCAGAGATTGAGCGCCTCGCCCGACGGGAGGG

TGTGGACGGCTCGTATCAACAGATGCTGTGGATGCACAAGAAGTGCTTGTCGGATGTGCAGGAGGCCATCACTCACGCTGGCCAAGTTCTTCGCCTCATTCGTGAGAT

GCCACGAGGCATTCGCCCGCCGTGGTGGGCTGGTGCTGTCTATCGGGCAGGGTTGGTCTTCTGGGCTGACTCGCTCGCTCACAACGAGTCCTTGAGTCCCAACCAT

CAAGGGACATATCAGCCATCTAATACTGCTTTCTCTGTGGATCGCCTCTCTGCTGACCACCCAACCATACTGAGGTACATGTCACGGAGGGAGGGTATTCCCACCTTG

ACCAAGCGCGATGGTAGCCCTGTTCCACTGGATAACGGCTTTGCCGTCCTGTCGCATTGTATCGATGTCCTTGACGAGGGTGTCGCCACCAGGTTTTCCGATGGCATT

CGTAGTAAACTCGAGAAACTTGCGCGCGGTTAA

>*Alternaria conjuncta* (NA)

ATGGTCTTTTGCACATACTGTGGACAGTCGTTCACCAGAGACGAGCATCTCGAACGACATATCTTGACTCGTATGTTGACCCCGCGTCTTTCGCTCTTTCAATCTTCCTC

ACACCAGCTACCATCCAATCTTTCTTCTGGGATATCTGCTAATCACTGTGGATGAGCAGACACCAACGTCAAGCCTTTCAAATGCTTCACATGTCATATGAGCTTTGCTC

GACGGTATGTGGCACCCTTCCTTGCTCTTTCTCATCCTGTGGCATCGACCGTTCGGAACAAAGTACTGACAGCCTCTTCAGAGATCTTTTGCAAAGACACTACACCGTA

CACGGCCGCAACCAGAACAACGAAGAGGGACTCCCCCCACAAGGCATAATACCAAAGTCCGCCGGCCGCACACCCATCGCCTGCAGTAATTGTGCCAAGACAAAGAC

CAAGTGTGACAAGAAGTTTCCGTGCACCAGGTGCGCTCAGAGGAATCTGAAATGCACATTGCGTCCTACAAGGCGGGCATCGAAAGGTGTCCAGCGGGTTGGCGGTC

CTCCTGAGGCTGGGAGCGGGGACTCGAGCGAGAGTGGCAGCATTGCGGATAACCACAATACATCCGGCAATGTCTCTCCAAACCACGATAGCCAACCGAAACAAGCG

ATGTCGAAGTCCTCTCCTGCTCAGATCAACCAGACTGCCCATATTCCGGACAGCCAACCACCGTCGCAAAGACAATCGCAGTCGCCCTCGGCCCCTCATTCACGAAAC

CCTTCCATCTCACAACCGGTACAGCCACCGACACACATAAATCCCGAGGAGAAGTCGTTGCATCTTCATATGCCGATCTCCGATACGCCGCCCTTTTTCGACCAGTCC

CCTTCCAACGGACTTGCTCAATCATCGTCGCTTCTTTCGCCACTCCCCACCCCGGTGAACGGCATGAATGGATTCGTCACCTCCACACCTATGTCAGGATATGACGACT

TCGTCAGAACGGTACGGGACCAATCCGATAATGAGAGCCCTCAATTCATGATGGACCCTTGGAACGGCATGCCCATGGATACAACCTTTGATCCCATGCGGATAGACC

CCGCGTTGATGATGAGCATGAGTATGGACATGAGCATGGGACCACCTCAGGATGGCATCCTTGGCATGATACCAGAAATGTCGCCTGCTCAAACATTTGGACAGCCTG

TCCAGACACCTCGCATGGACGAGTCCTTTTCGGACCTGCAAATTGGTAGTTCGGCTTCGATGTTCTACCCCTCCAACAGGCACTCTTCGATAGCAGATGCCGGCATAC

CTGATCTTGGTGCCATCATCGCGGCTCAAGATGGCTGGACTGTTTTCCGTTGCACTCCTTCTATTGCTTCAAGCTCGTGTCCACGCACAGCGAGACTCAACCTCGAGC

GGTTAGAGCTGAGCCTCAAGAATCACGAGGGCTGGGCTAACTGGTCCCCATCGTGGGATGAATCCGACTTTCAACAAGGCGGGCAAATCGCGGTATCGAAGTTGCAG

GAAGCCACGCGAGACAAACTTCTCGCCATCACCCAAAGTTTCCTCCACAAGGCTTTGGACATCCACAAAGACGGAAGCAGCTCGTCCAGCAGTGGCGAGTCTCCAGG

ATCCACGGCCTCTCACTCCAACTTCGTTCTTCTTCCACCTGCTCGCGTCCTGGAGTACTTTCTCAAGTCATACGCCAACTCTTTCGAACGCTATTATCCCACCTCCGCCA

GAGGTGTGCTCGATACGAATGAACTAATGCAAAACTACAACGACAAAGCGTCGAGCTTGCTCATTCTCATGATGATCGCGCAGGGCGCCATGACCATTCCATCCATAG

AGGCCAGGTGGCTAACTGGTGGCTTCACCGAAGCCTGCCGGATATCGCTCTTCGACCTTATTGAGAAGAACATCATCATGGCTGGTGATCCTATCGTTCTGCGTGCAG

CCCTCCTCTTCACTGTCCAGGCGGCGTGGAGTGGCGACAAATGGCAGATGGACATCGCTATGGGCCAGCGTGGCATGTATTTCTCCATGTTGCGACATTCCGGTATTC

TCGATGTGCGACCGCCGGCAACGCCTCATATCAACGGAAATATTTCGACGGATGCGTTGTGGAAGGACTGGCTTCAGCAAGAAAGCCGCAGTCGGTAAGTGTTGAAA

ACTTCGGTACCGTCACGTGAACGCACTTACTAATATTGGCGCAGCCTCATCTACTCCTGGGTCATGGTAGACCAAGACTTATCGCTCTTCCACGATACCGCTCCACTGT

TCTCAGTAACAGAGTTTGGCGCGCCGATGCCTGATTCGGATCGACTTTGGCAAGCGCAGACCGCCTCAGAGTGGTCTGAGATGTTCAACCAAGTCCATGAGTTCTCGA

ACGGGTACTCTTCAGTAGGCTCTGGGGCCAGACCACCCAGTCTCAGAGACCTGTTCCGTTACTTCCTAGATGACGAAATCGTCGTACAGGATGTACAGGAGATTCATC

TGACACCTATGCACCTGCGTCTGCTCCTCCACCCCCTGCAGACTCTGGTCTGCCAGTACTGCCAGCTTCTCAGCTGCTTTTCCGATAGCGTCGCTTCACGATCGCGGA

ATCGCGCACTGACAGCAGCGTCGACCCGCGTGCGTCTCGAGGAGGTGCAGGCGTTGCTAGGGCGTTGGTTCGATTTGGCGCAACGGTACATGAAGTCCAACCCAGT

GTGTCCGATGATGCAGGCGAACCTTGTCATGTTCCATCTTATCAGCTTGAATGCTGTGTCCAACTTTCCAGAGATAGAGCGCCTTGCGCGTCGAGAGGGTGTGGACGG

CTCATACCAGCAGATGCTGTGGATGCACAAGAAGTGTCTGTCCGATGTGCAGGAGGCCATCACCCACGCCGGTCAGGTCCTTCGACTCATCCGTGAGATGCCAAGAG

GTATCCGGCCGCCATGGTGGGCTGGCGCCGTCTACCGTGCGGGTTTGGTCTTCTGGACTGATTCGCTGGCACATGGCGAGTCCTTGAGTCCCAACCACCAAGGATC

GTATCAGCCATCGAACTCCGCGTTCTCTGTGGATCGCCTTCCAGCTGACCACCCTACCATACTGAGGTACATGTCTCGTAGGGAGGGCATACCCACTCTGACCAAGCG

CGACGGTGCACACGTCCCCTTGGATAACGGCTTCGCGATCCTGTCGCATTGTATCGACGTCCTTGATGAGGGTGTTGCCACCAGGTTCTCCGATGGCATTCGTAGTAA

ACTCGAGAAACTCGCACGAGGATAA

>*Alternaria consortialis* (NA)

ATGGTCTTCTGCACATACTGTGGACAGTCGTTCACCAGAGACGAGCATCTCGAACGACATATCTTAACTCGTAAGTCGACCTTCGTTTGTCCTTCCAAATTGGTTGTCG

CAGTGCCAACTACGAACCGATCGTTCCGTAGATTCCCCACTGACCACCACTGCCAAACAGACACCAACGTCAAGCCATTCAAATGCTTTACATGCCATATGAGCTTCGC

TCGACGGTAAGTCCTATCCTTCTTGCTCTTTCTCGTCTGGTGGTATCTCTCATATGGAACGAAATGCTGACGACCCTTCTAGAGATCTTTTGCAAAGACATTATACCGTA

CACGGCCGCAACCAGAACAACGAAGAGGGTCTCCCACCGCAAGGGATAATACCCAAGTCCGCCGGCCGCACACCCATCGCCTGCAGTAATTGTGCAAAGACAAAGAC

GAAATGCGACAAGAAGTTTCCGTGCACCCGGTGCGCTCAAAGGAATCTGAAGTGTACTCTACGTCCTACACGGCGAGCATCCAAAGGCGTCCAGAGAGTGGGAGGTC

CTCCTGAGACAGGGAGTGGTGACTCGAGCGAGAGTGGTAGCAACGCTGATAACCAGAATACATCCGGGAATGTCTCCCCAAACCACGATGGCCAACCGAAACAAGCA

ACCGCACAGTCGTCACCGTCTCAAATCCAGCAGCCTTCCCAGGTGTCCGACAGCCAACCTCCGTCACAAAGACAATCGCAATCGCCCTCAGCGCCCCACTCGCGGAA

TCCTTCCATCTCACACCCAGTACAACCACCACCACACATAAATCCCGAGGAAAAGTCGCTTCACATCCACATGCCCATCTCGAATACACCCCCGTTTTTGATCAGTCC

CCTTCTAACGGACTTGCTCATTCGTCATCGTTACTCTCACCACTTCCTACACCGGTTACGGGCATGAATAGCTTTGTCTCCTCAACACCCATGTCCGGATACGACGACT

TTGTAAGGACAGTGCGCGATCAGTCAGACAATGAGAGCCCACAATTCATCATGGATCCATGGCACTCGATGCCTATGGATACGAGTTTTGATCCCATGCGAATTGACC

CCTCGCTGATAATGACCATGGGCATGGATATGACTATGGGACCACCCCAGGACGGCATTCTCGGTATGATACCCGAAATGTCACCTGCGCAGACATTTGGACAGCCTG

TCCAGACTCCTCGCATGGTGGATGAATCTTTCTCAGACCTGCAGATTGGCAGTTCGGCTTCGATGTTCTACCCCTCCAACAGACACTCTTCAATAGCCGACGCCGGCA

TCCCCGATCTTGGCGCCATCATTGCGGCTCAAGATGGCTGGACGGTTTTCCGTTGCACTCCTTCCATCGCTTCGAGCTCATGCCCGCGAACTGCAAGGCTGAATCTTG

AGCGACTAGAGTTGAGTCTAAAGAACCACGAGGGCTGGGCTAACTGGACCCCATCATGGGACGAATCCGACTTTCAACAAGGTGGTCAAATAGCCGTGTCGAAACTAC

ATGAAGCCACACGAGACAAACTCCTCGCCATCACCCAAAGCTTCCTTCACAAGGCCCTGGACATCCATAAAGATGGAAGCAGCTCATCTAGCAGTGGCGAGTCTCCGG

GATCCACGGCCTCGCACTCTAACTTCGTTCTTCTTCCGCCTGCTCGCGTATTGGAGTACTTCCTCAAGTCGTATGCCAACTCTTTCGAAAGGTATTATCCTACCTCCGC

CAGAGGTGTTCTCGACGCCAACGAACTGATGCAAAACTACAACGACAAGGCATCAAGCTTGCTCATTCTCATGATGATTGCGCAGGGCGCCATGACTATTCCGTCCAT

AGAAGCGAGATGGCTGACTGGTGGCTTCACCGAGGCATGTCGGATATCGCTCTTCGACCTCATCGAGAAGAACATTATCATGGCTGGTGATCCTATCGTCCTGCGGG

CAGCCCTTCTCTTCACTGTCCAAGCGGCGTGGAGTGGCGACAAGTGGCAGATGGACATCGCCATGGGTCAGCGCGGCATGTATTTTTCCATGTTACGGCACTCCGGT

ATCCTAGATGTTCGGCCGCCGACAACGCCTCATATGAATGGAAACACGTCGACGGATGTGTTGTGGAAGGACTGGCTTCAGCAAGAAAGCCGCAGTCGGTAAGTGTT

GAAAACCTCGGTACAGTCACGTGTGCACACTTACTAATATTGTGCAGCCTCATCTACTCCTGGGTTATGGTAGACCAAGACCTGTCGCTCTTCCACGATACCGCCCCTC

TATTCTCGGTAACAGAGTTTGGCGCGCCAATGCCTGATTCAGATCAACTCTGGCAGGCGCAGACCGCTTCAGAGTGGTCGGAGACGTTCAACCAAGTCCATGAATTCT

CGAATGGATACTCCTCGGTAGGGTCTGGAGCAAGGCCACCGAGTCTGAGAGATCTCTTCCGTTACTTCCTAGATGACGAGATAGTCGTTCAGGATGTCCAGGAGATTC

ATCTGACCCCAATGCACCTGCGGCTGCTGCTTCATCCTTTGCAGACTCTGGTGTGCCAGTATTGCCAACTCCTCAGTTGCTTTTCCGATAGCGTAGCCTCTCGATCGCG

AAATCGGGCACTGACAGCTGCTTCGACTCGCGTACGTCTAGAAGAGGTGCAGGCGTTACTGGGGCGGTGGTTCGACTTAGCACAACGGTACATGAAGGCGAACCCAG

TGTGTCCAATGATGCAAGCCAACCTCGTCATGTTCCATCTTATCAGCATGAATGCTGTATCAAACTTCCCAGAGATTGAACGTCTCGCGCGACGAGAGGGCGTAGACG

GCTCGTACCAGCAGATGATGTGGATGCACAAGAAGTGCTTGTCGGATGTGCAAGAGGCTATCACCCACGCTGGCCAAGTACTTCGCCTCATCCGTGAGATGCCGCAA

GGCATTCGCCCGCCGTGGTGGGCTGGTGCTGTCTATCGGGCAGGGTTGGTCTTCTGGACTGATTCGCTCGCTCATGGCGAGTCCTTGAGCCCCAGCAACCACCAAG

GAACATATCAAACGTCTAATGCTGCTTTCTCTGTGGACCGCCTCCCTGCTGACCACCCAACGATACTGAGGTACATGTCACGAAGGGATGGTATCCCTACTTTGACCAA

GCGCGACGGAAATCCTGTCCCACTGGATAATGGTTTCGCTGTCCTGTCACATTGTATCGATGTACTAGACGATGGTGTCGCTACCCGATTTTCCGATGGCATTCGTAGT

AAACTCGAGAAGCTTGCGCGGGGTTAA

>*Alternaria crassa* (ACM_PT08031[b])

ATGGTCTTCTGCACATATTGTGGACAGTCGTTCACCAGGGACGAGCATCTCGAACGACATATTCTAACTCGTAAGTTGGCCCTTGCTCCGTCCTTGAGGCTTGATTTCC

GCAGTGACGAGTACAGTCCGGTCCTTCCGTGGGATCTTCGCTAACCGCCATTGCCAAACAGACACAAATGTCAAGCCCTTCAAGTGCTTTACATGCCATATGAGTTTCG

CTCGACGGTCAGTCTTACCCTCCATGCACTCCTTGCTCTTTCTCGGCCGATGGCAATCCTCGTAGGGAACGAAACGCTGATGATCCTCCCAGAGATCTTTTGCAAAGA

CATTATACCGTCCACGGCCGCAACCAGAACAACGAGGAAGGTCTGCCACCGCAAGGGATCATACCTAAATCCGCTGGTCGCACACCCATCGCCTGCAGTAATTGTGC

AAAGACGAAGACGAAATGCGACAAGAAGTTTCCCTGCACCCGGTGCGCTCAGAGGAACCTGAAATGTACTCTGCGTCCTACACGTCGAGCATCGAAAGGTGTCCAGC

GAGTGGGGGGTCCTCCTGAAACAGGGAGTGGTGATTCGAGCGAGAGTGGTAGCAATGCGGACAACCAGATCACATCCGGCAATGTCTCTCCAAACCACGACGGTCAA

TCGAAACAAGCAACCGCACAGTCATCTCCATCTCAGATTCAGCAGCCTGCCCTGATGTCTGACAGCCAACCTCCGTCACAACGACAATCGCAGTCGCCTTCAGCGCCC

CACTCGCGGAACCCTTCCATCTCGCATCCAGTGCAACCACCACCGCACATGAACCCCGAGGAGAAGTCGCTCCATATACACATGCCGATTTCAAATACTCCCCCATTC

TTCGACCAGTCACCGTCCAATGGCCTAGCTCATTCCTCGTCATTACTTTCGCCTCTTCCCACACCGGTTACGGGCATGAATAGCTTTGTCTCCTCGACACCCATGTCTG

GATACGACGATTTTGTCAGAACAGTGCGCGATCAGCCGGAGAATGAGAGTCCGCAATTCATGATGGATCCTTGGCATTCGATGCCTATGGACACGAACTTCGATCCTA

TGCGGATTGACCCCACTCTGATGATGACCATGGGCATGGATATGACCATGGGACCACCCCAAGACGGTATTCTTAGTATGATACCCGAAATGTCACCTGCGCAGACGT

TCGGACAACCTGTTCAGACTCCTCGCATGGTGGACGAATCATTCTCAGACTTGCAGATTGGCAGTTCGGCTTCGATGTTCTACCCTTCCAACAGACATTCTTCGATAGC

GGATGCCGGCATCCCTGATCTTGGAGCTATCATCGCGGCTCAAGACGGCTGGACTGTTTTCCGCTGCACTCCTTCTATTGCTTCCAGCTCATGTCCGCGGACTGCAAG

GCTGAATCTTGAGCGCCTAGAGTTGAGCCTGAAGAACCATGAGGGTTGGGCTAACTGGGCTCCGTCCTGGGATGAATCCGATTTCCAACAAGGCGGGCAAATAGCCG

TGTCGAAATTACAGGAAGCCACACGAGACAAACTCCTCGCCATCACACAGAGCTTTCTTCACAAAGCCCTAGACATCCACAAAGACGGAAACAGCACGTCCAGTAGTG

GTGAATCTCCAGGGTCCACGGCTTCGCATTCCAACTTTGTCCTTCTTCCGCCTGCTCGCGTCCTGGAGTACTTCCTCAAGTCGTACGCCAACTCTTTCGAACGCTATTA

TCCTACCTCCGCCAGAGGAGTCCTTGACGCCAACGAGTTGATGCAGAACTATAATGACAAGGCATCAAGCTTGCTCATTCTCATGATGATCGCGCAGGGCGCCATGAC

TATTCCGTCCATAGAAGCGAGATGGCTGACCGGTGGGTTTACCGAGGCATGTCGGATATCGCTGTTCGACCTGATCGAGAAGAACATTATCATGGCTGGCGATCCTAT

CGTCCTAAGAGCGGCCCTTCTCTTTACTGTCCAAGCGGCGTGGAGTGGCGACAAGTGGCAGATGGACATCGCCATGGGTCAGCGCGGCATGTATTTTTCCATGTTAC

GGCACTCCGGTATTCTTGATGTCCGACCGCCTACCACGCCTCACATGAACGGAAACACGTCGACGGATGTGTTGTGGAAGGACTGGCTCCAGCAAGAAAGCCGCAGC

CGGTAAGTGATGAAAACCTCGGTACAGTCACGTGCGGACACTTACTAATATCATGTAGCCTCATCTACTCTTGGGTCATGGTAGACCAGGACCTATCGCTGTTCCATGA

TACCGCCCCGCTGTTCTCGGTAACAGAATTCGGCGCGCCAATGCCTGATTCAGACCAGCTCTGGCAGGCGCAGACTGCTTCAGAGTGGTCGGAGATGTTCAACCAAG

TGCATGAATTCTCCAATGGGTGCTCCTCAGTAGGCTCTGGAGCAAGACCACCGAGTCTCAGAGATCTCTTCCGGTACTTCCTAGATGACGAGATAGTCGTCCAGGATG

TCCAGGAGATTCATCTGACCCCAATGCACCTGCGGCTGCTGCTCCACCCTCTGCAGACCCTTGTGTGCCAGTATTGTCAACTCCTCAGCTGCTTTTCAGATAGCGTAG

CCTCTCGATCGAGGAATCGGGCACTGACTGCCGCTTCGACTCGCGTGCGCTTAGAAGAGGTACAGGCCCTACTGGGGCGGTGGTTCGACTTGGCGCAAAGATACAT

GAAGGCGAACCCAGCATGTCCAATGATGCAGGCAAACCTCGTCATGTTCCATCTGATCAGCATGAATGCTGTGTCGAACTTCCCAGAGATCGAGCGCCTCGCGCGAC

GAGAGGGCGTGGACGGCTCGTACCAGCAGATGTTGAGGATGCACAAGAAGTGCTTGTCGGATGTGCAGGAGGCCATAACCCATGCTGGTCAAGTTCTTCGCCTCATT

CGTGAGATGCCACGAGGCATCCGCCCGCCGTGGTGGGCTGGTGCTGTCTATCGGGCAGGGTTGGTGTTTTGGACTGATTCACTCGCCCGTAACGAGTCTCTGAGCC

CCAATCACCAAGGAGCATATCAGCAGCCATCTAATACTGCTTTCTCTGTGGATCGCCTTTCTGCTGACCACCCTACCATACTGAGGTACATGTCACGAAGGGAGGGCAT

CCCCACCTTGACCAAGCGTGACGGAAACCCCGTTCCACTGGATAACGGCTTCGCTATCCTGTCGCATTGTATCGATGTCCTTGACGAGGGCGTCGCCACCAGATTTTC

CGATGGCATTCGTAGTAAACTCGAGAAACTTGCGCGTGGTTGA

>*Alternaria dauci* (ADC_PT01443[c])

ATGGTCTTCTGCACATATTGTGGACAGTCGTTCACCAGGGACGAGCATCTCGAACGACATATCCTAACTCGTAAGTTGGCTCTTGCTCCGTCCTTGAGGGTTGATTTCC

GCAGTGACGAGCACAGTCCGGTCGTTCCGTGGGATCTTCGCTAACCGCCATTGCCAAACAGACACAAATGTCAAGCCCTTCAAGTGCTTTACATGCCATATGAGTTTC

GCTCGACGGTCAGTCTTACCCATCGTGTGCTCCTTGCTCTTTCTCGGCCGGTGGCAACCCTTGTAGCGAACGAAACGCTGACGATCCTCCCAGAGATCTTTTGCAAAG

ACATTATACCGTCCACGGCCGCAACCAGAACAACGAGGAAGGTCTGCCACCACAAGGGATCATACCCAAATCTGCTGGTCGCACACCCATCGCCTGCAGTAATTGTGC

AAAGACGAAGACGAAATGCGACAAGAAGTTTCCTTGCACCCGGTGCGCTCAGAGGAACCTGAAATGTACTCTGCGTCCTACACGTCGAGCATCGAAAGGTGTCCAGC

GAGTGGGGGGTCCTCCTGAAACAGGGAGTGGTGATTCGAGCGAGAGTGGTAGCAATGCGGACAACCAGATCACATCCGGCAATGTCTCTCCAAACCACGACGGCCA

ATCGAAACAAGCAACCGCACAGTCATCTCCATCTCAGATTCAGCAGCCTGCCCTGATGACTGACAGCCAACCTCCGTCACAACGACAATCGCAGTCGCCTTCAGCGCC

CCACTCGCGGAATCCTTCCATCTCGCATCCAGTGCAACCACCACCGCACATGAACCCCGAGGAGAAGTCGCTCCATATACACATGCCGATTTCAAATACTCCCCCATT

CTTCGACCAGTCACCGTCCAATGGCCTAGCTCATTCCTCGTCATTACTTTCGCCTCTTCCCACACCGGTTACGGGCATGAATAGCTTTGTCTCCTCGACACCCATGTCT

GGATACGACGATTTTGTCAGGACAGTGCGCGATCAGCCGGAGAATGAGAGTCCGCAATTCATGATGGATCCTTGGCATTCGATGCCTATGGACACGAACTTCGATCCT

ATGCGGATTGACCCCGCTCTGATGATGACCATGGGCATGGATATGACTATGGGACCACCCCAAGACGGCATTCTTAGTATGATACCAGAGATGCCACCTGCGCAGACA

TTCGGACAGCCTGTCCAGACTCCTCGCATGGTGGACGAATCATTCTCGGACTTGCAGATTGGCAGCTCGGCTTCGATGTTCTACCCTTCCAACAGACATTCTTCGATAG

CGGACGCCGGCATCCCTGATCTTGGAGCCATTATCGCGGCTCAAGACGGCTGGACTGTTTTCCGTTGCACTCCTTCTATTGCTTCCAGCTCATGTCCGCGGACTGCAA

GGCTGAATCTTGAGCGACTAGAGTTGAGCCTGAAGAACCATGAGGGTTGGGCCAACTGGGCTCCATCCTGGGATGAATCCGATTTCCAACAAGGCGGGCAAATAGCC

GTGTCGAAATTACAGGAAGCCACACGAGACAAACTCCTCGCCATCACACAGAGCTTTCTTCACAAAGCTCTAGACATCCACAAAGACGGAAGCAGCACGTCCAGTAGT

GGTGAATCTCCAGGGTCCACGGCTTCGCATTCCAACTTCGTCCTTCTTCCGCCTGCTCGCGTCCTGGAGTACTTCCTCAAGTCGTATGCCAACTCTTTCGAACGCTATT

ATCCTACCTCCGCCAGAGGAGTCCTTGACGCCAACGAGTTGATGCAGAACTACAATGACAAGGCATCAAGCTTGCTCATTCTCATGATGATTGCGCAGGGCGCCATGA

CCATTCCGTCCATAGAAGCGAGATGGCTGACCGGTGGATTTACCGAGGCATGTCGGATATCGCTGTTCGACCTGATCGAGAAGAATATCATCATGGCTGGCGATCCTA

TCGTCCTGAGAGCGGCCCTTCTCTTCACTGTCCAAGCGGCGTGGAGCGGCGACAAGTGGCAGATGGACATCGCCATGGGTCAGCGCGGCATGTATTTTTCCATGTTA

CGGCACTCGGGTATTCTTGATGTTCGGCCACCTACCACGCCTCACATGAACGGAAACACGTCGACGGATGTGTTGTGGAAGGACTGGCTTCAGCAAGAAAGCCGCAG

CCGGTAAGTGTTGAAAACCTCGGTACAGTCACGTGCGCACACTTACTAATATGATGTAGCCTCATCTACTCTTGGGTCATGGTAGACCAGGACCTATCGCTCTTCCATG

ATACCGTCCCGCTGTTCTCGGTAACAGAATTCGGCGCGCCAATGCCTGATTCAGATCAGCTCTGGCAGGCGCAGACCGCTTCAGAGTGGTCGGAGATGTTCAACCAA

GTGCATGAATTCTCCAATGGGTACTCCTCAGTAGGCTCTGGAGCAAGACCACCGAGTCTCAGAGATCTCTTCCGTTACTTCCTAGATGACGAGATAGTCGTTCAGGATG

TCCAGGAGATTCATCTGACCCCGATGCACCTGCGGCTGCTGCTCCACCCTCTGCAGACCCTTGTGTGCCAGTATTGTCAACTCCTCAGCTGCTTTTCAGATAGCGTAG

CCTCTCGATCGAGAAATCGGGCACTGACTGCCGCTTCGACTCGGGTGCGCCTAGAAGAGGTACAGGCCTTACTGGGGCGGTGGTTCGACTTGGCGCAAAGATACATG

AAGGCGAACCCAGTATGTCCGATGATGCAGGCAAACCTCGTCATGTTCCATCTGATCAGCATGAATGCTGTGTCGAACTTCCCAGAGATCGAGCGCCTCGCGCGACG

AGAAGGCGTGGACGGCTCGTACCAGCAGATGTTGTGGATGCACAAGAAGTGCTTGTCAGATGTGCAGGAGGCCATAACCCATGCTGGTCAAGTTCTTCGCCTCATCC

GTGAGATGCCACGAGGCATCCGCCCGCCGTGGTGGGCTGGTGCTGTCTATCGGGCAGGGTTGGTGTTCTGGACTGATTCGCTCGCCCATAACGAGTCTCTGAGCCC

CAACCACCAAGGAACATATCAGCAGCCATCTAATACTGCTTTCTCTGTGGATCGCCTTTCTGCTGACCACCCTACCATACTGAGGTACATGTCACGAAGGGAGGGTATC

CCCACCTTGACCAAGCGTGACGGAAACCCCGTTCCACTGGATAACGGCTTCGCTATCCTGTCGCATTGTATCGACGTCCTTGACGAGGGCGTCGCCACCAGATTTTCC

GATGGCATTCGTAGTAAACTCGAGAAACTTGCGCGTGGTTGA

>*Alternaria destruens* (ADT_PT05662[b])

ATGGTCTTCTGCACATATTGTGGACAGTCGTTCACCAGGGACGAACATCTCGAACGACATATCCTAACTCGTAAGTCAACTCTTGCTCTGTCCATGAGGGTGGATTTCC

GCAATGTCGAGTTCAATCCGATCGTTCCGTAGCATCTCCGCTGACCGTCAATGCCAAACAGACACCAATGTCAAGCCATTCAAGTGCTTTACATGTCATATGAGCTTTG

CTCGACGGTAAGTCCCACCCTGCGTGCGCTCCTTCCTCTTCCTGTTTGGTCATAATTTCCGTACCGAACGAAACGCTGACAATCTTTCCAGAGATCTTTTGCAAAGACA

TTACACCGTACACGGCCGCAACCAGAACAACGAAGAAGGCCTCCCACCGCAAGGCATCATACCTAAGTCCGCCGGCCGCACACCCATCGCCTGCAGTAATTGTGCAA

AGACGAAGACGAAATGCGACAAGAAGTTTCCTTGCACCCGGTGCGCTCAAAGGAACCTGAAGTGTACTCTACGTCCTACACGGCGAGCATCGAAAGGTGTCCAACGA

GTAGGAGGTCCTCCTGAAACAGGCAGTGGTGATTCNNNNNNNNNNNNNNNNNNNAGCGAGAGTGGTAGCAACGCGGATAACCAGAACACATCCGGCAATGTCTCTCCA

AACCACGATGGCCAGTCGAAACAAGCAACCGCTCAGTCGTCTCCATCTCAAATTCAGCAGCCTAGCCAGATTTCTGACAGCCAACCTCCATCGCAACGGCAATCGCAA

TCTCCCTCAGCGCCCCACTCGCGGAATCCTTCCATCTCACATCCAGTGCAACCACCACCGCACATGAACCCCGAGGAGAAGTCGCTCCACATACACATGCCTATCTCG

AATACTCCCCCGTTCTTCGATCAGTCACCCTCTAACGGACTGTCTCATTCCTCATCATTACTTTCACCTCTTCCCACACCAGTTACGGGCATGAATAGTTTTGTTTCTTCG

ACACCCATGTCTGGGTACGACGACTTCGTCAGGACAGTGCGCGATCAACCGGAAAATGAGAGCCCGCAATTCATGATGGATCCTTGGCACTCGATGCCCATGGATAC

GAGTTACGATCCTATGCGGATTGACCCCGCTCTGATGATGACCATGGGCATGGACATGACGATGGGACCGCCCCAAGACGGTATCCTTAGTATGATACCCGAAATGTC

ACCTGCGCAAACATTCGGACAGCCTGTTCAGACTCCTCGTATGGTGGACGAATCGTTCTCGGACTTGCAAATCGGCAGTTCGGCTTCGATGTTCTACCCCTCTAACAG

ACATTCCTCAATAGCGGACGCCGGCATCCCTGATCTTGGCGCCATTATCGCAGCCCAAGACGGCTGGACGGTCTTCCGTTGCACTCCTTCTATCGCTTCGAGCTCATG

TCCGCGGACCGCAAGGCTGAATCTTGAGAGATTAGAGTTGAGCCTGAAGAACCATGAGGGCTGGGCTAACTGGACCCCATCGTGGGACGAATCTGATTTCCAACAAG

GCGGACAGATCGCCGTGTCAAAACTCCAGGAAGCCACACGAGACAAACTTCTCGCGATCACACAGAGCTTCCTTCACAAGGCTCTAGATATCCACAAAGATGGGAGCA

GCTCGTCTAGTAGTGGCGAGTCTCCAGGATCTACGGCTTCACATTCCAACTTCGTTCTTCTTCCGCCTGCTCGTGTCCTGGAGTACTTCCTCAAGTCGTACGCCAACTC

TTTCGAACGCTATTATCCTACCTCGGCCAGAGGAGTCCTCGACACCAACGAGTTGATGCAGAACTACAATGACAAAGCTTCGAGCTTGCTCATTCTCATGATGATTGCG

CAGGGCGCTATGACCATTCCGTCCATAGAAGCGAGATGGCTGACTGGCGGCTTTACTGAGGCATGTCGGATATCCCTGTTCGACCTCATCGAGAAGAACATCATNNNN

NNNNNNNNNNNNNNNNNNNNNNNNNGGCTGGCGATCCTATTGTCCTGAGGGCGGCCCTTCTCTTCACTGTTCAAGCGGCGTGGAGTGGCGACAAGTGGCAGATGGA

CATCGCCATGGGTCAGCGCGGCATGTATTTTTCCATGTTACGACATTCCGGTATTCTCGATGTTCGACCGCCTACCACGCCTCACATGAATGGAAACACTTCGACGGAT

GTGTTGTGGAAGGACTGGCTTCAGCAAGAAAGCCGCAGCCGGTAAGTGTTGAAAACCTCGGTACTGTCACGTGCGCGCACTTACTAATATCGTGTAGCCTCATCTACT

CTTGGGTCATGGTAGACCAAGACCTATCGCTATTCCACGATACCGCTCCCCTGTTTTCGGTAACAGAATTTGGCGCGCCAATGCCTGATTCAGATCAACTCTGGCAGG

CGCAGACAGCTCCAGAGTGGTCGGAGATGTTCAACCAGGTCCATGAATTCTCGAATGGATACTCCTCAGTAGGCTCTGGAGCAAGACCACCGAGTCTCAGAGATCTCT

TCCGCTACTTCCTAGATGACGAGATAGTCGTCCAGGATGTTCAGGAGATTCATCTGACCCCGATGCACCTGCGACTACTACTGCACCCTCTGCAGACCCTTGTGTGCC

AATATTGCCAACTCCTCAGTTGCTTCTCCGACAGTGTAGCCTCTCGATCGCGGAATCGGGCATTGACAGCTGCTTCGACTCGCGTACGCCTAGAAGAGGTACAGGCGT

TATTGGGGCGGTGGTTCGACTTGGCGCAACGGTACATGAAGGCGAACCCAGTATGTCCGATGATGCAAGCAAATCTCGTCATGTTCCATCTTATCAGCATGAATGCTG

TGTCAAATTTCCCAGAGATTGAGCGCCTCGCCCGACGGGAGGGTGTGGACGGCTCGTATCAACAGATGCTGTGGATGCACAAGAAGTGCTTGTCGGATGTGCAGGAG

GCCATCACTCACGCTGGCCAAGTTCTNNNNNNNNNNNNNNNNNNNNNNNNNNNNNNNNNNNNNNNNNNNNNNNNNNAGGCATTCGCCCGCCGTGGTGGGCTGG

TGCTGTCTATCGGGCAGGGTTGGTCTTCTGGGCTGACTCGCTCGCTCACAACGAGTCCTTGAGTCCCAACCATCAAGGGACATATCAGCCATCTAATACTGCTTTCTCT

GTGGATCGCCTCTCTGCTGACCACCCAACCATACTGAGGTACATGTCACGGAGGGAGGGTATTCCCACCTTGACCAAGCGCGATGGTAGCCCTGTTCCACTGGATAA

CGGCTTTGCCGTCCTGTCGCATTGTATCGATGTCCTTGACGAGGGTGTCGCCACCAGGTTTTCCGATGGCATTCGTAGTAAACTCGAGAAACTTGCGCGCGGTTAA

>*Alternaria ethzedia* (NA)

ATGGTCTTTTGCACATACTGTGGACAGTCGTTCACCAGAGACGAGCATCTCGAACGACATATCTTGACTCGTATGTTGACCCCGCGTCTTTCGCTCTTTCAATCTTCCTC

GCACCAGCTACCATCCAATCTTTCTTGTGGGATATCTGCTAATCACTGTGGATGAGCAGACACCAACGTCAAGCCTTTCAAATGCTTCACATGTCATATGAGCTTTGCTC

GACGGTATGTGGCACCCTTCCTTGCTCTTTCTCATCCTGTGGCATCGACCGTTCGGAACAAAGTACTGACAGCCTCTTCAGAGATCTTTTGCAAAGACACTACACCGTA

CACGGCCGCAACCAGAACAACGAAGAGGGACTCCCCCACAAGGCATAATACCAAAGTCCGCCGGCCGCACACCCATCGCCTGCAGTAATTGTGCGAAGACAAAGAC

CAAGTGTGACAAGAAGTTTCCGTGCACCAGGTGCGCTCAGAGGAATCTGAAATGCACATTGCGTCCTACAAGGCGGGCATCGAAAGGTGTCCAGCGGGTTGGCGGTC

CTCCTGAGGCTGGGAGCGGGGACTCGAGCGAGAGTGGTAGCAACGCGGATAACCACAATACATCCGGCAATGTCTCTCCAAACCACGATAGCCAACCGAAACAAGC

GATGTCGAAGTCCTCTCCTGCTCAGATCAACCAGACTTCCCATATTCCGGACAGCCAACCACCGTCGCAAAGACAATCGCAGTCGCCCTCGGCCCCTCATTCACGAAA

CCCTTCCATCTCACAACCGGTACAGCCACCGACACACATAAATCCCGAGGAGAAGTCGTTGCATCTTCATATGCCGATCTCCGATACGCCACCCTTTTTCGACCAGTCC

CCTTCCAACGGACTTGCTCAATCATCGTCGCTTCTTTCGCCACTCCCCACCCCGGTGAACGGCATGAATGGATTCGTCACCTCCACACCTATGTCAGGATATGACGACT

TCGTCAGAACGGTACGGGACCAATCCGATAATGAGAGCCCTCAATTCATGATGGACCCTTGGAACGGCATGCCCATGGATACAACCTTTGATCCCATGCGGATAGACC

CCGCGTTGATGATGAGCATGAGTATGGACATGAGCATGGGACCACCTCAGGATGGAATCCTTGGCATGATACCAGAAATGTCGCCTGCTCAAACATTTGGACAGCCTG

TCCAGACACCTCGCATGGACGAGTCCTTTTCGGACCTGCAAATTGGTAGTTCGGCTTCGATGTTCTACCCATCCAACAGGCACTCTTCGATAGCAGATGCCGGCATAC

CTGATCTTGGTGCCATCATCGCGGCTCAAGATGGCTGGACTGTTTTCCGTTGCACTCCTTCTATTGCTTCAAGCTCGTGTCCACGCACAGCGAGACTCAACCTCGAGC

GGTTAGAGCTGAGCCTCAAGAATCACGAGGGCTGGGCTAACTGGTCCCCATCGTGGGATGAATCCGACTTTCAACAAGGCGGGCAAATCGCGGTATCGAAGTTGCAG

GAAGCCACGCGAGACAAACTTCTCGCCATCACCCAAAGCTTCCTCCACAAGGCTTTGGACATCCACAAAGACGGAAGCAGCTCGTCCAGCAGTGGCGAGTCTCCAGG

ATCCACGGCCTCTCACTCCAACTTCGTTCTTCTTCCACCTGCTCGCGTCCTGGAGTACTTTCTCAAGTCATACGCCAACTCTTTCGAACGCTATTATCCCACCTCCGCCA

GAGGTGTGCTCGATACGAATGAACTAATGCAAAACTACAACGACAAAGCATCGAGCTTGCTCATTCTCATGATGATCGCGCAGGGCGCCATGACCATTCCATCCATAG

AGGCCAGGTGGCTAACTGGTGGCTTCACCGAAGCCTGCCGAATATCGCTCTTCGACCTTATTGAGAAGAACATCATCATGGCTGGTGATCCTATCGTTCTGCGTGCAG

CCCTCCTCTTCACTGTCCAGGCGGCGTGGAGTGGCGACAAATGGCAGATGGACATCGCTATGGGCCAGCGTGGCATGTATTTCTCCATGTTGCGACATTCCGGTATTC

TCGATGTGCGACCGCCGGCAACGCCTCATATCAACGGAAATATTTCGACGGATGCGTTGTGGAAGGACTGGCTTCAGCAAGAAAGCCGCAGTCGGTAAGTGTTGAAA

ACTTCGGTACCGTCACGTGAACGCACTTACTAATATTGGCGCAGCCTCATCTACTCCTGGGTCATGGTAGACCAAGACTTATCGCTCTTCCACGATACCGCTCCACTGT

TCTCAGTAACAGAGTTTGGCGCGCCGATGCCTGATTCGGATCGACTTTGGCAAGCGCAGACCGCCTCAGAGTGGTCTGAGATGTTCAACCAAGTCCATGAGTTCTCGA

ACGGGTACTCTTCAGTAGGCTCTGGGGCCAGACCACCCAGTCTCAGAGACCTGTTCCGTTACTTCCTAGATGACGAAATCGTCGTACAGGATGTACAGGAGATTCATC

TGACACCTATGCACCTGCGTCTGCTCCTCCACCCCCTGCAGACTCTGGTCTGCCAGTATTGCCAGCTTCTCAGCTGCTTTTCCGATAGCGTCGCTTCACGATCGCGGA

ATCGCGCACTGACAGCAGCGTCGACCCGCGTGCGTCTCGAGGAGGTGCAGGCGTTGCTAGGGCGTTGGTTCGATTTGGCGCAACGGTACATGAAGTCCAACCCAGT

GTGTCCGATGATGCAGGCGAACCTTGTCATGTTCCATCTTATCAGCTTGAACGCTGTGTCCAACTTTCCAGAGATAGAGCGCCTTGCGCGTCGAGAGGGTGTGGACGG

CTCATACCAGCAGATGCTGTGGATGCACAAGAAGTGTCTGTCCGATGTGCAGGAGGCCATCACCCACGCCGGTCAGGTCCTTCGACTCATCCGTGAGATGCCAAGAG

GTATCCGGCCGCCATGGTGGGCTGGCGCCGTCTACCGTGCGGGTTTGGTCTTCTGGACTGATTCGCTGGCACATGGCGAGTCCTTGAGTTCCAACCACCAAGGATCG

TATCAGCCATCGAACTCCGCGTTCTCTGTGGATCGCCTTCCAGCTGACCACCCTACCATACTGAGGTACATGTCTCGTAGGGAGGGCATACCCACTCTGACCAAGCGC

GACGGTACACACGTCCCCTTGGATAACGGCTTCGCGATCCTGTCGCATTGTATCGACGTCCTTGATGAGGGTGTTGCCACCAGGTTCTCCGATGGCATTCGTAGTAAA

CTCGAGAAACTCGCGCGAGGATAA

>*Alternaria fragariae* (AFG_PT01606[c])

ATGGTCTTCTGCACATATTGTGGACAGTCGTTCACCAGGGACGAACATCTCGAACGACATATCCTAACTCGTAAGTCAACTCTTGCTCTGTCCATGAGGGTGGATTTCC

GCGATGTCGAGTTCAATCCGATCGTTCCGTAGCATCTCCGCTGACTGTCAATGCCAAACAGACACCAATGTCAAGCCATTCAAGTGCTTTACATGTCATATGAGCTTTG

CTCGACGGTAAGTCCCACCCTGCGTGCGCTCCTTCCTCCTCTTGTTTGGTCATAATTTCGGTACCGAACGAAACGCTGACAATCTTTCCAGAGATCTTTTGCAAAGACA

TTACACCGTACACGGCCGCAACCAGAACAACGAAGAAGGCCTCCCACCGCAAGGCATCATACCTAAGTCCGCCGGCCGCACACCCATCGCCTGCAGTAATTGTGCAA

AGACGAAGACGAAATGCGACAAGAAGTTTCCTTGCACCCGGTGCGCTCAAAGGAATCTGAAGTGTACTCTACGTCCTACACGGCGAGCATCGAAAGGTGTCCAACGA

GTAGGAGGTCCTCCTGAAACAGGCAGTGGTGATTCGAGTGAGAGTGGTAGCAACGCGGATAACCAGAACACATCCGGCAATGTCTCTCCAAACCACGATGGCCAGCC

GAAACAAGCAACCGCTCAGTCGTCTCCATCTCAAATTCAGCAGCCTAGCCAGATTTCCGACAGCCAACCTCCATCGCAACGGCAATCGCAATCTCCCTCAGCGCCCCA

CTCGCGGAATCCTTCCATCTCACATCCAGTGCAACCACCACCGCACATGAACCCCGAGGAGAAGTCGCTCCACATACACATGCCTATCTCGAATACTCCCCCGTTCTT

CGATCAGTCACCCTCTAATGGACTGTCTCATTCCTCATCATTACTTTCACCTCTTCCCACACCAGTTACGGGCATGAATAGTTTTGTTTCTTCGACACCCATGTCTGGGT

ACGACGACTTCGTCAGGACAGTGCGCGATCAACCGGAAAATGAGAGCCCGCAATTCATGATGGATCCTTGGCACTCGATGCCCATGGATACCAGTTACGATCCTATGC

GGATTGACCCCGCTCTGATGATGACCATGGGCATGGACATGACGATGGGACCGCCCCAAGACGGTATCCTTAGTATGATACCCGAAATGTCACCTGCGCAAACATTCG

GACAGCCTGTTCAGACTCCTCGTATGGTGGACGAATCGTTCTCGGACTTGCAAATCGGCAGTTCGGCTTCGATGTTCTACCCCTCTAACAGACATTCCTCAATAGCGGA

CGCCGGCATCCCTGATCTTGGCGCCATTATCGCAGCCCAAGACGGCTGGACGGTCTTCCGTTGCACTCCTTCTATCGCTTCGAGCTCATGTCCGCGGACTGCAAGAC

TGAATCTTGAGAGATTAGAGTTGAGCCTGAAGAACCATGAGGGCTGGGCTAACTGGACCCCATCGTGGGACGAATCTGATTTCCAACAAGGCGGACAGATCGCCGTG

TCAAAACTCCAAGAAGCCACGCGAGACAAACTCCTCGCGATCACACAGAGCTTCCTTCACAAGGCTCTAGATATCCACAAAGACGGAAGCAGCTCATCTAGTAGTGGC

GAGTCTCCAGGATCTACGGCTTCACATTCCAACTTCGTTCTTCTTCCGCCTGCTCGTGTCCTGGAGTACTTCCTCAAGTCGTACGCCAACTCTTTCGAACGCTATTATCC

TACCTCGGCCAGAGGAGTCCTCGACACCAACGAGTTGATGCAGAACTACAATGACAAAGCTTCGAGCTTGCTCATTCTCATGATGATTGCGCAGGGCGCCATGACCAT

TCCGTCCATAGAAGCGAGATGGCTGACTGGTGGCTTTACTGAGGCATGTCGGATATCGCTGTTCGACCTCATCGAGAAGAACATCATTATGGCTGGCGATCCTATTGT

CCTGAGGGCGGCCCTTCTCTTCACTGTTCAAGCGGCGTGGAGTGGCGACAAGTGGCAGATGGACATCGCCATGGGTCAGCGCGGCATGTATTTTTCCATGTTACGAC

ATTCCGGTATTCTCGATGTTCGACCGCCTACCACGCCTCACATGAATGGAAACACTTCGACGGATGTGTTGTGGAAGGACTGGCTTCAGCAAGAAAGCCGCAGCCGGT

AAGTGTTGAAAACCTCGGTACAGTCACGTGCGCGCACTTACTAATATCGTGTAGCCTCATCTACTCTTGGGTCATGGTAGACCAAGACCTATCGCTATTCCACGACACT

GCTCCTCTGTTTTCGGTAACAGAATTTGGCGCGCCAATGCCTGATTCAGATCAACTCTGGCAGGCGCAGACAGCTCCAGAGTGGTCGGAGATGTTCAACCAGGTCCAT

GAATTCTCGAATGGATACTCCTCAGTAGGCTCTGGAGCAAGACCACCGAGTCTCAGGGATCTCTTCCGCTACTTCTTAGATGACGAGATAGTCGTCCAGGATGTTCAG

GAGATTCATCTGACCCCGATGCACCTGCGACTACTACTGCATCCTCTGCAGACCCTTGTGTGCCAATATTGCCAACTCCTCAGTTGCTTTTCCGACAGTGTAGCCTCTC

GATCACGGAATCGGGCATTGACAGCTGCTTCGACTCGCGTACGCCTAGAAGAGGTGCAGGCGTTATTGGGGCGGTGGTTCGACTTGGCGCAACGGTACATGAAGGC

GAACCCAGTATGTCCGATGATGCAAGCAAATCTCGTCATGTTCCATCTTATCAGCATGAATGCTGTGTCAAATTTCCCAGAGATTGAGCGCCTCGCCCGACGGGAGGG

TGTGGACGGCTCGTATCAACAGATGCTGTGGATGCACAAGAAGTGCTTGTCGGATGTGCAGGAGGCCATCACTCACGCTGGCCAAGTTCTTCGCCTCATTCGTGAGAT

GCCACGAGGCATTCGCCCGCCGTGGTGGGCTGGTGCTGTCTATCGGGCAGGGTTGGTCTTCTGGGCTGACTCGCTCGCTCACAACGAGTCCTTGAGTCCCAACCAT

CAAGGGACGTATCAGCCATCTAATACTGCTTTCTCTGTGGATCGCCTCTCTGCTGATCACCCAACCATACTGAGGTACATGTCACGGAGGGAGGGTATTCCCACCTTG

ACCAAGCGCGATGGTAGCCCTGTTCCACTGGATAACGGCTTTGCCGTCCTGTCGCATTGTATCGATGTCCTTGACGAGGGTGTCGCCACCAGGTTTTCCGATGGCATT

CGTAGTAAACTCGAGAAACTTGCGCGCGGTTAG

>*Alternaria gansuensis* (NA)

ATGGTCTTCTGCACATATTGTGGACAGTCGTTCACCCGAGACGAACATCTCGAACGACATATCCTAACCCGTAAGTTGATCCCCTTCGTCTGTCCTTGCGAACTGGTTT

TCGCAGTGCCAACTACGAACCCATCGTTCTGTGGGATTCTCCGCTGACCACTCTTACCGAACAGACACCAACGTCAAGCCTTTCAAGTGCTTTACATGTCATATGAGCT

TTGCTCGCCGGTATGTTCCATCTTTCGTGCGCTTCCTTGCTCTTCTTCGTCTTGTGCTACCTGTCATATGAAATGAAGCGCTGACAACCCCCTCTAGAGATCTCTTGCAA

AGACATTATACCGTACACGGCCGCAACCAGAACAACGAAGAGGGTCTCCCACCGCAAGGGATAATACCCAAGTCCGCCGGTCGCACACCCATCGCTTGCAGTAATTG

TGCAAAGACAAAGACGAAATGCGACAAGAAGTTTCCGTGCACTCGGTGCGCTCAACGGAATCTGAAGTGTACTCTACGTCCTACACGGCGAGCATCGAAAGGTGTCCA

GCGAGTGGGAGGTCCTCCTGAAACGGGGAGTGGTGACTCGAGCGAGAGTGGCAGTAACGCGGATAACCAGAACACATCTGGGAATGTCTCTCCAAACCACGATGGC

CAACCAAAACAAGCGACAGCACAGTCGTCTCCATCTCAACTCCAGCAGCCTGGCCAGGTTTCTGACAGCCAGCCTTCGTCACAACGACAATCGCAATCGCCCTCAGCG

CCCCACTCGCGGAACACCTCCATCTCACAGCCAGTACAACCACCACCACATATAAATCCTGAGGAAAAGTCGCTCCACATTCATATGCCAATCTCGAATACACCCCCAT

TTTTCGATCAGTCCCCTTCTAACGGACTTGCTCAATCGTCATCACTACTCTCTCCTCTTCCCACACCGGTTACTGGCATGAATAGTTTCGTCTCTTCAACACCCATGTCC

GGATACGATGACTTTGTAAGGACAGTGCGGGACCAGTCCGACAACGAGAGCCCACAATTCATGATGGATCCTTGGCACTCGATGCCTATGGATACGAGCTTCGACTCC

ATGCGGATAGACCCCGCGCTGATGATGAGTATGGGCATGGATATGACAATGGGACCACCCCAAGACGGCATCCTTAGTATGATACCCGAAATGTCACCTGCGCAAAC

GTTCGGACAGCCTGTCCAGACACCTCGCATGGACGAATCGTTCTCAGACCTGCAGATTGGCAGTTCGGCTGCGATGTTCTACCCCTCTAACAGACACTCTTCGATAGC

GGACGCCGGTATTCCTGATCTTGGTGCCATCATCGCGGCCCAAGATGGCTGGACAGTTTTCCGATGCACTCCTTCTATCGCTTCGAGTTCTTGTCCGCGAACTGCAAG

GTTGAATCTTGAGCGGTTAGAGTTGAGCCTGAAGAACCACGAGGGCTGGGCTAACTGGACCCCATCGTGGGACGAATCCGACTTTCAACAAGGCGGGCAAATAGCTG

TTTCGAAGTTACAAGAAGCCACACGAGACAAACTTCTCGCCATCACCCAAAGCTTCCTTCACAAGGCCCTGGACATTCACAAGGACGGAAGTAGCTCCTCGAGCAGTG

GTGAGTCTCCAGGATCCACGGCTTCTCACTCCAACTTCGTTCTTCTCCCGCCTGCTCGCGTTCTGGAGTACTTTCTCAAGTCGTACACCAACTCTTTCGAACGGTATTA

TCCCACCTCCGCCAGAGGTGTTCTCGACGCCAACGAGTTGATGCAAAACTACAATGACAAGGCATCGAGCTTGCTCATCCTCATGATGATTGCACAGGGCGCAATGAC

CATTCCGTCCATAGAAGCGAGATGGCTGACTGGTGGGTTCACCGAAGCATGTCGGATATCGCTCTTCGACCTCATCGAGAAGAACATAATCTTGGCTGGTGATCCCAT

CGTCCTGCGTGCAGCCCTTCTCTTCACCGTCCAAGCGGCGTGGAGTGGTGACAAGTGGCAGATGGACATCGCCATGGGTCAGCGCGGTATGTATTTCTCCATGTTAC

GACACTCCGGTATTCTTGATGTCCGGCCGCCGACAACACCTCACATGAACGGAACCACGTCGACGGATGCGTTGTGGAAGGACTGGCTTCAGCAAGAAAGCCGTAGT

CGGTAAGTGCTGAAAACCTCGGTACAGTCACGTGCGCACGCTTACTAATATCGCCGTAGCCTTGTCTACTCTTGGGTCATGGTAGACCAGGACCTGTCACTGTTCCAC

GATACCGCGCCTCTGTTCTCGGTAACGGAATTCAGCGCGCCAATGCCTGATTCAGATCAACTCTGGCAGGCGCAGACCGCTTCAGAGTGGTCGGAGATGTTCAACCA

AGTCCATGAGTTCTCGAATGGGTACTCTTCAGTAGGCTCTGGAGCAAGGCCGCCGAGTCTCAGAGATCTCTTCCGTTACTTCCTAGATGACGAGATAGTTGTTCAGGAT

GTTCAGGAGATCCACCTGACGCCCATGCACCTGCGGCTGCTGCTTCATCCTTTGCAGACTCTTGTGTGCCAATATTGCCAACTCCTTAGCTGCTTTTCAGATAGCGTGG

CCTCTCGATCGCGAAATCGGGCACTGACAGCCGCTTCGACTCGCGTGCGCCTGGAAGAGGTGCAGGCGTTACTGGGGCGGTGGTTCGACTTGGCGCAACGGTACAC

GAAGGCAAACCCAGCGTGTCCAATGATGCAAGCAAACCTCGTCATGTTCCATCTCATCAGCATGAACGCTGTGTCGAATTTCCCAGAAATCGAGCGCCTCGCGCGACG

AGAGGGCGTCGACGGCTCGTACCAGCAGATGTTGTGGATGCACAAGAAGTGCCTGTCGGATGTGCAGGAGGCTGTCACCCACGCTGGTCAAGTCATTCGCCTCCTCC

GTGAGATGCCACGAGGCATTCGCCCACCGTGGTGGGCTGGTGCTGTTTACCGGGTAGGGTTGGTTTTCTGGACTGATTCGCTCGCTCATAACGAGTCCCTGAGCCCC

AACCACCAGGGGACATATCAGCCCTCTAATGCCGCTTTTTCTGTAGATCGCCTTCCTGCTGACCACCCAACTATACTGAGATACATGTCACGAAGGGAGGGTATCCCC

ACCTTGACCAAGCGCGACGGTACACCCGTCCCACTGGACAACGGCTTCGCTGTCCTGTCGCATTGTATCGACGTCCTTGACGAGGGTGTCGCTACCAGATTTTCTGAT

GGCATTCGTAGCAAACTCGAGAAACTTGCGCGGGGTTAA

>*Alternaira gaisen* (AGS_PT02526[c])

ATGGTCTTCTGCACATATTGTGGACAGTCGTTCACCAGGGACGAACATCTCGAACGACATATCCTAACTCGTAAGTCAACTCTTGCTCTGTCCATGAGGGTGGATTTCC

GCGATGTCGAGTTCAATCCGATCGTTCCGTAGCATCTCCGCTGACTGTCAATGCCAAACAGACACCAATGTCAAGCCATTCAAGTGCTTTACATGTCATATGAGCTTTG

CTCGACGGTAAGTCCCACCCTGCGTGCGCTCCTTCCTCCTCTTGTTTGGTCATAATTTCGGTACCGAACGAAACGCTGACAATCTTTCCAGAGATCTTTTGCAAAGACA

TTACACCGTACACGGCCGCAACCAGAACAACGAAGAAGGCCTCCCACCGCAAGGCATCATACCTAAGTCCGCCGGCCGCACACCCATCGCCTGCAGTAATTGTGCAA

AGACGAAGACGAAATGCGACAAGAAGTTTCCTTGCACCCGGTGCGCTCAAAGGAATCTGAAGTGTACTCTACGTCCTACACGGCGAGCATCGAAAGGTGTCCAACGA

GTAGGAGGTCCTCCTGAAACAGGCAGTGGTGATTCGAGTGAGAGTGGTAGCAACGCGGATAACCAGAACACATCCGGCAATGTCTCTCCAAACCACGATGGCCAGCC

GAAACAAGCAACCGCTCAGTCGTCTCCATCTCAAATTCAGCAGCCTAGCCAGATTTCCGACAGCCAACCTCCATCGCAACGGCAATCGCAATCTCCCTCAGCGCCCCA

CTCGCGGAATCCTTCCATCTCACATCCAGTGCAACCACCACCGCACATGAACCCCGAGGAGAAGTCGCTCCACATACACATGCCTATCTCGAATACTCCCCCGTTCTT

CGATCAGTCACCCTCTAATGGACTGTCTCATTCCTCATCATTACTTTCACCTCTTCCCACACCAGTTACGGGCATGAATAGTTTTGTTTCTTCGACACCCATGTCTGGGT

ACGACGACTTCGTCAGGACAGTGCGCGATCAACCGGAAAATGAGAGCCCGCAATTCATGATGGATCCTTGGCACTCGATGCCCATGGATACCAGTTACGATCCTATGC

GGATTGACCCCGCTCTGATGATGACCATGGGCATGGACATGACGATGGGACCGCCCCAAGACGGTATCCTTAGTATGATACCCGAAATGTCACCTGCGCAAACATTCG

GACAGCCTGTTCAGACTCCTCGTATGGTGGACGAATCGTTCTCGGACTTGCAAATCGGCAGTTCGGCTTCGATGTTCTACCCCTCTAACAGACATTCCTCAATAGCGGA

CGCCGGCATCCCTGATCTTGGCGCCATTATCGCAGCCCAAGACGGCTGGACGGTCTTCCGTTGCACTCCTTCTATCGCTTCGAGCTCATGTCCGCGGACTGCAAGAC

TGAATCTTGAGAGATTAGAGTTGAGCCTGAAGAACCATGAGGGCTGGGCTAACTGGACCCCATCGTGGGACGAATCTGATTTCCAACAAGGCGGACAGATCGCCGTG

TCAAAACTCCAAGAAGCCACGCGAGACAAACTCCTCGCGATCACACAGAGCTTCCTTCACAAGGCTCTAGATATCCACAAAGACGGAAGCAGCTCATCTAGTAGTGGC

GAGTCTCCAGGATCTACGGCTTCACATTCCAACTTCGTTCTTCTTCCGCCTGCTCGTGTCCTGGAGTACTTCCTCAAGTCGTACGCCAACTCTTTCGAACGCTATTATCC

TACCTCGGCCAGAGGAGTCCTCGACACCAACGAGTTGATGCAGAACTACAATGACAAAGCTTCGAGCTTGCTCATTCTCATGATGATTGCGCAGGGCGCCATGACCAT

TCCGTCCATAGAAGCGAGATGGCTGACTGGTGGCTTTACTGAGGCATGTCGGATATCGCTGTTCGACCTCATCGAGAAGAACATCATTATGGCTGGCGATCCTATTGT

CCTGAGGGCGGCCCTTCTCTTCACTGTTCAAGCGGCGTGGAGTGGCGACAAGTGGCAGATGGACATCGCCATGGGTCAGCGCGGCATGTATTTTTCCATGTTACGAC

ATTCCGGTATTCTCGATGTTCGACCGCCTACCACGCCTCACATGAATGGAAACACTTCGACGGATGTGTTGTGGAAGGACTGGCTTCAGCAAGAAAGCCGCAGCCGGT

AAGTGTTGAAAACCTCGGTACAGTCACGTGCGCGCACTTACTAATATCGTGTAGCCTCATCTACTCTTGGGTCATGGTAGACCAAGACCTATCGCTATTCCACGACACT

GCTCCTCTGTTTTCGGTAACAGAATTTGGCGCGCCAATGCCTGATTCAGATCAACTCTGGCAGGCGCAGACAGCTCCAGAGTGGTCGGAGATGTTCAACCAGGTCCAT

GAATTCTCGAATGGATACTCCTCAGTAGGCTCTGGAGCAAGACCACCGAGTCTCAGGGATCTCTTCCGCTACTTCTTAGATGACGAGATAGTCGTCCAGGATGTTCAG

GAGATTCATCTGACCCCGATGCACCTGCGACTACTACTGCATCCTCTGCAGACCCTTGTGTGCCAATATTGCCAACTCCTCAGTTGCTTTTCCGACAGTGTAGCCTCTC

GATCACGGAATCGGGCATTGACAGCTGCTTCGACTCGCGTACGCCTAGAAGAGGTGCAGGCGTTATTGGGGCGGTGGTTCGACTTGGCGCAACGGTACATGAAGGC

GAACCCAGTATGTCCGATGATGCAAGCAAATCTCGTCATGTTCCATCTTATCAGCATGAATGCTGTGTCAAATTTCCCAGAGATTGAGCGCCTCGCCCGACGGGAGGG

TGTGGACGGCTCGTATCAACAGATGCTGTGGATGCACAAGAAGTGCTTGTCGGATGTGCAGGAGGCCATCACTCACGCTGGCCAAGTTCTTCGCCTCATTCGTGAGAT

GCCACGAGGCATTCGCCCGCCGTGGTGGGCTGGTGCTGTCTATCGGGCAGGGTTGGTCTTCTGGGCTGACTCGCTCGCTCACAACGAGTCCTTGAGTCCCAACCAT

CAAGGGACGTATCAGCCATCTAATACTGCTTTCTCTGTGGATCGCCTCTCTGCTGATCACCCAACCATACTGAGGTACATGTCACGGAGGGAGGGTATTCCCACCTTG

ACCAAGCGCGATGGTAGCCCTGTTCCACTGGATAACGGCTTTGCCGTCCTGTCGCATTGTATCGATGTCCTTGACGAGGGTGTCGCCACCAGGTTTTCCGATGGCATT

CGTAGTAAACTCGAGAAACTTGCGCGCGGTTAG

>*Alternaria hordeiaustralica* (NA)

ATGGTCTTTTGCACATACTGTGGACAGTCGTTCACCAGAGACGAGCATCTCGAACGACATATCTTGACTCGTATGTTGACCCCGCGTCTTTCGCTCTTTCAATCTTCCTC

GCACCAGCTACCATCCAATCTTTCTTCTGGGATATCTGCTAATCACTGTGGATGAGCAGACACCAACGTCAAGCCTTTCAAATGCTTCACATGTCATATGAGCTTTGCTC

GACGGTATGTGGCACCCTTCCTTGCTCTTTCTCATCCTGTGGCATCGACCGTTCGGAACAAAGTACTGACAGCCTCTTCAGAGATCTTTTGCAAAGACACTACACCGTA

CACGGCCGCAACCAGAACAACGAAGAGGGACTCCCCCCACAAGGCATAATACCAAAGTCCGCCGGCCGCACACCCATCGCCTGCAGTAATTGTGCGAAGACAAAGAC

CAAGTGTGACAAGAAGTTTCCGTGCACCAGGTGCGCTCAGAGGAATCTGAAATGCACATTGCGTCCTACAAGGCGGGCATCGAAAGGTGTCCAGCGGGTTGGCGGTC

CTCCTGAGGCTGGGAGCGGGGACTCGAGCGAGAGTGGTAGCAACGCGGATAACCACAATACATCCGGCAATGTCTCTCCAAACCACGATAGCCAACCGAAACAAGC

GATGTCGAAGTCCTCTCCTGCTCAGATCAACCAGACTTCCCATATTCCGGACAGCCAACCACCGTCGCAAAGACAATCGCAGTCGCCCTCGGCCCCTCATTCACGAAA

CCCTTCCATCTCACAACCGGTACAGCCACCGACACACATAAATCCCGAGGAGAAGTCGTTGCATCTTCATATGCCGATCTCCGATACGCCACCCTTTTTCGACCAGTCT

CCTTCCAACGGACTTGCTCAATCATCGTCGCTTCTTTCGCCACTCCCCACCCCGGTGAACGGCATGAATGGATTCGTCACCTCCACACCTATGTCAGGATATGACGACT

TCGTCAGAACGGTACGGGACCAATCCGATAATGAGAGCCCTCAATTCATGATGGACCCTTGGAACGGCATGCCCATGGATACAACCTTTGATCCCATGCGGATAGACC

CCGCGTTGATGATGAGCATGAGTATGGACATGAGCATGGGACCACCTCAGGATGGAATCCTTGGCATGATACCAGAAATGTCGCCTGCTCAAACATTTGGACAGCCTG

TCCAGACACCTCGCATGGACGAGTCCTTTTCGGACCTGCAAATTGGTAGTTCGGCTTCGATGTTCTACCCCTCCAACAGGCACTCTTCGATAGCAGATGCCGGCATAC

CTGATCTTGGTGCCATCATCGCGGCTCAAGATGGCTGGACTGTTTTCCGTTGCACTCCTTCTATTGCTTCAAGCTCGTGTCCACGCACAGCGAGACTCAACCTCGAGC

GGTTAGAGCTGAGCCTCAAGAATCACGAGGGCTGGGCTAACTGGTCCCCATCGTGGGATGAATCCGACTTTCAACAAGGCGGGCAAATCGCGGTATCGAAGTTGCAG

GAAGCCACGCGAGACAAACTTCTCGCCATCACCCAAAGCTTCCTCCACAAGGCTTTGGACATCCACAAAGACGGAAGCAGCTCGTCCAGCAGTGGCGAGTCTCCAGG

ATCCACGGCCTCTCACTCCAACTTCGTTCTTCTTCCACCTGCTCGCGTCCTGGAGTACTTTCTCAAGTCATACGCCAACTCTTTCGAACGCTATTATCCCACCTCCGCCA

GAGGTGTGCTCGATACGAATGAACTAATGCAAAACTACAACGACAAAGCATCGAGCTTGCTCATTCTCATGATGATCGCGCAGGGCGCCATGACCATTCCATCCATAG

AGGCCAGGTGGCTAACTGGTGGCTTCACCGAAGCCTGCCGGATATCGCTCTTCGACCTTATTGAGAAGAACATCATCATGGCTGGTGATCCTATCGTTCTGCGTGCAG

CCCTCCTCTTCACTGTCCAGGCGGCGTGGAGTGGCGACAAATGGCAGATGGACATCGCTATGGGCCAGCGTGGCATGTATTTCTCCATGTTGCGACATTCCGGTATTC

TCGATGTGCGACCGCCGGCAACGCCTCATATCAACGGAAATATTTCGACGGATGCGTTGTGGAAGGACTGGCTTCAGCAAGAAAGCCGCAGTCGGTAAGTGTTGAAA

ACTTCGGTACCGTCACGTGAACGCACTTACTAATATTGGCGCAGCCTCATCTACTCCTGGGTCATGGTAGACCAAGACTTATCGCTCTTCCACGATACCGCTCCACTGT

TCTCAGTAACAGAGTTTGGCGCGCCGATGCCTGATTCGGATCGACTTTGGCAAGCGCAGACCGCCTCAGAGTGGTCTGAGATGTTCAACCAAGTCCATGAGTTCTCGA

ACGGGTACTCTTCAGTAGGCTCTGGAGCCAGACCACCCAGTCTCAGAGATCTGTTCCGTTACTTCCTAGATGACGAAATCGTCGTACAGGATGTACAGGAGATTCATCT

GACACCTATGCACCTGCGTCTGCTCCTCCACCCCCTGCAGACTCTGGTCTGCCAGTATTGCCAGCTTCTCAGCTGCTTTTCCGATAGCGTCGCTTCACGATCGCGGAA

TCGCGCACTGACAGCAGCGTCGACCCGCGTGCGTCTCGAGGAGGTGCAGGCGTTGCTAGGGCGTTGGTTCGATTTGGCGCAACGGTACATGAAGTCCAACCCAGTG

TGTCCGATGATGCAGGCGAACCTTGTCATGTTCCATCTTATCAGCTTGAACGCTGTGTCCAACTTTCCAGAGATAGAGCGCCTTGCGCGTCGAGAGGGTGTGGACGGC

TCATACCAGCAGATGCTGTGGATGCACAAGAAGTGTCTGTCCGATGTGCAGGAGGCCATCACCCACGCCGGTCAGGTCCTTCGACTCATCCGTGAGATGCCAAGAGG

TATCCGGCCGCCATGGTGGGCTGGCGCCGTCTACCGTGCGGGTTTGGTCTTCTGGACTGATTCGCTGGCACATGGCGAGTCCTTGAGTCCCAACCACCAAGGATCGT

ATCAGCCATCGAACTCCGCGTTCTCTGTGGATCGCCTTCCAGCTGACCATCCTACCATACTGAGGTACATGTCTCGTAGGGAGGGCATACCCACTCTGACCAAGCGCG

ACGGTACACACGTCCCTTTGGATAATGGCTTCGCGATCCTGTCGCATTGTATCGACGTCCTTGATGAGGGTGTTGCCACCAGGTTCTCCGATGGCATTCGTAGTAAACT

CGAGAAACTCGCGCGAGGATAA

>*Alternaria incomplexa* (NA)

ATGGTTTTTTGCACATATTGTGGACAGTCGTTCACCAGAGACGAGCATCTCGAACGACATATCTTGACTCGTATGTTGACCCCTCGTCTTTCGCTCTTTCATTCTTCCTC

GCACCAGCTACTATCCAATCTTTCTTCTGGGATATCTGCTAATCACTATGGACGAGCAGACACCAACGTCAAGCCTTTCAAATGCTTCACATGTCATATGAGCTTTGCTC

GACGGTATGTGGCACCCTTCCTTGCTCTTCCTCATCCTGTGGCATCGACCGTTCGGAACAAAGTACTGACAGACTCTTCAGAGATCTTTTGCAAAGACACTACACCGTA

CACGGCCGCAACCAGAACAACGAAGAGGGCCTCCCTCCGCAAGGCATAATACCAAAGTCCGCCGGCCGCACGCCCATCGCCTGCAGTAATTGTGCGAAGACAAAGA

CCAAGTGTGACAAGAAGTTTCCGTGCACCAGGTGCGCTCAGAGGAATCTGAAATGCACATTGCGTCCTACAAGGCGGGCATCGAAAGGTGTCCAGCGGGTTGGCGGT

CCTCCTGAGGCTGGGAGCGGGGATTCGAGCGAGAGTGGCAGCAACGCGGATAACCACAACACATCCGGCAATGTCTCTCCAAACCACGATAGCCAACCAAAACAAGC

GGTGTCGAAGTCCTCTCCCGCTCAGATCCGCCAGACTTCCCATATTCCGGACAGCCAGCCACCGTCGCAAAGACAATCGCAGTCGCCCTCGGCCCCTCATTCACGAA

ACCCTTCCATCTCGCAACCAGTACAGCCACCAACACACATAAATCCCGAGGAGAAGTCGTTGCATCTTCATATGCCGATCTCCGATACGCCGCCCTTTTTCGACCAGTC

TCCTTCCAACGGACTTGCTCAATCATCGTCGCTTCTTTCGCCACTCCCCACCCCGGTGACCGGCATGAATGGCTTCGTCACCTCCACACCTATGTCAGGATATGACGA

CTTCGTTAGAACGGTACGGGACCAATCCGATAATGAGAGCCCTCAATTCATGATGGACCCTTGGAACGGCATGCCCATGGACACAACCTTTGATCCCATGCGGATAGA

CCCCGCGTTGATGATGAGCATGAGTATGGACATGAGTATGGGACCGCCTCAGGATGGTATCCTTGGCATGATACCAGAAATGTCGCCTGCTCAAGCATTTGGACAGCC

TGTTCAGACACCTCGCATGGACGAGTCCTTTTCGGATCTGCAAATTGGTAGTTCGGCTTCGATGTTCTACCCCTCCAACAGGCACTCTTCGATAGCAGATGCCGGCATA

CCTGATCTTGGTGCCATTATCGCGGCTCAAGATGGCTGGACTGTTTTCCGTTGCACTCCTTCTATTGCTTCAAGCTCGTGTCCACGCACAGCGAGACTCAACCTCGAG

CGGTTAGAGCTGAGCCTCAAGAACCACGAGGGCTGGGCTAACTGGTCCCCATCGTGGGATGAATCCGACTTTCAACAAGGCGGACAAATCGCGGTATCGAAGTTGCA

GGAAGCCACGCGAGACAAACTTCTCGCCATCACCCAAAGCTTCCTCCACAAGGCTTTGGACATCCACAAAGACGGCAGTAGCTCGTCCAGCAGTGGCGAGTCTCCAG

GATCCACGGCCTCTCACTCCAACTTCGTTCTTCTTCCACCTGCTCGCGTCCTGGAGTACTTTCTCAAATCATACGCCAACTCTTTCGAACGCTATTATCCCACCTCCGCC

AGAGGTGTACTCGATACGAATGAACTGATGCAAAACTACAACGATAAAGCATCGAGCTTGCTCATTCTCATGATGATTGCGCAGGGCGCCATGACCATTCCATCCATAG

AGGCCAGGTGGCTAACTGGTGGCTTCACCGAAGCCTGCCGGATATCGCTCTTCGACCTTATCGAGAAGAACATCATCATGGCTGGTGATCCTATCGTTCTGCGTGCAG

CCCTCCTCTTCACTGTCCAGGCGGCGTGGAGTGGCGACAAATGGCAGATGGACATCGCTATGGGCCAGCGTGGCATGTATTTTTCCATGTTGCGACATTCCGGTATTC

TCGATGTGCGACCGCCAGCAACGCCTCATATAAACGGAAACATTTCGACGGATGCGTTGTGGAAGGACTGGCTTCAGCAAGAAAGCCGCAGTCGGTAAGTGTTGAAA

ACTTCGGTACCGTCACGTGAACGCACTTACTAATATTGGCGCAGCCTCATCTACTCCTGGGTCATGGTAGACCAAGACTTATCGCTGTTCCACGATACCGCTCCACTGT

TTTCAGTAACAGAGTTTGGCGCGCCGATGCCTGATTCGGATCGACTTTGGCAAGCGCAGACCGCCTCGGAGTGGTCTGAAATGTTCAACCAAGTCCACGAGTTCTCGA

ACGGGTACTCTTCAGTAGGCTCTGGGGCCAGACCACCCAGTCTCAGAGATCTCTTCCGTTACTTCCTAGATGACGAAATCGTCGTACAGGATGTACAGGAGATTCATC

TGACACCTATGCACCTGCGTCTGCTCCTCCACCCCCTGCAGACTTTGGTCTGCCAGTATTGCCAGCTTCTCAGCTGCTTTTCCGATAGCGTCGCTTCACGATCGCGGA

ATCGCGCACTGACAGCAGCGTCGACCCGCGTGCGTCTCGAGGAGGTGCAGGCGTTGCTAGGGCGTTGGTTCGATTTGGCGCAACGGTATATGAAGTCCAACCCAGT

GTGTCCGATGATGCAGGCCAACCTTGTCATGTTCCATCTTATCAGCTTGAACGCTGTGTCCAACTTTCCAGAGATAGAGCGCCTTGCGCGTCGAGAGGGCGTGGACG

GCTCATACCAGCAGATGCTGTGGATGCACAAGAAGTGTCTGTCCGATGTGCAGGAGGCCATCACCCACGCCGGTCAGGTCCTTCGACTCATCCGTGAGATGCCAAGA

GGTATCCGGCCGCCATGGTGGGCTGGCGCCGTCTACCGTGCGGGTTTGGTCTTCTGGACTGATTCGCTGGCACATGGCGAGTCCTTGAGTCCCAACCACCAAGGAT

CGTATCAGCCATCGAACTCCGCGTTCTCTGTGGATCGCCTTCCTGCTGACCACCCAACCATACTGAGGTACATGTCTCGCAGAGAGGGCATCCCCACTCTGACCAAGC

GAGACGGTACACACGTCCCCTTGGATAACGGCTTCGCGATCTTGTCGCATTGTATCGACGTCCTTGATGAAGGTGTTGCCACCAGGTTCTCCGATGGCATTCGTAGTA

AACTCGAGAAACTCGCGCGAGGATAA

>*Alternaria infectoria* (J4E92_008460[a])

ATGGTCTTTTGCACATACTGTGGACAGTCGTTCACCAGAGACGAGCATCTCGAACGACATATCTTGACTCGTATGTTGACCCCGCGTCTTTCGCTCTTTATATCTTCCTC

ACACCAGCTACCATCCAATCTTTCTTCTGGGATATCTGCTAATCACTGTGGATGAGCAGACACCAACGTCAAGCCTTTCAAATGCTTCACATGTCATATGAGCTTTGCTC

GACGGTATGTGGCACCCTTCCTTGCTCTTTCTCATCCTGTGGCATCGACCGTTCGGAACAAAGTACTGACAGCCTCTTCAGAGATCTTTTGCAAAGACACTACACCGTA

CACGGCCGCAACCAGAACAACGAAGAGGGACTCCCCCCACAAGGCATAATACCAAAGTCCGCCGGCCGCACACCCATCGCCTGCAGTAATTGTGCCAAGACAAAGAC

CAAGTGTGACAAGAAGTTTCCGTGCACCAGGTGCGCTCAGAGGAATCTGAAATGCACATTGCGTCCTACAAGGCGGGCATCGAAAGGTGTCCAGCGGGTTGGCGGTC

CTCCTGAGGCTGGGAGCGGGGACTCGAGCGAGAGTGGCAGCAACGCGGATAACCACAATACATCCGGCAATGTCTCTCCAAACCACGATAGCCAACCGAAACAAGC

GATGTCGAAGTCCTCTCCTGCTCAGATCAACCAGACTGCCCATATTCCGGACAGCCAACCACCGTCGCAAAGACAATCGCAGTCGCCCTCGGCCCCTCATTCACGAAA

CCCTTCCATCTCACAACCGGTACAGCCACCGACACACATAAATCCCGAGGAGAAGTCGTTGCATCTTCATATGCCGATCTCCGATACGCCGCCCTTTTTCGACCAGTC

CCCTTCCAACGGACTTGCTCAATCATCGTCGCTTCTTTCGCCACTCCCCACCCCGGTGAACGGCATGAATGGATTCGTCACCTCCACACCTATGTCAGGATATGACGA

CTTCGTCAGAACGGTACGGGACCAATCCGATAATGAGAGCCCTCAATTCATGATGGACCCTTGGAACGGCATGCCCATGGATACAACCTTTGATCCCATGCGGATAGA

CCCCGCGTTGATGATGAGCATGAGTATGGACATGAGCATGGGACCACCTCAGGATGGCATCCTTGGCATGATACCAGAAATGTCGCCTGCTCAAACATTTGGACAGCC

TGTCCAGACACCTCGCATGGACGAGTCCTTTTCGGACCTGCAAATTGGTAGTTCGGCTTCGATGTTCTACCCCTCCAACAGGCACTCTTCGATAGCAGATGCCGGCAT

ACCTGATCTTGGTGCCATCATCGCGGCTCAAGATGGCTGGACTGTTTTCCGTTGCACTCCTTCTATTGCTTCAAGCTCGTGTCCACGCACAGCGAGACTCAACCTCGA

GCGGTTAGAGCTGAGCCTCAAGAATCACGAGGGCTGGGCTAACTGGTCCCCATCGTGGGATGAATCCGACTTTCAACAAGGCGGGCAAATCGCGGTATCGAAGTTGC

AGGAAGCCACGCGAGACAAACTTCTCGCCATCACCCAAAGTTTCCTCCACAAGGCTTTGGACATCCACAAAGACGGAAGCAGCTCGTCCAGCAGTGGCGAGTCTCCA

GGATCCACGGCCTCTCACTCCAACTTCGTTCTTCTTCCACCTGCTCGTGTCCTGGAGTACTTTCTCAAGTCATACGCCAACTCTTTCGAACGCTATTATCCCACCTCCG

CCAGAGGTGTGCTCGATACGAATGAACTAATGCAAAACTACAACGACAAAGCGTCGAGCTTGCTCATTCTCATGATGATCGCGCAGGGCGCCATGACCATTCCATCCA

TAGAGGCCAGGTGGCTAACTGGTGGCTTCACCGAAGCCTGCCGGATATCGCTCTTCGACCTTATTGAGAAGAACATCATCATGGCTGGTGATCCTATCGTTCTGCGTG

CAGCCCTCCTCTTCACTGTCCAGGCGGCGTGGAGTGGCGACAAATGGCAGATGGACATCGCTATGGGCCAGCGTGGCATGTATTTCTCCATGTTGCGACATTCCGGT

ATTCTCGATGTGCGACCGCCGGCAACGCCTCATATCAACGGAAATATTTCGACGGATGCGTTGTGGAAGGACTGGCTTCAGCAAGAAAGCCGCAGTCGGTAAGTGTT

GAAAACTTCGGTACCGTCACGTGAACGCACTTACTAATATTGGCGCAGCCTCATCTACTCCTGGGTCATGGTAGACCAAGACTTATCGCTCTTCCACGATACCGCTCCA

CTGTTCTCAGTAACAGAGTTTGGCGCGCCGATGCCTGATTCGGATCGACTTTGGCAAGCGCAGACCGCCTCAGAGTGGTCTGAGATGTTCAACCAAGTCCATGAGTTC

TCGAACGGGTACTCTTCAGTAGGCTCTGGGGCCAGACCACCCAGTCTCAGAGACCTGTTCCGTTACTTCCTAGATGACGAAATCGTCGTACAGGATGTACAGGAGATT

CATCTGACACCTATGCACCTGCGTCTGCTCCTCCACCCCCTGCAGACTCTGGTCTGCCAGTATTGCCAGCTTCTCAGCTGCTTTTCCGATAGCGTCGCTTCACGATCG

CGGAATCGCGCACTGACAGCAGCGTCGACCCGCGTGCGTCTCGAGGAGGTGCAGGCGTTGCTAGGGCGTTGGTTCGATTTGGCGCAACGGTACATGAAGTCCAACC

CAGTGTGTCCGATGATGCAGGCGAACCTTGTCATGTTCCATCTTATCAGCTTGAACGCTGTGTCCAACTTTCCAGAGATAGAGCGCCTTGCGCGTCGAGAGGGTGTGG

ACGGCTCATACCAGCAGATGCTGTGGATGCACAAGAAGTGTCTGTCCGATGTGCAGGAGGCCATCACCCACGCCGGTCAGGTCCTTCGACTCATCCGTGAGATGCCA

AGAGGTATCCGGCCGCCATGGTGGGCTGGCGCCGTCTACCGTGCGGGTTTGGTCTTCTGGACTGATTCGCTGGCACATGGCGAGTCCTTGAGTCCCAACCACCAAG

GATCGTATCAGCCATCGAACTCCGCGTTCTCTGTGGATCGCCTTCCAGCTGACCATCCTACCATACTGAGGTACATGTCTCGTAGGGAGGGCATACCCACTCTGACCA

AGCGCGACGGTGCACACGTCCCCTTGGATAACGGCTTCGCGATCCTGTCGCATTGTATCGACGTCCTTGATGAGGGTGTTGCCACCAGGTTCTCCGATGGCATTCGT

AGTAAACTCGAGAAACTCGCGCGAGGATAA

>*Alternaria limoniasperae* (NA)

ATGGTCTTCTGCACATATTGTGGACAGTCGTTCACCAGGGACGAACATCTCGAACGACATATCCTAACTCGTAAGTCAACTCTTGCTCTGTCCATGAGGGTGGATTTCC

GCAATGTCGAGTTCAATCCGATCGTTCCGTAGCATCTCCGCTGACCGTCAATGCCAAACAGACACCAATGTCAAGCCATTCAAGTGCTTTACATGTCATATGAGCTTTG

CTCGACGGTAAGTCCCACCCTGCGTGCGCTCCTTCCTCTTCCTGTTTGGTCATAATTTCCGTACCGAACGAAACGCTGACAATCTTTCCAGAGATCTTTTGCAAAGACA

TTACACCGTACACGGCCGCAACCAGAACAACGAAGAAGGCCTCCCACCGCAAGGCATCATACCTAAGTCCGCCGGCCGCACACCCATCGCCTGCAGTAATTGTGCAA

AGACGAAGACGAAATGCGACAAGAAGTTTCCTTGCACCCGGTGCGCTCAAAGGAACCTGAAGTGTACTCTACGTCCTACACGGCGAGCATCGAAAGGTGTCCAACGA

GTAGGAGGTCCTCCTGAAACAGGCAGTGGTGATTCTAGCGAGAGTGGTAGCAACGCGGATAACCAGAACACATCCGGCAATGTCTCTCCAAACCACGATGGCCAGTC

GAAACAAGCAACCGCTCAGTCGTCTCCATCTCAAATTCAGCAGCCTAGCCAGATTTCTGACAGCCAACCTCCATCGCAACGGCAATCGCAATCTCCCTCAGCGCCCCA

CTCGCGGAATCCTTCCATCTCACATCCAGTGCAACCACCACCGCACATGAACCCCGAGGAGAAGTCGCTCCACATACACATGCCTATCTCGAATACTCCCCCGTTCTT

CGATCAGTCACCCTCTAACGGACTGTCTCATTCCTCATCATTACTTTCACCTCTTCCCACACCAGTTACGGGCATGAATAGTTTTGTTTCTTCGACACCCATGTCTGGGT

ACGACGACTTCGTCAGGACAGTGCGCGATCAACCGGAAAATGAGAGCCCGCAATTCATGATGGATCCTTGGCACTCGATGCCCATGGATACGAGTTACGATCCTATGC

GGATTGACCCCGCTCTGATGATGACCATGGGCATGGACATGACGATGGGACCGCCCCAAGACGGTATCCTTAGTATGATACCCGAAATGTCACCTGCGCAAACATTCG

GACAGCCTGTTCAGACTCCTCGTATGGTGGACGAATCGTTCTCGGACTTGCAAATCGGCAGTTCGGCTTCGATGTTCTACCCCTCTAACAGACATTCCTCAATAGCGGA

CGCCGGCATCCCTGATCTTGGCGCCATTATCGCAGCCCAAGACGGCTGGACGGTCTTCCGTTGCACTCCTTCTATCGCTTCGAGCTCATGTCCGCGGACCGCAAGGC

TGAATCTTGAGAGATTAGAGTTGAGCCTGAAGAACCATGAGGGCTGGGCTAACTGGACCCCATCGTGGGACGAATCTGATTTCCAACAAGGCGGACAGATCGCCGTG

TCAAAACTCCAGGAAGCCACACGAGACAAACTTCTCGCGATCACACAGAGCTTCCTTCACAAGGCTCTAGATATCCACAAAGATGGGAGCAGCTCGTCTAGTAGTGGC

GAGTCTCCAGGATCTACGGCTTCACATTCCAACTTCGTTCTTCTTCCGCCTGCTCGTGTCCTGGAGTACTTCCTCAAGTCGTACGCCAACTCTTTCGAACGCTATTATCC

TACCTCGGCCAGAGGAGTCCTCGACACCAACGAGTTGATGCAGAACTACAATGACAAAGCTTCGAGCTTGCTCATTCTCATGATGATTGCGCAGGGCGCTATGACCAT

TCCGTCCATAGAAGCGAGATGGCTGACTGGCGGCTTTACTGAGGCATGTCGGATATCCCTGTTCGACCTCATCGAGAAGAACATCATTATGGCTGGCGATCCTATTGT

CCTGAGGGCGGCCCTTCTCTTCACTGTTCAAGCGGCGTGGAGTGGCGACAAGTGGCAGATGGACATCGCCATGGGTCAGCGCGGCATGTATTTTTCCATGTTACGAC

ATTCCGGTATTCTCGATGTTCGACCGCCTACCACGCCTCACATGAATGGAAACACTTCGACGGATGTGTTGTGGAAGGACTGGCTTCAGCAAGAAAGCCGCAGCCGGT

AAGTGTTGAAAACCTCGGTACAGTCACGTGCGCGCACTTACTAATATCGTGTAGCCTCATCTACTCTTGGGTCATGGTAGACCAAGACCTATCGCTATTCCACGATACC

GCTCCCCTGTTTTCGGTAACAGAATTTGGCGCGCCAATGCCTGATTCAGATCAACTCTGGCAGGCGCAGACAGCTCCAGAGTGGTCGGAGATGTTCAACCAGGTCCAT

GAATTCTCGAATGGATACTCCTCAGTAGGCTCTGGAGCAAGACCACCGAGTCTCAGAGATCTCTTCCGCTACTTCCTAGATGACGAGATAGTCGTCCAGGATGTTCAG

GAGATTCATCTGACCCCGATGCACCTGCGACTACTACTGCACCCTCTGCAGACCCTTGTGTGCCAATATTGCCAACTCCTCAGTTGCTTCTCCGACAGTGTAGCCTCTC

GATCGCGGAATCGGGCATTGACAGCTGCTTCGACTCGCGTACGCCTAGAAGAGGTACAGGCGTTATTGGGGCGGTGGTTCGACTTGGCGCAACGGTACATGAAGGC

GAACCCAGTATGTCCGATGATGCAAGCAAATCTCGTCATGTTCCATCTTATCAGCATGAATGCTGTGTCAAATTTCCCAGAGATTGAGCGCCTCGCCCGACGGGAGGG

TGTGGACGGCTCGTATCAACAGATGCTGTGGATGCACAAGAAGTGCTTGTCGGATGTGCAGGAGGCCATCACTCACGCTGGCCAAGTTCTTCGCCTCATTCGTGAGAT

GCCACGAGGCATTCGCCCGCCGTGGTGGGCTGGTGCTGTCTATCGGGCAGGGTTGGTCTTCTGGGCTGACTCGCTCGCTCACAACGAGTCCTTGAGTCCCAACCAT

CAAGGGACATATCAGCCATCTAATACTGCTTTCTCTGTGGATCGCCTCTCTGCTGACCACCCAACCATACTGAGGTACATGTCACGGAGGGAGGGTATTCCCACCTTG

ACCAAGCGCGATGGTAGCCCTGTTCCACTGGATAACGGCTTTGCCGTCCTGTCGCATTGTATCGATGTCCTTGACGAGGGTGTCGCCACCAGGTTTTCCGATGGCATT

CGTAGTAAACTCGAGAAACTTGCGCGCGGTTAA

>*Alternaria longipes* (NA)

ATGGTCTTCTGCACATATTGTGGACAGTCGTTCACCAGGGACGAACATCTCGAACGACATATCCTAACTCGTAAGTCAACTCTTGCTCTGTCCATGAAGGTGGATTTCC

GCAATGTCGAGTTCAATCCGATCGTTCCGTAGCATCTCCGCTGACCGTCAATGCCAAACAGACACCAATGTCAAGCCATTCAAGTGCTTTACATGTCATATGAGCTTTG

CTCGACGGTAAGTCCCACCCTGCGTGCGCTCCTTCCTCTTCCTGTTTGGTCATAATTTCCGTACCGAACGAAACGCTGACAATCTTTCCAGAGATCTTTTGCAAAGACA

TTACACCGTACACGGCCGCAACCAGAACAACGAAGAAGGCCTCCCACCGCAAGGCATCATACCTAAGTCCGCCGGCCGCACACCCATCGCCTGCAGTAATTGTGCAA

AGACGAAGACGAAATGCGACAAGAAGTTTCCTTGCACCCGGTGCGCTCAAAGGAACCTGAAGTGTACTCTACGTCCTACACGGCGAGCATCGAAAGGTGTCCAACGA

GTAGGAGGTCCTCCTGAAACAGGCAGTGGTGATTCTAGCGAGAGTGGCAGCAACGCGGATAACCAGAACACATCCGGCAATGTCTCTCCAAACCACGATGGCCAGTC

GAAACAAGCAACCGCTCAGTCGTCTCCATCTCAAATTCAGCAGCCTAGCCAGATTTCTGACAGCCAACCTCCATCGCAACGGCAATCGCAATCTCCCTCAGCGCCCCA

CTCGCGGAATCCTTCCATCTCACATCCAGTGCAACCACCACCGCACATGAACCCCGAAGAGAAGTCGCTCCACATACACATGCCTATCTCGAATACTCCCCCGTTCTTC

GATCAGTCACCCTCTAACGGACTGTCTCATTCCTCATCATTACTTTCACCTCTTCCCACACCAGTTACGGGCATGAATAGTTTTGTATCTTCGACACCCATGTCTGGGTA

CGACGACTTCGTCAGAACAGTGCGCGATCAACCGGAAAATGAGAGTCCGCAGTTCATGATGGATCCTTGGCACTCGATGCCCATGGATACGAGTTACGATCCTATGCG

GATTGACCCCGCTCTGATGATGACCATGGGCATGGACATGACGATGGGACCGCCCCAAGACGGTATCCTTAGTATGATACCCGAAATGTCACCTGCGCAAACATTCGG

ACAGCCTGTTCAGACTCCTCGTATGGTGGACGAATCGTTCTCGGACTTGCAAATCGGCAGTTCGGCTTCGATGTTCTACCCCTCTAACAGACATTCCTCAATAGCGGAC

GCCGGCATCCCTGATCTTGGCGCCATTATTGCAGCCCAAGACGGCTGGACGGTCTTCCGTTGCACTCCTTCTATCGCTTCGAGCTCATGTCCGCGGACCGCAAGGCT

GAATCTTGAGAGATTAGAGTTGAGCCTGAAGAACCATGAGGGCTGGGCTAACTGGACCCCATCGTGGGACGAATCTGATTTCCAACAAGGCGGACAGATCGCCGTGT

CAAAACTCCAGGAAGCCACACGAGACAAACTCTTCGCGATCACACAGAGCTTCCTTCACAAGGCTCTAGATATCCACAAAGATGGAAGCAGCTCGTCTAGTAGTGGCG

AGTCTCCAGGATCTACGGCTTCACATTCCAACTTCGTTCTTCTTCCGCCTGCTCGTGTCCTGGAGTACTTCCTCAAGTCGTACGCCAACTCTTTCGAACGCTATTATCCT

ACCTCGGCCAGAGGAGTCCTCGACACCAACGAGTTGATGCAGAACTACAATGACAAAGCTTCGAGCTTGCTCATTCTCATGATGATTGCGCAGGGCGCTATGACCATT

CCGTCCATAGAAGCGAGATGGCTGACTGGCGGCTTTACTGAGGCATGTCGGATATCCCTGTTCGATCTCATCGAGAAGAACATCATTATGGCTGGCGATCCTATTGTC

CTGAGGGCGGCCCTTCTCTTCACTGTTCAAGCGGCGTGGAGTGGCGACAAGTGGCAGATGGACATCGCCATGGGTCAGCGCGGCATGTATTTTTCCATGTTACGACA

TTCCGGTATTCTCGATGTTCGACCGCCTACCACGCCTCACATGAATGGAAATACTTCGACGGATGTGTTGTGGAAGGACTGGCTTCAGCAAGAAAGCCGCAGCCGGTA

AGTGTTGAAAACCTCGGTACAGTCACGTGCGCGCACTTACTAATATCGTGTAGCCTCATCTACTCTTGGGTCATGGTAGACCAAGACCTATCGCTATTCCACGATACCG

CTCCCCTGTTTTCGGTAACAGAATTTGGCGCGCCAATGCCTGATTCAGATCAACTCTGGCAGGCGCAGACAGCTCCAGAGTGGTCGGAGATGTTCAACCAGGTCCATG

AATTCTCGAATGGATACTCCTCAGTAGGCTCTGGAGCAAGACCACCGAGTCTCAGAGATCTCTTCCGCTACTTCCTAGACGACGAGATAGTCGTCCAGGATGTTCAAG

AGATTCATCTGACCCCAATGCACCTGCGACTACTACTGCACCCTCTGCAGACCCTTGTGTGCCAATATTGCCAACTCCTCAGTTGCTTTTCCGACAGTGTAGCCTCTCG

ATCGCGGAATCGGGCATTGACAGCTGCTTCGACTCGCGTACGCCTAGAAGAGGTGCAGGCGTTATTGGGGCGGTGGTTCGACTTGGCGCAACGGTACATGAAGGCG

AACCCAGTATGTCCGATGATGCAAGCAAATCTCGTCATGTTCCATCTTATCAGCATGAATGCTGTGTCAAATTTCCCAGAGATTGAGCGCCTCGCCCGACGGGAGGGT

GTGGACGGCTCGTATCAACAGATGTTGTGGATGCACAAGAAGTGCTTGTCGGATGTGCAGGAGGCCATCACTCACGCTGGCCAAGTTCTTCGCCTCATTCGTGAGATG

CCACGAGGCATTCGCCCGCCGTGGTGGGCTGGTGCTGTCTATCGGGCAGGGTTGGTCTTCTGGGCTGACTCGCTCGCTCACAACGAGTCCTTGAGTCCCAACCATCA

AAAGACATATCAGCCGTCTAATACTGCTTTCTCTGTGGATCGCCTCTCTGCTGACCACCCAACCATACTGAGGTACATGTCACGGAGGGAGGGTATTCCCACCTTGACC

AAGCGCGATGGTAGCCCTGTTCCACTGGATAACGGCTTTGCCATCCTGTCGCATTGTATCGATGTCCTTGACGAGGGTGTCGCCACCAGGTTTTCCGATGGCATTCGT

AGTAAACTCGAGAAACTTGCGCGCGGTTAA

>*Alternaria macrospora* (NA)

ATGGTCTTCTGCACATATTGTGGACAGTCGTTCACCAGGGACGAGCATCTCGAACGACATATCCTAACTCGTAAGTTGGCTCTTGCTCCGTCCTTGAGGGTTGATTTCC

GCAGTGACGAGTACAGTCCGGTCCTTCCGTGGGATCTTCGCTAACCGCCATTGCCAAACAGACACAAATGTCAAGCCCTTCAAGTGCTTTACATGCCATATGAGTTTCG

CTCGACGGTCAGTCTTACCCTCCATGCACTCCTTGCTCTTTCTCGGCCGATGGCAATCCTCGTAGGGAACGAAACGCTGACGATCCTCCCAGAGATCTTTTGCAAAGA

CATTATACCGTCCACGGCCGCAACCAGAACAACGAGGAAGGTCTGCCACCGCAAGGGATCATACCTAAATCCGCTGGCCGCACACCCATCGCCTGCAGTAATTGTGC

AAAGACGAAGACGAAATGCGACAAGAAGTTTCCTTGCACCCGGTGCGCTCAGAGGAACCTGAAATGTACTCTGCGTCCTACACGTCGAGCATCGAAAGGTGTCCAGC

GAGTGGGGGGTCCTCCTGAAACAGGGAGTGGTGATTCGAGCGAGAGTGGTAGCAATGCGGACAACCAGATCACATCCGGCAATGTCTCTCCAAACCACGACGGTCAA

TCGAAACAAGCAACCGCACAGTCATCTCCATCTCAGATTCAGCAGCCTGCCCTGATGTCTGACAGCCAACCTCCGTCACAACGACAATCGCAGTCGCCTTCAGCGCCC

CACTCGCGGAATCCTTCCATCTCGCATCCAGTGCAACCACCACCGCACATGAATCCCGAGGAGAAGTCACTCCATATACACATGCCGATTTCAAATACTCCCCCATTCT

TCGACCAGTCACCGTCCAATGGCCTAGCTCATTCCTCGTCGTTACTTTCGCCTCTTCCCACACCGGTTACGGGCATGAATAGCTTTGTCTCCTCGACACCCATGTCTGG

ATACGACGATTTTGTCAGAACAGTGCGCGATCAGCCGGAGAATGAGAGTCCGCAATTCATGATGGATCCTTGGCATTCGATGCCTATGGACACGAACTTCGATCCTAT

GCGGATTGACCCCACTCTGATGATGACCATGGGTATGGATATGACCATGGGACCACCCCAAGACGGTATTCTTAGTATGATACCCGAAATGTCACCTGCGCAGACGTT

CGGACAACCTGTTCAGACTCCTCGCATGGTGGACGAATCATTATCAGACTTGCATATTGGCAGTTCGGCTTCGATGTTCTACCCTTCCAACAGACATTCTTCGATAGCG

GACGCCGGCATCCCTGATCTTGGAGCCATCATCGCGGCTCAAGACGGCTGGACTGTTTTCCGTTGCACTCCTTCTATTGCTTCCAGCTCATGTCCGCGGACTGCAAGG

CTGAATCTTGAGCGCCTAGAGTTGAGCCTGAAGAACCATGAGGGTTGGGCTAACTGGGCCCCATCCTGGGATGAATCCGATTTCCAACAAGGCGGGCAAATAGCCGT

GTCGAAATTACAGGAAGCCACACGAGACAAACTCCTCGCCATCACACAGAGCTTTCTTCACAAAGCCCTAGACATCCACAAAGACGGAAACAGCACGTCCAGTAGTGG

TGAATCTCCAGGGTCCACGGCTTCGCATTCCAACTTTGTCCTTCTTCCGCCTGCTCGCGTCCTGGAGTACTTCCTCAAGTCGTACGCCAACTCTTTCGAACGCTATTAT

CCTACCTCCGCCAGAGGAGTCCTTGACGCCAACGAGTTGATGCAGAACTATAACGACAAGGCATCAAGCTTGCTCATTCTCATGATGATCGCGCAGGGCGCCATGACT

ATTCCGTCCATAGAAGCGAGATGGCTGACCGGTGGGTTTACCGAGGCATGTCGGATATCGCTGTTCGACCTGATCGAGAAGAACATTATCATGGCTGGCGATCCTATC

GTCCTAAGAGCAGCCCTTCTCTTTACTGTCCAAGCGGCGTGGAGTGGCGACAAGTGGCAGATGGACATCGCCATGGGTCAGCGCGGCATGTATTTTTCCATGTTACG

GCACTCCGGTATTCTTGATGTCCGACCGCCTACCACGCCTCACATGAACGGGAACACGTCGACGGATGTGTTGTGGAAGGACTGGCTCCAGCAAGAAAGCCGTAGCC

GGTAAGTGATGAAAACCTCGGTACAGTCACGTGCGCACACTTACTAATATCATGTAGCCTCATCTACTCTTGGGTCATGGTAGACCAGGACCTATCGCTCTTCCATGAT

ACCGCCCCGCTGTTCTCGGTAACAGAATTCGGCGCGCCAATGCCTGATTCAGACCAGCTCTGGCAGGCGCAGACTGCTTCGGAGTGGTCGGAGATGTTCAACCAAGT

GCATGAATTCTCCAATGGGTACTCCTCAGTAGGCTCTGGAGCAAGACCACCGAGTCTCAGAGATCTCTTCCGGTACTTCCTAGATGACGAGATAGTCGTCCAGGATGT

CCAGGAGATTCATCTGACCCCAATGCACCTGCGGCTGCTGCTACACCCTCTGCAGACCCTTGTGTGCCAGTATTGTCAACTCCTCAGCTGCTTTTCAGATAGCGTAGC

CTCTCGATCGAGGAATCGGGCACTGACTGCCGCTTCGACTCGCGTGCGCTTAGAAGAGGTACAGGCCCTACTGGGGCGGTGGTTCGACTTGGCGCAAAGATACATGA

AGGCGAACCTAGTATGTCCAATGATGCAGGCAAACCTCGTCATGTTCCATCTGATCAGCATGAATGCTGTGTCGAACTTCCCAGAGATCGAGCGCCTCGCGCGACGAG

AGGGCGTGGACGGCTCGTACCAGCAGATGTTGTGGATGCACAAGAAGTGCTTGTCGGATGTGCAGGAGGCCATAACCCATGCTGGTCAAGTTCTTCGCCTCATTCGT

GAGATGCCACGAGGCATCCGCCCGCCGTGGTGGGCTGGTGCTGTCTATCGGGCGGGGTTGGTGTTCTGGACTGATTCACTCGCCCATAACGAGTCTCTGAGCCCCA

ATCACCAAGGAGCATATCAGCAGCCATCTAATACTGCTTTCTCTGTGGATCGACTTTCTGCTGACCACCCTACCATACTGAGGTACATGTCACGAAGGGAGGGCATCCC

CACCTTGACCAAGCGTGACGGAAACCCCGTTCCACTGGATAACGGCTTCGCTATCCTGTCGCATTGTATCGATGTCCTTGACGAGGGCATCGCCACCAGATTTTCCGA

TGGCATTCGTAGTAAACTCGAGAAACTTGCGCGTGGTTGA

>*Alternaria mali* (AML_PT04777[c])

ATGGTCTTCTGCACATATTGCGGACAGTCGTTCACCAGGGACGAACATCTCGAACGACATATCCTAACTCGTAAGTCAACTCTTGCTCTGTCCATGAGGGTGGATTTCC

GCAACGTCGAGTTCAATCCGATCGTTCCGTAGCATCTCCGCTGACCGTCAATGCCAAACAGACACCAATGTCAAGCCATTCAAGTGCTTTACATGTCATATGAGCTTTG

CTCGACGGTAAGTCCCACCCTGCGTGCGCTCCTTCCTCTTCTTGTTTGGTCATAATTTCCGTACCGAACGAAACGCTGACAATCTTTCCAGAGATCTTTTGCAAAGACAT

TACACCGTACACGGCCGCAACCAGAACAACGAAGAAGGCCTCCCACCGCAAGGCATCATACCTAAGTCCGCCGGCCGCACACCCATCGCCTGCAGTAATTGTGCAAA

GACGAAGACGAAATGCGACAAGAAGTTTCCTTGCACCCGGTGCGCTCAAAGGAACCTGAAGTGTACTCTACGTCCTACACGGCGAGCATCGAAAGGTGTCCAGCGAG

TAGGAGGTCCTCCTGAAACAGGCAGTGGTGATTCTAGCGAGAGTGGTAGCAACGCGGATAACCAGAACACATCCGGCAATGTCTCTCCAAACCACGATGGCCAGTCG

AAACAAGCAACCGCTCAGTCGTCTCCATCTCAAATTCAGCAGCCTAGCCAGATTTCTGACAGCCAACCTCCATCGCAACGGCAATCGCAATCTCCCTCAGCGCCCCAC

TCGCGAAATCCTTCCATCTCACATCCAGTGCAACCACCACCGCACATGAACCCCGAGGAGAAGTCGCTCCACATACACATGCCTATCTCGAATACTCCCCCGTTCTTC

GATCAGTCACCCTCTAACGGACTGTCTCATTCCTCATCATTACTTTCACCTCTTCCCACACCAGTGACGGGCATGAATAGTTTTGTTTCTTCGACACCCATGTCTGGATA

CGACGACTTCGTCAGGACAGTGCGCGATCAACCGGAAAATGAGAGCCCACAATTCATGATGGATCCTTGGCACTCGATGCCCATGGATACGAGTTACGATCCTATGCG

GATTGACCCCGCTCTGATGATGACCATGGGCATGGACATGACGATGGGACCGCCCCAAGACGGTATCCTTAGTATGATACCCGAAATGTCACCTGCGCAAACATTCGG

ACAGCCTGTTCAGACTCCTCGTATGGTGGACGAATCGTTCTCGGACTTGCAAATCGGCAGTTCGGCTTCGATGTTCTACCCCTCTAACAGACATTCCTCAATAGCGGAC

GCCGGCATCCCTGATCTTGGCGCCATTATCGCAGCCCAAGACGGCTGGACGGTCTTCCGTTGCACTCCTTCTATCGCTTCGAGCTCATGTCCGCGGACTGCAAGGCT

GAATCTTGAGAGATTAGAGTTGAGCCTGAAGAACCATGAGGGCTGGGCTAACTGGACCCCATCGTGGGACGAGTCTGATTTCCAACAAGGCGGACAGATCGCCGTGT

CAAAACTCCAAGAAGCCACACGAGACAAACTCCTCGCGATCACACAGAGCTTCCTTCACAAGGCTCTAGATATCCACAAAGATGGAAGCAGCTCGTCTAGTAGTGGTG

AGTCTCCAGGATCTACGGCTTCACATTCCAACTTCGTTCTTCTTCCGCCTGCTCGCGTCCTGGAGTACTTCCTCAAGTCGTACGCCAACTCTTTCGAACGCTATTATCCT

ACCTCGGCCAGAGGAGTCCTCGACACCAACGAGTTGATGCAGAACTACAATGACAAAGCTTCGAGCTTGCTCATTCTCATGATGATTGCGCAGGGCGCTATGACCATT

CCGTCCATAGAAGCGAGATGGCTGACTGGTGGCTTTACTGAGGCATGTCGGATATCCCTGTTCGACCTCATCGAGAAGAACATCATTATGGCTGGCGATCCTATTGTC

CTGAGGGCGGCCCTTCTCTTCACTGTTCAAGCGGCGTGGAGTGGCGACAAGTGGCAGATGGACATCGCCATGGGCCAGCGCGGCATGTATTTTTCCATGTTACGACA

TTCCGGTATTCTCGATGTTCGACCGCCTACCACGCCTCACATGAATGGAAACACTTCGACGGATGTGTTGTGGAAGGACTGGCTTCAGCAAGAAAGCCGCAGCCGGTA

AGTGTTGAAAACCTCAGTACAGGCACGTGCGCGCACTTACTAATATCGTGTAGCCTCATCTACTCTTGGGTCATGGTAGACCAAGACCTATCGCTATTCCACGATACTG

CTCCCCTATTTTCGGTAACAGAATTTGGCGCGCCAATGCCTGATTCAGATCAACTCTGGCAGGCGCAGACAGCTCCAGAGTGGTCGGAGATGTTCAACCAGGTCCATG

AATTCTCGAATGGATACTCCTCAGTAGGCTCTGGAGCAAGACCACCGAGTCTCAGAGATCTCTTCCGCTACTTCCTAGATGACGAGATAGTTGTCCAGGATGTTCAGGA

GATTCATCTGACCCCGATGCACCTGCGACTACTACTGCACCCTCTGCAGACCCTTGTGTGCCAATATTGCCAACTCCTCAGTTGCTTTTCCGACAGTGTAGCCTCTCGA

TCGCGGAATCGGGCATTGACAGCTGCTTCGACTCGCGTACGCCTAGAAGAGGTGCAGGCGTTGTTGGGTCGGTGGTTCGACTTGGCGCAACGGTACATGAAGGCGA

ACCCAGTATGTCCGATGATGCAGGCAAATCTCGTCATGTTCCATCTTATCAGCATGAATGCTGTGTCAAATTTCCCAGAGATTGAGCGCCTCGCCCGACGGGAGGGTG

TGGACGGCTCGTATCAACAGATGCTGTGGATGCACAAGAAGTGCTTGTCGGATGTGCAGGAGGCCATCACTCACGCTGGCCAAGTTCTTCGCCTCATTCGTGAGATG

CCACGAGGCATTCGCCCGCCGTGGTGGGCTGGTGCTGTCTATCGGGCAGGGTTGGTCTTCTGGGCTGACTCACTCGCTCACAACGAGTCCTTGAGTCCCAACCATCA

AGGGACATATCAGCCATCAAATACTGCTTTCTCTGTGGATCGCCTCTCTGCTGACCACCCAACCATACTGCGGTACATGTCACGGAGGGAGGGTATTCCCACCTTGAC

CAAGCGCGATGGTAGCCCTGTTCCACTGGATAACGGCTTTGCCGTCCTGACGCATTGTATCGATGTCCTTGACGAGGGTGTCGCCACCAGGTTTTCCGATGGCATTCG

TAGTAAACTCGAGAAACTTGCACGCGGTTAA

>*Alternaria metachromatica* (J4E83_001950[a])

ATGGTCTTTTGCACATACTGTGGACAGTCGTTCACCAGAGACGAGCATCTCGAACGACATATCTTGACTCGTATGTTGACCCCGCGTCTTTCGCTCTTTCAATCTTCCTC

ACACCAGCTACCATCCAATCTTTCTTCTGGGATATCTGCTAATCACTGTGGATGAGCAGACACCAACGTCAAGCCTTTCAAATGCTTCACATGTCATATGAGCTTTGCTC

GACGGTATGTGGCACCCTTCCTTGCTCTTTCTCATCCTGTGGCATCGACCGTTCGGAACAAAGTACTGACAGCCTCTTCAGAGATCTTTTGCAAAGACACTACACCGTA

CACGGCCGCAACCAGAACAACGAAGAGGGACTCCCCCCACAAGGCATAATACCAAAGTCCGCCGGCCGCACACCCATCGCCTGCAGTAATTGTGCCAAGACAAAGAC

CAAGTGTGACAAGAAGTTTCCGTGCACCAGGTGCGCTCAGAGGAATCTGAAATGCACATTGCGTCCTACAAGGCGGGCATCGAAAGGTGTCCAGCGGGTTGGCGGTC

CTCCTGAGGCTGGGAGCGGGGACTCGAGCGAGAGTGGCAGCAACGCGGATAACCACAATACATCCGGCAATGTCTCTCCAAACCACGATAGCCAACCGAAACAAGC

GATGTCGAAGTCCTCTCCTGCTCAGATCAACCAGACTGCCCATATTCCGGACAGCCAACCACCGTCGCAAAGACAATCGCAGTCGCCCTCGGCCCCTCATTCACGAAA

CCCTTCCATCTCACAACCGGTACAGCCACCGACACACATAAATCCCGAGGAGAAGTCGTTGCATCTTCATATGCCGATCTCCGATACGCCGCCCTTTTTCGACCAGTC

CCCTTCCAACGGACTTGCTCAATCATCGTCGCTTCTTTCGCCACTCCCCACCCCGGTGAACGGCATGAATGGATTCGTCACCTCCACACCTATGTCAGGATATGACGA

CTTCGTCAGAACGGTACGGGACCAATCCGATAATGAGAGCCCTCAATTCATGATGGACCCTTGGAACGGCATGCCCATGGATACAACCTTTGATCCCATGCGGATAGA

CCCCGCGTTGATGATGAGCATGAGTATGGACATGAGCATGGGACCACCTCAGGATGGCATCCTTGGCATGATACCAGAAATGTCGCCTGCTCAAACATTTGGACAGCC

TGTCCAGACACCTCGCATGGACGAGTCCTTTTCGGACCTGCAAATTGGTAGTTCGGCTTCGATGTTCTACCCCTCCAACAGGCACTCTTCGATAGCAGATGCCGGCAT

ACCTGATCTTGGTGCCATCATCGCGGCTCAAGATGGCTGGACTGTTTTCCGTTGCACTCCTTCTATTGCTTCAAGCTCGTGTCCACGCACAGCGAGACTCAACCTCGA

GCGGTTAGAGCTGAGCCTCAAGAATCACGAGGGCTGGGCTAACTGGTCCCCATCGTGGGATGAATCCGACTTTCAACAAGGCGGGCAAATCGCGGTATCGAAGTTGC

AGGAAGCCACGCGAGACAAACTTCTCGCCATCACCCAAAGCTTCCTCCACAAGGCTTTGGACATCCACAAAGACGGAAGCAGCTCGTCCAGCAGTGGCGAGTCTCCA

GGATCCACGGCCTCTCACTCCAACTTCGTTCTTCTTCCACCTGCTCGCGTCCTGGAGTACTTTCTCAAGTCATACGCCAACTCTTTCGAACGCTATTATCCCACCTCCG

CCAGAGGTGTGCTCGATACGAATGAACTAATGCAAAACTACAACGACAAAGCATCGAGCTTGCTCATTCTCATGATGATCGCGCAGGGCGCCATGACCATTCCATCCA

TAGAGGCCAGGTGGCTAACTGGTGGCTTCACCGAAGCCTGCCGGATATCGCTCTTCGACCTTATTGAGAAGAACATCATCATGGCTGGTGATCCTATCGTTCTGCGTG

CAGCCCTCCTCTTCACTGTCCAGGCGGCGTGGAGTGGCGACAAATGGCAGATGGACATCGCTATGGGCCAGCGTGGCATGTATTTCTCCATGTTGCGACATTCCGGT

ATTCTCGATGTGCGACCGCCGGCAACGCCTCATATCAACGGAAATATTTCGACGGATGCGTTGTGGAAGGACTGGCTTCAGCAAGAAAGCCGCAGTCGGTAAGTGTT

GAAAACTTCGGTACCGTCACGTGAACGCACTTACTAATATTGGCGCAGCCTCATCTACTCCTGGGTCATGGTAGACCAAGACTTATCGCTCTTCCACGATACCGCTCCA

CTGTTCTCAGTAACAGAGTTTGGCGCGCCGATGCCTGATTCGGATCGACTTTGGCAAGCGCAGACCGCCTCAGAGTGGTCTGAGATGTTCAACCAAGTCCATGAGTTC

TCGAACGGGTACTCTTCAGTAGGCTCTGGGGCCAGACCACCCAGTCTCAGAGATCTGTTCCGTTACTTCCTAGATGACGAAATCGTCGTACAGGATGTACAGGAGATT

CATCTGACACCTATGCACCTGCGTCTGCTCCTCCACCCCCTGCAGACTCTGGTCTGCCAGTATTGCCAGCTTCTCAGCTGCTTTTCCGATAGCGTCGCTTCACGATCG

CGGAATCGCGCACTGACAGCAGCGTCGACCCGCGTGCGTCTCGAGGAGGTGCAGGCGTTGCTAGGGCGTTGGTTCGATTTGGCGCAACGGTACATGAAGTCCAACC

CAGTGTGTCCGATGATGCAGGCGAACCTTGTCATGTTCCATCTTATCAGCTTGAACGCTGTGTCCAACTTTCCAGAGATAGAGCGCCTTGCGCGTCGAGAGGGTGTGG

ACGGCTCATACCAGCAGATGCTGTGGATGCACAAGAAGTGTCTGTCCGATGTGCAGGAGGCCATCACCCACGCCGGTCAGGTCCTTCGACTCATCCGTGAGATGCCA

AGAGGTATCCGGCCGCCATGGTGGGCTGGCGCCGTCTACCGTGCGGGTTTGGTCTTCTGGACTGATTCGCTGGCACATGGCGAGTCCTTGAGTCCCAACCACCAAG

GATCGTATCAGCCATCGAACTCCGCGTTCTCTGTGGATCGCCTTCCAGCTGACCATCCTACCATACTGAGGTACATGTCTCGTAGGGAGGGCATACCCACTCTGACCA

AGCGCGACGGTACACACGTCCCTTTGGATAACGGCTTCGCGATCCTGTCGCATTGTATCGACGTCCTTGATGAGGGTGTTGCCACCAGGTTCTCCGATGGCATTCGTA

GTAAACTCGAGAAACTCGCGCGAGGATAA

>*Alternaria novae-zelandiae* (J4E88_001357[a])

ATGGTCTTTTGCACATACTGTGGACAGTCGTTCACCAGAGACGAGCATCTCGAACGACATATCTTGACTCGTATGTTGACCCCGCGTCTTTCGCTCTTTCAATCTTCCTC

ACACCAGCTACCATCCAATCTTTCTTCTGGGATATCTGCTAATCACTGTGGATGAGCAGACACCAACGTCAAGCCTTTCAAATGCTTCACATGTCATATGAGCTTTGCTC

GACGGTATGTGGCACCCTTCCTTGCTCTTTCTCATCCTGTGGCATCGACCGTTCGGAACAAAGTACTGACAGCCTCTTCAGAGATCTTTTGCAAAGACACTACACCGTA

CACGGCCGCAACCAGAACAACGAAGAGGGACTCCCCCCACAAGGCATAATACCAAAGTCCGCCGGCCGCACACCCATCGCCTGCAGTAATTGTGCCAAGACAAAGAC

CAAGTGTGACAAGAAGTTTCCGTGCACCAGGTGCGCTCAGAGGAATTTGAAATGCACATTGCGTCCTACAAGGCGGGCATCGAAAGGTGTCCAGCGGGTTGGCGGTC

CTCCTGAGGCTGGGAGCGGGGACTCGAGCGAGAGTGGCAGCAACGCGGATAACCACAATACATCCGGCAATGTCTCTCCAAACCACGATAGCCAACCGAAACAAGC

GATGTCGAAGTCCTCTCCTGCTCAGATCAACCAGACTGCCCATATTCCGGACAGCCAACCACCGTCGCAAAGACAATCGCAGTCGCCCTCGGCCCCTCATTCACGAAA

CCCTTCCATCTCACAACCGGTACAGCCACCGACACACATAAATCCCGAGGAGAAGTCGTTGCATCTTCATATGCCGATCTCCGATACGCCGCCCTTTTTCGACCAGTC

CCCTTCCAACGGACTTGCTCAATCATCGTCGCTTCTTTCGCCACTCCCCACCCCGGTGAACGGCATGAATGGATTCGTCACCTCCACACCTATGTCAGGATATGACGA

CTTCGTCAGAACGGTACGGGACCAATCCGATAATGAGAGCCCTCAATTCATGATGGACCCTTGGAACGGCATGCCCATGGATACAACCTTTGATCCCATGCGGATAGA

CCCCGCGTTGATGATGAGCATGAGTATGGACATGAGCATGGGACCACCTCAGGATGGCATCCTTGGCATGATACCAGAAATGTCGCCTGCTCAAACATTTGGACAGCC

TGTCCAGACACCTCGCATGGACGAGTCCTTTTCGGACCTGCAAATTGGTAGTTCGGCTTCGATGTTCTACCCCTCCAACAGGCACTCTTCGATAGCAGATGCCGGCAT

ACCTGATCTTGGTGCCATCATCGCGGCTCAAGATGGCTGGACTGTTTTCCGTTGCACTCCTTCTATTGCTTCAAGCTCGTGTCCACGCACAGCGAGACTCAACCTCGA

GCGGTTAGAGCTGAGCCTCAAGAATCACGAGGGCTGGGCTAACTGGTCCCCATCGTGGGATGAATCCGACTTTCAACAAGGCGGGCAAATCGCGGTATCGAAGTTGC

AGGAAGCCACGCGAGACAAACTTCTCGCCATCACCCAAAGTTTCCTCCACAAGGCTTTGGACATCCACAAAGACGGAAGCAGCTCGTCCAGCAGTGGCGAGTCTCCA

GGATCCACGGCCTCTCACTCCAACTTCGTTCTTCTTCCACCTGCTCGCGTCCTGGAGTACTTTCTCAAGTCATACGCCAACTCTTTCGAACGCTATTATCCCACCTCCG

CCAGAGGTGTGCTCGATACGAATGAACTAATGCAAAACTACAACGACAAAGCGTCGAGCTTGCTCATTCTCATGATGATCGCGCAGGGCGCCATGACCATTCCATCCA

TAGAGGCCAGGTGGCTAACTGGTGGCTTCACCGAAGCCTGCCGGATATCGCTCTTCGACCTTATTGAGAAGAACATCATCATGGCTGGTGATCCTATCGTTCTGCGTG

CAGCCCTCCTCTTCACTGTCCAGGCGGCGTGGAGTGGCGACAAATGGCAGATGGACATCGCTATGGGCCAGCGTGGCATGTATTTCTCCATGTTGCGACATTCCGGT

ATTCTCGATGTGCGACCGCCGGCAACGCCTCATATCAACGGAAATATTTCGACGGATGCGTTGTGGAAGGACTGGCTTCAGCAAGAAAGCCGCAGTCGGTAAGTGTT

GAAAACTTCGGTACCGTCACGTGAACGCACTTACTAATATTGGCGCAGCCTCATCTACTCCTGGGTCATGGTAGACCAAGACTTATCGCTCTTCCACGATACCGCTCCA

CTGTTCTCAGTAACAGAGTTTGGCGCGCCGATGCCTGATTCGGATCGACTTTGGCAAGCGCAGACCGCCTCAGAGTGGTCTGAGATGTTCAACCAAGTCCATGAGTTC

TCGAACGGGTACTCTTCAGTAGGCTCTGGGGCCAGACCACCCAGTCTCAGAGACCTGTTCCGTTACTTCCTAGATGACGAAATCGTCGTACAGGATGTACAGGAGATT

CATCTGACACCTATGCACCTGCGTCTGCTCCTCCACCCCCTGCAGACTCTGGTCTGCCAGTATTGCCAGCTTCTCAGCTGCTTTTCCGATAGCGTCGCTTCACGATCG

CGGAATCGCGCACTGACAGCAGCGTCGACCCGCGTGCGTCTCGAGGAGGTGCAGGCGTTGCTAGGGCGTTGGTTCGATTTGGCGCAACGGTACATGAAGTCCAACC

CAGTGTGTCCGATGATGCAGGCGAACCTTGTCATGTTCCATCTTATCAGCTTGAACGCTGTGTCCAACTTTCCAGAGATAGAGCGCCTTGCGCGTCGAGAGGGTGTGG

ACGGCTCATACCAGCAGATGCTGTGGATGCACAAGAAGTGTCTGTCCGATGTGCAGGAGGCCATCACCCACGCCGGTCAGGTCCTTCGACTCATCCGTGAGATGCCA

AGAGGTATCCGGCCGCCATGGTGGGCTGGCGCCGTCTACCGTGCGGGTTTGGTCTTCTGGACTGATTCGCTGGCACATGGCGAGTCCTTGAGTCCCAACCACCAAG

GATCGTATCAGCCATCGAACTCCGCGTTCTCTGTGGATCGCCTTCCAGCTGACCATCCTACCATACTGAGGTACATGTCTCGTAGGGAGGGCATACCCACTCTGACCA

AGCGCGACGGTGCACACGTCCCCTTGGATAACGGCTTCGCGATCCTGTCGCATTGTATCGACGTCCTTGATGAGGGTGTTGCCACCAGGTTCTCCGATGGCATTCGT

AGTAAACTCGAGAAACTCGCGCGAGGATAA

>*Alternaria panax* (G6011_11168[a])

ATGGTCTTCTGCACCTATTGTGGACAGTCGTTCACCAGAGACGAGCATCTCGAACGACATATTCTAACTCGTAAGTGGACCCTTCGCCTGTCTTCACGGATTGGTTTTC

GCAGTGCCAAGTACGAACCGATCGTTCCGTAGCATCTCCGCTGACTGCTATTGCCAAACAGACACCAATGTCAAGCCATTCAAGTGCTTTACATGCCATATGAGCTTTG

CTCGACGGTATGTCTTATCCTTTGTGTGCTTGCTAGCTCTTTCTCGTCTGGTGGTATTTCTCGTACGGAACGAAACGCTGACGAGCCTTGTAGAGACCTTTTGCAAAGA

CATTATACCGTACACGGCCGCAACCAGAACAACGAAGAGGGCCTCCCGCCACAAGGGATAATACCCAAGTCCGCTGGCCGCACACCCATCGCCTGCAGTAATTGTGC

CAAGACAAAGACGAAATGCGACAAGAAGTTTCCGTGCACTCGGTGCGCTCAGAGGAACCTGAAGTGTACTTTGCGCCCCACAAGGCGAGCATCGAAAGGTGTCCAGC

GAGTAGGAGGTCCTCCTGAAACAGGGAGCGGCGACTCGAGCGAGAGCGGTAGCAACGCGGATAACCAGAATACATCTGGGAATGTCTCTCCAAGCCACAATGGCCA

ACAGAAACAAGCAACCGCACAGTCGTCTCCTTCTCAGATCCAGCAGCCTGGGCAGGTATCAGATAGCCAACCCCCGTCACAACGACAATCGCAATCGCCCTCAGCAC

CCCACTCGCGAAATCCCTCCATCTCACATCCAGTACCACCACCACCGCACATAAATCCCGAGGAAAAGTCGCTCCACATTCATATGCCTATCTCGAATACACCCCCATT

CTTCGATCAGTCTCCTTCCAACGGACTGGCTCATTCGTCATCACTACTCTCACCACTCCCTACCCCAGTTACAGGCATGAACAGCTTTGTCTCCCCAACACCCATGTCC

GGATACGACGACTTTGTAAGGACAGTGCGTGATCAATCAGACAATGAGAGCCCCCAGTTCGTGATGGATCCTTGGCACTCGATGCCCATGGACACGACCTTTGATCCC

ATGCGGATTGATCCTGCGTTGATGATGACCATGGGTATGGATATGACGATGGGACCGCCTCAGGACGGCATCCTCAGTATGATACCCGAAATGTCACCTGCGCAGAC

CTTTGGACAGCCTATCCAGACTCCTCGCATGGTAGACGAATCATTCTCAGACCTGCAGATCGGCAGCTCGGCTTCAGTGTTTTACTCCTCTAATAGACACTCTTCAATA

GCAGACGCCGGCATCCCTGACCTTGGTGCCATCATCGCAGCTCAAGATGGCTGGACGGTTTTCCGCTGCACTCCTTCTATCGCTTCGAGTTCATGTCCGCGAACGGC

AAGGCTGAATCTTGAGCGACTAGAGTTAAGCTTGAAGAACCACGAAGGATGGGCTAACTGGACCCCATCGTGGGACGAATCCGACTACCAACAAGGCGGGCAAGTAG

TTGTGTCAAAATTACACGAAGCCACACGAGATAAACTCCTCGCCATCACTCAAAGCTTCCTCCACAAGGCTCTGGATATCCACAAGGACGGTAATAGCTCATCCAGTAG

TGGCGAGTCTCCCGGCTCCACGGCTTCTCATTCCAACTTTGTTCTTCTTCCGCCTGCTCGCGTCCTGGAGTACTTCCTCAAGTCGTATGCCAACTCTTTTGAACGGTAT

TACCCTACCTCCGCCAGAGGTGTTCTCGACACCAACGAGTTGATGCAAAACTATAATGACAAGGCATCAAGCTTGCTCATCCTCATGATGATCGCACAGGGTGCCATG

ACTATTCCGTCCATTGAAGCGAGATGGCTGACAGGTGGCTTTACCGAAGCATGTCGGATATCGCTATTCGACCTCATCGAGAAGAACATCATCATGGCTGGGGATCCT

ATCGTCTTGCGCGCAGCCCTTCTCTTCACTGTCCAAGCGGCGTGGAGTGGCGACAAGTGGCAGATGGACATCGCAATGGGTCAACGTGGCATGTATTTTTCTATGTTG

CGACACTCCGGTATTCTTGATGTTCGGCCGCCTACTACACCTCACATGAATGGAAACACGTCGACGGATGTGTTGTGGAAGGACTGGCTTCAGCAAGAAAGTCGCAGT

CGGTAAGTGTTGGAGAAGAAAAAGTCGGTACAGTCGCGTGCACACTTACTAATATCGTGCAGCCTCATCTACTCTTGGGTAATGGTAGACCAAGACCTGTCGCTGTTC

CACGATACCGCTCCTCTGTTCTCGGTGACAGAATTCGGCGCGCCGATGCCTGATTCGGATCAACTCTGGCAGGCGCAGACCGCCTCAGAATGGTCGGAGATGTTCAA

TCAAGTCCATGAGTTTTCGAATGGATACTCCTCAGTGGGCTCTGGAGCAAGGCCACCAAGTCTCAGAGATCTTTTCCGTTACTTCCTAGACGACGAGATAGTCGTTCAG

GATGTACAGGAGATTCATCTCACCCCAATGCACTTGCGGCTGCTGCTGCATCCTTTGCAGACCCTTGTGTGCCAATATTGCCAACTTCTCAGTTGCTTTTCCGACAGCG

TAGCCCCTCGATCGCGAAATCGGGCACTGACGGCCGCTTCGACTCGCGTACGCCTAGAAGAGGTGCAAGCGTTACTGGGACGGTGGTTCAACTTGGCGCAACGGTA

CATGAAAGCGAACCCAGTGTGTCCAATGATGCAGGCAAATCTCGTCATGTTTCATCTTATCAGCATGAATGCTGTGTCAAATTTCCCAGAGATCGAGCGTCTCGCGCGG

CGAGAGGGTGTGGATGGCTCGTACCAGCAGATGCTGTGGATACACAAGAAGTGCTTGTCCGATGTGCAGGAGGCCATCACCCACGCTGGCCAAGTCATTCGACTCAT

CCGTGAGATGCCCCGAGGAATTCGCCCGCCGTGGTGGGCTGGTGCTGTCTACCGGGCAGGGCTAGTCTTCTGGACTGATTCGCTCTCTCATAACGAGGCTTTGAGCC

CGAGCCATCAAAATACATACCAGCAGTCATCGAATACTGCTTTCGCGGTGGACCGCCTTCCTGCTGACCACCCAACCATACTGAGGTATATGTCACGGAGGGAGGGTA

TCCCCACTTTGACTAAGCGCGACGGTACATCTGTGCCACTCGACAACGGCTTTGCTGTCCTCTCACATTGTATTGATGTCCTTGACGAGGGTGTCGCCACCAGATTTTC

CGATGGCATCCGTAGTAAACTCGAGAAACTCGCACGGGGTTGA

>*Alternaria porri* (APR_PT04911[c])

ATGGTCTTCTGCACATATTGTGGACAGTCGTTCACCAGGGACGAGCATCTCGAACGACATATCCTGACTCGTAAGTTGGCTCTTGCTCCGTCCTTGAGGATTGATTTCC

GCCGTGACGAGTACAGTCCGGTCGTTCCGTGGGACCTTCGCTAACCGCCGTTGCCAAACAGACACAAATGTCAAGCCCTTCAAGTGCTTTACATGCCATATGAGCTTT

GCTCGACGGTAAGTCTTACCCTTCATGTGCTCCTTGCTCTTTCTCCGCCGGTGGCAACCCTTGTAGCGAACGAAACGCTGACGATCCTCCCAGAGATCTTTTGCAAAG

ACATTACACCGTCCACGGCCGCAACCAGAACAACGAGGAAGGTCTACCACCACAAGGGATCATACCTAAGTCCGCTGGCCGCACACCCATCGCCTGCAGCAATTGTG

CAAAGACGAAGACGAAATGCGACAAGAAGTTTCCTTGCACCCGGTGCGCTCAGAGAAACCTGAAATGTACTCTGCGTCCTACACGTCGAGCATCGAAAGGTGTCCAGC

GAGTGGGGGGTCCTCCTGAAACAGGGAGTGGTGATTCGAGCGAGAGTGGTAGCAATGCGGACAACCAGATCACATCCGGCAATGTCTCTCCAAACCACGATGGCCAA

TCGAAACAAGCAACCGCACAGTCATCTCCATCTCAGATTCAGCAGACTGCCTTGATGACTGACAGCCAACCTCCGTCACAACGACAATCGCAATCGCCTTCAGCGTCC

CACTCGCGGAATCCCTCCATCTCGCATCCAGTGCAACCACCACCGCACATGAATCCCGAGGAGAAGTCGCTCCACATACACATGCCGATCTCAAATACTCCCCCGTTC

TTCGACCAGTCACCGTCCAATGGCCTAGCTCATTCCTCGTCATTACTTTCGCCTCTTCCCACTCCGGTTACGGGCATGAATAGCTTTGTCTCCTCGACACCAATGTCTG

GATACGACGATTTCGTCAGGACAGTGCGCGATCAGCCGGAGAATGAGAGCCCGCAGTTCATGATGGATCCTTGGCATTCGATGCCTATGGATACGAGCTTCGATCCTA

TGCGGATTGACCCCGCTCTGATGATGACCATGGGCATGGATATGACTATGGGACCACCCCAAGACGGCATTCTCAGTATGATACCCGAAATGTCACCTGCGCAGACAT

TCGGGCAGCCTGTCCAGACTCCTCGCATGGTGGACGAATCTTTCTCGGACTTGCAGATTGGCAGTTCGGCTTCGATGTTCTACCCTTCCAACAGACATTCTTCAATAGC

GGACGCCGGCATCCCTGATCTCGGAGCCATTATCGCGGCTCAAGACGGCTGGACGGTTTTTCGTTGCACTCCTTCTATTGCTTCCAGCTCATGTCCGCGGACAGCGA

GGCTGAATCTTGAGCGACTAGAGTTGAGCCTGAAGAACCATGAAGGGTGGGCCAACTGGTCACCATCCTGGGATGAATCCGATTTTCAACAAGGCGGGCAAATAGCC

GTGTCGAAATTACAGGAAGCCACACGAGACAAACTCCTCGCCATCACACAGAGCTTTCTTCACAAAGCTCTAGACATCCACAAAGACGGAAGCAGCACGTCCAGTAGT

GGTGAATCTCCAGGGTCCACGGCTTCGCATTCCAATTTCGTCCTTCTTCCGCCTGCTCGCGTCCTAGAGTACTTCCTCAAGTCGTACGCCAACTCTTTCGAACGCTACT

ACCCTACCTCCGCCAGAGGAGTCCTCGACGCCAACGAGTTGATGCAGAACTACAATGACAAGGCATCAAGCTTGCTCATTCTCATGATGATTGCGCAGGGCGCCATGA

CCATTCCGTCCATAGAAGCGAGATGGCTGACTGGTGGGTTTACCGAGGCATGTCGGATATCACTGTTCGACCTGATCGAGAAGAACATCATCATGGCTGGCGATCCTA

TCGTCCTGAGAGCGGCCCTTCTCTTCACTGTCCAAGCGGCGTGGAGTGGCGACAAGTGGCAGATGGACATCGCCATGGGTCAGCGCGGCATGTATTTTTCCATGTTA

CGGCACTCCGGTATTCTTGATGTTCGGCCACCTACCACGCCTCACATGAACGGAAACACGTCGACGGATGTGTTGTGGAAGGACTGGCTTCAGCAAGAAAGCCGCAG

CCGGTAAGTGTTGAAAACCTCGGTACAGTCACGTGCGCACACTTACTAATATGATGTAGCCTCATCTACTCTTGGGTCATGGTGGACCAAGACCTATCGCTCTTCCACG

ATACTGCCCCACTGTTCTCGGTAACAGAATTCGGCGCGCCAATGCCGGATTCAGATCAGCTCTGGCAGGCACAGACCGCTTCCGAGTGGTCGGAGATGTTCAACCAA

GTGCATGAATTCTCCAATGGGTACTCCTCAGTAGGCTCTGGAGCAAGACCACCGAGTCTCAGAGATCTCTTCCGTTACTTCCTAGATGACGAGATAGTCGTCCAGGAT

GTCCAGGAGATTCACTTGACCCCGATGCACCTGCGGCTGCTGCTCCACCCTCTGCAGACCCTTGTGTGCCAGTATTGTCAACTCCTCAGCTGCTTTTCAGATAGCGTA

GCCTCTCGATCGAGGAATCGGGCACTGACTGCCGCTTCGACTCGCGTGCGCCTAGAAGAGGTACAGGCCTTGCTGGGGCGGTGGTTCGACTTGGCGCAACGATACA

TGAAGGCGAACCCAGTATGTCCGATGATGCAGGCAAACCTCGTCATGTTCCATCTGATCAGCATGAATGCTGTGTCGAACTTCCCAGAGATTGAGCGCCTCGCGCGAC

GAGAGGGCGTGGACGGCTCGTACCAGCAGATGCTGTGGATGCACAAGAAGTGCTTGTCGGATGTGCAGGAGGCCATAACACATGCTGGTCAAGTTCTTCGCCTCATC

CGTGAGATGCCACGAGGCATCCGCCCGCCGTGGTGGGCTGGTGCTGTCTATCGTGCAGGATTGGTGTTCTGGACTGATTCGCTCGCTCATAACGAGTCTCTGAGCCC

CAACCACCAAGGAACATATCAGCAGCCATCGAATACTGCTTTCTCTGTGGATCGCCTTTCTTCTGACCACCCTACCATACTGAGGTACATGTCACGAAGAGAGGGTATC

CCCACCTTGACCAAGCGTGACGGAAACCCCGTTCCACTGGATAACGGCTTCGCTATCCTGTCGCATTGTATCGATGTCCTTGACGAGGGCGTCGCCACCAGATTTTCC

GATGGCATTCGTAGTAAACTCGAGAAACTTGCGCGTGGTTGA

>*Alternaria postmessia* (NA)

ATGGTCTTCTGCACATATTGTGGACAGTCGTTCACCAGGGACGAACATCTCGAACGACATATCCTAACTCGTAAGTCAACTCTTGCTCTGTCCATGAGGGTGGATTTCC

GCAATGTCGAGTTCAATCCGATCGTTCCGTAGCATCTCCACTGACCGTCAATGCCAAACAGACACCAATGTCAAGCCATTCAAGTGCTTTACATGTCATATGAGCTTTG

CTCGACGGTAAGTCCCACCCTGCGTGCGCTCCTTCCTCTTCCTGTTTGGTCATAATTTCCGTACCGAACGAAACGCTGACAATCTTTCCAGAGATCTTTTGCAAAGACA

TTACACCGTACACGGCCGCAACCAGAACAACGAAGAAGGCCTCCCACCGCAAGGCATCATACCTAAGTCCGCCGGCCGCACACCCATCGCCTGCAGTAATTGTGCAA

AGACGAAGACGAAATGCGACAAGAAGTTTCCTTGCACCCGGTGCGCTCAAAGGAACCTGAAGTGTACTCTACGTCCTACACGGCGAGCATCGAAAGGTGTCCAACGA

GTAGGAGGTCCTCCTGAAACAGGCAGTGGTGATTCTAGCGAGAGTGGTAGCAACGCGGATAACCAGAACACATCCGGCAATGTCTCTCCAAACCACGATGGCCAGTC

GAAACAAGCAACCGCTCAGTCGTCTCCATCTCAAATTCAGCAGCCTAGCCAGATTTCTGACAGCCAACCTCCATCGCAACGGCAATCGCAATCTCCCTCAGCGCCCCA

CTCGCGGAATCCTTCCATCTCACATCCAGTGCAACCACCACCGCACATGAACCCCGAGGAGAAGTCGCTCCACATACACATGCCTATCTCGAATACTCCCCCGTTCTT

CGATCAGTCACCCTCTAACGGACTGTCTCATTCCTCATCATTACTTTCACCTCTTCCCACACCAGTTACGGGCATGAATAGTTTTGTTTCTTCGACACCCATGTCTGGGT

ACGACGACTTCGTCAGGACAGTGCGCGATCAACCGGAAAATGAGAGCCCGCAATTCATGATGGATCCTTGGCACTCGATGCCCATGGATACGAGTTACGATCCTATGC

GGATTGACCCCGCTCTGATGATGACCATGGGCATGGACATGACGATGGGACCGCCCCAAGACGGTATCCTTAGTATGATACCCGAAATGTCACCTGCGCAAACATTCG

GACAGCCTGTTCAGACTCCTCGTATGGTGGACGAATCGTTCTCGGACTTGCAAATCGGCAGTTCGGCTTCGATGTTCTACCCCTCTAACAGACATTCCTCAATAGCGGA

CGCCGGCATCCCTGATCTTGGCGCCATTATCGCAGCCCAAGACGGCTGGACGGTCTTCCGTTGCACTCCTTCTATCGCTTCGAGCTCATGTCCGCGGACCGCAAGGC

TGAATCTTGAGAGATTAGAGTTGAGCCTGAAGAACCATGAGGGCTGGGCTAACTGGACCCCATCGTGGGACGAATCTGATTTCCAACAAGGCGGACAGATCGCCGTG

TCAAAACTCCAGGAAGCCACACGAGACAAACTTCTCGCGATCACACAGAGCTTCCTTCACAAGGCTCTAGATATCCACAAAGATGGGAGCAGCTCGTCTAGTAGTGGC

GAGTCTCCAGGATCTACGGCTTCACATTCCAACTTCGTTCTTCTTCCGCCTGCTCGTGTCCTGGAGTACTTCCTCAAGTCGTACGCCAACTCTTTCGAACGCTATTATCC

TACCTCGGCCAGAGGAGTCCTCGACACCAACGAGTTGATGCAGAACTACAATGACAAAGCTTCGAGCTTGCTCATTCTCATGATGATTGCGCAGGGCGCTATGACCAT

TCCGTCCATAGAAGCGAGATGGCTGACTGGCGGCTTTACTGAGGCATGTCGGATATCCCTGTTCGACCTCATCGAGAAGAACATCATTATGGCTGGCGATCCTATTGT

CCTGAGGGCGGCCCTTCTCTTCACTGTTCAAGCGGCGTGGAGTGGCGACAAGTGGCAGATGGACATCGCCATGGGTCAGCGCGGCATGTATTTTTCCATGTTACGAC

ATTCCGGTATTCTCGATGTTCGACCGCCTACCACGCCTCACATGAATGGAAACACTTCGACGGATGTGTTGTGGAAGGACTGGCTTCAGCAAGAAAGTCGCAGCCGGT

AAGTGTTGAAAACCTCGGTACTGTCACGTGCGCGCACTTACTAATATCGTGTAGCCTCATCTACTCTTGGGTCATGGTAGACCAAGACCTATCGCTATTCCACGATACC

GCTCCCCTGTTTTCGGTAACAGAATTTGGCGCGCCAATGCCTGATTCAGATCAACTCTGGCAGGCGCAGACAGCTCCAGAGTGGTCGGAGATGTTCAACCAGGTCCAT

GAATTCTCGAATGGATACTCCTCAGTAGGCTCTGGAGCAAGACCACCGAGTCTCAGAGATCTCTTCCGCTACTTCCTAGATGACGAGATAGTCGTCCAGGATGTTCAG

GAGATTCATCTGACCCCGATGCACCTGCGACTACTACTGCACCCTCTGCAGACCCTTGTGTGCCAATATTGCCAACTCCTCAGTTGCTTCTCCGACAGTGTAGCCTCTC

GATCGCGGAATCGGGCATTGACAGCTGCTTCGACTCGCGTACGCCTAGAAGAGGTACAGGCGTTATTGGGGCGGTGGTTCGACTTGGCGCAACGGTACATGAAGGC

GAACCCAGTATGTCCGATGATGCAAGCAAATCTCGTCATGTTCCATCTTATCAGCATGAATGCTGTGTCAAATTTCCCAGAGATTGAGCGCCTCGCCCGACGGGAGGG

TGTGGACGGCTCGTATCAACAGATGCTGTGGATGCACAAGAAGTGCTTGTCGGATGTGCAGGAGGCCATCACTCACGCTGGCCAAGTTCTTCGCCTCATTCGTGAGAT

GCCACGAGGCATTCGCCCGCCGTGGTGGGCTGGTGCTGTCTATCGGGCAGGGTTGGTCTTCTGGGCTGACTCGCTCGCTCACAACGAGTCCTTGAGTCCCAACCAT

CAAGGGACATATCAGCCATCTAATACTGCTTTCTCTGTGGATCGCCTCTCTGCTGACCACCCAACCATACTGAGGTACATGTCACGGAGGGAGGGTATTCCCACCTTG

ACCAAGCGCGATGGTAGCCCTGTTCCACTGGATAACGGCTTTGCCGTCCTGTCGCATTGTATCGATGTCCTTGACGAGGGTGTCGCCACCAGGTTTTCCGATGGCATT

CGTAGTAAACTCGAGAAACTTGCGCGCGGTTAA

>*Alternaria rosae* (XM_046164914[a])

ATGGTCTTTTGCACATACTGTGGACAGTCGTTCACCAGAGACGAGCACCTCGAACGACATATCTTGACTCGTACGTCGTCCCCTCATCCTTCGCTCCTTCGTTCTTCCT

TGTGCCAGCTACTATCCAATCGTCTCCTGAGATGTCTGCTAATCACTTTAGTTGAGCAGACACCAACGTCAAGCCATTCAAATGCTTCACATGTCACATGAGCTTTGCTC

GACGGTACGTGGCACCCTTCGCCTGCTTCCTTGCTCTTTCTGTTCCCGTGGTATCGATTGGTCCAAACAAAGCGCTGACAGACTCTTCAGGGATCTTTTGCAAAGACAC

TACACCGTACACGGCCGCAACCAGAATAACGAAGAGGGTCTCCCACCGCAAGGGATAATACCCAAATCCGCCGGCCGCACACCCATAGCCTGCAGCAATTGTGCAAA

GACAAAGACCAAGTGCGACAAGAAATTTCCGTGCACCCGGTGCGCCCAGAGGAATCTGAAATGCACGTTACGTCCTACAAGGCGTGCGTCGAAAGGTGTCCAACGGG

TGGGGGGTCCCCCAGAAGCTGGGAGCGGAGACTCGAGCGAGAGCGGCAGCAACGCGGATGTGCACAACACATCCGGCAATGTTTCTCCAAACCACGATAGCCAGCC

AAAACCAGCCATGTCGAAATCCTCTCCTTCTCAGATCCATCAGGCTTCCCATGTTCCGGACAGCCAGCCACCGTCGCAAAGACAATCGCAGTCGCCCTCAGCCCCTCA

TTCACGAAACCCTTCCATCTCACAACCGGTACAGCCACCGACACACATAAATCCCGAAGAGAAGTCGTTGCATCTTCATATGCCAATCTCTGATACACCGCCCTTTTTC

GATCAATCTCCTTCCAACGGGCTCGCTCAATCATCGTCGCTTCTTTCGCCACTCCCTACGCCGGTGACCGGCATGAACGGATTTGTCACCTCTACGCCAATGTCAGGA

TACGATGACTTCGTCAGAACAGTACGGGACCAATCCGACAACGAGAGCCCTCAATTCATGATGGACCCTTGGAATGGCATGCCCATGGATACTACCTTCGATCCCATG

CGGATAGACCCCGCATTGATGATGAGCATGAGTATGGACATGAGTATGGGACCGCCTCAGGATGGCATCCTTGGTATGATACCAGAAATGTCGCCTGCTCAAACATTT

GGACAGCCTGTCCAGACGCCTCGCATGGACGAGTCCTTCTCGGACCTGCAAATCGGTAGTTCGGCGTCGATGTTCTACCCCTCAAACAGACACTCTTCCATAGCGGAT

GCCGGCATTCCTGATCTTGGTGCTATCATCGCGGCTCAAGATGGCTGGACTGTTTTCCGTTGCACTCCTTCCATCGCCTCAAGCTCGTGTCCTCGAACAGCGAGACTC

AACCTCGAGCGGTTGGAGTTGAGCCTCAAGAATCACGAGGGTTGGGCTAACTGGTCCCCATCGTGGGATGAATCCGACTTTCAACAAGGCGGACAAATCGCCGTATC

GAAGTTACAGGAAGCAACGCGAGACAAACTCCTCGCCATCACCCAAAGCTTTCTCCACAAGGCTTTGGACATCCACAAAGACGGAAGTAGCTCATCCAGCAGTGGCGA

GTCTCCGGGATCCACGGCCTCTCACTCAAACTTCGTTCTTCTTCCACCTGCTCGCGTCCTGGAGTACTTTCTCAAATCATATGCCAACTCTTTCGAACGCTACTATCCCA

CTTCCGCTAGAGGTGTCCTCGATACGAATGAGCTGATGCAAAACTACAACGACAAAGCATCGAGCTTGCTCATTCTCATGATGATTGCGCAGGGCGCCATGACCATTC

CGTCCATAGAGGCCAGGTGGCTAACTGGTGGCTTCACCGAAGCCTGCCGGATATCGCTCTTCGACCTGATTGAGAAGAACATCATAATGGCTGGTGATCCTATCGTTT

TGCGTGCAGCCCTTCTCTTCACTGTTCAGGCGGCGTGGAGTGGCGACAAATGGCAGATGGACATCGCCATGGGCCAGCGTGGCATGTATTTCTCCATGTTGCGACAT

TCCGGTATTCTCGATGTGCGACCACCAGCAACGCCTCATGTGAACGGAAATATTTCGACGGATGCGTTGTGGAAGGACTGGCTTCAGCAAGAAAGCCGCAGTCGGTA

AGTGTCGAAAACTTCGGTACCGTCACGTGAACGCACTTACTAATATTGTCGCAGCCTCATCTACTCTTGGGTCATGGTAGACCAAGACTTGTCGCTCTTCCACGATACC

GCTCCTCTGTTCTCAGTAACGGAATTTGGCGCGCCGATGCCAGATTCCGATCGACTTTGGCAGGCGCAGACCGCCTCGGAATGGTCTGAGATGTTCAACCAAGTCCAT

GAGTTCTCGAACGGGTACTCTTCGGTAGGCTCTGGAGCCAGACCACCCAGTCTCCGAGATCTCTTCCGTTACTTCCTAGACGACGAGATAGTCGTCCAGGATGTGCAG

GAGATCCATCTGACCCCTATGCACCTGCGTCTGCTCCTCCACCCTCTGCAGACTTTGGTCTGTCAGTATTGCCAACTCCTCAGCTGCTTTTCAGATAGCGTCGCTTCAC

GTTCGCGAAATCGTGCACTCACGGCAGCGTCGACTCGCGTGCGCCTCGAGGAGGTGCAGGCGTTACTTGGGCGTTGGTTCGATTTGGCGCAACGGTACATGAAGTC

CAACCCAGTGTGTCCGATGATGCAGGCCAACCTTGTAATGTTCCACCTTATCAGCCTGAACGCAGTGTCCAACTTCCCAGAGATAGAGCGCCTAGCGCGTCGAGAGG

GCGTGGACGGCTCCTACCAGCAGATGCTGTGGATGCACAAGAAGTGTTTGTCCGATGTGCAGGAAGCCATCACCCACGCCGGTCAGGTCCTTCGACTTATCCGTGAG

ATGCCGCGAGGGATCAGGCCGCCGTGGTGGGCTGGCGCTGTCTACCGCGCAGGGTTGGTCTTCTGGACGGATTCGCTGGCCCATGGCGAGTCCTTGAGTCCCAACC

ACCAAGGATCGTATCAGCCATCGAACTCCGCTTTCTCTGTGGATCGCCTTCCTTCTGACCACCCTACCATACTGAGGTACATGTCTCGCAGGGAGGGCATACCCACTC

TGACCAAGCGCGACGGTACACACGTTCCCTTGGATAACGGCTTCGCGATCCTGTCGCATTGTATCGACGTCCTCGATGAGGGTGTCGCCACCAGGTTCTCGGATGGT

ATTCGTAGTAAACTCGAGAAACTCGCGCGAGGATAA

>*Alternaria solani* (ASL_PT00349[b])

ATGGTCTTCTGCACATATTGTGGACAGTCGTTCACCAGGGACGAGCATCTCGAACGACATATCCTGACTCGTAAGTTGGCTCTTGCTCCGTCCTTGAGGATTGATTTCC

GCCGTGACGAGTACAGTCCGGTCGTTCCGTGGGACCTTCGCTAACCGCCGTTGCCAAACAGACACAAATGTCAAGCCCTTCAAGTGCTTTACATGCCATATGAGCTTT

GCCCGACGGTAAGTCTTACCCTTCATGTGCTCCTTGCTCTTTCTCCGCCGGTGGCAACCCTTGTAGCGAACGAAACGCTGACGATCCTCCCAGAGATCTTTTGCAAAG

ACATTACACCGTCCACGGCCGCAACCAGAACAACGAGGAAGGTCTACCACCACAAGGGATCATACCTAAGTCCGCTGGCCGCACACCCATCGCCTGCAGCAATTGTG

CAAAGACGAAGACGAAATGCGACAAGAAGTTTCCTTGCACCCGGTGCGCTCAGAGGAACCTGAAATGTACTCTGCGTCCTACACGTCGAGCATCGAAAGGTGTCCAG

CGAGTGGGGGGTCCTCCTGAAACAGGGAGTGGTGATTCGAGCGAGAGTGGTAGCAATGCGGACAACCAGATCACATCCGGCAATGTCTCTCCAAACCACGATGGCC

AATCGAAACAAGCAACCGCACAGTCATCTCCATCTCAGATTCAGCAGACTGCCTTGATGACTGACAGCCAACCTCCGTCACAACGACAATCGCAATCGCCTTCAGCGT

CCCACTCGCGGAATCCCTCCATCTCGCATCCAGTGCAACCACCACCGCACATGAATCCCGAGGAGAAGTCGCTCCACATACACATGCCGATCTCAAATACTCCCCCGT

TCTTCGACCAGTCACCGTCCAATGGCCTAGCTCATTCCTCGTCATTACTTTCGCCTCTTCCCACTCCGGTTACGGGCATGAATAGCTTTGTCTCCTCGACACCAATGTC

TGGATACGACGATTTCGTCAGGACAGTGCGCGATCAGCCGGAGAATGAGAGCCCGCAGTTCATGATGGATCCTTGGCATTCGATGCCTATGGATACGAGCTTCGATC

CTATGCGGATTGACCCCGCTCTGATGATGACCATGGGCATGGATATGACTATGGGACCACCCCAAGACGGCATTCTCAGTATGATACCCGAAATGTCACCTGCGCAGA

CATTCGGGCAGCCTGTCCAGACTCCTCGCATGGTGGACGAATCTTTCTCGGACTTGCAGATTGGCAGTTCGGCTTCGATGTTCTACCCTTCCAACAGACATTCTTCAAT

AGCGGACGCCGGCATCCCTGATCTCGGAGCCATTATCGCGGCTCAAGACGGCTGGACGGTTTTTCGTTGCACTCCTTCTATTGCTTCCAGCTCATGTCCGCGGACAG

CGAGGCTGAATCTTGAGCGACTAGAGTTGAGCCTGAAGAACCATGAAGGGTGGGCCAACTGGTCACCATCCTGGGATGAATCCGATTTTCAACAAGGCGGGCAAATA

GCCGTGTCGAAATTACAGGAAGCCACACGAGACAAACTCCTCGCCATCACACAGAGCTTTCTTCACAAAGCTCTAGATATCCACAAAGACGGAAGCAGCACGTCCAGT

AGTGGTGAATCTCCAGGGTCCACGGCTTCGCATTCCAACTTCGTCCTTCTTCCGCCTGCTCGCGTCCTGGAGTACTTCCTCAAGTCGTACGCCAACTCTTTCGAACGC

TACTACCCTACCTCCGCCAGAGGAGTCCTCGACGCCAACGAGTTGATGCAGAACTACAATGACAAGGCATCAAGCTTGCTCATTCTCATGATGATTGCGCAGGGCGCC

ATGACCATTCCGTCCATAGAAGCGAGATGGCTGACTGGTGGGTTTACCGAGGCATGTCGGATATCACTGTTCGACCTGATCGAGAAGAACATCATCATGGCTGGCGAT

CCTATCGTCCTGAGAGCGGCCCTTCTCTTCACTGTCCAAGCGGCGTGGAGTGGCGACAAGTGGCAGATGGACATCGCCATGGGTCAGCGCGGCATGTATTTTTCCAT

GTTACGGCACTCCGGTATTCTTGATGTTCGGCCACCTACCACGCCTCACATGAACGGAAACACGTCGACGGATGTGTTGTGGAAGGACTGGCTTCAGCAAGAAAGCC

GCAGCCGGTAAGTGTTGAAAACCTCGGTACAGTCACGTGCGCACACTTACTAATATGATGTAGCCTCATCTACTCTTGGGTCATGGTGGACCAAGACCTATCGCTCTTC

CACGATACTGCCCCGCTGTTCTCGGTAACAGAATTCGGCGCGCCAATGCCGGATTCAGATCAGCTCTGGCAGGCACAGACCGCTTCAGAGTGGTCGGAGATGTTCAA

CCAAGTGCATGAATTCTCCAATGGGTACTCCTCAGTAGGCTCTGGAGCAAGACCACCGAGTCTCAGAGATCTCTTCCGTTACTTCCTAGATGACGAGATAGTCGTCCA

GGATGTCCAGGAGATTCACTTGACCCCGATGCACCTGCGGCTGCTGCTCCACCCTCTGCAGACCCTTGTGTGCCAGTATTGTCAACTCCTCAGCTGCTTTTCAGATAG

CGTAGCCTCTCGATCGAGGAATCGGGCACTGACTGCCGCTTCGACTCGCGTGCGCCTAGAAGAGGTACAGGCCCTGCTGGGGCGGTGGTTCGACTTGGCGCAACGA

TACATGAAGGCGAACCCAGTATGTCCGATGATGCAGGCAAACCTCGTCATGTTCCATCTGATCAGCATGAATGCTGTGTCGAACTTCCCAGAGATTGAGCGCCTCGCG

CGACGAGAGGGCGTGGACGGCTCGTACCAGCAGATGCTGTGGATGCACAAGAAGTGCTTGTCGGATGTGCAGGAGGCCATAACACATGCTGGTCAAGTTCTTCGCCT

CATCCGTGAGATGCCACGAGGCATCCGCCCGCCGTGGTGGGCTGGTGCTGTCTATCGTGCAGGATTGGTGTTCTGGACTGATTCGCTCGCTCATAACGAGTCTCTGA

GCCCCAACCACCAAGGAACATATCAGCAGCCATCGAATACTGCTTTCTCTGTGGATCGCCTTTCTTCTGACCACCCTACCATACTGAGGTACATGTCACGAAGAGAGG

GTATCCCCACCTTGACCAAGCGTGACGGAAACCCCGTTCCACTGGATAACGGCTTCGCTATCCTGTCGCATTGTATCGATGTCCTTGACGAGGGCGTCGCCACCAGAT

TTTCCGATGGCATTCGTAGTAAACTCGAGAAACTTGCGCGTGGTTGA

>*Alternaria tagetica* (ATT_PT04326[b])

ATGGTCTTCTGCACATATTGTGGACAGTCGTTCACCAGGGACGAACATCTCGAACGACATATCCTAACTCGTAAGTTGGCTCTTGCTCCGTCTTTGAGGGTGGATTTCC

GCAGTGACGAGTACAGTCCGGTCGTTCCGTGGGATCTTCGCTAACCGCCATTGCCAAACAGACACAAATGTCAAGCCCTTCAAGTGCTTTACATGCCATATGAGTTTC

GCTCGACGGTCAGTCTTACCCATCGTGTGCTTTTTGCTCTTTCTCGGCCGGTGGCAACCCTTGTAGCGAACGAAACGCTGACGATCCTCCCAGAGATCTTTTGCAAAG

ACATTATACTGTCCACGGCCGCAACCAGAACAACGAGGAAGGTCTGCCACCACAAGGGATCATACCTAAATCCGCTGGCCGCACACCCATCGCCTGCAGTAATTGTGC

AAAGACGAAGACGAAATGCGACAAGAAGTTTCCTTGCACCCGGTGCGCTCAGAGGAACCTGAAATGTACTCTGCGTCCTACACGTCGAGCATCGAAAGGTGTCCAGC

GAGTGGGGGGTCCTCCTGAAACAGGGAGTGGTGATTCGAGCGAGAGTGGTAGCAATGCGGACAACCAGATCACATCCGGCAATGTCTCTCCAAACCACGATGGCCAA

TCGAAACAAGCAACCGCACAGTCATCGCCATCTCAGATTCAGCAGACTGTCTTGATGACTGACAGCCAACCTCCGTCACAACGACAATCACAATCGCCTTCAGCGCCC

CACTCGCGGAATCCTTCCATCTCGCATCCAGTGCAACCACCACCGCACATGAATCCCGAGGAGAAGTCGCTCCACATCCATATGCCGATTTCAAATACTCCCCCATTCT

TCGACCAGTCACCGTCCAATGGCCTAGCTCATTCCTCGTCGTTACTTTCGCCTCTTCCCACACCAGTTACGGGCATGAATAGCTTCGTCTCCTCGACACCCATGTCTGG

ATACGACGATTTCGTCAGGACAGTACGCGATCAGTCGGAGAATGAGAGCCCGCAATTCATGATGGACCCTTGGCATTCGATGCCTATGGACACGAGCTTCGATCCTAT

GCGGATTGACCCCGCTCTGATGATGACCATGGGCATGGATATGACTATGGGACCACCCCAAGACGGCATTCTTAGTATGATACCAGAGATGTCACCTGCGCAGACATT

CGGACAGCCTGTCCAGACTCCTCGCATGGTGGAAGAATCATTCTCGGACTTGCAGATTGGCAGCTCGGCTTCGATGTTCTACCCTTCCAACAGACATTCTTCGATAGC

GGACGCCGGCATCCCTGATCTTGGAGCCATTATCGCGGCTCAAGACGGCTGGACTGTTTTCCGTTGCACTCCTTCTATTGCTTCCAGCTCATGTCCGCGGACTGCAAG

GCTGAATCTTGAGCGACTAGAGCTGAGCCTGAAGAACCATGAGGGTTGGGCCAACTGGGCTCCATCCTGGGATGAATCCGATTTCCAACAAGGCGGGCAAATAGCCG

TGTCGAAATTACAGGAAGCCACACGAGACAAACTCCTCGCCATCACACAGAGCTTTCTTCACAAAGCTCTAGACATCCACAAAGACGGAAGCAGCACGTCCAGTAGTG

GTGAATCTCCAGGTTCCACAGCTTCGCATTCCAACTTCGTCCTTCTTCCGCCTGCTCGCGTCCTGGAGTACTTCCTCAAGTCGTATGCCAACTCTTTCGAACGCTATTAT

CCTACCTCCGCCAGAGGAGTCCTTGACGCCAACGAGTTGATGCAGAACTACAATGACAAGGCATCAAGCTTGCTCATTCTCATGATGATTGCGCAGGGCGCCATGACC

ATTCCGTCCATAGAAGCGAGATGGCTGACCGGTGGATTTACCGAGGCATGTCGGATATCGCTGTTCGACCTGATCGAGAAGAACATCATCATGGCTGGCGATCCTATC

GTCCTGAGAGCGGCCCTTCTCTTCACTGTCCAAGCGGCGTGGAGCGGCGACAAGTGGCAGATGGACATCGCCATGGGTCAGCGCGGCATGTATTTTTCCATGTTACG

GCACTCCGGTATTCTTGATGTTCGGCCACCTACCACGCCTCACATGAACGGAAACACGTCGACGGATGTGCTGTGGAAGGACTGGCTTCAGCAAGAAAGCCGCAGCC

GGTAAGTGCTGAAAACCTCGGTACAGTCACGTGCGCACACTTACTAATATGATGTAGCCTCATCTACTCTTGGGTCATGGTAGACCAGGACCTATCGCTCTTCCATGAT

ACCGCCCCGCTGTTCTCGGTAACAGAATTCGGCGCGCCAATGCCTGATTCAGATCAGCTCTGGCAGGCGCAGACCGCTTCAGAGTGGTCGGAGATGTTCAACCAAGT

GCATGAATTCTCCAATGGGTACTCCTCAGTAGGCTCTGGAGCAAGACCACCGAGTCTCAGAGATCTCTTCCGTTACTTCCTAGATGACGAGATAGTCGTTCAGGATGTC

CAGGAGATTCATCTGACCCCGATGCACCTGCGGCTGCTGCTCCACCCTCTGCAGACCCTTGTGTGCCAGTATTGTCAACTCCTCAGCTGCTTTTCAGATAGCGTAGCC

TCTCGATCGAGAAATCGGGCACTGACTGCCGCTTCGACTCGGGTGCGCCTAGAAGAGGTACAGGCCTTGCTGGGGCGGTGGTTCGACTTGGCGCAAAGATACATGAA

GGCGAACCCAGTATGTCCGATGATGCAGGCAAACCTCGTCATGTTCCATCTGATCAGCATGAATGCTGTGTCGAACTTCCCAGAGATCGAGCGCCTCGCGCGACGAG

AAGGCGTGGACGGCTCGTACCAGCAGATGTTGTGGATGCACAAGAAGTGCTTGTCAGATGTGCAGGAGGCCATAACCCATGCTGGTCAAGTTCTTCGCCTCATTCGT

GACATGCCACGAGGCATCCGCCCGCCGTGGTGGGCTGGTGCTGTCTATCGGGCAGGGTTGGTGTTCTGGACTGATTCGCTCGCCCATAACGAGTCTCTGAGCCCCA

ACCACCAAGGAACATATCAGCAGCCATCTAACACTGCTTTCTCTGTGGATCGCCTTTCTGCTGACCACCCTACCATACTGAGGTACATGTCACGAAGGGAGGGTATCC

CCACCTTGACCAAGCGTGACGGAAACCCCGTTCCACTGGATAACGGCTTCGCTATCCTGTCGCATTGTATCGACGTCCTTGACGAGGGCGTCGCCACCAGATTTTCCG

ATGGCATTCGTAGTAAACTCGAGAAACTTGCGCGTGGTTGA

>*Alternaria tangelonis* (NA)

ATGGTCTTCTGCACATATTGTGGACAGTCGTTCACCAGGGACGAACATCTCGAACGACATATCCTAACTCGTAAGTCAACTCTTGCTCTGTCCATGAGGGTGGATTTCC

GCAATGTCGAGTTCAATCCGATCGTTCCGTAGCATCTCCGCTGACCGTCAATGCCAAACAGACACCAATGTCAAGCCATTCAAGTGCTTTACATGTCATATGAGCTTTG

CTCGACGGTAAGTCCCACCCTGCGTGTGCTCCTTCCCCTTCCTGTTTGGTCATAATTTCCGTACCGAACGAAACGCTGACAATCTTTCCAGAGATCTTTTGCAAAGACA

TTACACCGTACACGGCCGCAACCAGAACAACGAAGAAGGCCTCCCACCGCAAGGCATCATACCTAAGTCCGCCGGCCGCACACCCATCGCCTGCAGTAATTGTGCAA

AGACGAAGACGAAATGCGACAAGAAGTTTCCTTGCACCCGGTGCGCTCAAAGGAACCTGAAGTGTACTCTACGTCCTACACGGCGAGCATCGAAAGGTGTCCAACGA

GTAGGAGGTCCTCCTGAAACAGGCAGTGGTGATTCTAGCGAGAGTGGTAGCAACGCGGATAACCAGAACACATCCGGCAATGTCTCTCCAAACCACGATGGCCAGTC

GAAACAAGCAACCGCTCAGTCGTCTCCATCTCAAATTCAGCAGCCTAGCCAGATTTCTGACAGCCAACCTCCATCGCAACGGCAATCGCAATCTCCCTCAGCGCCCCA

CTCGCGGAATCCTTCCATCTCACATCCAGTGCAACCACCACCGCACATGAACCCCGAGGAGAAGTCGCTCCACATACACATGCCCATCTCGAATACTCCCCCGTTCTT

CGATCAGTCACCCTCTAACGGACTGTCTCATTCCTCATCATTACTTTCACCTCTTCCCACACCAGTTACGGGCATGAATAGTTTTGTTTCTTCGACACCCATGTCTGGGT

ACGACGACTTCGTCAGAACAGTGCGCGATCAACCGGAAAATGAGAGTCCGCAGTTCATGATGGATCCTTGGCACTCGATGCCCATGGATACGAGTTACGATCCTATGC

GGATTGACCCCGCTCTGATGATGACCATGGGCATGGACATGACGATGGGACCGCCCCAAGACGGTATCCTTAGTATGATACCCGAAATGTCACCTGCGCAAACATTCG

GACAGCCTGTTCAGACTCCTCGTATGGTGGACGAATCGTTCTCGGACTTGCAAATCGGCAGTTCGGCTTCGATGTTCTACCCCTCTAACAGACATTCCTCAATAGCGGA

CGCCGGCATCCCTGATCTTGGCGCCATTATCGCAGCCCAAGACGGCTGGACGGTCTTCCGTTGCACTCCTTCTATCGCTTCAAGCTCATGTCCGCGGACCGCAAGGC

TGAATCTTGAGAGATTAGAGTTGAGCCTGAAGAACCATGAGGGCTGGGCTAACTGGACCCCATCGTGGGACGAATCTGATTTCCAACAAGGCGGACAGATCGCCGTG

TCAAAACTCCAGGAAGCCACACGAGACAAACTCCTCGCGATCACACAGAGCTTCCTTCACAAGGCTCTAGATATCCACAAAGATGGAAGCAGCTCGTCTAGTAGTGGC

GAGTCTCCAGGATCTACGGCTTCACATTCCAACTTCGTTCTTCTTCCGCCTGCTCGTGTCCTGGAGTACTTCCTCAAGTCGTACGCCAACTCTTTCGAACGCTATTATCC

TACCTCGGCCAGAGGAGTCCTCGACACCAACGAGTTGATGCAGAACTACAATGACAAAGCTTCGAGCTTGCTCATTCTCATGATGATTGCGCAGGGCGCTATGACCAT

TCCGTCCATAGAAGCGAGATGGCTGACTGGCGGCTTTACTGAGGCATGTCGGATATCCCTGTTCGATCTCATCGAGAAGAACATCATTATGGCTGGCGATCCTATTGT

CCTGAGGGCGGCCCTTCTCTTCACTGTTCAAGCGGCGTGGAGTGGCGACAAGTGGCAGATGGACATCGCCATGGGTCAGCGCGGCATGTATTTTTCCATGTTACGAC

ATTCCGGTATTCTCGATGTTCGACCGCCTACCACGCCTCACATGAATGGAAATACTTCGACGGATGTGTTGTGGAAGGACTGGCTTCAGCAAGAAAGCCGCAGCCGGT

AAGTGTTGAAAACCTCGGTACAGTCACGTGCGCGCACTTACTAATATCGTGTAGCCTCATCTACTCTTGGGTCATGGTAGACCAAGACCTATCGCTATTCCACGATACC

GCTCCCCTGTTTTCGGTAACAGAATTTGGCGCGCCAATGCCTGATTCAGATCAACTCTGGCAGGCGCAGACAGCTCCAGAGTGGTCGGAGATGTTCAACCAGGTCCAT

GAATTCTCGAATGGATACTCCTCAGTAGGCTCTGGAGCAAGACCACCGAGTCTCAGAGATCTCTTCCGCTACTTCCTAGACGACGAAATAGTCGTCCAGGATGTTCAA

GAGATTCATCTGACCCCAATGCACCTGCGACTACTACTGCACCCTCTGCAGACCCTTGTGTGCCAATATTGCCAACTCCTCAGTTGCTTTTCCGACAGTGTAGCCTCTC

GATCGCGGAATCGGGCATTGACAGCTGCTTCGACTCGCGTACGCCTAGAAGAGGTGCAGGCGTTATTGGGGCGGTGGTTCGACTTGGCGCAACGGTACATGAAGGC

GAACCCAGTATGTCCGATGATGCAAGCAAATCTCGTCATGTTCCATCTTATCAGCATGAATGCTGTGTCAAATTTCCCAGAGATTGAGCGCCTCGCCCGACGGGAGGG

TGTGGACGGCTCGTATCAACAGATGTTGTGGATGCACAAGAAGTGCTTGTCGGATGTGCAGGAGGCCATCACTCACGCTGGCCAAGTTCTTCGCCTCATTCGTGAGAT

GCCACGAGGCATTCGCCCGCCGTGGTGGGCTGGTGCTGTCTATCGGGCAGGGTTGGTCTTCTGGGCTGACTCGCTCGCTCACAACGAGTCCTTGAGTCCCAACCAT

CAAAAGACATATCAGCCATCTAATACTGCTTTCTCTGTGGATCGCCTCTCTGCTGACCACCCAACCATACTGAGGTACATGTCACGGAGGGAGGGTATTCCCACCTTGA

CCAAGCGCGATGGTAGCCCTGTTCCACTGGATAACGGCTTTGCCGTCCTGTCGCATTGTATCGATGTCCTTGACGAGGGTGTCGCCACCAGGTTTTCCGATGGCATTC

GTAGTAAACTCGAGAAACTTGCGCGCGGTTAA

>*Alternaria tenuissima* (NA)

ATGGTCTTCTGCACATATTGTGGACAGTCGTTCACCAGGGACGAACATCTCGAACGACATATCCTAACTCGTAAGTCAACTCTTGCTCTGTCCATGAGGGTGGATTTCC

GCAATGTCGAGTTCAATCCGATCGTTCCGTAGCATCTCCGCTGACCGTCAATGCCAAACAGACACCAATGTCAAGCCATTCAAGTGCTTTACATGTCATATGAGCTTTG

CTCGACGGTAAGTCCCACCCTGCGTGCGCTCCTTCCTCTTCCTGTTTGATCATAATTTCCGTACCGAACGAAACGCTGACAATCTTTCCAGAGATCTTTTGCAAAGACAT

TACACCGTACACGGCCGCAACCAGAACAACGAAGAAGGCCTCCCACCGCAAGGCATCATACCTAAGTCCGCCGGCCGCACACCCATCGCCTGCAGTAATTGTGCAAA

GACGAAGACGAAATGCGACAAGAAGTTTCCTTGCACCCGGTGCGCTCAAAGGAACCTGAAGTGTACTCTACGTCCTACACGGCGAGCATCGAAAGGTGTCCAACGAG

TAGGAGGTCCTCCTGAAACAGGCAGTGGTGATTCTAGCGAGAGTGGTAGCAACGCGGATAACCAGAACACATCCGGCAATGTCTCTCCAAACCACGATGGCCAGTCG

AAACAAGCAACCGCTCAGTCGTCTCCATCTCAAATTCAGCAGCCTAGCCAGATTTCTGACAGCCAACCTCCATCGCAACGGCAATCGCAATCTCCCTCAGCGCCCCAC

TCGCGGAATCCTTCCATCTCACATCCAGTGCAACCACCACCGCACATGAACCCCGAGGAGAAGTCGCTCCACATACACATGCCTATCTCGAATACTCCCCCGTTCTTC

GATCAGTCACCCTCTAACGGACTGTCTCATTCCTCATCATTACTTTCACCTCTTCCCACACCAGTTACGGGCATGAATAGTTTTGTTTCTTCGACACCCATGTCTGGGTA

CGACGACTTCGTCAGGACAGTGCGCGATCAACCGGAAAATGAGAGCCCGCAATTCATGATGGATCCTTGGCACTCGATGCCCATGGATACGAGTTACGATCCTATGC

GGATTGACCCCGCTCTGATGATGACCATGGGCATGGACATGACGATGGGACCGCCCCAAGACGGTATCCTTAGTATGATACCCGAAATGTCACCTGCGCAAACATTCG

GACAGCCTGTTCAGACTCCTCGTATGGTGGACGAATCGTTCTCGGACTTGCAAATCGGCAGTTCGGCTTCGATGTTCTACCCCTCTAACAGACATTCCTCAATAGCGGA

CGCCGGCATCCCTGATCTTGGCGCCATTATCGCAGCCCAAGACGGCTGGACGGTCTTCCGTTGCACTCCATCTATCGCTTCGAGCTCATGTCCGCGGACCGCAAGGC

TGAATCTTGAGAGATTAGAGTTGAGCCTGAAGAACCATGAGGGCTGGGCTAACTGGACCCCATCGTGGGACGAATCTGATTTCCAACAAGGCGGACAGATCGCCGTG

TCAAAACTCCAGGAAGCCACACGAGACAAACTCCTCGCGATCACACAGAGCTTCCTTCACAAGGCTCTAGATATCCACAAAGATGGAAGCAGCTCGTCTAGTAGTGGC

GAGTCTCCAGGATCTACGGCTTCACATTCCAACTTCGTTCTTCTTCCGCCTGCTCGTGTCCTGGAGTACTTCCTCAAGTCGTACGCCAACTCTTTCGAACGCTATTATCC

TACCTCGGCCAGAGGAGTCCTCGACACCAACGAGTTGATGCAGAACTACAATGACAAAGCTTCGAGCTTGCTCATTCTCATGATGATTGCGCAGGGCGCTATGACCAT

TCCGTCCATAGAAGCGAGATGGCTGACTGGCGGCTTTACTGAGGCATGTCGGATATCCCTGTTCGACCTCATCGAGAAGAACATCATTATGGCTGGCGATCCTATTGT

CCTGAGGGCGGCCCTTCTCTTCACTGTTCAAGCGGCGTGGAGTGGCGACAAGTGGCAGATGGACATCGCCATGGGTCAGCGCGGCATGTATTTTTCCATGTTACGAC

ATTCCGGTATTCTCGATGTTCGACCGCCTACCACGCCTCACATGAATGGAAACACTTCGACGGATGTGTTGTGGAAGGACTGGCTTCAGCAAGAAAGCCGCAGCCGGT

AAGTGTTGAAAACCTCGGTACAGTCACGTGCGCGCACTTACTAATATCGTGTAGCCTCATCTACTCTTGGGTCATGGTAGACCAAGACCTATCGCTATTCCACGATACC

GCTCCCCTGTTTTCGGTAACAGAATTTGGCGCGCCAATGCCTGATTCAGATCAACTCTGGCAGGCGCAGACAGCTCCAGAGTGGTCGGAGATGTTCAACCAGGTCCAT

GAATTCTCGAATGGATACTCCTCAGTAGGCTCTGGAGCAAGACCACCGAGTCTCAGAGATCTCTTCCGCTACTTCCTAGATGACGAGATAGTCGTCCAGGATGTTCAG

GAGATTCATCTGACCCCGATGCACCTGCGACTACTACTGCACCCTCTGCAGACCCTTGTGTGCCAATATTGCCAACTCCTCAGTTGCTTTTCCGACAGTGTAGCCTCTC

GATCGCGGAATCGGGCATTGACAGCTGCTTCGACTCGCGTACGCCTAGAAGAGGTGCAGGCGTTATTGGGGCGGTGGTTCGACTTGGCGCAACGGTACATGAAGGC

GAACCCAGTATGTCCGATGATGCAAGCAAATCTCGTCATGTTCCATCTTATCAGCATGAATGCTGTGTCAAATTTCCCAGAGATTGAGCGCCTCGCCCGACGGGAGGG

TGTGGACGGCTCGTATCAACAGATGCTGTGGATGCACAAGAAGTGCTTGTCGGACGTGCAGGAGGCCATCACTCACGCTGGCCAAGTTCTTCGCCTCATTCGTGAGA

TGCCACGAGGCATTCGCCCGCCGTGGTGGGCTGGTGCTGTCTATCGGGCAGGGTTGGTCTTCTGGGCTGACTCGCTCGCTCACAACGAGTCCTTGAGTCCCAACCAT

CAAGGGACATATCAGCCATCTAATACTGCTTTCTCTGTGGATCGCCTCTCTGCTGACCACCCAACCATACTGAGGTACATGTCACGGAGGGAGGGTATTCCCACCTTG

ACCAAGCGCGATGGTAGCCCTGTTCCACTGGATAACGGCTTTGCCGTCCTGTCGCATTGTATCGATGTCCTTGACGAGGGTGTCGCCACCAGGTTTTCCGATGGCATT

CGTAGTAAACTCGAGAAACTTGCGCGCGGTTAA

>*Alternaria tomatophila* (ATM_PT05196[b])

ATGGTCTTCTGCACATATTGTGGACAGTCGTTCACCAGGGACGAGCATCTCGAACGACATATCCTGACTCGTAAGTTGGCTCTTGCTCCGTCCTTGAGGATTGATTTCC

GCCGTGACGAGTACAGTCCGGTCGTTCCGTGGGACCTTCGCTAACCGCCGTTGCCAAACAGACACAAATGTCAAGCCCTTCAAGTGCTTTACATGCCATATGAGCTTT

GCTCGACGGTAAGTCTTACCCTTCATGTGCTCCTTGCTCTTTCTCCGCCGGTGGCAACCCTTGTAGCGAACGAAACGCTGACGATCCTCCCAGAGATCTTTTGCAAAG

ACATTACACCGTCCACGGCCGCAACCAGAACAACGAGGAAGGTCTACCACCACAAGGGATCATACCTAAGTCCGCTGGCCGCACACCCATCGCCTGCAGCAATTGTG

CAAAGACGAAGACGAAATGCGACAAGAAGTTTCCTTGCACCCGGTGCGCTCAGAGGAACCTGAAATGTACTCTGCGTCCTACACGTCGAGCATCGAAAGGTGTCCAG

CGAGTGGGGGGTCCTCCTGAAACAGGGAGTGGTGATTCGAGCGAGAGTGGTAGCAATGCGGACAACCAGATCACATCCGGCAATGTCTCTCCAAACCACGATGGCC

AATCGAAACAAGCAACCGCACAGTCATCTCCATCTCAGATTCAGCAGACTGCCTTGATGACTGACAGCCAGCCTCCGTCACAACGACAATCGCAATCGCCTTCAGCGT

CCCACTCGCGGAATCCCTCCATCTCGCATCCAGTGCAACCACCACCGCACATGAATCCCGAGGAGAAGTCGCTCCACATACACATGCCGATCTCAAATACTCCCCCGT

TCTTCGACCAGTCACCGTCCAATGGCCTAGCTCATTCCTCGTCATTACTTTCGCCTCTTCCCACTCCGGTTACGGGCATGAATAGCTTTGTCTCCTCGACACCAATGTC

TGGATACGACGATTTCGTCAGGACAGTGCGCGATCAGCCGGAGAATGAGAGCCCGCAGTTCATGATGGATCCTTGGCATTCGATGCCTATGGATACGAGCTTCGATC

CTATGCGGATTGACCCCGCTCTGATGATGACCATGGGCATGGATATGACTATGGGACCACCCCAAGACGGCATTCTCAGTATGATACCCGAAATGTCACCTGCGCAGA

CATTCGGGCAGCCTGTCCAGACTCCTCGCATGGTGGACGAATCTTTCTCGGACTTGCAGATTGGCAGTTCGGCTTCGATGTTCTACCCTTCCAACAGACATTCTTCAAT

AGCGGACGCCGGCATCCCTGATCTCGGAGCCATTATCGCGGCTCAAGACGGCTGGACGGTTTTTCGTTGCACTCCTTCTATTGCTTCCAGCTCATGTCCGCGGACAG

CGAGGCTGAATCTTGAGCGACTAGAGTTGAGCCTGAAGAACCATGAAGGGTGGGCCAACTGGTCACCATCCTGGGATGAATCCGATTTTCAACAAGGCGGGCAAATA

GCCGTGTCGAAATTACAGGAAGCCACACGAGACAAACTCCTCGCCATCACACAGAGCTTTCTTCACAAAGCTCTAGACATCCACAAAGACGGAAGCAGCACGTCCAGT

AGTGGTGAATCTCCAGGGTCCACGGCTTCGCATTCCAATTTCGTCCTTCTTCCGCCTGCTCGCGTCCTGGAGTACTTCCTCAAGTCGTACGCCAACTCTTTCGAACGCT

ACTACCCTACCTCCGCCAGAGGAGTCCTCGACGCCAACGAGTTGATGCAGAACTACAATGACAAGGCATCAAGCTTGCTCATTCTCATGATGATTGCGCAGGGCGCCA

TGACCATTCCGTCCATAGAAGCGAGATGGCTGACTGGTGGGTTTACCGAGGCATGTCGGATATCACTGTTCGACCTGATCGAGAAGAACATCATCATGGCTGGCGATC

CTATCGTCCTGAGAGCGGCGCTTCTCTTCACTGTCCAAGCGGCGTGGAGTGGCGACAAGTGGCAGATGGACATCGCCATGGGTCAGCGCGGCATGTATTTTTCCATG

TTACGGCACTCCGGTATTCTTGATGTTCGGCCACCTACCACGCCTCACATGAACGGAAACACGTCGACGGATGTGTTGTGGAAGGACTGGCTTCAGCAAGAAAGCCG

CAGCCGGTAAGTGTTGAAAACCTCGGTACAGTCACGTGCGCACACTTACTAATATGATGTAGCCTCATCTACTCTTGGGTCATGGTGGACCAAGACCTATCGCTCTTCC

ACGATACTGCCCCACTGTTCTCGGTAACAGAATTCGGCGCGCCAATGCCGGATTCAGATCAGCTCTGGCAGGCACAGACCGCTTCAGAGTGGTCGGAGATGTTCAAC

CAAGTGCATGAATTCTCCAATGGGTACTCCTCAGTAGGCTCTGGAGCAAGACCACCGAGTCTCAGAGATCTCTTCCGTTACTTCCTAGATGACGAGATAGTCGTCCAG

GATGTCCAGGAGATTCACTTGACCCCGATGCACCTGCGGCTGCTGCTCCACCCTCTGCAGACCCTTGTGTGCCAGTATTGTCAACTCCTCAGCTGCTTTTCAGATAGC

GTAGCCTCTCGATCGAGGAATCGGGCACTGACTGCCGCTTCGACTCGCGTGCGCCTAGAAGAGGTACAGGCCTTGCTGGGGCGGTGGTTCGACTTGGCGCAACGAT

ACATGAAGGCGAACCCAGTATGTCCGATGATGCAGGCAAACCTCGTCATGTTCCATCTGATCAGCATGAATGCTGTGTCGAACTTCCCAGAGATTGAGCGCCTCGCGC

GACGAGAGGGCGTGGACGGCTCGTACCAGCAGATGCTGTGGATGCACAAGAAGTGCTTGTCGGATGTGCAGGAGGCCATAACACATGCTGGTCAAGTTCTTCGCCTC

ATCCGTGAGATGCCACGAGGCATCCGCCCGCCGTGGTGGGCTGGTGCTGTCTATCGTGCAGGATTGGTGTTCTGGACTGATTCGCTCGCTCATAACGAGTCTCTGAG

CCCCAACCACCAAGGAACATATCAGCAGCCATCGAATACTGCTTTCTCTGTGGATCGCCTTTCTTCTGACCACCCTACCATACTGAGGTACATGTCACGAAGAGAGGGT

ATCCCCACCTTGACCAAGCGTGACGGAAACCCCGTTCCACTGGATAACGGCTTCGCTATCCTGTCGCATTGTATCGATGTCCTTGACGAGGGCGTCGCCACCAGATTT

TCCGATGGCATTCGTAGTAAACTCGAGAAACTTGCGCGTGGTTGA

>*Alternaria triticina* (NA)

ATGGTCTTTTGCACATACTGTGGACAGTCGTTCACCAGAGACGAGCATCTCGAACGACATATCTTGACTCGTATGCTGACCCCGCGTCTTTCGCTTTTTCATTCTTCCTC

GCACCAGCTACCATCCAATCTTTCTTCTGGGATATCTGCTAATCACTGTGGATGAGCAGACACCAACGTCAAGCCTTTCAAATGCTTCACATGTCATATGAGCTTTGCTC

GACGGTATGTGGCACCCTTCCTTGCTCTTTCTCATCCTGTGGCATCGACCGTTCGGAACAAAGTACTGACAGCCTCTTCAGAGATCTTTTGCAAAGACACTACACCGTA

CACGGCCGCAACCAGAACAACGAAGAGGGACTCCCCCCACAAGGCATAATACCAAAGTCCGCCGGCCGCACACCCATCGCCTGCAGTAATTGTGCGAAGACAAAGAC

CAAGTGTGACAAGAAGTTTCCGTGCACCAGGTGCGCTCAGAGGAATCTGAAATGCACATTGCGTCCTACAAGGCGGGCATCGAAAGGTGTCCAGCGGGTTGGCGGTC

CTCCTGAGGCTGGGAGCGGGGACTCTAGCGAGAGTGGCAGCAACGCGGATAACCACAATACATCCGGCAATGTCTCTCCAAACCACGATAGCCAACCGAAACAAGCG

ATGTCGAAGTCCTCTCCTGCTCAGATCAACCAGACTTCCCATATTCCGGACAGCCAACCACCGTCGCAAAGACAATCGCAGTCGCCCTCGGCCCCTCATTCACGAAAC

CCTTCCATCTCACAACCGGTACAGCCACCGACACACATAAATCCCGAGGAGAAGTCGTTGCATCTTCATATGCCGATCTCCGATACGCCGCCCTTTTTCGACCAGTCC

CCTTCCAACGGACTTGCTCAATCATCGTCGCTTCTTTCGCCACTCCCCACCCCGGTGAACGGCATGAATGGGTTCGTCACCTCCACACCTATGTCAGGATATGACGAC

TTCGTCAGAACGGTCCGGGACCAATCCGATAATGAGAGCCCTCAATTCATGATGGACCCTTGGAACGGCATGCCCATGGATACAACCTTTGATCCCATGCGGATAGAC

CCCGCGTTGATGATGAGCATGAGTATGGACATGAGCATGGGACCACCTCAGGATGGCATCCTTGGCATGATACCAGAAATGTCGCCTGCTCAAACATTTGGACAGCCT

GTCCAGACACCTCGCATGGACGAGTCCTTTTCGGACCTGCAAATTGGTAGTTCGGCTTCGATGTTCTACCCCTCCAACAGGCACTCTTCGATAGCAGATGCCGGCATA

CCTGATCTTGGTGCCATCATCGCGGCTCAAGATGGCTGGACTGTTTTCCGTTGCACTCCTTCTATTGCTTCAAGCTCGTGTCCACGCACAGCGAGACTCAACCTCGAG

CGGTTAGAGCTGAGCCTCAAGAATCACGAGGGCTGGGCTAATTGGTCCCCATCGTGGGATGAATCCGACTTTCAACAAGGCGGGCAAATCGCGGTATCGAAGTTGCA

GGAAGCCACGCGAGACAAACTTCTCGCCATCACCCAAAGCTTCCTCCACAAGGCTTTGGACATCCACAAAGACGGAAGCAGCTCGTCCAGCAGTGGCGAGTCTCCAG

GATCCACGGCCTCTCACTCCAACTTCGTTCTTCTTCCACCTGCTCGCGTCCTGGAGTACTTTCTCAAGTCATACGCCAACTCTTTCGAACGCTATTATCCCACCTCCGC

CAGAGGTGTACTCGATACGAATGAACTAATGCAAAACTACAACGACAAAGCATCGAGCTTGCTCATTCTCATGATGCTTGCGCAGGGCGCCATGACCATCCCATCCATA

GAGGCCAGGTGGCTAACTGGTGGCTTCACCGAAGCCTGCCGGATATCGCTCTTCGACCTTATCGAGAAGAACATCATCATGGCTGGTGATCCTATCGTTCTGCGTGCA

GCCCTCCTCTTCACTGTCCAGGCGGCGTGGAGTGGCGACAAATGGCAGATGGACATCGCTATGGGCCAGCGTGGCATGTATTTCTCCATGTTGCGACATTCCGGTAT

TCTCGATGTGCGACCGCCAGCAACGCCTCATATCAACGGAAATATTTCGACGGATGCGTTGTGGAAGGACTGGCTTCAGCAAGAAAGCCGCAGTCGGTAAGTGTTGAA

AACCTCGGTACCGTCACGTGAACGCACTTACTAATATTGGCGCAGCCTCATCTACTCCTGGGTCATGGTAGACCAAGACTTATCGCTCTTCCACGATACCGCTCCACTG

TTCTCAGTAACAGAGTTTGGCGCGCCGATGCCTGATTCGGATCGACTTTGGCAAGCGCAGACCGCCTCAGAGTGGTCTGAGATGTTCAACCAAGTCCATGAGTTCTCG

AATGGGTACTCTTCAGTAGGATCTGGGGCCAGACCACCTAGTCTCAGAGATCTCTTCCGTTACTTTCTAGATGACGAAATCGTCGTACAGGATGTACAGGAGATTCATC

TGACACCTATGCACCTGCGTCTGCTCCTCCACCCCCTGCAGACTCTGGTCTGCCAGTATTGCCAGCTTCTCAGCTGCTTTTCCGATAGCGTCGCTTCACGATCGCGGA

ACCGCGCACTGACAGCAGCGTCGACCCGCGTGCGTCTCGAGGAGGTGCAGGCGTTGCTAGGGCGTTGGTTCGATCTGGCGCAACGGTACATGAAGTCCAACCCAGT

GTGTCCGATGATGCAGGCCAACCTTGTAATGTTCCATCTTATCAGCTTGAACGCTGTGTCCAACTTTCCAGAGATAGAGCGCCTTGCGCGTCGAGAGGGCGTGGACGG

CTCATACCAGCAGATGCTGTGGATGCACAAGAAGTGTCTGTCCGATGTACAGGAGGCCATCACCCACGCCGGTCAGGTCCTTCGACTCATCCGTGAGATGCCAAGAG

GTATCCGGCCGCCATGGTGGGCTGGCGCCGTCTACCGTGCGGGTTTGGTCTTCTGGACTGATTCGCTGGCACATGGCGAGTCCTTGAGTCCCAACCACCAAGGATC

GTATCAGCCATCGAACTCCGCGTTCTCTGTGGATCGCCTTCCTGCTGACCACCCTACCATACTGAGGTATATGTCTCGCAGGGAGGGCATACCCACTCTGACCAAGCG

CGACGGTACACACGTCCCCTTGGATAACGGCTTCGCGATCCTATCGCATTGTATCGACGTCCTTGATGAGGGTGTTGCCACCAGGTTCTCCGATGGCATTCGTAGTAA

ACTCGAGAAACTCGCGCGAGGATAA

>*Alternaria triticimaculans* (NA)

ATGGTCTTTTGCACATACTGTGGACAGTCGTTCACCAGAGACGAGCATCTCGAACGACATATCTTGACTCGTATGTTGACCCCGCGTCTTTCGCTCTTTCAATCTTCCTC

GCACCAGCTACCATCCAATATTTCTTCTGGGATATCTGCTAATCACTGTGGATGAGCAGACACCAACGTCAAGCCTTTCAAATGCTTCACATGTCATATGAGCTTTGCTC

GACGGTATGTGGCACCCTTCCTTGCTCTTTCTCATCCTGTGGCATCGACCGTTCGGAACAAAGTACTGACAGCCTCTTCAGAGATCTTTTGCAAAGACACTACACCGTA

CACGGCCGCAACCAGAACAACGAAGAGGGACTCCCCCCACAAGGCATAATACCAAAGTCCGCCGGCCGCACACCCATCGCCTGCAGTAATTGTGCGAAGACAAAGAC

CAAGTGTGACAAGAAGTTTCCGTGCACCAGGTGCGCTCAGAGGAATCTGAAATGCACATTGCGTCCTACAAGGCGGGCATCGAAAGGTGTCCAGCGGGTTGGCGGTC

CTCCTGAGGCTGGGAGCGGGGACTCGAGCGAGAGTGGTAGCAACGCGGATAACCACAATACATCCGGCAATGTCTCTCCAAACCACGATAGCCAACCGAAACAAGC

GATGTCGAAGTCCTCTCCTGCTCAGATCAACCAGACTTCCCATATTCCGGACAGCCAACCACCGTCGCAAAGACAATCGCAGTCGCCCTCGGCCCCTCATTCACGAAA

CCCTTCCATCTCACAACCGGTACAGCCACCGACACACATAAATCCCGAGGAGAAGTCGTTGCATCTTCATATGCCGATCTCCGATACGCCACCCTTTTTCGACCAGTCC

CCTTCCAACGGACTTGCTCAATCATCGTCGCTTCTTTCGCCACTCCCCACCCCGGTGAACGGCATGAATGGATTCGTCACCTCCACACCTATGTCAGGATATGACGACT

TCGTCAGAACGGTACGGGACCAATCCGATAATGAGAGCCCTCAATTCATGATGGACCCTTGGAACGGCATGCCCATGGATACAACCTTTGATCCCATGCGGATAGACC

CCGCGTTGATGATGAGCATGAGTATGGACATGAGCATGGGACCACCTCAGGATGGAATCCTTGGCATGATACCAGAAATGTCGCCTGCTCAAACATTTGGACAGCCTG

TCCAGACACCTCGCATGGACGAGTCCTTTTCGGACCTGCAAATTGGTAGTTCGGCTTCGATGTTCTACCCATCCAACAGGCACTCTTCGATAGCAGATGCCGGCATAC

CTGATCTTGGTGCCATCATCGCGGCTCAAGATGGCTGGACTGTTTTCCGTTGCACTCCTTCTATTGCTTCAAGCTCGTGTCCACGCACAGCGAGACTCAACCTCGAGC

GGTTAGAGCTGAGCCTCAAGAATCACGAGGGCTGGGCTAACTGGTCCCCATCGTGGGATGAATCCGACTTTCAACAAGGCGGGCAAATCGCGGTATCGAAGTTGCAG

GAAGCCACGCGAGACAAACTTCTCGCCATCACCCAAAGCTTCCTCCACAAGGCTTTGGACATCCACAAAGACGGAAGCAGCTCGTCCAGCAGTGGCGAGTCCCCAGG

ATCCACGGCCTCTCACTCCAACTTCGTTCTTCTTCCACCTGCTCGCGTCCTGGAGTACTTTCTCAAGTCATACGCCAACTCTTTCGAACGCTATTATCCCACCTCCGCCA

GAGGTGTGCTCGATACGAATGAACTAATGCAAAACTACAACGACAAAGCATCGAGCTTGCTCATTCTCATGATGATCGCGCAGGGCGCCATGACCATTCCATCCATAG

AGGCCAGGTGGCTAACTGGTGGCTTCACCGAAGCCTGCCGGATATCGCTCTTCGACCTTATTGAGAAGAACATCATCATGGCTGGTGATCCTATCGTTCTGCGTGCAG

CCCTCCTCTTCACTGTCCAGGCGGCGTGGAGTGGCGACAAATGGCAGATGGACATCGCTATGGGCCAGCGTGGCATGTATTTCTCCATGTTGCGACATTCCGGTATTC

TCGATGTGCGACCGCCGGCAACGCCTCATATCAACGGAAATATTTCGACGGATGCGTTGTGGAAGGACTGGCTTCAGCAAGAAAGCCGCAGTCGGTAAGTGTTGAAA

ACTTCGGTACCGTCACGTGAACGCACTTACTAATATTGGCGCAGCCTCATCTACTCCTGGGTCATGGTAGACCAAGACTTATCGCTCTTCCACGATACCGCTCCACTGT

TCTCAGTAACAGAGTTTGGCGCGCCGATGCCTGATTCGGATCGACTTTGGCAAGCGCAGACCGCCTCAGAGTGGTCTGAGATGTTCAACCAAGTCCATGAGTTCTCGA

ACGGGTACTCTTCAGTAGGCTCTGGGGCCAGACCACCCAGTCTCAGAGATCTGTTCCGTTACTTCCTAGATGACGAAATCGTCGTACAGGATGTACAGGAGATTCATC

TGACACCTATGCACCTGCGTCTGCTCCTCCACCCCCTGCAGACTCTGGTCTGCCAGTATTGCCAGCTTCTCAGCTGCTTTTCCGATAGCGTCGCTTCACGATCGCGGA

ATCGCGCACTGACAGCAGCGTCGACCCGCGTGCGTCTCGAGGAGGTGCAGGCGTTGCTAGGGCGTTGGTTCGATTTGGCGCAACGGTACATGAAGTCCAACCCAGT

GTGTCCGATGATGCAGGCGAACCTTGTCATGTTCCATCTTATCAGCTTGAACGCTGTGTCCAACTTTCCAGAGATAGAGCGCCTTGCGCGTCGAGAGGGTGTGGACGG

CTCATACCAGCAGATGCTGTGGGTGCACAAGAAGTGTCTGTCCGATGTGCAGGAGGCCATCACCCACGCCGGTCAGGTCCTTCGACTCATCCGTGAGATGCCAAGAG

GTATCCGGCCGCCATGGTGGGCTGGCGCCGTCTACCGTGCGGGTTTGGTCTTCTGGACTGATTCGCTGGCACATGGCGAGTCCTTGAGTCCCAACCACCAAGGATC

GTATCAGCCATCGAACTCCGCGTTCTCTGTGGATCGCCTTCCAGCTGACCATCCTACCATACTGAGGTACATGTCTCGTAGGGAGGGCATACCCACTCTGACCAAGCG

CGACGGTACACACGTCCCTTTGGATAACGGCTTCGCGATCCTGTCGCATTGTATCGACGTCCTTGATGAGGGTGTTGCCACCAGGTTCTCCGATGGCATTCGTAGTAA

ACTCGAGAAACTCGCGCGAGGATAA

>*Alternaria turkisafria* (NA)

ATGGTCTTCTGCACATATTGTGGACAGTCGTTCACCAGGGACGAACATCTCGAACGACATATCCTAACTCGTAAGTCAACTCTTGCTCTGTCCATGAGGGTGGATTTCC

GCAATGTCGAGTTCAATCCGATCGTTCCGTAGCATCTCCGCTGACCGTCAATGCCAAACAGACACCAATGTCAAGCCATTCAAGTGCTTTACATGTCATATGAGCTTTG

CTCGACGGTAAGTCCCACCCTGCGTGCGCTCCTTCCTCTTCCTGTTTGATCATAATTTCCGTACCGAACGAAACGCTGACAATCTTTCCAGAGATCTTTTGCAAAGACAT

TACACCGTACACGGCCGCAACCAGAACAACGAAGAAGGCCTCCCACCGCAAGGCATCATACCTAAGTCCGCCGGCCGCACACCCATCGCCTGCAGTAATTGTGCAAA

GACGAAGACGAAATGCGACAAGAAGTTTCCTTGCACCCGGTGCGCTCAAAGGAACCTGAAGTGTACTCTACGTCCTACACGGCGAGCATCGAAAGGTGTCCAACGAG

TAGGAGGTCCTCCTGAAACAGGCAGTGGTGATTCTAGCGAGAGTGGTAGCAACGCGGATAACCAGAACACATCCGGCAATGTCTCTCCAAACCACGATGGCCAGTCG

AAACAAGCAACCGCTCAGTCGTCTCCATCTCAAATTCAGCAGCCTAGCCAGATTTCTGACAGCCAACCTCCATCGCAACGGCAATCGCAATCTCCCTCAGCGCCCCAC

TCGCGGAATCCTTCCATCTCACATCCAGTGCAACCACCACCGCACATGAACCCCGAGGAGAAGTCGCTCCACATACACATGCCTATCTCGAATACTCCCCCGTTCTTC

GATCAGTCACCCTCTAACGGACTGTCTCATTCCTCATCATTACTTTCACCTCTTCCCACACCAGTTACGGGCATGAATAGTTTTGTTTCTTCGACACCCATGTCTGGGTA

CGACGACTTCGTCAGGACAGTGCGCGATCAACCGGAAAATGAGAGCCCGCAATTCATGATGGATCCTTGGCACTCGATGCCCATGGATACGAGTTACGATCCTATGC

GGATTGACCCCGCTCTGATGATGACCATGGGCATGGACATGACGATGGGACCGCCCCAAGACGGTATCCTTAGTATGATACCCGAAATGTCACCTGCGCAAACATTCG

GACAGCCTGTTCAGACTCCTCGTATGGTGGACGAATCGTTCTCGGACTTGCAAATCGGCAGTTCGGCTTCGATGTTCTACCCCTCTAACAGACATTCCTCAATAGCGGA

CGCCGGCATCCCTGATCTTGGCGCCATTATCGCAGCCCAAGACGGCTGGACGGTCTTCCGTTGCACTCCATCTATCGCTTCGAGCTCATGTCCGCGGACCGCAAGGC

TGAATCTTGAGAGATTAGAGTTGAGCCTGAAGAACCATGAGGGCTGGGCTAACTGGACCCCATCGTGGGACGAATCTGATTTCCAACAAGGCGGACAGATCGCCGTG

TCAAAACTCCAGGAAGCCACACGAGACAAACTCCTCGCGATCACACAGAGCTTCCTTCACAAGGCTCTAGATATCCACAAAGATGGAAGCAGCTCGTCTAGTAGTGGC

GAGTCTCCAGGATCTACGGCTTCACATTCCAACTTCGTTCTTCTTCCGCCTGCTCGTGTCCTGGAGTACTTCCTCAAGTCGTACGCCAACTCTTTCGAACGCTATTATCC

TACCTCGGCCAGAGGAGTCCTCGACACCAACGAGTTGATGCAGAACTACAATGACAAAGCTTCGAGCTTGCTCATTCTCATGATGATTGCGCAGGGCGCTATGACCAT

TCCGTCCATAGAAGCGAGATGGCTGACTGGCGGCTTTACTGAGGCATGTCGGATATCCCTGTTCGACCTCATCGAGAAGAACATCATTATGGCTGGCGATCCTATTGT

CCTGAGGGCGGCCCTTCTCTTCACTGTTCAAGCGGCGTGGAGTGGCGACAAGTGGCAGATGGACATCGCCATGGGTCAGCGCGGCATGTATTTTTCCATGTTACGAC

ATTCCGGTATTCTCGATGTTCGACCGCCTACCACGCCTCACATGAATGGAAACACTTCGACGGATGTGTTGTGGAAGGACTGGCTTCAGCAAGAAAGCCGCAGCCGGT

AAGTGTTGAAAACCTCGGTACAGTCACGTGCGCGCACTTACTAATATCGTGTAGCCTCATCTACTCTTGGGTCATGGTAGACCAAGACCTATCGCTATTCCACGATACC

GCTCCCCTGTTTTCGGTAACAGAATTTGGCGCGCCAATGCCTGATTCAGATCAACTCTGGCAGGCGCAGACAGCTCCAGAGTGGTCGGAGATGTTCAACCAGGTCCAT

GAATTCTCGAATGGATACTCCTCAGTAGGCTCTGGAGCAAGACCACCGAGTCTCAGAGATCTCTTCCGCTACTTCCTAGATGACGAGATAGTCGTCCAGGATGTTCAG

GAGATTCATCTGACCCCGATGCACCTGCGACTACTACTGCACCCTCTGCAGACCCTTGTGTGCCAATATTGCCAACTCCTCAGTTGCTTTTCCGACAGTGTAGCCTCTC

GATCGCGGAATCGGGCATTGACAGCTGCTTCGACTCGCGTACGCCTAGAAGAGGTGCAGGCGTTATTGGGGCGGTGGTTCGACTTGGCGCAACGGTACATGAAGGC

GAACCCAGTATGTCCGATGATGCAAGCAAATCTCGTCATGTTCCATCTTATCAGCATGAATGCTGTGTCAAATTTCCCAGAGATTGAGCGCCTCGCCCGACGGGAGGG

TGTGGACGGCTCGTATCAACAGATGCTGTGGATGCACAAGAAGTGCTTGTCGGACGTGCAGGAGGCCATCACTCACGCTGGCCAAGTTCTTCGCCTCATTCGTGAGA

TGCCACGAGGCATTCGCCCGCCGTGGTGGGCCGGTGCTGTCTATCGGGCAGGGTTGGTCTTCTGGGCTGACTCGCTCGCTCACAACGAGTCCTTGAGTCCCAACCA

TCAAGGGACATATCAGCCATCTAATACTGCTTTCTCTGTGGATCGCCTCTCTGCTGACCACCCAACCATACTGAGGTACATGTCACGGAGGGAGGGTATTCCCACCTTG

ACCAAGCGCGATGGTAGCCCTGTTCCACTGGATAACGGCTTTGCCGTCCTGTCGCATTGTATCGATGTCCTTGACGAGGGTGTCGCCACCAGGTTTTCCGATGGCATT

CGTAGTAAACTCGAGAAACTTGCGCGCGGTTAA

>*Alternaria ventricosa* (NA)

ATGGTCTTTTGCACATACTGTGGACAGTCGTTCACCAGAGACGAGCATCTCGAACGACATATCTTGACTCGTATGCTGACCCCGCGTCTTTCGCTCTTTCATTCTTCCTC

GCACCAGCTACCATCCAATCTTTCTTCTGGGATATCTGCTAATCACTGTGGATGAGCAGACACCAATGTCAAGCCTTTCAAATGCTTCACATGTCATATGAGCTTTGCTC

GACGGTATGTGGCACCCTTCCTTGCTCTTTCTCATCCTGTGGCATCGACCGTTCGGAACAAAGTACTGACAGCCTCTTCAGAGATCTTTTGCAAAGACACTACACCGTA

CACGGCCGCAACCAGAACAACGAAGAGGGACTCCCCCCACAAGGCATAATACCAAAGTCCGCCGGCCGCACACCCATCGCCTGCAGTAATTGTGCGAAGACAAAGAC

CAAGTGTGACAAGAAGTTTCCGTGCACCAGGTGCGCTCAGAGGAATCTGAAATGCACATTGCGTCCTACAAGGCGGGCATCGAAAGGTGTCCAGCGGGTTGGCGGTC

CTCCTGAGGCTGGGAGCGGGGACTCTAGCGAGAGTGGCAGCAACGCGGATAACCACAATACATCCGGCAATGTCTCTCCAAACCACGATAGCCAACCGAAACAAACG

ATGTCGAAGTCCTCTCCTGCTCAGATCAACCAGACTTCCCATATTCCGGACAGCCAACCACCGTCGCAAAGACAATCGCAGTCGCCCTCGGCCCCTCATTCAAGAAAC

CCTTCCATCTCACAACCGGTACAGCCACCGACACACATAAATCCCGAGGAGAAGTCGTTGCATCTTCATATGCCGATCTCCGATACGCCGCCCTTTTTCGACCAGTCC

CCTTCCAACGGACTTGCTCAATCATCGTCGCTTCTTTCGCCACTCCCCACCCCGGTGAACGGCATGAATGGATTCGTCACCTCCACACCTATGTCAGGATATGACGACT

TCGTCAGAACGGTACGGGACCAATCCGATAATGAGAGCCCTCAATTCATGATGGACCCTTGGAATGGCATGCCCATGGATACAACCTTTGATCCCATGCGGATAGACC

CCGCGTTGATGATGAGCATGAGTATGGACATGAGCATGGGACCACCTCAGGACGGCATCCTTGGCATGATACCAGAAATGTCGCCTGCTCAAACATTTGGACAGCCTG

TCCAGACACCTCGCATGGACGAGTCCTTTTCGGACCTGCAAATTGGTAGTTCGGCTTCGATGTTCTACCCCTCCAACAGGCACTCTTCGATAGCAGATGCCGGCATAC

CTGATCTTGGTGCCATCATCGCGGCTCAAGATGGCTGGACTGTTTTCCGTTGCACTCCTTCTATTGCTTCAAGCTCGTGTCCACGCACAGCGAGACTCAACCTCGAGC

GGTTAGAGCTGAGCCTCAAGAATCACGAGGGCTGGGCTAATTGGTCCCCGTCGTGGGATGAATCCGACTTTCAACAAGGCGGGCAAATCGCGGTATCGAAGTTGCAG

GAAGCCACGCGAGACAAACTTCTCGCCATCACCCAAAGCTTCCTCCACAAGGCTTTGGACATCCACAAAGACGGAAGCAGCTCGTCCAGCAGTGGCGAGTCTCCAGG

ATCCACGGCCTCTCACTCCAACTTCGTTCTTCTTCCACCTGCTCGCGTCCTGGAGTACTTTCTCAAGTCATACGCCAACTCTTTCGAACGCTATTATCCCACCTCCGCCA

GAGGTGTGCTCGATACGAATGAACTAATGCAAAACTACAACGACAAAGCATCGAGCTTGCTCATTCTCATGATGATTGCGCAGGGCGCCATGACCATCCCATCCATAG

AGGCCAGGTGGCTAACTGGTGGCTTCACCGAAGCCTGCCGGATATCGCTCTTCGACCTTATCGAGAAGAACATCATCATGGCTGGTGATCCTATCGTTCTGCGTGCAG

CCCTCCTCTTCACTGTCCAGGCGGCGTGGAGTGGCGACAAATGGCAGATGGACATCGCTATGGGCCAGCGTGGCATGTATTTCTCCATGTTGCGACATTCCGGTATTC

TCGATGTGCGACCGCCAGCAACGCCTCATATCAACGGAAACATTTCGACGGATGCGTTGTGGAAGGACTGGCTTCAGCAAGAAAGCCGCAGTCGGTAAGTGTTGAAA

ACCTCGGTACCGTCACGTGAACGCACTTACTAATATTGGCGCAGCCTCATCTACTCCTGGGTCATGGTAGACCAAGACTTATCGCTCTTCCACGATACCGCTCCACTGT

TCTCAGTAACAGAGTTTGGCGCGCCGATGCCTGATTCGGATCGACTTTGGCAAGCGCAGACCGCCTCAGAGTGGTCTGAGATGTTCAACCAAGTCCATGAGTTCTCGA

ATGGGTACTCTTCAGTAGGCTCTGGGGCCAGACCACCTAGTCTCAGAGATCTCTTCCGTTACTTTCTAGATGACGAAATCGTCGTACAGGATGTACAGGAGATTCATCT

GACACCTATGCACCTGCGTCTGCTCCTCCACCCCCTGCAGACTCTGGTCTGCCAGTATTGCCAGCTTCTCAGCTGCTTTTCCGATAGCGTCGCTTCACGATCGCGGAA

CCGCGCACTGACAGCAGCGTCGACCCGCGTGCGTCTCGAGGAGGTGCAGGCGTTGCTAGGGCGTTGGTTCGATTTGGCGCAACGGTACATGAAGTCCAACCCAGTG

TGTCCGATGATGCATGCCAACCTTGTAATGTTCCATCTTATCAGCTTGAACGCTGTGTCCAACTTTCCAGAGATAGAGCGCCTTGCGCGTCGAGAGGGCGTGGACGGC

TCATACCAGCAGATGCTGTGGATGCACAAGAAGTGTCTGTCCGATGTACAGGAGGCCATCACCCACGCCGGTCAGGTCCTTCGACTCATCCGTGAGATGCCAAGAGG

TATCCGGCCGCCATGGTGGGCTGGCGCCGTCTACCGTGCGGGTTTGGTCTTCTGGACTGATTCGCTGGCACATGGCGAGTCCTTGAGTCCCAACCACCAAGGATCGT

ATCAGCCATCGAACTCCGCGTTCTCTGTGGATCGCCTTCCTGCTGACCACCCTACCATACTGAGGTATATGTCTCGCAGGGAGGGCATACCCACTCTGACCAAGCGCG

ACGGTACACACGTCCCCTTGGATAACGGGTTCGCCATCCTATCGCATTGTATCGACGTCCTTGATGAGGGTGTTGCCACCAGGTTCTCCGATGGCATTCGTAGTAAAC

TCGAGAAACTCGCGCGAGGATAA

>*Alternaria viburni* (J4E79_003358[a])

ATGGTCTTTTGCACATACTGTGGACAGTCGTTCACCAGAGACGAGCATCTCGAACGACATATCTTGACTCGTATGTTGACCCCGCGTCTTTCGCTCTTTCAATCTTCCTC

GCACCAGCTACCATCCAATCTTTCTTCTGGGATATCTGCTAATCACTGTGGATGAGCAGACACCAACGTCAAGCCTTTCAAATGCTTCACATGTCATATGAGCTTTGCTC

GACGGTATGTGGCACCCTTCCTTGCTCTTTCTCATCCTGTGGCATCGACCGTTCGGAACAAAGTACTGACAGCCTCTTCAGAGATCTTTTGCAAAGACACTACACCGTA

CACGGCCGCAACCAGAACAACGAAGAGGGACTCCCCCCACAAGGCATAATACCAAAGTCCGCCGGCCGCACACCCATCGCCTGCAGTAATTGTGCGAAGACAAAGAC

CAAGTGTGACAAGAAGTTTCCGTGCACCAGGTGCGCTCAGAGGAATCTGAAATGCACATTGCGTCCTACAAGGCGGGCATCGAAAGGTGTCCAGCGGGTTGGCGGTC

CTCCTGAGGCTGGGAGCGGGGACTCGAGCGAGAGTGGTAGCAACGCGGATAACCACAATACATCCGGCAATGTCTCTCCAAACCACGATAGCCAACCGAAACAAGC

GATGTCGAAGTCCTCTCCTGCTCAGATCAACCAGACTTCCCATATTCCGGACAGCCAACCACCGTCGCAAAGACAATCGCAGTCGCCCTCGGCCCCTCATTCACGAAA

CCCTTCCATCTCACAACCGGTACAGCCACCGACACACATAAATCCCGAGGAGAAGTCGTTGCATCTTCATATGCCGATCTCCGATACGCCACCCTTTTTCGACCAGTCC

CCTTCCAACGGACTTGCTCAATCATCGTCGCTTCTTTCGCCACTCCCCACCCCGGTGAACGGCATGAATGGATTCGTCACCTCCACACCTATGTCAGGATATGACGACT

TCGTCAGAACGGTACGGGACCAATCCGATAATGAGAGCCCTCAATTCATGATGGACCCTTGGAACGGCATGCCCATGGATACAACCTTTGATCCCATGCGGATAGACC

CCGCGTTGATGATGAGCATGAGTATGGACATGAGCATGGGACCACCTCAGGATGGAATCCTTGGCATGATACCAGAAATGTCGCCTGCTCAAACATTTGGACAGCCTG

TCCAGACACCTCGCATGGACGAGTCCTTTTCGGACCTGCAAATTGGTAGTTCGGCTTCGATGTTCTACCCATCCAACAGGCACTCTTCGATAGCAGATGCCGGCATAC

CTGATCTTGGTGCCATCATCGCGGCTCAAGATGGCTGGACTGTTTTCCGTTGCACTCCTTCTATTGCTTCAAGCTCGTGTCCACGCACAGCGAGACTCAACCTCGAGC

GGTTAGAGCTGAGCCTCAAGAATCACGAGGGCTGGGCTAACTGGTCCCCATCGTGGGATGAATCCGACTTTCAACAAGGCGGGCAAATCGCGGTATCGAAGTTGCAG

GAAGCCACGCGAGACAAACTTCTCGCCATCACCCAAAGCTTCCTCCACAAGGCTTTGGACATCCACAAAGACGGAAGCAGCTCGTCCAGCAGTGGCGAGTCTCCAGG

ATCCACGGCCTCTCACTCCAACTTCGTTCTTCTTCCACCTGCTCGCGTCCTGGAGTACTTTCTCAAGTCATACGCCAACTCTTTCGAACGCTATTATCCCACCTCCGCCA

GAGGTGTGCTCGATACGAATGAACTAATGCAAAACTACAACGACAAAGCATCGAGCTTGCTCATTCTCATGATGATCGCGCAGGGCGCCATGACCATTCCATCCATAG

AGGCCAGGTGGCTAACTGGTGGCTTCACCGAAGCCTGCCGGATATCGCTCTTCGACCTTATTGAGAAGAACATCATCATGGCTGGTGATCCTATCGTTCTGCGTGCAG

CCCTCCTCTTCACTGTCCAGGCGGCGTGGAGTGGCGACAAATGGCAGATGGACATCGCTATGGGCCAGCGTGGCATGTATTTCTCCATGTTGCGACATTCCGGTATTC

TCGATGTGCGACCGCCGGCAACGCCTCATATCAACGGAAATATTTCGACGGATGCGTTGTGGAAGGACTGGCTTCAGCAAGAAAGCCGCAGTCGGTAAGTGTTGAAA

ACTTCGGTACCGTCACGTGAACGCACTTACTAATATTGGCGCAGCCTCATCTACTCCTGGGTCATGGTAGACCAAGACTTATCGCTCTTCCACGATACCGCACCACTGT

TCTCAGTAACAGAGTTTGGCGCGCCGATGCCTGATTCGGATCGACTTTGGCAAGCGCAGACCGCCTCAGAGTGGTCTGAGATGTTCAACCAAGTCCATGAGTTCTCGA

ACGGGTACTCTTCAGTAGGCTCTGGGGCCAGACCACCCAGTCTCAGAGATCTGTTCCGTTACTTCCTAGATGACGAAATCGTCGTACAGGATGTACAGGAGATTCATC

TGACACCTATGCACCTGCGTCTGCTCCTCCACCCCCTGCAGACTCTGGTCTGCCAGTATTGCCAGCTTCTCAGCTGCTTTTCCGATAGCGTCGCTTCACGATCGCGGA

ATCGCGCACTGACAGCAGCGTCGACCCGCGTGCGTCTCGAGGAGGTGCAGGCGTTGCTAGGGCGTTGGTTCGATTTGGCGCAACGGTACATGAAGTCCAACCCAGT

GTGTCCGATGATGCAGGCGAACCTTGTCATGTTCCATCTTATCAGCTTGAACGCTGTGTCCAACTTTCCAGAGATAGAGCGCCTTGCGCGTCGAGAGGGTGTGGACGG

CTCATACCAGCAGATGCTGTGGATGCACAAGAAGTGTCTGTCCGATGTGCAGGAGGCCATCACCCACGCCGGTCAGGTCCTTCGACTCATCCGTGAGATGCCAAGAG

GTATCCGGCCGCCATGGTGGGCTGGCGCCGTCTACCGTGCGGGTTTGGTCTTCTGGACTGATTCGCTGGCACATGGCGAGTCCTTGAGTCCCAACCACCAAGGATC

GTATCAGCCATCGAACTCCGCGTTCTCTGTGGATCGCCTTCCAGCTGACCATCCTACCATACTGAGGTACATGTCTCGTAGGGAGGGCATACCCACTCTGACCAAGCG

CGACGGTACACACGTCCCTTTGGATAACGGCTTCGCGATCCTGTCGCATTGTGTCGACGTCCTTGATGAGGGTGTTGCCACCAGGTTCTCCGATGGCATTCGTAGTAA

ACTCGAGAAACTCGCGCGAGGATAA

Fig. S1 Gene sequences of 44 Cmr1 homologs in *Alternaria*. The sequences were acquired from the following resources: [a]GenBank, National Center for Biotechnology Information (http://www.ncbi.nih.gov); [b]AGD, *Alternaria* Genome Database (http://alternaria.vbi.vt.edu); [c]JGI, Joint Genome Institute, US Department of Energy (http://www.jgi.doe.gov). NA=not applicable.

>*Alternaria alstroemeriae* (NA)

MVFCTYCGQSFTRDEHLERHILTHTNVKPFKCFTCHMSFARRDLLQRHYTVHGRNQNNEEGLPPQGIIPKSAGRTPIACSNCAKTKTKCDKKFPCTRCAQRNLKCTLRPTRRA

SKGVQRVGGPPETGSGDSSESGSNADNQNTSGNVSPNHDGQSKQATAQSSPSQIQQPSQISDSQPPSQRQSQSPSAPHSRNPSISHPVQPPPHMNPEEKSLHIHMPISNT

PPFFDQSPSNGLSHSSSLLSPLPTPVTGMNSFVSSTPMSGYDDFVRTVRDQPENESPQFMMDPWHSMPMDTSYDPMRIDPALMMTMGMDMTMGPPQDGILSMIPEMSPA

QTFGQPVQTPRMVDESFSDLQIGSSASMFYPSNRHSSIADAGIPDLGAIIAAQDGWTVFRCTPSIASSSCPRTARLNLERLELSLKNHEGWANWTPSWDESDFQQGGQIAVS

KLQEATRDKLLAITQSFLHKALDIHKDGSSSSSSGESPGSTASHSNFVLLPPARVLEYFLKSYANSFERYYPTSARGVLDTNELMQNYNDKASSLLILMMIAQGAMTIPSIEARW

LTGGFTEACRISLFDLIEKNIIMAGDPIVLRAALLFTVQAAWSGDKWQMDIAMGQRGMYFSMLRHSGILDVRPPTTPHMNGNTSTDVLWKDWLQQESRSRLIYSWVMVDQDL

SLFHDTAPLFSVTEFGAPMPDSDQLWQAQTAPEWSEMFNQVHEFSNGYSSVGSGARPPSLRDLFRYFLDDEIVVQDVQEIHLTPMHLRLLLHPLQTLVCQYCQLLSCFSDSV

ASRSRNRALTAASTRVRLEEVQALLGRWFDLAQRYMKANPVCPMMQANLVMFHLISMNAVSNFPEIERLARREGVDGSYQQMLWMHKKCLSDVQEAITHAGQVLRLIREMP

RGIRPPWWAGAVYRAGLVFWADSLAHNESLSPNHQGTYQPSNTAFSVDRLSADHPTILRYMSRREGIPTLTKRDGSPVPLDNGFAVLSHCIDVLDEGVATRFSDGIRSKLEKL

ARG

>*Alternaria alternata* (AIG15468[a])

MVFCTYCGQSFTRDEHLERHILTHTNVKPFKCFTCHMSFARRDLLQRHYTVHGRNQNNEEGLPPQGIIPKSAGRTPIACSNCAKTKTKCDKKFPCTRCAQRNLKCTLRPTRRA

SKGVQRVGGPPETGSGDSSESGSNADNQNTSGNVSPNHDGQSKQATAQSSPSQIQQPSQISDSQPPSQRQSQSPSAPHSRNPSISHPVQPPPHMNPEEKSLHIHMPISNT

PPFFDQSPSNGLSHSSSLLSPLPTPVTGMNSFVSSTPMSGYDDFVRTVRDQPENESPQFMMDPWHSMPMDTSYDPMRIDPALMMTMGMDMTMGPPQDGILSMIPEMSPA

QTFGQPVQTPRMVDESFSDLQIGSSASMFYPSNRHSSIADAGIPDLGAIIAAQDGWTVFRCTPSIASSSCPRTARLNLERLELSLKNHEGWANWTPSWDESDFQQGGQIAVS

KLQEATRDKLLAITQSFLHKALDIHKDGSSSSSSGESPGSTASHSNFVLLPPARVLEYFLKSYANSFERYYPTSARGVLDTNELMQNYNDKASSLLILMMIAQGAMTIPSIEARW

LTGGFTEACRISLFDLIEKNIIMAGDPIVLRAALLFTVQAAWSGDKWQMDIAMGQRGMYFSMLRHSGILDVRPPTTPHMNGNTSTDVLWKDWLQQESRSRLIYSWVMVDQDL

SLFHDTAPLFSVTEFGAPMPDSDQLWQAQTAPEWSEMFNQVHEFSNGYSSVGSGARPPSLRDLFRYFLDDEIVVQDVQEIHLTPMHLRLLLHPLQTLVCQYCQLLSCFSDSV

ASRSRNRALTAASTRVRLEEVQALLGRWFDLAQRYMKANPVCPMMQANLVMFHLISMNAVSNFPEIERLARREGVDGSYQQMLWMHKKCLSDVQEAITHAGQVLRLIREMP

RGIRPPWWAGAVYRAGLVFWADSLAHNESLSPNHQGTYQPSNTAFSVDRLSADHPTILRYMSRREGIPTLTKRDGNPVPLDNGFAVLSHCIDVLDEGVATRFSDGIRSKLEKL

ARG

>*Alternaria angustiovoidea* (NA)

MVFCTYCGQSFTRDEHLERHILTHTNVKPFKCFTCHMSFARRDLLQRHYTVHGRNQNNEEGLPPQGIIPKSAGRTPIACSNCAKTKTKCDKKFPCTRCAQRNLKCTLRPTRRA

SKGVQRVGGPPETGSGDSSESGSNADNQNTSGNVSPNHDGQSKQATAQSSPSQIQQPSQISDSQPPSQRQSQSPSAPHSRNPSISHPVQPPPHMNPEEKSLHIHMPISNT

PPFFDQSPSNGLSHSSSLLSPLPTPVTGMNSFVSSTPMSGYDDFVRTVRDQPENESPQFMMDPWHSMPMDTSYDPMRIDPALMMTMGMDMTMGPPQDGILSMIPEMSPA

QTFGQPVQTPRMVDESFSDLQIGSSASMFYPSNRHSSIADAGIPDLGAIIAAQDGWTVFRCTPSIASSSCPRTARLNLERLELSLKNHEGWANWTPSWDESDFQQGGQIAVS

KLQEATRDKLLAITQSFLHKALDIHKDGSSSSSSGESPGSTASHSNFVLLPPARVLEYFLKSYANSFERYYPTSARGVLDTNELMQNYNDKASSLLILMMIAQGAMTIPSIEARW

LTGGFTEACRISLFDLIEKNIIMAGDPIVLRAALLFTVQAAWSGDKWQMDIAMGQRGMYFSMLRHSGILDVRPPTTPHMNGNTSTDVLWKDWLQQESRSRLIYSWVMVDQDL

SLFHDTAPLFSVTEFGAPMPDSDQLWQAQTAPEWSEMFNQVHEFSNGYSSVGSGARPPSLRDLFRYFLDDEIVVQDVQEIHLTPMHLRLLLHPLQTLVCQYCQLLSCFSDSV

ASRSRNRALTAASTRVRLEEVQALLGRWFDLAQRYMKANPVCPMMQANLVMFHLISMNAVSNFPEIERLARREGVDGSYQQMLWMHKKCLSDVQEAITHAGQVLRLIREMP

RGIRPPWWAGAVYRAGLVFWADSLAHNESLSPNHQGTYQPSNTAFSVDRLSADHPTILRYMSRREGIPTLTKRDGSPVPLDNGFAVLSHCIDVLDEGVATRFSDGIRSKLEKL

ARG

>*Alternaria arborescens* (XP_028511872[a])

MVFCTYCGQSFTRDEHLERHILTHTNVKPFKCFTCHMSFARRDLLQRHYTVHGRNQNNEEGLPPQGIIPKSAGRTPIACSNCAKTKTKCDKKFPCTRCAQRNLKCTLRPTRRA

SKGVQRVGGPPETGSGDSSESGSNADNQNTSGNVSPNHDGQSKQATAQSSPSQIQQPSQISDSQPPSQRQSQSPSAPHSRNPSISHPVQPPPHMNPEEKSLHIHMPISNT

PPFFDQSPSNGLSHSSSLLSPLPTPVTGMNSFVSSTPMSGYDDFVRTVRDQPENESPQFMMDPWHSMPMDTSYDPMRIDPALMMTMGMDMTMGPPQDGILSMIPEMSPA

QTFGQPVQTPRMVDESFSDLQIGSSASMFYPSNRHSSIADAGIPDLGAIIAAQDGWTVFRCTPSIASSSCPRTARLNLERLELSLKNHEGWANWTPSWDESDFQQGGQIAVS

KLQEATRDKLLAITQSFLHKALDIHKDGSSSSSSGESPGSTASHSNFVLLPPARVLEYFLKSYANSFERYYPTSARGVLDTNELMQNYNDKASSLLILMMIAQGAMTIPSIEARW

LTGGFTEACRISLFDLIEKNIIMAGDPIVLRAALLFTVQAAWSGDKWQMDIAMGQRGMYFSMLRHSGILDVRPPTTPHMNGNTSTDVLWKDWLQQESRSRLIYSWVMVDQDL

SLFHDTAPLFSVTEFGAPMPDSDQLWQAQTAPEWSEMFNQVHEFSNGYSSVGSGARPPSLRDLFRYFLDDEIVVQDVQEIHLTPMHLRLLLHPLQTLVCQYCQLLSCFSDSV

ASRSRNRALTAASTRVRLEEVQALLGRWFDLAQRYMKANPVCPMMQANLVMFHLISMNAVSNFPEIERLARREGVDGSYQQMLWMHKKCLSDVQEAITHAGQVLRLIREMP

RGIRPPWWAGAVYRAGLVFWADSLAHNESLSPNHQGTYQPSNTAFSVDRLSADHPTILRYMSRREGIPTLTKRDGSPVPLDNGFAVLTHCIDVLDEGVATRFSDGIRSKLEKL

ARG

>*Alternaria arbusti* (NA)

MVFCTYCGQSFTRDEHLERHILTHTNVKPFKCFTCHMSFARRDLLQRHYTVHGRNQNNEEGLPPQGIIPKSAGRTPIACSNCAKTKTKCDKKFPCTRCAQRNLKCTLRPTRRA

SKGVQRVGGPPEAGSGDSSESGSNADNHNTSGNVSPNHDSQPKQAVSKSSPAQIHQTSHIPDSQPPSQRQSQSPSAPHSRNPSISQSVQPPTHINPEEKSLHLHMPISDTP

PFFDQSPSNGLAQSSSLLSPLPTPVTGMNGFVTSTPMSGYDDFVRTVRDQSDNESPQFMMDPWNGMPMDTTFDPMRIDPALMMSMSMDMSMGPPQDGILGMIPEMSPAQ

TFGQPVQTPRMDESFSDLQIGSSASMFYPSNRHSSIADAGIPDLGAIIAAQDGWTVFRCTPSIASSSCPRTARLNLERLELSLKNHEGWANWSPSWDESDFQQGGQIAVSKL

QEATRDKLLAITQSFLHKALDIHKDGSSSSSSGESPGSTASHSNFVLLPPARVLEYFLKSYANSFERYYPTSARGVLDTNELMQNYNDKASSLLILMMIAQGAMTIPSIEARWLT

GGFTEACRISLFDLIEKNIIMAGDPIVLRAALLFTVQAAWSGDKWQMDIAMGQRGMYFSMLRHSGILDVRPPATPHINGNISTDALWKDWLQQESRSRLIYSWVMVDQDLSLFH

DTAPLFSVTEFGAPMPDSDRLWQAQTASEWSEMFNQVHEFSNGYSSVGSGARPPSLRDLFRYFLDDEIVVQDVQEIHLTPMHLRLLLHPLQTLVCQYCQLLSCFSDSVASRS

RNRALTAASTRVRLEEVQALLGRWFDLAQRYMKSNPVCPMMQANLVMFHLISLNAVSNFPEIERLARREGVDGSYQQMLWMHKKCLSDVQEAITHAGQVLRLIREMPRGIRP

PWWAGAVYRAGLVFWTDSLAHGESLSPNHQGSYQPSNSAFSVDRLPADHPTILRYMSRREGIPTLTKRDGTHVPLDNGFAILSHCIDVLDEGVATRFSDGIRSKLEKLARG

>*Alternaria atra* (NA)

MVFCTYCGQSFTRDEHLERHILTHTNVKPFKCFTCHMSFARRDLLQRHYTVHGRNQNNEEGLPPQGIIPKSAGRTPIACSNCAKTKTKCDKKFPCTRCAQRNLKCTLRPTRRA

SKGVQRVGGPPEAGSGDSSESGSNADNQNTSGNVSPNHDGQPKQATAQSSPSQIQQPAQVSDSQPPSQRQSQSPSAPHSRNPSISHPVQPPPHINPEEKSLHIHMPISNT

PPFFDQSPSNGLAHSSSLLSPLPTPVTGMNSFVSSTPMSGYDDFVRTVRDQSDNESPQFIMDPWHSMPMDTSFDPMRIDPSLIMTMGMDMTMGPPQDGILGMIPEMSPAQT

FGQPVQTPRMVDESFSDLQIGSSASMFYPSNRHSSIADAGIPDLGAIIAAQDGWTVFRCTPSIASSSCPRTARLNLERLELSLKNHEGWANWTPSWDESDFQQGGQIAVSKLH

EATRDKLLAITQSFLHKALDIHKDGSSSSSSGESPGSTASHSNFVLLPPARVLEYFLKSYANSFERYYPTSARGVLDANELMQNYNDKASSLLILMMIAQGAMTIPSIEARWLTG

GFTEACRISLFDLIEKNIIMAGDPIVLRAALLFTVQAAWSGDKWQMDIAMGQRGMYFSMLRHSGILDVRPPTTPHMNGNTSTDVLWKDWLQQESRSRLIYSWVMVDQDLSLF

HDTAPLFSVTEFGAPMPDSDQLWQAQTASEWSEMFNQVHEFSNGYSSVGSGARPPSLRDLFRYFLDDEIVVQDVQEIHLTPMHLRLLLHPLQTLVCQYCQLLSCFSDSVASR

SRNRALTAASTRVRLEEVQALLGRWFDLAQRYMKANPVCPMMQANLVMFHLISMNAVSNFPEIERLARREGVDGSYQQMMWMHKKCLSDVQEAITHAGQVLRLIREMPQGI

RPPWWAGAVYRAGLVFWTDSLAHSESLSPNNHQGTYQTSNAAFSVDRLPADHPTILRYMSRRDGIPTLTKRDGNPVPLDNGFAVLSHCIDVLDDGVATRFSDGIRSKLEKLA

RG

>*Alternaria brassicicola* (NA)

MVFCTYCGQSFTRDEHLERHILTHTNVKPFKCFTCHMSFARRDLLQRHYTVHGRNQNNEEGLPPQGIIPKSAGRTPIACSNCAKTKTKCDKKFPCTRCAQRNLKCTLRPTRRA

SKGIQRVGVPPETGSGDSSESGSNADNQNTSGNVSPNHDGQSKQATAQSSPSQIQKPAQVSDSQPPSQRQSQSPSAPHSRNPSISHPVQPPSHINPEEKSLHIHMPISNTP

PFFDHSPSNGLAHSSSLLSPLPTPVTGMNSFVSSTPMSGYDDFVRTVRDQSDNESPQFMMDPWHSMPMDTSFDSMRIDPTLIMNMGMDMTMGPPQDGILSMIPEMSPAQT

FGQPVQTPHMADESFSDLHMGSSASMYYPSNRHSSIADAGIPDLGAIIAAQDGWTVFRCTPSIASSSCPRTARLNLERLELSLKNHEGWANWTPSWDESDFQQGGQIAVSKL

QEATRDKLLAITQSFLHKALDIHKDGNSSSSSGESPGSTASHSNFVLLPPARVLEYFLKSYANSFERYYPTSARGVLDTNELMQNYNDKASSLLILMMIAQGAMTIPSIEARWLT

GGFTEACRISLFDLIEKNIIMAGDPIVLRAALLFTVQAAWSGDKWQMDIAMGQRGMYFSMLRHSGILDVRPPTTPHMNGNTSTDVLWKDWLQQESRSRLIYSWVMVDQDLSL

FHDTAPLFSVTEFGAPMPDSDQLWQAQTASEWSEMFNQVHEFSNGYSSVGSGARPPSLRDLFRYFLDDEIVVQDVQEIHLTPMHLRLLLHPLQTLVCQYCQLLSCFSDSVAS

RSRNRALTAASTRVRLEEVQALLGRWFDLAQRYMKANPVCPMMQANLVMFHLISMNAVSNFPEIERLARREGVDGSYQQMLWMHKKCLSDVQEAITHAGQVLRLIREMPRG

IRPPWWAGAVYRAGLVLWTDSLAHNESLSPNHQGTYQQPSNAAFSVDRLPADHPTILRYMSRRDGTPTLTKRDGTSVPLDNGFAVLSHCIDVLDEGVATRFSDGIRSKLEKL

ARG

>*Alternaria brassicae* (NA)

MVFCTYCGQSFTRDEHLERHILTHTNVKPFKCFTCHMSFARRDLLQRHYTVHGRNQNNEEGLPPQGIIPKSAGRTPIACSNCAKTKTKCDKKFPCTRCAQRNLKCTLRPTRRA

SKGVQRVGGPPETGSGDSSESGSNADNQNTSGNVSPNHDGQPKQGTAHSSPSHIQQPVQVSDSQPPSQRPSQSPSAPHSRNTSISRPVQPPPHINPEEKSLHIHMPISNTP

PFFDQSPSSGLAHSSSLLSPLPTPVTGMNSFVSSTPMSGYDDFVRTVRDQPDNESPQFMMDPWHTMPMDTNFDAMRIDPALILNMGMDMTMGPPQDGILGMIPEMSPAQT

FGQPVQTPRMVDESFSDLHLGSSASVYYTSNRHSSIADAGIPDLGAIIAAQDGWTVFRCTPSIASSSCPRTARLNLERLELSLKNHEGWANWTPSWDETDFQQGGQIAVSKL

QEATRDKLLAITQSFLHKALDIHKDGNSSSSSGESPGSTASNSNFVLLPPARVLEYFLKSYANSFERYYPTSARGVLDTNELMQNYNDKASSLLILMMIAQGAMTIPSIEARWLT

GGFTEACRISLFDLIEKNIIMAGDPIVLRAALLFTVQAAWSGDKWQMDIAMGQRGMYFSMLRHSGILDVRQPATPHMNGNTSTDVLWKDWLQQESRSRLIYSWVMVDQDLSL

FHDTAPLFSVTEFGAPMPDSDQLWQAQTASEWSEMFNQVHEFSNGYSSVGSGARPPSLRDLFRYFLDDEIVVQDVQEIHLTPMHLRLLLHPLQTLVCQYCQLLSCFSDSVAS

RSRNRALTAASTRVRLEEVQALLGRWFDLAQRYMKANPPCPMMQANLVMFHLISMNAVSNFPEIERLARRESVDGSYQQMLWMHKKCLSDVQEAITHAGQVLRLIREMPRG

IRPPWWPGAVYRAGLVFWTESLAHNESLSPNHQGTYQQPSNAALAVDRLPADHPTILRYMSRREGIPTLTKRDGTSVSLDNSYAVLSHCLDLLDEGVATRFSDGIRSKLDKLA

RG

>*Alternaria burnsii* (XP_038791471[a])

MVFCTYCGQSFTRDEHLERHILTHTNVKPFKCFTCHMSFARRDLLQRHYTVHGRNQNNEEGLPPQGIIPKSAGRTPIACSNCAKTKTKCDKKFPCTRCAQRNLKCTLRPTRRA
SKGVQRVGGPPETGSGDSSESGSNADNQNTSGNVSPNHDGQPKQATAQSSPSQIQQPSQISDSQPPSQRQSQSPSAPHSRNPSISHPVQPPPHMNPEEKSLHIHMPISNT
PPFFDQSPSNGLSHSSSLLSPLPTPVTGMNSFVSSTPMSGYDDFVRTVRDQPENESPQFMMDPWHSMPMDTSYDPMRIDPALMMTMGMDMTMGPPQDGILSMIPEMSPA
QTFGQPVQTPRMVDESFSDLQIGSSASMFYPSNRHSSIADAGIPDLGAIIAAQDGWTVFRCTPSIASSSCPRTARLNLERLELSLKNHEGWANWTPSWDESDFQQGGQIAVS
KLQEATRDKLLAITQSFLHKALDIHKDGSSSSSSGESPGSTASHSNFVLLPPARVLEYFLKSYANSFERYYPTSARGVLDTNELMQNYNDKASSLLILMMIAQGAMTIPSIEARW
LTGGFTEACRISLFDLIEKNIIMAGDPIVLRAALLFTVQAAWSGDKWQMDIAMGQRGMYFSMLRHSGILDVRPPTTPHMNGNTSTDVLWKDWLQQESRSRLIYSWVMVDQDL
SLFHDTAPLFSVTEFGAPMPDSDQLWQAQTASEWSEMFNQVHEFSNGYSSVGSGARPPSLRDLFRYFLDDEIVVQDVQEIHLTPMHLRLLLHPLQTLVCQYCQLLSCFSDSV
ASRSRNRALTAASTRVRLEEVQALLGRWFDLAQRYMKANPVCPMMQANLVMFHLISMNAVSNFPEIERLARREGVDGSYQQMLWMHKKCLSDVQEAITHAGQVLRLIREMP
RGIRPPWWAGAVYRAGLVFWADSLAHNESLSPNHQGTYQPSNTAFSVDRLSADHPTILRYMSRREGIPTLTKRDGSPVPLDNGFAVLSHCIDVLDEGVATRFSDGIRSKLEKL
ARG

>*Alternaria capsici* (NA)

MVFCTYCGQSFTRDEHLERHILTHTNVKPFKCFTCHMSFARRDLLQRHYTVHGRNQNNEEGLPPQGIIPKSAGRTPIACSNCAKTKTKCDKKFPCTRCAQRNLKCTLRPTRRA
SKGVQRVGGPPETGSGDSSESGSNADNQITSGNVSPNHDGQSKQATAQSSPSQIQQPALMSDSQPPSQRQSQSPSAPHSRNPSISHPVQPPPHMNPEEKSLHIHMPISNT
PPFFDQSPSNGLAHSSSLLSPLPTPVTGMNSFVSSTPMSGYDDFVRTVRDQPENESPQFMMDPWHSMPMDTNFDPMRIDPTLMMTMGMDMTMGPPQDGILSMIPEMSPA
QTFGQPVQTPRMVDESFSDLQIGSSASMFYPSNRHSSIADAGIPDLGAIIAAQDGWTVFRCTPSIASSSCPRTARLNLERLELSLKNHEGWANWAPSWDESDFQQGGQIAVS
KLQEATRDKLLAITQSFLHKALDIHKDGNSTSSSGESPGSTASHSNFVLLPPARVLEYFLKSYANSFERYYPTSARGVLDANELMQNYNDKASSLLILMMIAQGAMTIPSIEARW
LTGGFTEACRISLFDLIEKNIIMAGDPIVLRAALLFTVQAAWSGDKWQMDIAMGQRGMYFSMLRHSGILDVRPPTTPHMNGNTSTDVLWKDWLQQESRSRLIYSWVMVDQDL
SLFHDTAPLFSVTEFGAPMPDSDQLWQAQTASEWSEMFNQVHEFSNGCSSVGSGARPPSLRDLFRYFLDDEIVVQDVQEIHLTPMHLRLLLHPLQTLVCQYCQLLSCFSDSV
ASRSRNRALTAASTRVRLEEVQALLGRWFDLAQRYMKANPACPMMQANLVMFHLISMNAVSNFPEIERLARREGVDGSYQQMLRMHKKCLSDVQEAITHAGQVLRLIREMP
RGIRPPWWAGAVYRAGLVFWTDSLARNESLSPNHQGAYQQPSNTAFSVDRLSADHPTILRYMSRREGIPTLTKRDGNPVPLDNGFAILSHCIDVLDEGVATRFSDGIRSKLEK
LARG

>*Alternaria carthami* (ACM_PP10841[b])

MVFCTYCGQSFTRDEHLERHILTHTNVKPFKCFTCHMSFARRDLLQRHYTVHGRNQNNEEGLPPQGIIPKSAGRTPIACSNCAKTKTKCDKKFPCTRCAQRNLKCTLRPTRRA
SKGVQRVGGPPETGSGDSSESGSNADNQITSGNVSPNHDGQSKQATAQSSPSQIQQTALMTDSQPPSQRQSQSPSASHSRNPSISHPVQPPPHMNPEEKSLHIHMPISNTP
PFFDQSPSNGLAHSSSLLSPLPTPVTGMNSFVSSTPMSGYDDFVRTVRDQPENESPQFMMDPWHSMPMDTSFDPMRIDPALMMTMGMDMTMGPPQDGILSMIPEMSPAQ
TFGQPVQTPRMVDESFSDLQIGSSASMFYPSNRHSSIADAGIPDLGAIIAAQDGWTVFRCTPSIASSSCPRTARLNLERLELSLKNHEGWANWSPSWDESDFQQGGQIAVSKL
QEATRDKLLAITQSFLHKALDIHKDGSSTSSSGESPGSTASHSNFVLLPPARVLEYFLKSYANSFERYYPTSARGVLDANELMQNYNDKASSLLILMMIAQGAMTIPSIEARWLT
GGFTEACRISLFDLIEKNIIMAGDPIVLRAALLFTVQAAWSGDKWQMDIAMGQRGMYFSMLRHSGILDVRPPTTPHMNGNTSTDVLWKDWLQQESRSRLIYSWVMVDQDLSL
FHDTAPLFSVTEFGAPMPDSDQLWQAQTASEWSEMFNQVHEFSNGYSSVGSGARPPSLRDLFRYFLDDEIVVQDVQEIHLTPMHLRLLLHPLQTLVCQYCQLLSCFSDSVAS
RSRNRALTAASTRVRLEEVQALLGRWFDLAQRYMKANPVCPMMQANLVMFHLISMNAVSNFPEIERLARREGVDGSYQQMLWMHKKCLSDVQEAITHAGQVLRLIRLVFWT
DSLAHNESLSPNHQGTYQQPSNTAFSVDRLSSDHPTILRYMSRREGIPTLTKRDGNPVPLDNGFAILSHCIDVLDEGVATRFSDGIRSKLEKLARG

>*Alternaria citriarbusti* (NA)

MVFCTYCGQSFTRDEHLERHILTHTNVKPFKCFTCHMSFARRDLLQRHYTVHGRNQNNEEGLPPQGIIPKSAGRTPIACSNCAKTKTKCDKKFPCTRCAQRNLKCTLRPTRRA
SKGVQRVGGPPETGSGDSSESGSNADNQNTSGNVSPNHDGQSKQATAQSSPSQIQQPSQISDSQPPSQRQSQSPSAPHSRNPSISHPVQPPPHMNPEEKSLHIHMPISNT
PPFFDQSPSNGLSHSSSLLSPLPTPVTGMNSFVSSTPMSGYDDFVRTVRDQPENESPQFMMDPWHSMPMDTSYDPMRIDPALMMTMGMDMTMGPPQDGILSMIPEMSPA
QTFGQPVQTPRMVDESFSDLQIGSSASMFYPSNRHSSIADAGIPDLGAIIAAQDGWTVFRCTPSIASSSCPRTARLNLERLELSLKNHEGWANWTPSWDESDFQQGGQIAVS
KLQEATRDKLLAITQSFLHKALDIHKDGSSSSSSGESPGSTASHSNFVLLPPARVLEYFLKSYANSFERYYPTSARGVLDTNELMQNYNDKASSLLILMMIAQGAMTIPSIEARW
LTGGFTEACRISLFDLIEKNIIMAGDPIVLRAALLFTVQAAWSGDKWQMDIAMGQRGMYFSMLRHSGILDVRPPTTPHMNGNTSTDVLWKDWLQQESRSRLIYSWVMVDQDL
SLFHDTAPLFSVTEFGAPMPDSDQLWQAQTAPEWSEMFNQVHEFSNGYSSVGSGARPPSLRDLFRYFLDDEIVVQDVQEIHLTPMHLRLLLHPLQTLVCQYCQLLSCFSDSV
ASRSRNRALTAASTRVRLEEVQALLGRWFDLAQRYMKANPVCPMMQANLVMFHLISMNAVSNFPEIERLARREGVDGSYQQMLWMHKKCLSDVQEAITHAGQVLRLIREMP
RGIRPPWWAGAVYRAGLVFWADSLAHNESLSPNHQGTYQPSNTAFSVDRLSADHPTILRYMSRREGIPTLTKRDGSPVPLDNGFAVLSHCIDVLDEGVATRFSDGIRSKLEKL
ARG

>*Alternaria conjuncta* (NA)

MVFCTYCGQSFTRDEHLERHILTHTNVKPFKCFTCHMSFARRDLLQRHYTVHGRNQNNEEGLPPQGIIPKSAGRTPIACSNCAKTKTKCDKKFPCTRCAQRNLKCTLRPTRRA
SKGVQRVGGPPEAGSGDSSESGSIADNHNTSGNVSPNHDSQPKQAMSKSSPAQINQTAHIPDSQPPSQRQSQSPSAPHSRNPSISQPVQPPTHINPEEKSLHLHMPISDTPP

FFDQSPSNGLAQSSSLLSPLPTPVNGMNGFVTSTPMSGYDDFVRTVRDQSDNESPQFMMDPWNGMPMDTTFDPMRIDPALMMSMSMDMSMGPPQDGILGMIPEMSPAQT

FGQPVQTPRMDESFSDLQIGSSASMFYPSNRHSSIADAGIPDLGAIIAAQDGWTVFRCTPSIASSSCPRTARLNLERLELSLKNHEGWANWSPSWDESDFQQGGQIAVSKLQ

EATRDKLLAITQSFLHKALDIHKDGSSSSSSGESPGSTASHSNFVLLPPARVLEYFLKSYANSFERYYPTSARGVLDTNELMQNYNDKASSLLILMMIAQGAMTIPSIEARWLTG

GFTEACRISLFDLIEKNIIMAGDPIVLRAALLFTVQAAWSGDKWQMDIAMGQRGMYFSMLRHSGILDVRPPATPHINGNISTDALWKDWLQQESRSRLIYSWVMVDQDLSLFHD

TAPLFSVTEFGAPMPDSDRLWQAQTASEWSEMFNQVHEFSNGYSSVGSGARPPSLRDLFRYFLDDEIVVQDVQEIHLTPMHLRLLLHPLQTLVCQYCQLLSCFSDSVASRSR

NRALTAASTRVRLEEVQALLGRWFDLAQRYMKSNPVCPMMQANLVMFHLISLNAVSNFPEIERLARREGVDGSYQQMLWMHKKCLSDVQEAITHAGQVLRLIREMPRGIRPP

WWAGAVYRAGLVFWTDSLAHGESLSPNHQGSYQPSNSAFSVDRLPADHPTILRYMSRREGIPTLTKRDGAHVPLDNGFAILSHCIDVLDEGVATRFSDGIRSKLEKLARG

>*Alternaria consortialis* (NA)

MVFCTYCGQSFTRDEHLERHILTHTNVKPFKCFTCHMSFARRDLLQRHYTVHGRNQNNEEGLPPQGIIPKSAGRTPIACSNCAKTKTKCDKKFPCTRCAQRNLKCTLRPTRRA

SKGVQRVGGPPETGSGDSSESGSNADNQNTSGNVSPNHDGQPKQATAQSSPSQIQQPSQVSDSQPPSQRQSQSPSAPHSRNPSISHPVQPPPHINPEEKSLHIHMPISNTP

PFFDQSPSNGLAHSSSLLSPLPTPVTGMNSFVSSTPMSGYDDFVRTVRDQSDNESPQFIMDPWHSMPMDTSFDPMRIDPSLIMTMGMDMTMGPPQDGILGMIPEMSPAQTF

GQPVQTPRMVDESFSDLQIGSSASMFYPSNRHSSIADAGIPDLGAIIAAQDGWTVFRCTPSIASSSCPRTARLNLERLELSLKNHEGWANWTPSWDESDFQQGGQIAVSKLH

EATRDKLLAITQSFLHKALDIHKDGSSSSSSGESPGSTASHSNFVLLPPARVLEYFLKSYANSFERYYPTSARGVLDANELMQNYNDKASSLLILMMIAQGAMTIPSIEARWLTG

GFTEACRISLFDLIEKNIIMAGDPIVLRAALLFTVQAAWSGDKWQMDIAMGQRGMYFSMLRHSGILDVRPPTTPHMNGNTSTDVLWKDWLQQESRSRLIYSWVMVDQDLSLF

HDTAPLFSVTEFGAPMPDSDQLWQAQTASEWSETFNQVHEFSNGYSSVGSGARPPSLRDLFRYFLDDEIVVQDVQEIHLTPMHLRLLLHPLQTLVCQYCQLLSCFSDSVASR

SRNRALTAASTRVRLEEVQALLGRWFDLAQRYMKANPVCPMMQANLVMFHLISMNAVSNFPEIERLARREGVDGSYQQMMWMHKKCLSDVQEAITHAGQVLRLIREMPQGI

RPPWWAGAVYRAGLVFWTDSLAHGESLSPSNHQGTYQTSNAAFSVDRLPADHPTILRYMSRRDGIPTLTKRDGNPVPLDNGFAVLSHCIDVLDDGVATRFSDGIRSKLEKLA

RG

>*Alternaria crassa* (ACM_PP08031[b])

MVFCTYCGQSFTRDEHLERHILTHTNVKPFKCFTCHMSFARRDLLQRHYTVHGRNQNNEEGLPPQGIIPKSAGRTPIACSNCAKTKTKCDKKFPCTRCAQRNLKCTLRPTRRA

SKGVQRVGGPPETGSGDSSESGSNADNQITSGNVSPNHDGQSKQATAQSSPSQIQQPALMSDSQPPSQRQSQSPSAPHSRNPSISHPVQPPPHMNPEEKSLHIHMPISNT

PPFFDQSPSNGLAHSSSLLSPLPTPVTGMNSFVSSTPMSGYDDFVRTVRDQPENESPQFMMDPWHSMPMDTNFDPMRIDPTLMMTMGMDMTMGPPQDGILSMIPEMSPA

QTFGQPVQTPRMVDESFSDLQIGSSASMFYPSNRHSSIADAGIPDLGAIIAAQDGWTVFRCTPSIASSSCPRTARLNLERLELSLKNHEGWANWAPSWDESDFQQGGQIAVS

KLQEATRDKLLAITQSFLHKALDIHKDGNSTSSSGESPGSTASHSNFVLLPPARVLEYFLKSYANSFERYYPTSARGVLDANELMQNYNDKASSLLILMMIAQGAMTIPSIEARW

LTGGFTEACRISLFDLIEKNIIMAGDPIVLRAALLFTVQAAWSGDKWQMDIAMGQRGMYFSMLRHSGILDVRPPTTPHMNGNTSTDVLWKDWLQQESRSRLIYSWVMVDQDL

SLFHDTAPLFSVTEFGAPMPDSDQLWQAQTASEWSEMFNQVHEFSNGCSSVGSGARPPSLRDLFRYFLDDEIVVQDVQEIHLTPMHLRLLLHPLQTLVCQYCQLLSCFSDSV

ASRSRNRALTAASTRVRLEEVQALLGRWFDLAQRYMKANPACPMMQANLVMFHLISMNAVSNFPEIERLARREGVDGSYQQMLRMHKKCLSDVQEAITHAGQVLRLIREMP

RGIRPPWWAGAVYRAGLVFWTDSLARNESLSPNHQGAYQQPSNTAFSVDRLSADHPTILRYMSRREGIPTLTKRDGNPVPLDNGFAILSHCIDVLDEGVATRFSDGIRSKLEK

LARG

>*Alternaria dauci* (ADC_PP01443[c])

MVFCTYCGQSFTRDEHLERHILTHTNVKPFKCFTCHMSFARRDLLQRHYTVHGRNQNNEEGLPPQGIIPKSAGRTPIACSNCAKTKTKCDKKFPCTRCAQRNLKCTLRPTRRA

SKGVQRVGGPPETGSGDSSESGSNADNQITSGNVSPNHDGQSKQATAQSSPSQIQQPALMTDSQPPSQRQSQSPSAPHSRNPSISHPVQPPPHMNPEEKSLHIHMPISNTP

PFFDQSPSNGLAHSSSLLSPLPTPVTGMNSFVSSTPMSGYDDFVRTVRDQPENESPQFMMDPWHSMPMDTNFDPMRIDPALMMTMGMDMTMGPPQDGILSMIPEMPPAQ

TFGQPVQTPRMVDESFSDLQIGSSASMFYPSNRHSSIADAGIPDLGAIIAAQDGWTVFRCTPSIASSSCPRTARLNLERLELSLKNHEGWANWAPSWDESDFQQGGQIAVSKL

QEATRDKLLAITQSFLHKALDIHKDGSSTSSSGESPGSTASHSNFVLLPPARVLEYFLKSYANSFERYYPTSARGVLDANELMQNYNDKASSLLILMMIAQGAMTIPSIEARWLT

GGFTEACRISLFDLIEKNIIMAGDPIVLRAALLFTVQAAWSGDKWQMDIAMGQRGMYFSMLRHSGILDVRPPTTPHMNGNTSTDVLWKDWLQQESRSRLIYSWVMVDQDLSL

FHDTVPLFSVTEFGAPMPDSDQLWQAQTASEWSEMFNQVHEFSNGYSSVGSGARPPSLRDLFRYFLDDEIVVQDVQEIHLTPMHLRLLLHPLQTLVCQYCQLLSCFSDSVAS

RSRNRALTAASTRVRLEEVQALLGRWFDLAQRYMKANPVCPMMQANLVMFHLISMNAVSNFPEIERLARREGVDGSYQQMLWMHKKCLSDVQEAITHAGQVLRLIREMPRG

IRPPWWAGAVYRAGLVFWTDSLAHNESLSPNHQGTYQQPSNTAFSVDRLSADHPTILRYMSRREGIPTLTKRDGNPVPLDNGFAILSHCIDVLDEGVATRFSDGIRSKLEKLA

RG

>*Alternaria destruens* (ADT_PP05662[b])

NADNQNTSGNVSPNHDGQSKQATAQSSPSQIQQPSQISDSQPPSQRQSQSPSAPHSRNPSISHPVQPPPHMNPEEKSLHIHMPISNTPPFFDQSPSNGLSHSSSLLSPLPTP

VTGMNSFVSSTPMSGYDDFVRTVRDQPENESPQFMMDPWHSMPMDTSYDPMRIDPALMMTMGMDMTMGPPQDGILSMIPEMSPAQTFGQPVQTPRMVDESFSDLQIGSS

ASMFYPSNRHSSIADAGIPDLGAIIAAQDGWTVFRCTPSIASSSCPRTARLNLERLELSLKNHEGWANWTPSWDESDFQQGGQIAVSKLQEATRDKLLAITQSFLHKALDIHKD

GSSSSSSGESPGSTASHSNFVLLPPARVLEYFLKSYANSFERYYPTSARGVLDTNELMQNYNDKASSLLILMMIAQGAMTIPSIEARWLTGGFTEASAWSGDKWQMDIAMGQ

RGMYFSMLRHSGILDVRPPTTPHMNGNTSTDVLWKDWLQQESRSRLIYSWVMVDQDLSLFHDTAPLFSVTEFGAPMPDSDQLWQAQTAPEWSEMFNQVHEFSNGYSSVG
SGARPPSLRDLFRYFLDDEIVVQDVQEIHLTPMHLRLLLHPLQTLVCQYCQLLSCFSDSVASRSRNRALTAASTRVRLEEVQALLGRWFDLAQRYMKANPVCPMMQANLVMF
HLISMNAVSNFPEIERLARREGLVFWADSLAHNESLSPNHQGTYQPSNTAFSVDRLSADHPTILRYMSRREGIPTLTKRDGSPVPLDNGFAVLSHCIDVLDEGVATRFSDGIRS
KLEKLARG

>*Alternaria ethzedia* (NA)

MVFCTYCGQSFTRDEHLERHILTHTNVKPFKCFTCHMSFARRDLLQRHYTVHGRNQNNEEGLPPQGIIPKSAGRTPIACSNCAKTKTKCDKKFPCTRCAQRNLKCTLRPTRRA
SKGVQRVGGPPEAGSGDSSESGSNADNHNTSGNVSPNHDSQPKQAMSKSSPAQINQTSHIPDSQPPSQRQSQSPSAPHSRNPSISQPVQPPTHINPEEKSLHLHMPISDTP
PFFDQSPSNGLAQSSSLLSPLPTPVNGMNGFVTSTPMSGYDDFVRTVRDQSDNESPQFMMDPWNGMPMDTTFDPMRIDPALMMSMSMDMSMGPPQDGILGMIPEMSPAQ
TFGQPVQTPRMDESFSDLQIGSSASMFYPSNRHSSIADAGIPDLGAIIAAQDGWTVFRCTPSIASSSCPRTARLNLERLELSLKNHEGWANWSPSWDESDFQQGGQIAVSKL
QEATRDKLLAITQSFLHKALDIHKDGSSSSSSGESPGSTASHSNFVLLPPARVLEYFLKSYANSFERYYPTSARGVLDTNELMQNYNDKASSLLILMMIAQGAMTIPSIEARWLT
GGFTEACRISLFDLIEKNIIMAGDPIVLRAALLFTVQAAWSGDKWQMDIAMGQRGMYFSMLRHSGILDVRPPATPHINGNISTDALWKDWLQQESRSRLIYSWVMVDQDLSLFH
DTAPLFSVTEFGAPMPDSDRLWQAQTASEWSEMFNQVHEFSNGYSSVGSGARPPSLRDLFRYFLDDEIVVQDVQEIHLTPMHLRLLLHPLQTLVCQYCQLLSCFSDSVASRS
RNRALTAASTRVRLEEVQALLGRWFDLAQRYMKSNPVCPMMQANLVMFHLISLNAVSNFPEIERLARREGVDGSYQQMLWMHKKCLSDVQEAITHAGQVLRLIREMPRGIRP
PWWAGAVYRAGLVFWTDSLAHGESLSSNHQGSYQPSNSAFSVDRLPADHPTILRYMSRREGIPTLTKRDGTHVPLDNGFAILSHCIDVLDEGVATRFSDGIRSKLEKLARG

>*Alternaria fragariae* (AFG_PP01606[c])

MVFCTYCGQSFTRDEHLERHILTHTNVKPFKCFTCHMSFARRDLLQRHYTVHGRNQNNEEGLPPQGIIPKSAGRTPIACSNCAKTKTKCDKKFPCTRCAQRNLKCTLRPTRRA
SKGVQRVGGPPETGSGDSSESGSNADNQNTSGNVSPNHDGQPKQATAQSSPSQIQQPSQISDSQPPSQRQSQSPSAPHSRNPSISHPVQPPPHMNPEEKSLHIHMPISNT
PPFFDQSPSNGLSHSSSLLSPLPTPVTGMNSFVSSTPMSGYDDFVRTVRDQPENESPQFMMDPWHSMPMDTSYDPMRIDPALMMTMGMDMTMGPPQDGILSMIPEMSPA
QTFGQPVQTPRMVDESFSDLQIGSSASMFYPSNRHSSIADAGIPDLGAIIAAQDGWTVFRCTPSIASSSCPRTARLNLERLELSLKNHEGWANWTPSWDESDFQQGGQIAVS
KLQEATRDKLLAITQSFLHKALDIHKDGSSSSSSGESPGSTASHSNFVLLPPARVLEYFLKSYANSFERYYPTSARGVLDTNELMQNYNDKASSLLILMMIAQGAMTIPSIEARW
LTGGFTEACRISLFDLIEKNIIMAGDPIVLRAALLFTVQAAWSGDKWQMDIAMGQRGMYFSMLRHSGILDVRPPTTPHMNGNTSTDVLWKDWLQQESRSRLIYSWVMVDQDL
SLFHDTAPLFSVTEFGAPMPDSDQLWQAQTAPEWSEMFNQVHEFSNGYSSVGSGARPPSLRDLFRYFLDDEIVVQDVQEIHLTPMHLRLLLHPLQTLVCQYCQLLSCFSDSV
ASRSRNRALTAASTRVRLEEVQALLGRWFDLAQRYMKANPVCPMMQANLVMFHLISMNAVSNFPEIERLARREGVDGSYQQMLWMHKKCLSDVQEAITHAGQVLRLIREMP
RGIRPPWWAGAVYRAGLVFWADSLAHNESLSPNHQGTYQPSNTAFSVDRLSADHPTILRYMSRREGIPTLTKRDGSPVPLDNGFAVLSHCIDVLDEGVATRFSDGIRSKLEKL
ARG

>*Alternaria gansuensis* (NA)

MVFCTYCGQSFTRDEHLERHILTHTNVKPFKCFTCHMSFARRDLLQRHYTVHGRNQNNEEGLPPQGIIPKSAGRTPIACSNCAKTKTKCDKKFPCTRCAQRNLKCTLRPTRRA
SKGVQRVGGPPETGSGDSSESGSNADNQNTSGNVSPNHDGQPKQATAQSSPSQLQQPGQVSDSQPSSQRQSQSPSAPHSRNTSISQPVQPPPHINPEEKSLHIHMPISNT
PPFFDQSPSNGLAQSSSLLSPLPTPVTGMNSFVSSTPMSGYDDFVRTVRDQSDNESPQFMMDPWHSMPMDTSFDSMRIDPALMMSMGMDMTMGPPQDGILSMIPEMSPA
QTFGQPVQTPRMDESFSDLQIGSSAAMFYPSNRHSSIADAGIPDLGAIIAAQDGWTVFRCTPSIASSSCPRTARLNLERLELSLKNHEGWANWTPSWDESDFQQGGQIAVSKL
QEATRDKLLAITQSFLHKALDIHKDGSSSSSSGESPGSTASHSNFVLLPPARVLEYFLKSYTNSFERYYPTSARGVLDANELMQNYNDKASSLLILMMIAQGAMTIPSIEARWLT
GGFTEACRISLFDLIEKNIILAGDPIVLRAALLFTVQAAWSGDKWQMDIAMGQRGMYFSMLRHSGILDVRPPTTPHMNGTTSTDALWKDWLQQESRSRLVYSWVMVDQDLSL
FHDTAPLFSVTEFSAPMPDSDQLWQAQTASEWSEMFNQVHEFSNGYSSVGSGARPPSLRDLFRYFLDDEIVVQDVQEIHLTPMHLRLLLHPLQTLVCQYCQLLSCFSDSVAS
RSRNRALTAASTRVRLEEVQALLGRWFDLAQRYTKANPACPMMQANLVMFHLISMNAVSNFPEIERLARREGVDGSYQQMLWMHKKCLSDVQEAVTHAGQVIRLLREMPR
GIRPPWWAGAVYRVGLVFWTDSLAHNESLSPNHQGTYQPSNAAFSVDRLPADHPTILRYMSRREGIPTLTKRDGTPVPLDNGFAVLSHCIDVLDEGVATRFSDGIRSKLEKLA
RG

>*Alternaira gaisen* (AGS_PP02526[c])

MVFCTYCGQSFTRDEHLERHILTHTNVKPFKCFTCHMSFARRDLLQRHYTVHGRNQNNEEGLPPQGIIPKSAGRTPIACSNCAKTKTKCDKKFPCTRCAQRNLKCTLRPTRRA
SKGVQRVGGPPETGSGDSSESGSNADNQNTSGNVSPNHDGQPKQATAQSSPSQIQQPSQISDSQPPSQRQSQSPSAPHSRNPSISHPVQPPPHMNPEEKSLHIHMPISNT
PPFFDQSPSNGLSHSSSLLSPLPTPVTGMNSFVSSTPMSGYDDFVRTVRDQPENESPQFMMDPWHSMPMDTSYDPMRIDPALMMTMGMDMTMGPPQDGILSMIPEMSPA
QTFGQPVQTPRMVDESFSDLQIGSSASMFYPSNRHSSIADAGIPDLGAIIAAQDGWTVFRCTPSIASSSCPRTARLNLERLELSLKNHEGWANWTPSWDESDFQQGGQIAVS
KLQEATRDKLLAITQSFLHKALDIHKDGSSSSSSGESPGSTASHSNFVLLPPARVLEYFLKSYANSFERYYPTSARGVLDTNELMQNYNDKASSLLILMMIAQGAMTIPSIEARW
LTGGFTEACRISLFDLIEKNIIMAGDPIVLRAALLFTVQAAWSGDKWQMDIAMGQRGMYFSMLRHSGILDVRPPTTPHMNGNTSTDVLWKDWLQQESRSRLIYSWVMVDQDL
SLFHDTAPLFSVTEFGAPMPDSDQLWQAQTAPEWSEMFNQVHEFSNGYSSVGSGARPPSLRDLFRYFLDDEIVVQDVQEIHLTPMHLRLLLHPLQTLVCQYCQLLSCFSDSV
ASRSRNRALTAASTRVRLEEVQALLGRWFDLAQRYMKANPVCPMMQANLVMFHLISMNAVSNFPEIERLARREGVDGSYQQMLWMHKKCLSDVQEAITHAGQVLRLIREMP

RGIRPPWWAGAVYRAGLVFWADSLAHNESLSPNHQGTYQPSNTAFSVDRLSADHPTILRYMSRREGIPTLTKRDGSPVPLDNGFAVLSHCIDVLDEGVATRFSDGIRSKLEKL

ARG

>*Alternaria hordeiaustralica* (NA)

MVFCTYCGQSFTRDEHLERHILTHTNVKPFKCFTCHMSFARRDLLQRHYTVHGRNQNNEEGLPPQGIIPKSAGRTPIACSNCAKTKTKCDKKFPCTRCAQRNLKCTLRPTRRA

SKGVQRVGGPPEAGSGDSSESGSNADNHNTSGNVSPNHDSQPKQAMSKSSPAQINQTSHIPDSQPPSQRQSQSPSAPHSRNPSISQPVQPPTHINPEEKSLHLHMPISDTP

PFFDQSPSNGLAQSSSLLSPLPTPVNGMNGFVTSTPMSGYDDFVRTVRDQSDNESPQFMMDPWNGMPMDTTFDPMRIDPALMMSMSMDMSMGPPQDGILGMIPEMSPAQ

TFGQPVQTPRMDESFSDLQIGSSASMFYPSNRHSSIADAGIPDLGAIIAAQDGWTVFRCTPSIASSSCPRTARLNLERLELSLKNHEGWANWSPSWDESDFQQGGQIAVSKL

QEATRDKLLAITQSFLHKALDIHKDGSSSSSSGESPGSTASHSNFVLLPPARVLEYFLKSYANSFERYYPTSARGVLDTNELMQNYNDKASSLLILMMIAQGAMTIPSIEARWLT

GGFTEACRISLFDLIEKNIIMAGDPIVLRAALLFTVQAAWSGDKWQMDIAMGQRGMYFSMLRHSGILDVRPPATPHINGNISTDALWKDWLQQESRSRLIYSWVMVDQDLSLFH

DTAPLFSVTEFGAPMPDSDRLWQAQTASEWSEMFNQVHEFSNGYSSVGSGARPPSLRDLFRYFLDDEIVVQDVQEIHLTPMHLRLLLHPLQTLVCQYCQLLSCFSDSVASRS

RNRALTAASTRVRLEEVQALLGRWFDLAQRYMKSNPVCPMMQANLVMFHLISLNAVSNFPEIERLARREGVDGSYQQMLWMHKKCLSDVQEAITHAGQVLRLIREMPRGIRP

PWWAGAVYRAGLVFWTDSLAHGESLSPNHQGSYQPSNSAFSVDRLPADHPTILRYMSRREGIPTLTKRDGTHVPLDNGFAILSHCIDVLDEGVATRFSDGIRSKLEKLARG

>*Alternaria incomplexa* (NA)

MVFCTYCGQSFTRDEHLERHILTHTNVKPFKCFTCHMSFARRDLLQRHYTVHGRNQNNEEGLPPQGIIPKSAGRTPIACSNCAKTKTKCDKKFPCTRCAQRNLKCTLRPTRRA

SKGVQRVGGPPEAGSGDSSESGSNADNHNTSGNVSPNHDSQPKQAVSKSSPAQIRQTSHIPDSQPPSQRQSQSPSAPHSRNPSISQPVQPPTHINPEEKSLHLHMPISDTP

PFFDQSPSNGLAQSSSLLSPLPTPVTGMNGFVTSTPMSGYDDFVRTVRDQSDNESPQFMMDPWNGMPMDTTFDPMRIDPALMMSMSMDMSMGPPQDGILGMIPEMSPAQ

AFGQPVQTPRMDESFSDLQIGSSASMFYPSNRHSSIADAGIPDLGAIIAAQDGWTVFRCTPSIASSSCPRTARLNLERLELSLKNHEGWANWSPSWDESDFQQGGQIAVSKL

QEATRDKLLAITQSFLHKALDIHKDGSSSSSSGESPGSTASHSNFVLLPPARVLEYFLKSYANSFERYYPTSARGVLDTNELMQNYNDKASSLLILMMIAQGAMTIPSIEARWLT

GGFTEACRISLFDLIEKNIIMAGDPIVLRAALLFTVQAAWSGDKWQMDIAMGQRGMYFSMLRHSGILDVRPPATPHINGNISTDALWKDWLQQESRSRLIYSWVMVDQDLSLFH

DTAPLFSVTEFGAPMPDSDRLWQAQTASEWSEMFNQVHEFSNGYSSVGSGARPPSLRDLFRYFLDDEIVVQDVQEIHLTPMHLRLLLHPLQTLVCQYCQLLSCFSDSVASRS

RNRALTAASTRVRLEEVQALLGRWFDLAQRYMKSNPVCPMMQANLVMFHLISLNAVSNFPEIERLARREGVDGSYQQMLWMHKKCLSDVQEAITHAGQVLRLIREMPRGIRP

PWWAGAVYRAGLVFWTDSLAHGESLSPNHQGSYQPSNSAFSVDRLPADHPTILRYMSRREGIPTLTKRDGTHVPLDNGFAILSHCIDVLDEGVATRFSDGIRSKLEKLARG

>*Alternaria infectoria* (KAI4920335[a])

MVFCTYCGQSFTRDEHLERHILTHTNVKPFKCFTCHMSFARRDLLQRHYTVHGRNQNNEEGLPPQGIIPKSAGRTPIACSNCAKTKTKCDKKFPCTRCAQRNLKCTLRPTRRA

SKGVQRVGGPPEAGSGDSSESGSNADNHNTSGNVSPNHDSQPKQAMSKSSPAQINQTAHIPDSQPPSQRQSQSPSAPHSRNPSISQPVQPPTHINPEEKSLHLHMPISDTP

PFFDQSPSNGLAQSSSLLSPLPTPVNGMNGFVTSTPMSGYDDFVRTVRDQSDNESPQFMMDPWNGMPMDTTFDPMRIDPALMMSMSMDMSMGPPQDGILGMIPEMSPAQ

TFGQPVQTPRMDESFSDLQIGSSASMFYPSNRHSSIADAGIPDLGAIIAAQDGWTVFRCTPSIASSSCPRTARLNLERLELSLKNHEGWANWSPSWDESDFQQGGQIAVSKL

QEATRDKLLAITQSFLHKALDIHKDGSSSSSSGESPGSTASHSNFVLLPPARVLEYFLKSYANSFERYYPTSARGVLDTNELMQNYNDKASSLLILMMIAQGAMTIPSIEARWLT

GGFTEACRISLFDLIEKNIIMAGDPIVLRAALLFTVQAAWSGDKWQMDIAMGQRGMYFSMLRHSGILDVRPPATPHINGNISTDALWKDWLQQESRSRLIYSWVMVDQDLSLFH

DTAPLFSVTEFGAPMPDSDRLWQAQTASEWSEMFNQVHEFSNGYSSVGSGARPPSLRDLFRYFLDDEIVVQDVQEIHLTPMHLRLLLHPLQTLVCQYCQLLSCFSDSVASRS

RNRALTAASTRVRLEEVQALLGRWFDLAQRYMKSNPVCPMMQANLVMFHLISLNAVSNFPEIERLARREGVDGSYQQMLWMHKKCLSDVQEAITHAGQVLRLIREMPRGIRP

PWWAGAVYRAGLVFWTDSLAHGESLSPNHQGSYQPSNSAFSVDRLPADHPTILRYMSRREGIPTLTKRDGAHVPLDNGFAILSHCIDVLDEGVATRFSDGIRSKLEKLARG

>*Alternaria limoniasperae* (NA)

MVFCTYCGQSFTRDEHLERHILTHTNVKPFKCFTCHMSFARRDLLQRHYTVHGRNQNNEEGLPPQGIIPKSAGRTPIACSNCAKTKTKCDKKFPCTRCAQRNLKCTLRPTRRA

SKGVQRVGGPPETGSGDSSESGSNADNQNTSGNVSPNHDGQSKQATAQSSPSQIQQPSQISDSQPPSQRQSQSPSAPHSRNPSISHPVQPPPHMNPEEKSLHIHMPISNT

PPFFDQSPSNGLSHSSSLLSPLPTPVTGMNSFVSSTPMSGYDDFVRTVRDQPENESPQFMMDPWHSMPMDTSYDPMRIDPALMMTMGMDMTMGPPQDGILSMIPEMSPA

QTFGQPVQTPRMVDESFSDLQIGSSASMFYPSNRHSSIADAGIPDLGAIIAAQDGWTVFRCTPSIASSSCPRTARLNLERLELSLKNHEGWANWTPSWDESDFQQGGQIAVS

KLQEATRDKLLAITQSFLHKALDIHKDGSSSSSSGESPGSTASHSNFVLLPPARVLEYFLKSYANSFERYYPTSARGVLDTNELMQNYNDKASSLLILMMIAQGAMTIPSIEARW

LTGGFTEACRISLFDLIEKNIIMAGDPIVLRAALLFTVQAAWSGDKWQMDIAMGQRGMYFSMLRHSGILDVRPPTTPHMNGNTSTDVLWKDWLQQESRSRLIYSWVMVDQDL

SLFHDTAPLFSVTEFGAPMPDSDQLWQAQTAPEWSEMFNQVHEFSNGYSSVGSGARPPSLRDLFRYFLDDEIVVQDVQEIHLTPMHLRLLLHPLQTLVCQYCQLLSCFSDSV

ASRSRNRALTAASTRVRLEEVQALLGRWFDLAQRYMKANPVCPMMQANLVMFHLISMNAVSNFPEIERLARREGVDGSYQQMLWMHKKCLSDVQEAITHAGQVLRLIREMP

RGIRPPWWAGAVYRAGLVFWADSLAHNESLSPNHQGTYQPSNTAFSVDRLSADHPTILRYMSRREGIPTLTKRDGSPVPLDNGFAVLSHCIDVLDEGVATRFSDGIRSKLEKL

ARG

>*Alternaria longipes* (NA)

MVFCTYCGQSFTRDEHLERHILTHTNVKPFKCFTCHMSFARRDLLQRHYTVHGRNQNNEEGLPPQGIIPKSAGRTPIACSNCAKTKTKCDKKFPCTRCAQRNLKCTLRPTRRA

SKGVQRVGGPPETGSGDSSESGSNADNQNTSGNVSPNHDGQSKQATAQSSPSQIQQPSQISDSQPPSQRQSQSPSAPHSRNPSISHPVQPPPHMNPEEKSLHIHMPISNT

PPFFDQSPSNGLSHSSSLLSPLPTPVTGMNSFVSSTPMSGYDDFVRTVRDQPENESPQFMMDPWHSMPMDTSYDPMRIDPALMMTMGMDMTMGPPQDGILSMIPEMSPA

QTFGQPVQTPRMVDESFSDLQIGSSASMFYPSNRHSSIADAGIPDLGAIIAAQDGWTVFRCTPSIASSSCPRTARLNLERLELSLKNHEGWANWTPSWDESDFQQGGQIAVS

KLQEATRDKLFAITQSFLHKALDIHKDGSSSSSSGESPGSTASHSNFVLLPPARVLEYFLKSYANSFERYYPTSARGVLDTNELMQNYNDKASSLLILMMIAQGAMTIPSIEARW

LTGGFTEACRISLFDLIEKNIIMAGDPIVLRAALLFTVQAAWSGDKWQMDIAMGQRGMYFSMLRHSGILDVRPPTTPHMNGNTSTDVLWKDWLQQESRSRLIYSWVMVDQDL

SLFHDTAPLFSVTEFGAPMPDSDQLWQAQTAPEWSEMFNQVHEFSNGYSSVGSGARPPSLRDLFRYFLDDEIVVQDVQEIHLTPMHLRLLLHPLQTLVCQYCQLLSCFSDSV

ASRSRNRALTAASTRVRLEEVQALLGRWFDLAQRYMKANPVCPMMQANLVMFHLISMNAVSNFPEIERLARREGVDGSYQQMLWMHKKCLSDVQEAITHAGQVLRLIREMP

RGIRPPWWAGAVYRAGLVFWADSLAHNESLSPNHQKTYQPSNTAFSVDRLSADHPTILRYMSRREGIPTLTKRDGSPVPLDNGFAILSHCIDVLDEGVATRFSDGIRSKLEKLA

RG

>*Alternaria macrospora* (NA)

MVFCTYCGQSFTRDEHLERHILTHTNVKPFKCFTCHMSFARRDLLQRHYTVHGRNQNNEEGLPPQGIIPKSAGRTPIACSNCAKTKTKCDKKFPCTRCAQRNLKCTLRPTRRA

SKGVQRVGGPPETGSGDSSESGSNADNQITSGNVSPNHDGQSKQATAQSSPSQIQQPALMSDSQPPSQRQSQSPSAPHSRNPSISHPVQPPPHMNPEEKSLHIHMPISNT

PPFFDQSPSNGLAHSSSLLSPLPTPVTGMNSFVSSTPMSGYDDFVRTVRDQPENESPQFMMDPWHSMPMDTNFDPMRIDPTLMMTMGMDMTMGPPQDGILSMIPEMSPA

QTFGQPVQTPRMVDESLSDLHIGSSASMFYPSNRHSSIADAGIPDLGAIIAAQDGWTVFRCTPSIASSSCPRTARLNLERLELSLKNHEGWANWAPSWDESDFQQGGQIAVS

KLQEATRDKLLAITQSFLHKALDIHKDGNSTSSSGESPGSTASHSNFVLLPPARVLEYFLKSYANSFERYYPTSARGVLDANELMQNYNDKASSLLILMMIAQGAMTIPSIEARW

LTGGFTEACRISLFDLIEKNIIMAGDPIVLRAALLFTVQAAWSGDKWQMDIAMGQRGMYFSMLRHSGILDVRPPTTPHMNGNTSTDVLWKDWLQQESRSRLIYSWVMVDQDL

SLFHDTAPLFSVTEFGAPMPDSDQLWQAQTASEWSEMFNQVHEFSNGYSSVGSGARPPSLRDLFRYFLDDEIVVQDVQEIHLTPMHLRLLLHPLQTLVCQYCQLLSCFSDSV

ASRSRNRALTAASTRVRLEEVQALLGRWFDLAQRYMKANLVCPMMQANLVMFHLISMNAVSNFPEIERLARREGVDGSYQQMLWMHKKCLSDVQEAITHAGQVLRLIREMP

RGIRPPWWAGAVYRAGLVFWTDSLAHNESLSPNHQGAYQQPSNTAFSVDRLSADHPTILRYMSRREGIPTLTKRDGNPVPLDNGFAILSHCIDVLDEGIATRFSDGIRSKLEKL

ARG

>*Alternaria mali* (AML_PP04777[c])

MVFCTYCGQSFTRDEHLERHILTHTNVKPFKCFTCHMSFARRDLLQRHYTVHGRNQNNEEGLPPQGIIPKSAGRTPIACSNCAKTKTKCDKKFPCTRCAQRNLKCTLRPTRRA

SKGVQRVGGPPETGSGDSSESGSNADNQNTSGNVSPNHDGQSKQATAQSSPSQIQQPSQISDSQPPSQRQSQSPSAPHSRNPSISHPVQPPPHMNPEEKSLHIHMPISNT

PPFFDQSPSNGLSHSSSLLSPLPTPVTGMNSFVSSTPMSGYDDFVRTVRDQPENESPQFMMDPWHSMPMDTSYDPMRIDPALMMTMGMDMTMGPPQDGILSMIPEMSPA

QTFGQPVQTPRMVDESFSDLQIGSSASMFYPSNRHSSIADAGIPDLGAIIAAQDGWTVFRCTPSIASSSCPRTARLNLERLELSLKNHEGWANWTPSWDESDFQQGGQIAVS

KLQEATRDKLLAITQSFLHKALDIHKDGSSSSSSGESPGSTASHSNFVLLPPARVLEYFLKSYANSFERYYPTSARGVLDTNELMQNYNDKASSLLILMMIAQGAMTIPSIEARW

LTGGFTEACRISLFDLIEKNIIMAGDPIVLRAALLFTVQAAWSGDKWQMDIAMGQRGMYFSMLRHSGILDVRPPTTPHMNGNTSTDVLWKDWLQQESRSRLIYSWVMVDQDL

SLFHDTAPLFSVTEFGAPMPDSDQLWQAQTAPEWSEMFNQVHEFSNGYSSVGSGARPPSLRDLFRYFLDDEIVVQDVQEIHLTPMHLRLLLHPLQTLVCQYCQLLSCFSDSV

ASRSRNRALTAASTRVRLEEVQALLGRWFDLAQRYMKANPVCPMMQANLVMFHLISMNAVSNFPEIERLARREGVDGSYQQMLWMHKKCLSDVQEAITHAGQVLRLIREMP

RGIRPPWWAGAVYRAGLVFWADSLAHNESLSPNHQGTYQPSNTAFSVDRLSADHPTILRYMSRREGIPTLTKRDGSPVPLDNGFAVLTHCIDVLDEGVATRFSDGIRSKLEKL

ARG

>*Alternaria metachromatica* (KAI4634631[a])

MVFCTYCGQSFTRDEHLERHILTHTNVKPFKCFTCHMSFARRDLLQRHYTVHGRNQNNEEGLPPQGIIPKSAGRTPIACSNCAKTKTKCDKKFPCTRCAQRNLKCTLRPTRRA

SKGVQRVGGPPEAGSGDSSESGSNADNHNTSGNVSPNHDSQPKQAMSKSSPAQINQTAHIPDSQPPSQRQSQSPSAPHSRNPSISQPVQPPTHINPEEKSLHLHMPISDTP

PFFDQSPSNGLAQSSSLLSPLPTPVNGMNGFVTSTPMSGYDDFVRTVRDQSDNESPQFMMDPWNGMPMDTTFDPMRIDPALMMSMSMDMSMGPPQDGILGMIPEMSPAQ

TFGQPVQTPRMDESFSDLQIGSSASMFYPSNRHSSIADAGIPDLGAIIAAQDGWTVFRCTPSIASSSCPRTARLNLERLELSLKNHEGWANWSPSWDESDFQQGGQIAVSKL

QEATRDKLLAITQSFLHKALDIHKDGSSSSSSGESPGSTASHSNFVLLPPARVLEYFLKSYANSFERYYPTSARGVLDTNELMQNYNDKASSLLILMMIAQGAMTIPSIEARWLT

GGFTEACRISLFDLIEKNIIMAGDPIVLRAALLFTVQAAWSGDKWQMDIAMGQRGMYFSMLRHSGILDVRPPATPHINGNISTDALWKDWLQQESRSRLIYSWVMVDQDLSLFH

DTAPLFSVTEFGAPMPDSDRLWQAQTASEWSEMFNQVHEFSNGYSSVGSGARPPSLRDLFRYFLDDEIVVQDVQEIHLTPMHLRLLLHPLQTLVCQYCQLLSCFSDSVASRS

RNRALTAASTRVRLEEVQALLGRWFDLAQRYMKSNPVCPMMQANLVMFHLISLNAVSNFPEIERLARREGVDGSYQQMLWMHKKCLSDVQEAITHAGQVLRLIREMPRGIRP

PWWAGAVYRAGLVFWTDSLAHGESLSPNHQGSYQPSNSAFSVDRLPADHPTILRYMSRREGIPTLTKRDGTHVPLDNGFAILSHCIDVLDEGVATRFSDGIRSKLEKLARG

>*Alternaria novae-zelandiae* (KAI4692986[a])

MVFCTYCGQSFTRDEHLERHILTHTNVKPFKCFTCHMSFARRDLLQRHYTVHGRNQNNEEGLPPQGIIPKSAGRTPIACSNCAKTKTKCDKKFPCTRCAQRNLKCTLRPTRRA

SKGVQRVGGPPEAGSGDSSESGSNADNHNTSGNVSPNHDSQPKQAMSKSSPAQINQTAHIPDSQPPSQRQSQSPSAPHSRNPSISQPVQPPTHINPEEKSLHLHMPISDTP

PFFDQSPSNGLAQSSSLLSPLPTPVNGMNGFVTSTPMSGYDDFVRTVRDQSDNESPQFMMDPWNGMPMDTTFDPMRIDPALMMSMSMDMSMGPPQDGILGMIPEMSPAQ

TFGQPVQTPRMDESFSDLQIGSSASMFYPSNRHSSIADAGIPDLGAIIAAQDGWTVFRCTPSIASSSCPRTARLNLERLELSLKNHEGWANWSPSWDESDFQQGGQIAVSKL

QEATRDKLLAITQSFLHKALDIHKDGSSSSSSGESPGSTASHSNFVLLPPARVLEYFLKSYANSFERYYPTSARGVLDTNELMQNYNDKASSLLILMMIAQGAMTIPSIEARWLT

GGFTEACRISLFDLIEKNIIMAGDPIVLRAALLFTVQAAWSGDKWQMDIAMGQRGMYFSMLRHSGILDVRPPATPHINGNISTDALWKDWLQQESRSRLIYSWVMVDQDLSLFH

DTAPLFSVTEFGAPMPDSDRLWQAQTASEWSEMFNQVHEFSNGYSSVGSGARPPSLRDLFRYFLDDEIVVQDVQEIHLTPMHLRLLLHPLQTLVCQYCQLLSCFSDSVASRS

RNRALTAASTRVRLEEVQALLGRWFDLAQRYMKSNPVCPMMQANLVMFHLISLNAVSNFPEIERLARREGVDGSYQQMLWMHKKCLSDVQEAITHAGQVLRLIREMPRGIRP

PWWAGAVYRAGLVFWTDSLAHGESLSPNHQGSYQPSNSAFSVDRLPADHPTILRYMSRREGIPTLTKRDGAHVPLDNGFAILSHCIDVLDEGVATRFSDGIRSKLEKLARG

>*Alternaria panax* (KAG9192434[a])

MVFCTYCGQSFTRDEHLERHILTHTNVKPFKCFTCHMSFARRDLLQRHYTVHGRNQNNEEGLPPQGIIPKSAGRTPIACSNCAKTKTKCDKKFPCTRCAQRNLKCTLRPTRRA

SKGVQRVGGPPETGSGDSSESGSNADNQNTSGNVSPSHNGQQKQATAQSSPSQIQQPGQVSDSQPPSQRQSQSPSAPHSRNPSISHPVPPPPHINPEEKSLHIHMPISNT

PPFFDQSPSNGLAHSSSLLSPLPTPVTGMNSFVSPTPMSGYDDFVRTVRDQSDNESPQFVMDPWHSMPMDTTFDPMRIDPALMMTMGMDMTMGPPQDGILSMIPEMSPA

QTFGQPIQTPRMVDESFSDLQIGSSASVFYSSNRHSSIADAGIPDLGAIIAAQDGWTVFRCTPSIASSSCPRTARLNLERLELSLKNHEGWANWTPSWDESDYQQGGQVVVSK

LHEATRDKLLAITQSFLHKALDIHKDGNSSSSSGESPGSTASHSNFVLLPPARVLEYFLKSYANSFERYYPTSARGVLDTNELMQNYNDKASSLLILMMIAQGAMTIPSIEARWL

TGGFTEACRISLFDLIEKNIIMAGDPIVLRAALLFTVQAAWSGDKWQMDIAMGQRGMYFSMLRHSGILDVRPPTTPHMNGNTSTDVLWKDWLQQESRSRLIYSWVMVDQDLS

LFHDTAPLFSVTEFGAPMPDSDQLWQAQTASEWSEMFNQVHEFSNGYSSVGSGARPPSLRDLFRYFLDDEIVVQDVQEIHLTPMHLRLLLHPLQTLVCQYCQLLSCFSDSVA

PRSRNRALTAASTRVRLEEVQALLGRWFNLAQRYMKANPVCPMMQANLVMFHLISMNAVSNFPEIERLARREGVDGSYQQMLWIHKKCLSDVQEAITHAGQVIRLIREMPRG

IRPPWWAGAVYRAGLVFWTDSLSHNEALSPSHQNTYQQSSNTAFAVDRLPADHPTILRYMSRREGIPTLTKRDGTSVPLDNGFAVLSHCIDVLDEGVATRFSDGIRSKLEKLA

RG

>*Alternaria porri* (APR_PP04911[c])

MVFCTYCGQSFTRDEHLERHILTHTNVKPFKCFTCHMSFARRDLLQRHYTVHGRNQNNEEGLPPQGIIPKSAGRTPIACSNCAKTKTKCDKKFPCTRCAQRNLKCTLRPTRRA

SKGVQRVGGPPETGSGDSSESGSNADNQITSGNVSPNHDGQSKQATAQSSPSQIQQTALMTDSQPPSQRQSQSPSASHSRNPSISHPVQPPPHMNPEEKSLHIHMPISNTP

PFFDQSPSNGLAHSSSLLSPLPTPVTGMNSFVSSTPMSGYDDFVRTVRDQPENESPQFMMDPWHSMPMDTSFDPMRIDPALMMTMGMDMTMGPPQDGILSMIPEMSPAQ

TFGQPVQTPRMVDESFSDLQIGSSASMFYPSNRHSSIADAGIPDLGAIIAAQDGWTVFRCTPSIASSSCPRTARLNLERLELSLKNHEGWANWSPSWDESDFQQGGQIAVSKL

QEATRDKLLAITQSFLHKALDIHKDGSSTSSSGESPGSTASHSNFVLLPPARVLEYFLKSYANSFERYYPTSARGVLDANELMQNYNDKASSLLILMMIAQGAMTIPSIEARWLT

GGFTEACRISLFDLIEKNIIMAGDPIVLRAALLFTVQAAWSGDKWQMDIAMGQRGMYFSMLRHSGILDVRPPTTPHMNGNTSTDVLWKDWLQQESRSRLIYSWVMVDQDLSL

FHDTAPLFSVTEFGAPMPDSDQLWQAQTASEWSEMFNQVHEFSNGYSSVGSGARPPSLRDLFRYFLDDEIVVQDVQEIHLTPMHLRLLLHPLQTLVCQYCQLLSCFSDSVAS

RSRNRALTAASTRVRLEEVQALLGRWFDLAQRYMKANPVCPMMQANLVMFHLISMNAVSNFPEIERLARREGVDGSYQQMLWMHKKCLSDVQEAITHAGQVLRLIREMPRG

IRPPWWAGAVYRAGLVFWTDSLAHNESLSPNHQGTYQQPSNTAFSVDRLSSDHPTILRYMSRREGIPTLTKRDGNPVPLDNGFAILSHCIDVLDEGVATRFSDGIRSKLEKLA

RG

>*Alternaria postmessia* (NA)

MVFCTYCGQSFTRDEHLERHILTHTNVKPFKCFTCHMSFARRDLLQRHYTVHGRNQNNEEGLPPQGIIPKSAGRTPIACSNCAKTKTKCDKKFPCTRCAQRNLKCTLRPTRRA

SKGVQRVGGPPETGSGDSSESGSNADNQNTSGNVSPNHDGQSKQATAQSSPSQIQQPSQISDSQPPSQRQSQSPSAPHSRNPSISHPVQPPPHMNPEEKSLHIHMPISNT

PPFFDQSPSNGLSHSSSLLSPLPTPVTGMNSFVSSTPMSGYDDFVRTVRDQPENESPQFMMDPWHSMPMDTSYDPMRIDPALMMTMGMDMTMGPPQDGILSMIPEMSPA

QTFGQPVQTPRMVDESFSDLQIGSSASMFYPSNRHSSIADAGIPDLGAIIAAQDGWTVFRCTPSIASSSCPRTARLNLERLELSLKNHEGWANWTPSWDESDFQQGGQIAVS

KLQEATRDKLLAITQSFLHKALDIHKDGSSSSSSGESPGSTASHSNFVLLPPARVLEYFLKSYANSFERYYPTSARGVLDTNELMQNYNDKASSLLILMMIAQGAMTIPSIEARW

LTGGFTEACRISLFDLIEKNIIMAGDPIVLRAALLFTVQAAWSGDKWQMDIAMGQRGMYFSMLRHSGILDVRPPTTPHMNGNTSTDVLWKDWLQQESRSRLIYSWVMVDQDL

SLFHDTAPLFSVTEFGAPMPDSDQLWQAQTAPEWSEMFNQVHEFSNGYSSVGSGARPPSLRDLFRYFLDDEIVVQDVQEIHLTPMHLRLLLHPLQTLVCQYCQLLSCFSDSV

ASRSRNRALTAASTRVRLEEVQALLGRWFDLAQRYMKANPVCPMMQANLVMFHLISMNAVSNFPEIERLARREGVDGSYQQMLWMHKKCLSDVQEAITHAGQVLRLIREMP

RGIRPPWWAGAVYRAGLVFWADSLAHNESLSPNHQGTYQPSNTAFSVDRLSADHPTILRYMSRREGIPTLTKRDGSPVPLDNGFAVLSHCIDVLDEGVATRFSDGIRSKLEKL

ARG

>*Alternaria rosae* (XP_046026581[a])

MVFCTYCGQSFTRDEHLERHILTHTNVKPFKCFTCHMSFARRDLLQRHYTVHGRNQNNEEGLPPQGIIPKSAGRTPIACSNCAKTKTKCDKKFPCTRCAQRNLKCTLRPTRRA

SKGVQRVGGPPEAGSGDSSESGSNADVHNTSGNVSPNHDSQPKPAMSKSSPSQIHQASHVPDSQPPSQRQSQSPSAPHSRNPSISQPVQPPTHINPEEKSLHLHMPISDTP

PFFDQSPSNGLAQSSSLLSPLPTPVTGMNGFVTSTPMSGYDDFVRTVRDQSDNESPQFMMDPWNGMPMDTTFDPMRIDPALMMSMSMDMSMGPPQDGILGMIPEMSPAQ

TFGQPVQTPRMDESFSDLQIGSSASMFYPSNRHSSIADAGIPDLGAIIAAQDGWTVFRCTPSIASSSCPRTARLNLERLELSLKNHEGWANWSPSWDESDFQQGGQIAVSKL

QEATRDKLLAITQSFLHKALDIHKDGSSSSSSGESPGSTASHSNFVLLPPARVLEYFLKSYANSFERYYPTSARGVLDTNELMQNYNDKASSLLILMMIAQGAMTIPSIEARWLT

GGFTEACRISLFDLIEKNIIMAGDPIVLRAALLFTVQAAWSGDKWQMDIAMGQRGMYFSMLRHSGILDVRPPATPHVNGNISTDALWKDWLQQESRSRLIYSWVMVDQDLSLF

HDTAPLFSVTEFGAPMPDSDRLWQAQTASEWSEMFNQVHEFSNGYSSVGSGARPPSLRDLFRYFLDDEIVVQDVQEIHLTPMHLRLLLHPLQTLVCQYCQLLSCFSDSVASR

SRNRALTAASTRVRLEEVQALLGRWFDLAQRYMKSNPVCPMMQANLVMFHLISLNAVSNFPEIERLARREGVDGSYQQMLWMHKKCLSDVQEAITHAGQVLRLIREMPRGIR

PPWWAGAVYRAGLVFWTDSLAHGESLSPNHQGSYQPSNSAFSVDRLPSDHPTILRYMSRREGIPTLTKRDGTHVPLDNGFAILSHCIDVLDEGVATRFSDGIRSKLEKLARG

>*Alternaria solani* (ASL_PP00349[b])

MVFCTYCGQSFTRDEHLERHILTHTNVKPFKCFTCHMSFARRDLLQRHYTVHGRNQNNEEGLPPQGIIPKSAGRTPIACSNCAKTKTKCDKKFPCTRCAQRNLKCTLRPTRRA

SKGVQRVGGPPETGSGDSSESGSNADNQITSGNVSPNHDGQSKQATAQSSPSQIQQTALMTDSQPPSQRQSQSPSASHSRNPSISHPVQPPPHMNPEEKSLHIHMPISNTP

PFFDQSPSNGLAHSSSLLSPLPTPVTGMNSFVSSTPMSGYDDFVRTVRDQPENESPQFMMDPWHSMPMDTSFDPMRIDPALMMTMGMDMTMGPPQDGILSMIPEMSPAQ

TFGQPVQTPRMVDESFSDLQIGSSASMFYPSNRHSSIADAGIPDLGAIIAAQDGWTVFRCTPSIASSSCPRTARLNLERLELSLKNHEGWANWSPSWDESDFQQGGQIAVSKL

QEATRDKLLAITQSFLHKALDIHKDGSSTSSSGESPGSTASHSNFVLLPPARVLEYFLKSYANSFERYYPTSARGVLDANELMQNYNDKASSLLILMMIAQGAMTIPSIEARWLT

GGFTEACRISLFDLIEKNIIMAGDPIVLRAALLFTVQAAWSGDKWQMDIAMGQRGMYFSMLRHSGILDVRPPTTPHMNGNTSTDVLWKDWLQQESRSRLIYSWVMVDQDLSL

FHDTAPLFSVTEFGAPMPDSDQLWQAQTASEWSEMFNQVHEFSNGYSSVGSGARPPSLRDLFRYFLDDEIVVQDVQEIHLTPMHLRLLLHPLQTLVCQYCQLLSCFSDSVAS

RSRNRALTAASTRVRLEEVQALLGRWFDLAQRYMKANPVCPMMQANLVMFHLISMNAVSNFPEIERLARREGVDGSYQQMLWMHKKCLSDVQEAITHAGQVLRLIREMPRG

IRPPWWAGAVYRAGLVFWTDSLAHNESLSPNHQGTYQQPSNTAFSVDRLSSDHPTILRYMSRREGIPTLTKRDGNPVPLDNGFAILSHCIDVLDEGVATRFSDGIRSKLEKLA

RG

>*Alternaria tagetica* (ATT_PP04326[b])

MVFCTYCGQSFTRDEHLERHILTHTNVKPFKCFTCHMSFARRDLLQRHYTVHGRNQNNEEGLPPQGIIPKSAGRTPIACSNCAKTKTKCDKKFPCTRCAQRNLKCTLRPTRRA

SKGVQRVGGPPETGSGDSSESGSNADNQITSGNVSPNHDGQSKQATAQSSPSQIQQTVLMTDSQPPSQRQSQSPSAPHSRNPSISHPVQPPPHMNPEEKSLHIHMPISNTP

PFFDQSPSNGLAHSSSLLSPLPTPVTGMNSFVSSTPMSGYDDFVRTVRDQSENESPQFMMDPWHSMPMDTSFDPMRIDPALMMTMGMDMTMGPPQDGILSMIPEMSPAQ

TFGQPVQTPRMVEESFSDLQIGSSASMFYPSNRHSSIADAGIPDLGAIIAAQDGWTVFRCTPSIASSSCPRTARLNLERLELSLKNHEGWANWAPSWDESDFQQGGQIAVSKL

QEATRDKLLAITQSFLHKALDIHKDGSSTSSSGESPGSTASHSNFVLLPPARVLEYFLKSYANSFERYYPTSARGVLDANELMQNYNDKASSLLILMMIAQGAMTIPSIEARWLT

GGFTEACRISLFDLIEKNIIMAGDPIVLRAALLFTVQAAWSGDKWQMDIAMGQRGMYFSMLRHSGILDVRPPTTPHMNGNTSTDVLWKDWLQQESRSRLIYSWVMVDQDLSL

FHDTAPLFSVTEFGAPMPDSDQLWQAQTASEWSEMFNQVHEFSNGYSSVGSGARPPSLRDLFRYFLDDEIVVQDVQEIHLTPMHLRLLLHPLQTLVCQYCQLLSCFSDSVAS

RSRNRALTAASTRVRLEEVQALLGRWFDLAQRYMKANPVCPMMQANLVMFHLISMNAVSNFPEIERLARREGVDGSYQQMLWMHKKCLSDVQEAITHAGQVLRLIRDMPRG

IRPPWWAGAVYRAGLVFWTDSLAHNESLSPNHQGTYQQPSNTAFSVDRLSADHPTILRYMSRREGIPTLTKRDGNPVPLDNGFAILSHCIDVLDEGVATRFSDGIRSKLEKLA

RG

>*Alternaria tangelonis* (NA)

MVFCTYCGQSFTRDEHLERHILTHTNVKPFKCFTCHMSFARRDLLQRHYTVHGRNQNNEEGLPPQGIIPKSAGRTPIACSNCAKTKTKCDKKFPCTRCAQRNLKCTLRPTRRA

SKGVQRVGGPPETGSGDSSESGSNADNQNTSGNVSPNHDGQSKQATAQSSPSQIQQPSQISDSQPPSQRQSQSPSAPHSRNPSISHPVQPPPHMNPEEKSLHIHMPISNT

PPFFDQSPSNGLSHSSSLLSPLPTPVTGMNSFVSSTPMSGYDDFVRTVRDQPENESPQFMMDPWHSMPMDTSYDPMRIDPALMMTMGMDMTMGPPQDGILSMIPEMSPA

QTFGQPVQTPRMVDESFSDLQIGSSASMFYPSNRHSSIADAGIPDLGAIIAAQDGWTVFRCTPSIASSSCPRTARLNLERLELSLKNHEGWANWTPSWDESDFQQGGQIAVS

KLQEATRDKLLAITQSFLHKALDIHKDGSSSSSSGESPGSTASHSNFVLLPPARVLEYFLKSYANSFERYYPTSARGVLDTNELMQNYNDKASSLLILMMIAQGAMTIPSIEARW

LTGGFTEACRISLFDLIEKNIIMAGDPIVLRAALLFTVQAAWSGDKWQMDIAMGQRGMYFSMLRHSGILDVRPPTTPHMNGNTSTDVLWKDWLQQESRSRLIYSWVMVDQDL

SLFHDTAPLFSVTEFGAPMPDSDQLWQAQTAPEWSEMFNQVHEFSNGYSSVGSGARPPSLRDLFRYFLDDEIVVQDVQEIHLTPMHLRLLLHPLQTLVCQYCQLLSCFSDSV

ASRSRNRALTAASTRVRLEEVQALLGRWFDLAQRYMKANPVCPMMQANLVMFHLISMNAVSNFPEIERLARREGVDGSYQQMLWMHKKCLSDVQEAITHAGQVLRLIREMP

RGIRPPWWAGAVYRAGLVFWADSLAHNESLSPNHQKTYQPSNTAFSVDRLSADHPTILRYMSRREGIPTLTKRDGSPVPLDNGFAVLSHCIDVLDEGVATRFSDGIRSKLEKL

ARG

>*Alternaria tenuissima* (NA)

MVFCTYCGQSFTRDEHLERHILTHTNVKPFKCFTCHMSFARRDLLQRHYTVHGRNQNNEEGLPPQGIIPKSAGRTPIACSNCAKTKTKCDKKFPCTRCAQRNLKCTLRPTRRA

SKGVQRVGGPPETGSGDSSESGSNADNQNTSGNVSPNHDGQSKQATAQSSPSQIQQPSQISDSQPPSQRQSQSPSAPHSRNPSISHPVQPPPHMNPEEKSLHIHMPISNT

PPFFDQSPSNGLSHSSSLLSPLPTPVTGMNSFVSSTPMSGYDDFVRTVRDQPENESPQFMMDPWHSMPMDTSYDPMRIDPALMMTMGMDMTMGPPQDGILSMIPEMSPA

QTFGQPVQTPRMVDESFSDLQIGSSASMFYPSNRHSSIADAGIPDLGAIIAAQDGWTVFRCTPSIASSSCPRTARLNLERLELSLKNHEGWANWTPSWDESDFQQGGQIAVS

KLQEATRDKLLAITQSFLHKALDIHKDGSSSSSSGESPGSTASHSNFVLLPPARVLEYFLKSYANSFERYYPTSARGVLDTNELMQNYNDKASSLLILMMIAQGAMTIPSIEARW

LTGGFTEACRISLFDLIEKNIIMAGDPIVLRAALLFTVQAAWSGDKWQMDIAMGQRGMYFSMLRHSGILDVRPPTTPHMNGNTSTDVLWKDWLQQESRSRLIYSWVMVDQDL

SLFHDTAPLFSVTEFGAPMPDSDQLWQAQTAPEWSEMFNQVHEFSNGYSSVGSGARPPSLRDLFRYFLDDEIVVQDVQEIHLTPMHLRLLLHPLQTLVCQYCQLLSCFSDSV

ASRSRNRALTAASTRVRLEEVQALLGRWFDLAQRYMKANPVCPMMQANLVMFHLISMNAVSNFPEIERLARREGVDGSYQQMLWMHKKCLSDVQEAITHAGQVLRLIREMP

RGIRPPWWAGAVYRAGLVFWADSLAHNESLSPNHQGTYQPSNTAFSVDRLSADHPTILRYMSRREGIPTLTKRDGSPVPLDNGFAVLSHCIDVLDEGVATRFSDGIRSKLEKL

ARG

>*Alternaria tomatophila* (ATM_PP05196[b])

MVFCTYCGQSFTRDEHLERHILTHTNVKPFKCFTCHMSFARRDLLQRHYTVHGRNQNNEEGLPPQGIIPKSAGRTPIACSNCAKTKTKCDKKFPCTRCAQRNLKCTLRPTRRA

SKGVQRVGGPPETGSGDSSESGSNADNQITSGNVSPNHDGQSKQATAQSSPSQIQQTALMTDSQPPSQRQSQSPSASHSRNPSISHPVQPPPHMNPEEKSLHIHMPISNTP

PFFDQSPSNGLAHSSSLLSPLPTPVTGMNSFVSSTPMSGYDDFVRTVRDQPENESPQFMMDPWHSMPMDTSFDPMRIDPALMMTMGMDMTMGPPQDGILSMIPEMSPAQ

TFGQPVQTPRMVDESFSDLQIGSSASMFYPSNRHSSIADAGIPDLGAIIAAQDGWTVFRCTPSIASSSCPRTARLNLERLELSLKNHEGWANWSPSWDESDFQQGGQIAVSKL

QEATRDKLLAITQSFLHKALDIHKDGSSTSSSGESPGSTASHSNFVLLPPARVLEYFLKSYANSFERYYPTSARGVLDANELMQNYNDKASSLLILMMIAQGAMTIPSIEARWLT

GGFTEACRISLFDLIEKNIIMAGDPIVLRAALLFTVQAAWSGDKWQMDIAMGQRGMYFSMLRHSGILDVRPPTTPHMNGNTSTDVLWKDWLQQESRSRLIYSWVMVDQDLSL

FHDTAPLFSVTEFGAPMPDSDQLWQAQTASEWSEMFNQVHEFSNGYSSVGSGARPPSLRDLFRYFLDDEIVVQDVQEIHLTPMHLRLLLHPLQTLVCQYCQLLSCFSDSVAS

RSRNRALTAASTRVRLEEVQALLGRWFDLAQRYMKANPVCPMMQANLVMFHLISMNAVSNFPEIERLARREGVDGSYQQMLWMHKKCLSDVQEAITHAGQVLRLIREMPRG

IRPPWWAGAVYRAGLVFWTDSLAHNESLSPNHQGTYQQPSNTAFSVDRLSSDHPTILRYMSRREGIPTLTKRDGNPVPLDNGFAILSHCIDVLDEGVATRFSDGIRSKLEKLA

RG

>*Alternaria triticina* (NA)

MVFCTYCGQSFTRDEHLERHILTHTNVKPFKCFTCHMSFARRDLLQRHYTVHGRNQNNEEGLPPQGIIPKSAGRTPIACSNCAKTKTKCDKKFPCTRCAQRNLKCTLRPTRRA

SKGVQRVGGPPEAGSGDSSESGSNADNHNTSGNVSPNHDSQPKQAMSKSSPAQINQTSHIPDSQPPSQRQSQSPSAPHSRNPSISQPVQPPTHINPEEKSLHLHMPISDTP

PFFDQSPSNGLAQSSSLLSPLPTPVNGMNGFVTSTPMSGYDDFVRTVRDQSDNESPQFMMDPWNGMPMDTTFDPMRIDPALMMSMSMDMSMGPPQDGILGMIPEMSPAQ

TFGQPVQTPRMDESFSDLQIGSSASMFYPSNRHSSIADAGIPDLGAIIAAQDGWTVFRCTPSIASSSCPRTARLNLERLELSLKNHEGWANWSPSWDESDFQQGGQIAVSKL

QEATRDKLLAITQSFLHKALDIHKDGSSSSSSGESPGSTASHSNFVLLPPARVLEYFLKSYANSFERYYPTSARGVLDTNELMQNYNDKASSLLILMMLAQGAMTIPSIEARWLT

GGFTEACRISLFDLIEKNIIMAGDPIVLRAALLFTVQAAWSGDKWQMDIAMGQRGMYFSMLRHSGILDVRPPATPHINGNISTDALWKDWLQQESRSRLIYSWVMVDQDLSLFH

DTAPLFSVTEFGAPMPDSDRLWQAQTASEWSEMFNQVHEFSNGYSSVGSGARPPSLRDLFRYFLDDEIVVQDVQEIHLTPMHLRLLLHPLQTLVCQYCQLLSCFSDSVASRS

RNRALTAASTRVRLEEVQALLGRWFDLAQRYMKSNPVCPMMQANLVMFHLISLNAVSNFPEIERLARREGVDGSYQQMLWMHKKCLSDVQEAITHAGQVLRLIREMPRGIRP

PWWAGAVYRAGLVFWTDSLAHGESLSPNHQGSYQPSNSAFSVDRLPADHPTILRYMSRREGIPTLTKRDGTHVPLDNGFAILSHCIDVLDEGVATRFSDGIRSKLEKLARG

>*Alternaria triticimaculans* (NA)

MVFCTYCGQSFTRDEHLERHILTHTNVKPFKCFTCHMSFARRDLLQRHYTVHGRNQNNEEGLPPQGIIPKSAGRTPIACSNCAKTKTKCDKKFPCTRCAQRNLKCTLRPTRRA

SKGVQRVGGPPEAGSGDSSESGSNADNHNTSGNVSPNHDSQPKQAMSKSSPAQINQTSHIPDSQPPSQRQSQSPSAPHSRNPSISQPVQPPTHINPEEKSLHLHMPISDTP

PFFDQSPSNGLAQSSSLLSPLPTPVNGMNGFVTSTPMSGYDDFVRTVRDQSDNESPQFMMDPWNGMPMDTTFDPMRIDPALMMSMSMDMSMGPPQDGILGMIPEMSPAQ

TFGQPVQTPRMDESFSDLQIGSSASMFYPSNRHSSIADAGIPDLGAIIAAQDGWTVFRCTPSIASSSCPRTARLNLERLELSLKNHEGWANWSPSWDESDFQQGGQIAVSKL

QEATRDKLLAITQSFLHKALDIHKDGSSSSSSGESPGSTASHSNFVLLPPARVLEYFLKSYANSFERYYPTSARGVLDTNELMQNYNDKASSLLILMMIAQGAMTIPSIEARWLT

GGFTEACRISLFDLIEKNIIMAGDPIVLRAALLFTVQAAWSGDKWQMDIAMGQRGMYFSMLRHSGILDVRPPATPHINGNISTDALWKDWLQQESRSRLIYSWVMVDQDLSLFH

DTAPLFSVTEFGAPMPDSDRLWQAQTASEWSEMFNQVHEFSNGYSSVGSGARPPSLRDLFRYFLDDEIVVQDVQEIHLTPMHLRLLLHPLQTLVCQYCQLLSCFSDSVASRS

RNRALTAASTRVRLEEVQALLGRWFDLAQRYMKSNPVCPMMQANLVMFHLISLNAVSNFPEIERLARREGVDGSYQQMLWVHKKCLSDVQEAITHAGQVLRLIREMPRGIRP

PWWAGAVYRAGLVFWTDSLAHGESLSPNHQGSYQPSNSAFSVDRLPADHPTILRYMSRREGIPTLTKRDGTHVPLDNGFAILSHCIDVLDEGVATRFSDGIRSKLEKLARG

>*Alternaria turkisafria* (NA)

MVFCTYCGQSFTRDEHLERHILTHTNVKPFKCFTCHMSFARRDLLQRHYTVHGRNQNNEEGLPPQGIIPKSAGRTPIACSNCAKTKTKCDKKFPCTRCAQRNLKCTLRPTRRA

SKGVQRVGGPPETGSGDSSESGSNADNQNTSGNVSPNHDGQSKQATAQSSPSQIQQPSQISDSQPPSQRQSQSPSAPHSRNPSISHPVQPPPHMNPEEKSLHIHMPISNT

PPFFDQSPSNGLSHSSSLLSPLPTPVTGMNSFVSSTPMSGYDDFVRTVRDQPENESPQFMMDPWHSMPMDTSYDPMRIDPALMMTMGMDMTMGPPQDGILSMIPEMSPA

QTFGQPVQTPRMVDESFSDLQIGSSASMFYPSNRHSSIADAGIPDLGAIIAAQDGWTVFRCTPSIASSSCPRTARLNLERLELSLKNHEGWANWTPSWDESDFQQGGQIAVS

KLQEATRDKLLAITQSFLHKALDIHKDGSSSSSSGESPGSTASHSNFVLLPPARVLEYFLKSYANSFERYYPTSARGVLDTNELMQNYNDKASSLLILMMIAQGAMTIPSIEARW

LTGGFTEACRISLFDLIEKNIIMAGDPIVLRAALLFTVQAAWSGDKWQMDIAMGQRGMYFSMLRHSGILDVRPPTTPHMNGNTSTDVLWKDWLQQESRSRLIYSWVMVDQDL

SLFHDTAPLFSVTEFGAPMPDSDQLWQAQTAPEWSEMFNQVHEFSNGYSSVGSGARPPSLRDLFRYFLDDEIVVQDVQEIHLTPMHLRLLLHPLQTLVCQYCQLLSCFSDSV

ASRSRNRALTAASTRVRLEEVQALLGRWFDLAQRYMKANPVCPMMQANLVMFHLISMNAVSNFPEIERLARREGVDGSYQQMLWMHKKCLSDVQEAITHAGQVLRLIREMP

RGIRPPWWAGAVYRAGLVFWADSLAHNESLSPNHQGTYQPSNTAFSVDRLSADHPTILRYMSRREGIPTLTKRDGSPVPLDNGFAVLSHCIDVLDEGVATRFSDGIRSKLEKL

ARG

>*Alternaria ventricosa* (NA)

MVFCTYCGQSFTRDEHLERHILTHTNVKPFKCFTCHMSFARRDLLQRHYTVHGRNQNNEEGLPPQGIIPKSAGRTPIACSNCAKTKTKCDKKFPCTRCAQRNLKCTLRPTRRA

SKGVQRVGGPPEAGSGDSSESGSNADNHNTSGNVSPNHDSQPKQTMSKSSPAQINQTSHIPDSQPPSQRQSQSPSAPHSRNPSISQPVQPPTHINPEEKSLHLHMPISDTP

PFFDQSPSNGLAQSSSLLSPLPTPVNGMNGFVTSTPMSGYDDFVRTVRDQSDNESPQFMMDPWNGMPMDTTFDPMRIDPALMMSMSMDMSMGPPQDGILGMIPEMSPAQ

TFGQPVQTPRMDESFSDLQIGSSASMFYPSNRHSSIADAGIPDLGAIIAAQDGWTVFRCTPSIASSSCPRTARLNLERLELSLKNHEGWANWSPSWDESDFQQGGQIAVSKL

QEATRDKLLAITQSFLHKALDIHKDGSSSSSSGESPGSTASHSNFVLLPPARVLEYFLKSYANSFERYYPTSARGVLDTNELMQNYNDKASSLLILMMIAQGAMTIPSIEARWLT

GGFTEACRISLFDLIEKNIIMAGDPIVLRAALLFTVQAAWSGDKWQMDIAMGQRGMYFSMLRHSGILDVRPPATPHINGNISTDALWKDWLQQESRSRLIYSWVMVDQDLSLFH

DTAPLFSVTEFGAPMPDSDRLWQAQTASEWSEMFNQVHEFSNGYSSVGSGARPPSLRDLFRYFLDDEIVVQDVQEIHLTPMHLRLLLHPLQTLVCQYCQLLSCFSDSVASRS

RNRALTAASTRVRLEEVQALLGRWFDLAQRYMKSNPVCPMMHANLVMFHLISLNAVSNFPEIERLARREGVDGSYQQMLWMHKKCLSDVQEAITHAGQVLRLIREMPRGIRP

PWWAGAVYRAGLVFWTDSLAHGESLSPNHQGSYQPSNSAFSVDRLPADHPTILRYMSRREGIPTLTKRDGTHVPLDNGFAILSHCIDVLDEGVATRFSDGIRSKLEKLARG

>*Alternaria viburni* (KAI4665059[a])

MVFCTYCGQSFTRDEHLERHILTHTNVKPFKCFTCHMSFARRDLLQRHYTVHGRNQNNEEGLPPQGIIPKSAGRTPIACSNCAKTKTKCDKKFPCTRCAQRNLKCTLRPTRRA

SKGVQRVGGPPEAGSGDSSESGSNADNHNTSGNVSPNHDSQPKQAMSKSSPAQINQTSHIPDSQPPSQRQSQSPSAPHSRNPSISQPVQPPTHINPEEKSLHLHMPISDTP

PFFDQSPSNGLAQSSSLLSPLPTPVNGMNGFVTSTPMSGYDDFVRTVRDQSDNESPQFMMDPWNGMPMDTTFDPMRIDPALMMSMSMDMSMGPPQDGILGMIPEMSPAQ

TFGQPVQTPRMDESFSDLQIGSSASMFYPSNRHSSIADAGIPDLGAIIAAQDGWTVFRCTPSIASSSCPRTARLNLERLELSLKNHEGWANWSPSWDESDFQQGGQIAVSKL

QEATRDKLLAITQSFLHKALDIHKDGSSSSSSGESPGSTASHSNFVLLPPARVLEYFLKSYANSFERYYPTSARGVLDTNELMQNYNDKASSLLILMMIAQGAMTIPSIEARWLT

GGFTEACRISLFDLIEKNIIMAGDPIVLRAALLFTVQAAWSGDKWQMDIAMGQRGMYFSMLRHSGILDVRPPATPHINGNISTDALWKDWLQQESRSRLIYSWVMVDQDLSLFH

DTAPLFSVTEFGAPMPDSDRLWQAQTASEWSEMFNQVHEFSNGYSSVGSGARPPSLRDLFRYFLDDEIVVQDVQEIHLTPMHLRLLLHPLQTLVCQYCQLLSCFSDSVASRS

RNRALTAASTRVRLEEVQALLGRWFDLAQRYMKSNPVCPMMQANLVMFHLISLNAVSNFPEIERLARREGVDGSYQQMLWMHKKCLSDVQEAITHAGQVLRLIREMPRGIRP

PWWAGAVYRAGLVFWTDSLAHGESLSPNHQGSYQPSNSAFSVDRLPADHPTILRYMSRREGIPTLTKRDGTHVPLDNGFAILSHCVDVLDEGVATRFSDGIRSKLEKLARG

Fig. S2 Protein sequences of 44 Cmr1 homologs in *Alternaria*. The sequences were acquired from the following resources: [a]GenBank, National Center for Biotechnology Information (http://www.ncbi.nih.gov); [b]AGD, *Alternaria* Genome Database (http://alternaria.vbi.vt.edu); [c]JGI, Joint Genome Institute, US Department of Energy (http://www.jgi.doe.gov). NA=not applicable.

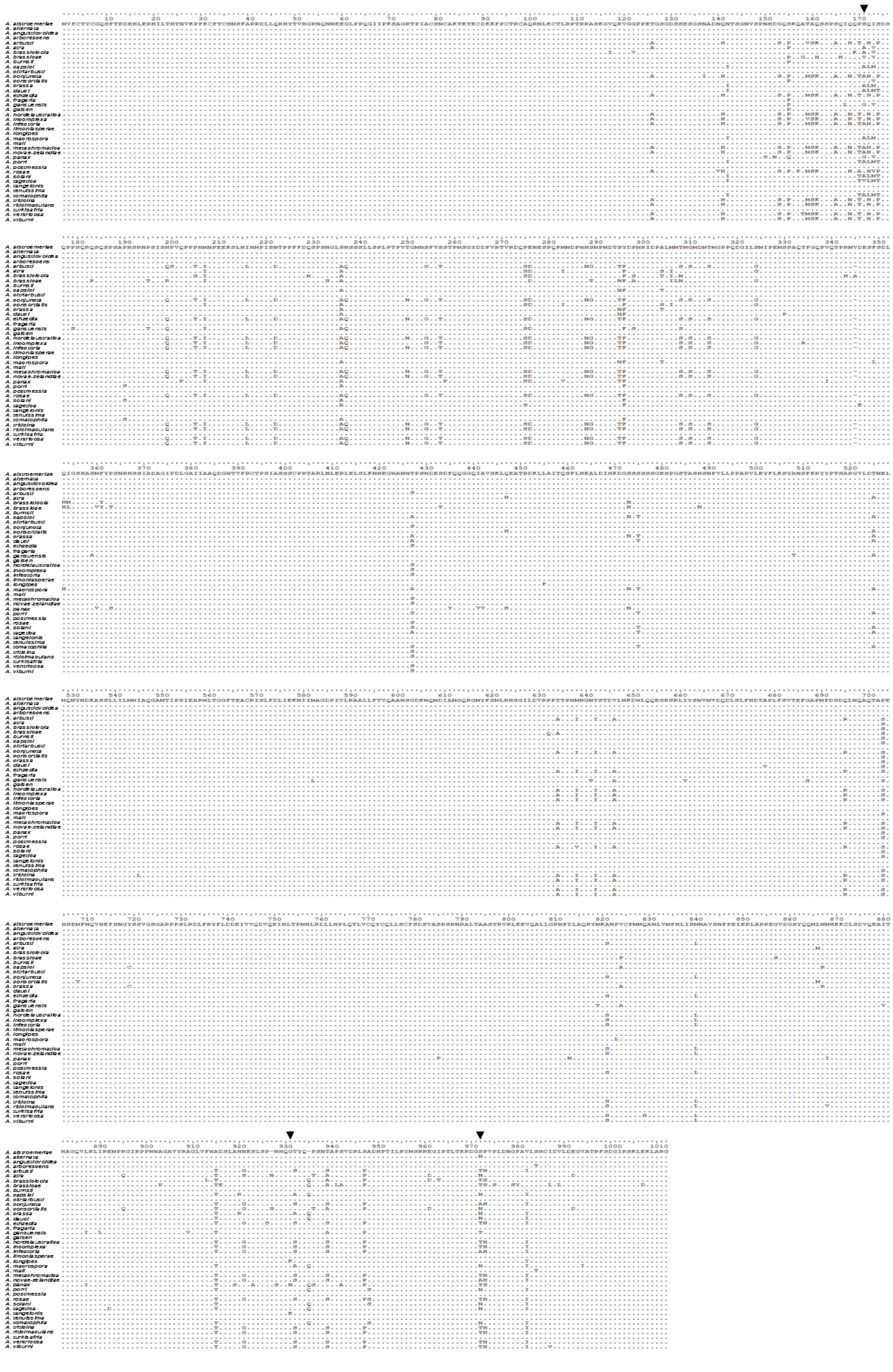

Fig. S3 Multiple sequence alignment of 42 Cmr1 homologs in *Alternaria*. The amino acid sequences were aligned using the ClustalW program implemented in Bioedit version 7.0.9. Identical amino acids are indicated by dots. Alignment gaps are shown by dashes. The amino acid sites under positive selection are indicated by black arrows.

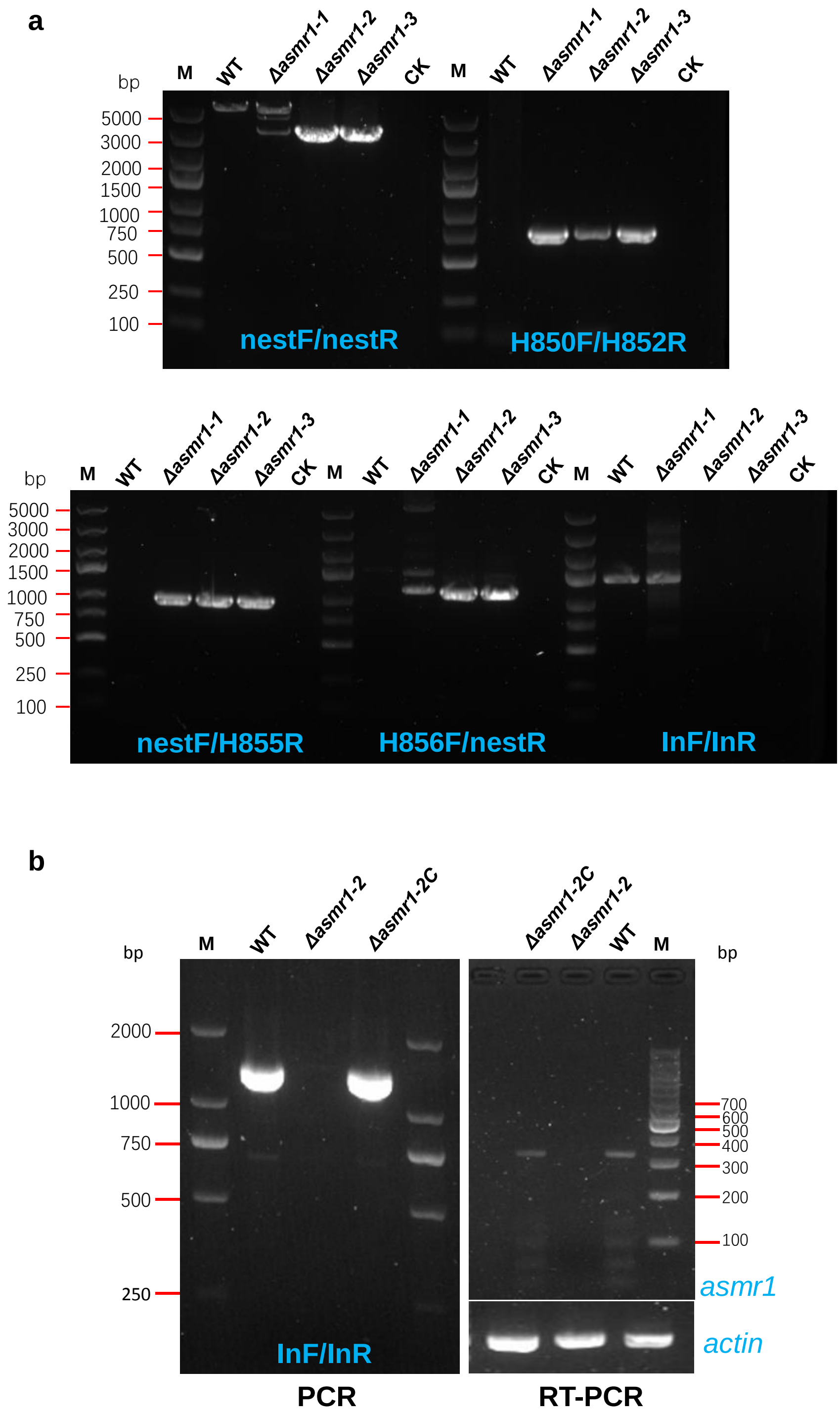

Fig. S4 Verification of *asmr1* deletion and complementation mutants. **a** PCR analysis of *asmr1* deletion mutants with five primer pairs, including Asmr1-nestF/Asmr1-nestR, H850F/H852R, Asmr1-nestF/H855R, H856F/Asmr1-nestR, and Asmr1-InF/Asmr1-InR. **b** PCR analysis of *asmr1* complementation mutant using the primer pair Asmr1-InF/Asmr1-InR. Abbreviations: WT = wild-type *A. solani*, Δ*asmr1-1*, Δ*asmr1-2*, Δ*asmr1-3* = *asmr1* deletion mutants, Δ*asmr1-2C* = complementation mutant Δ*asmr1-2:asmr1*, CK = ddH_2O, M = marker.

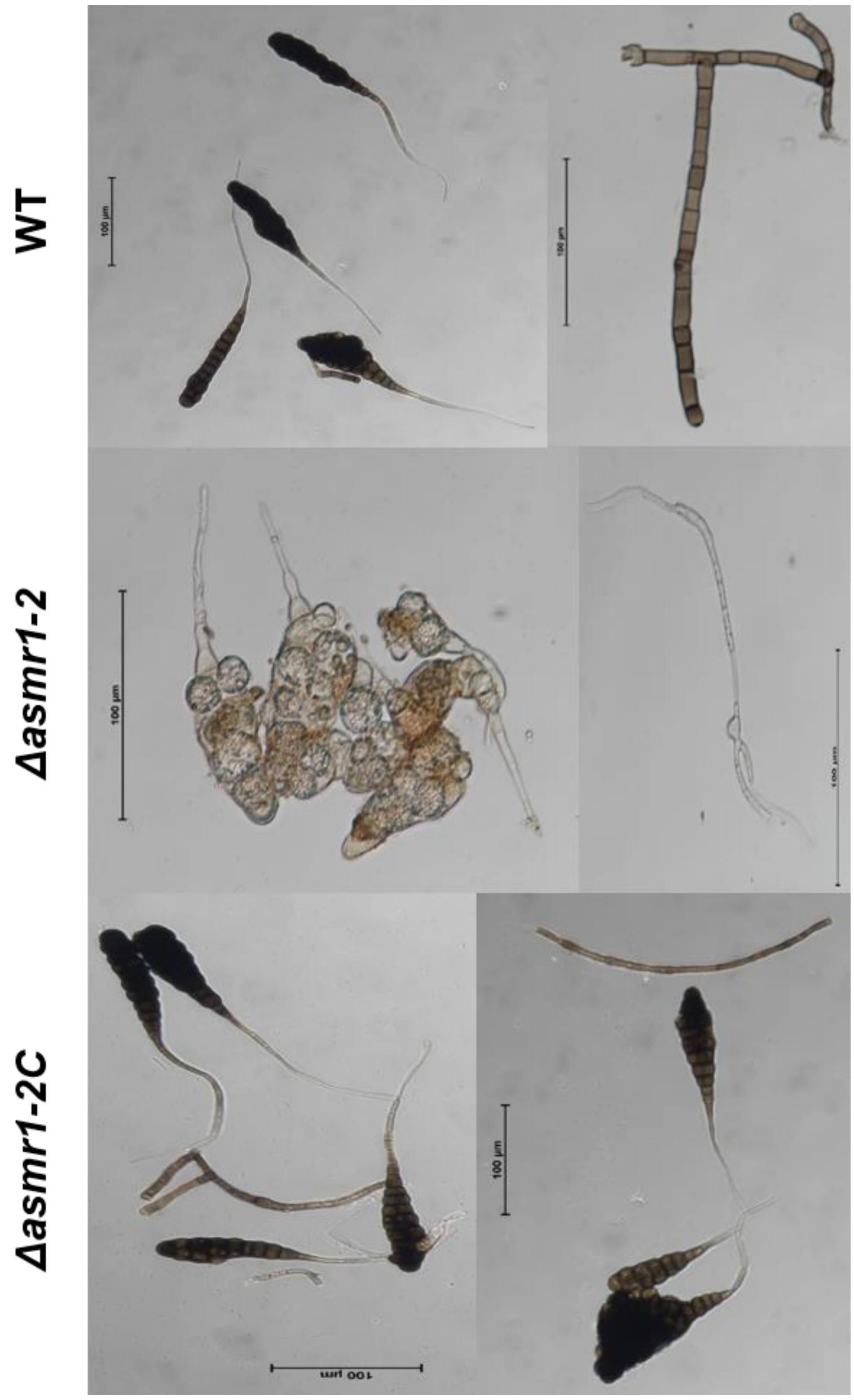

Fig. S5 Effect of *asmr1* deletion on conidial morphology in *A. solani*. Wild-type, *asmr1* deletion and complementation mutants were grown on PCA medium for 5 days at 25°C. Abbreviations: WT = wild-type *A. solani*, Δ*asmr1-2* = *asmr1* deletion mutant, Δ*asmr1-2C* = complementation mutant Δ*asmr1-2:asmr1*.

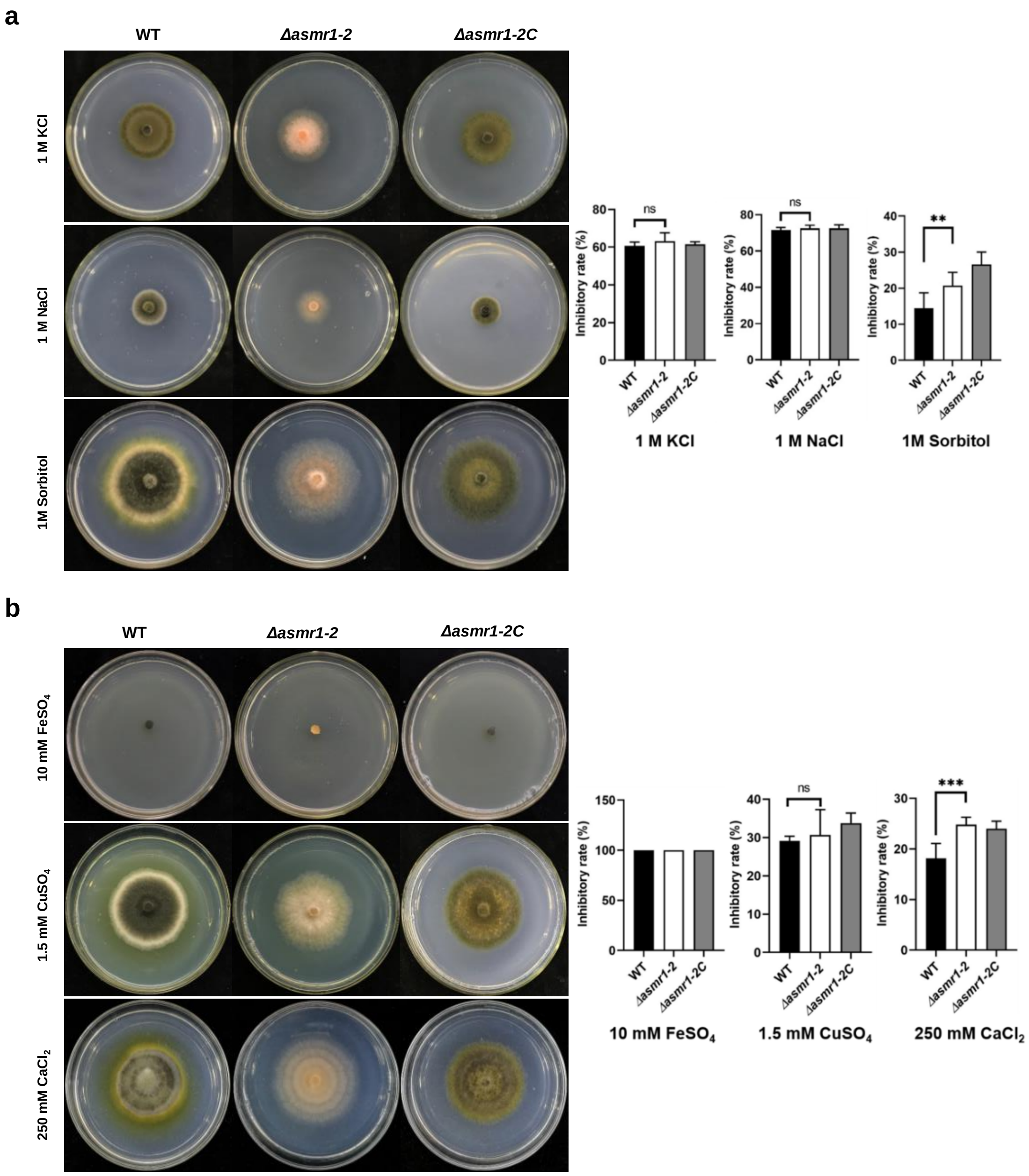

Fig. S6 Effects of *asmr1* deletion on *A. solani* responses to stressors. Comparison of mycelial growth inhibitory rates between mutants and wild-type on PDA medium in the presence of indicated (**a**) osmotic stressors or (**b**) metal ions. Values represent mean from three independent experiments with three replicates each. The significant difference in mycelial growth inhibitory rata was determined by one-way analysis of variance (ANOVA). ****P* < 0.001; ns: not significant (comparison with wild-type). Abbreviations: WT = wild-type *A. solani*, Δ*asmr1-2* = *asmr1* deletion mutant, Δ*asmr1-2C* = complementation mutant Δ*asmr1-2:asmr1*.

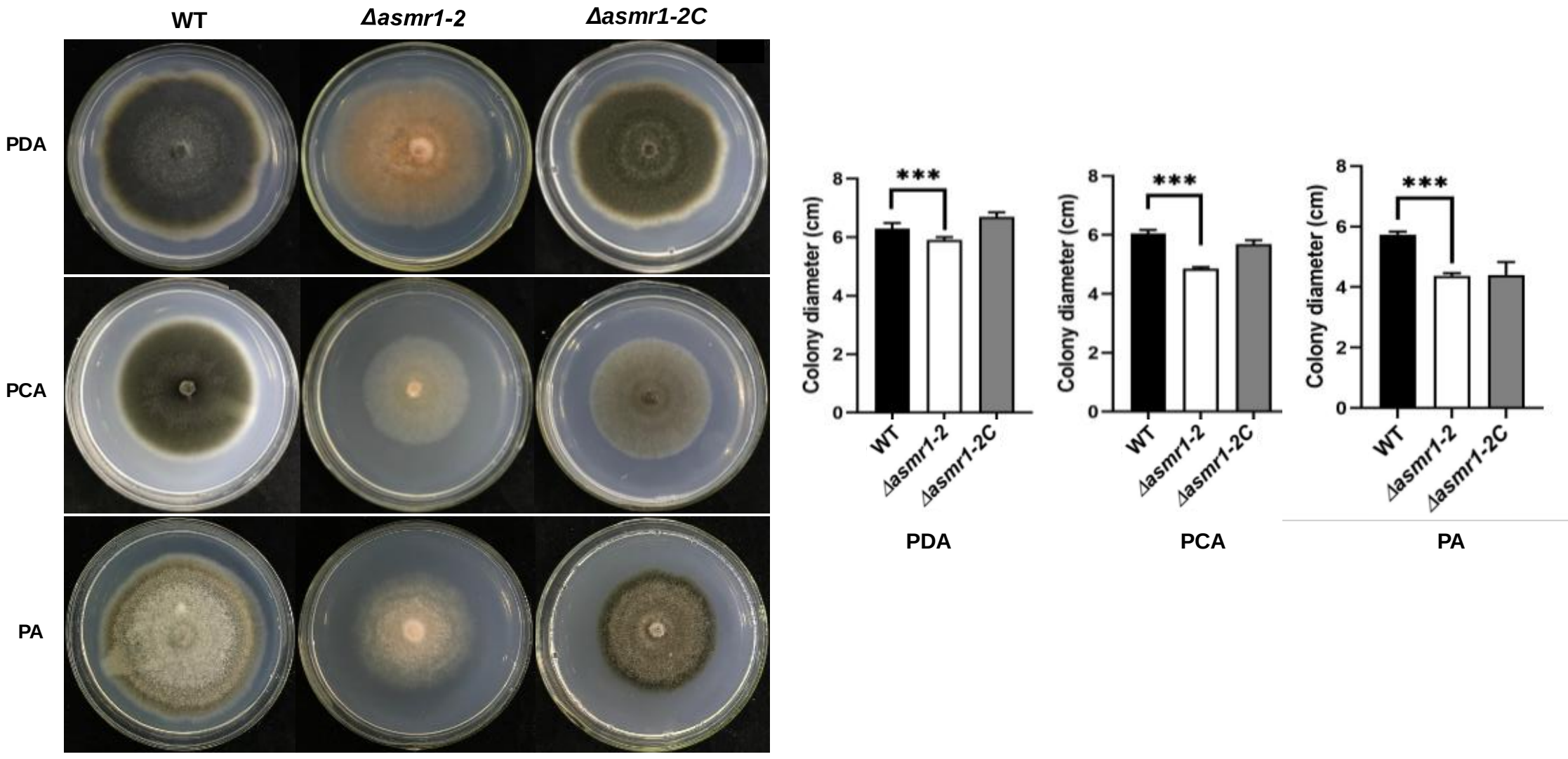

Fig. S7 Effect of *asmr1* deletion on the growth of *A. solani* in different media. Wild-type, *asmr1* deletion and complementation mutant were grown on PDA, PCA, and PA medium for 5 days at 25°C. Values represent mean from three independent experiments with three replicates each. The significant difference in colony diameter was determined by ANOVA. ***P < 0.001 (comparison with wild-type). Abbreviations: WT = wild-type *A. solani*, Δ*asmr1-2* = *asmr1* deletion mutant, Δ*asmr1-2C* = complementation mutant Δ*asmr1-2:asmr1*.

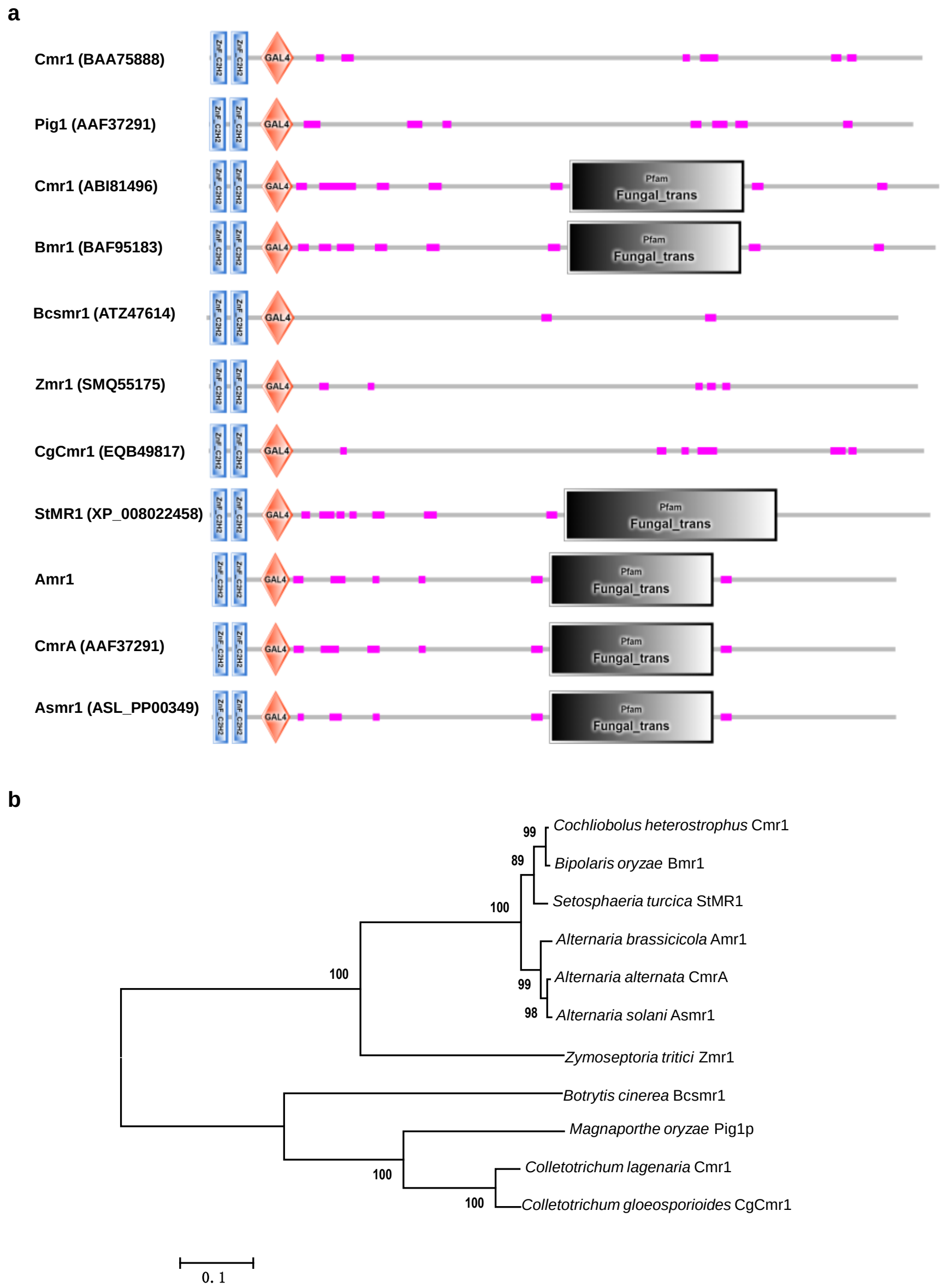

Fig. S8 Structure and phylogeny of 11 fungal Cmr1 homologs. Cmr1 homologs are from *Cochliobolus heterostrophus* (Cmr1), *Magnaporthe grisea* (Pig1), *Colletotrichum lagenarium* (Cmr1), *Bipolaris oryzae* (Bmr1), *Bipolaris cinerea* (Bcsmr1), *Zymoseptoria tritici* (Zmr1), *C. gloeosporioides* (CgCmr1), *Setosphaeria turcica* (StMR1), *Alternaria brassicicola* (Amr1), *A. alternata* (CmrA), and *A. solani* (Asmr1). **a** Graphical representation of motif structure of Cmr1 homologs predicted by SMART server at http://smart.embl-heidelberg.de. ZnF_C2H2 in blue box represents the Cys_2His_2 zinc finger motif (SM000355). GAL4 in an orange box indicates the GAL4-like $Zn(II)_2Cys_6$ binuclear cluster DNA-binding motif (SM00066). Fungal_trans in black box denotes the fungal specific transcription factor motif (PF04082). The pink box represents the low complexity region. **b** The maximum-likelihood (ML) tree generated by MEGA7.0.21 based on the protein sequences of 11 fungal Cmr1 homologs. The scale bar indicates weighted sequence divergence. Boostrap percentages >70% are given at the nodes.

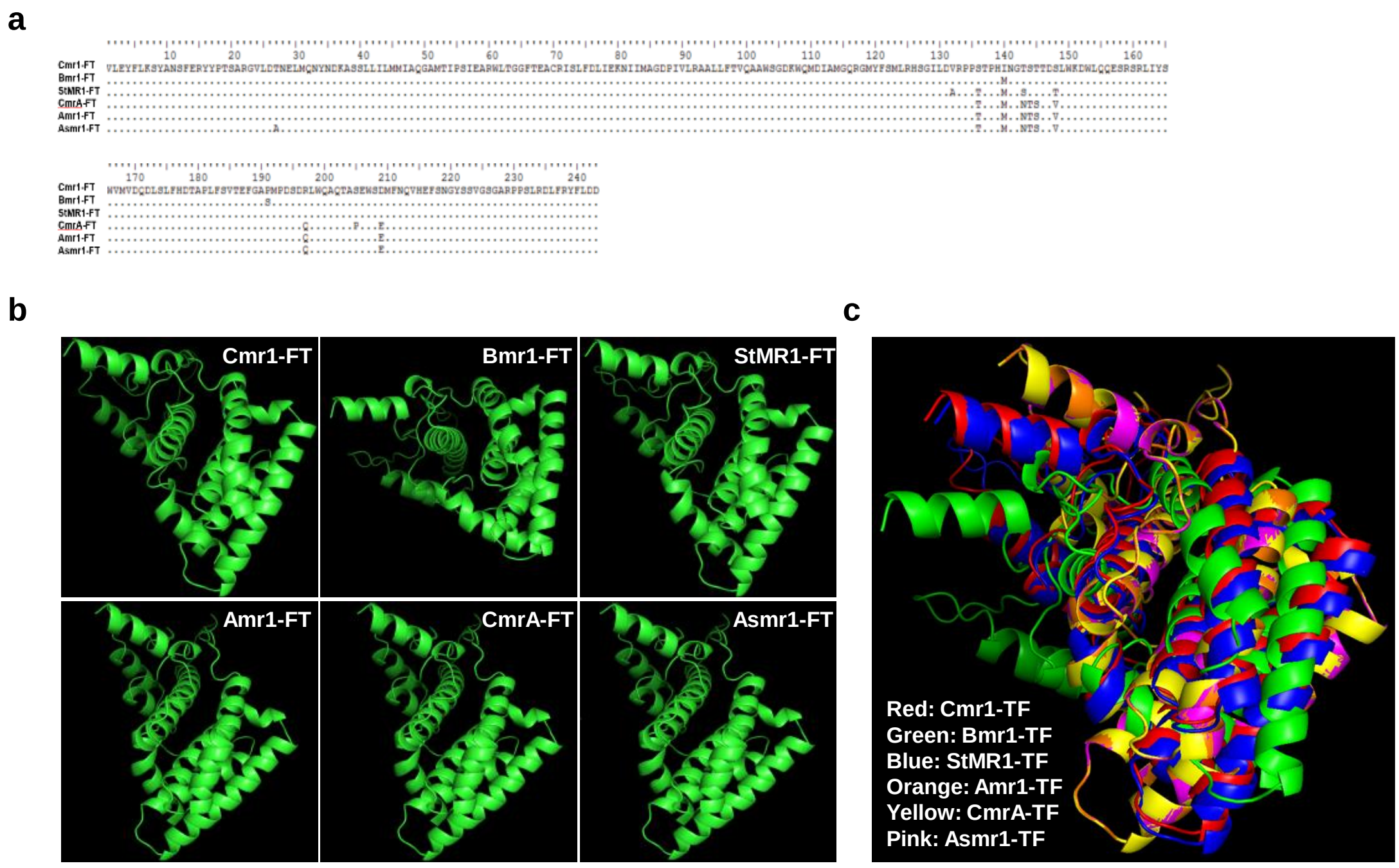

Fig. S9 Alignment and structure of fungal_trans motifs from 6 fungal Cmr1 homologs. Cmr1 homologs are from *Cochliobolus heterostrophus* (Cmr1-FT), *Bipolaris oryzae* (Bmr1-FT), *Setosphaeria turcica* (StMR1-FT), *Alternaria brassicicola* (Amr1-FT), *A. alternata* (CmrA-FT), and *A. solani* (Asmr1-FT). **a** The sequence alignment was generated from the amino acid sequences of 6 fungal_trans (FT) motifs using the ClustalW program implemented in Bioedit version 7.0.9. **b** The structure of FT motifs was illustrated using PyMOL software (http://www.pymol.org/). **c** Merged structures of 6 FT motifs.

Supplemental Table S1 Information about 10 *A. solani* isolates

Isolate	Isolated host	Isolated location	Additional information	Source
DX0917	*Solanum tuberosum*	Dingxi, China	Early blight of potatoes	This study
SH0806	*Solanum tuberosum*	Suihua, China	Early blight of potatoes	Lianmei Tai[a]
BX0917	*Solanum tuberosum*	Benxi, China	Early blight of potatoes	This study
JG0805	*Solanum tuberosum*	Jiagedaqi District, China	Early blight of potatoes	Lianmei Tai[a]
KS0809	*Solanum tuberosum*	Keshan, China	Early blight of potatoes	Lianmei Tai[a]
DQ0803	*Solanum tuberosum*	Daqing, China	Early blight of potatoes	Lianmei Tai[a]
BJPO2207	*Solanum tuberosum*	Beijing, China	Early blight of potatoes	This study
BJTO2207	*Solanum lycopersicum*	Beijing, China	Early blight of tomatoes	This study
JSTO2207	*Solanum lycopersicum*	Jiansu, China	Early blight of tomatoes	This study
SDTO2207	*Solanum lycopersicum*	Shandong, China	Early blight of tomatoes	This study

[a]Heilongjiang Bayi Agricultural University, China

Supplemental Table S2 Primer sequences used in this study

Primer	Sequence (5'-3')
Asmr1F	ATGGTCTTCTGCACATATTG
Asmr1R	TCAACCACGCGCAAGTTTCTC
Asmr1-BD-EcoRIF	TGGCCATGGAGGCCGAATTCATGGTCTTCTGCACATATTG
Asmr1-BD-SalIR	TGCGGCCGCTGCAGGTCGACTCAACCACGCGCAAGTTTCTC
Asmr1-IN1-firstR	AAGCACTTGAAGGGCTTGACATTTGTGTGAGTCAGGATATGTCGTTCGAG
Asmr1-IN1-secondF	ACACAAATGTCAAGCCCTTCAAGTG
Asmr1-IN2-firstR	GACGGTGTAATGTCTTTGCAAAAGATCTCGTCGGGCAAAGCTCATATG
Asmr1-IN2-secondF	AGATCTTTTGCAAAGACATTACAC
Asmr1-IN3-firstR	GTCCACCATGACCCAAGAGTAGATGAGGCGGCTGCGGCTTTCTTGCTG
Asmr1-IN3-secondF	CCTCATCTACTCTTGGGTCATGGTG
Asmr1-722F	ACTTTCGCCTCTTCCCACTC
Asmr1-1790F	GGCATCAAGCTTGCTCATTC
Asmr1-2838F	TGTGGATGCACAAGAAGTGC
Asmr1realF	TGTGGACAGTCGTTCACCAG
Asmr1realR	GGACGGTGTAATGTCTTTGC
Asmr1RTF	TGTGGACAGTCGTTCACCAG
Asmr1RTR	CTTTCGATGCTCGACGTGTA
AsActin1realF	GTATCATGATCGGTATGGGACAG
AsActin1realR	CCAGATCTTCTCCATGTCGTCC
AsActin1RTF	CAATGGTTCGGGTATGTGCAAG
AsActin1RTR	GAAGAGCGAAACCCTCGTGAGT
AsEF1αrealF	TGGGTCCTCGACAAGTTGAA
AsEF1αrealR	GATGAGAATAGCGCAGTCAGC
AspksArealF	ACAACCGTACCGATTTCTCG
AspksArealR	AGCTTGCCTAGAGTGACACC
AsaygArealF	AAGACTCCGGCGAAGCAT
AsaygArealR	TCTTTTACTATCGGCTCGAAATC
AsaygBrealF	GGACAAGTGCCAGGATGAAT
AsaygBrealR	ATCCTCAATCGGCATACACC
Asbrm1realF	GAAGAACGAATTGAAGCCTACG
Asbrm1realR	CTCAGTGTCGGTGCGATG
Asbrm2realF	CGCTGTCTACTCTGGCTCAA
Asbrm2realR	CGTGGTACATGTCGGTCTTG
Asbrm3realF	ACCCTGATGGCAAACCATT
Asbrm3realR	ATCCACCAGAGATGGTCAGG
AslccArealF	CGCTCACCCTATTCATCTCC
AslccArealR	GCTCGAGGAACTGGACACTAA
AslccBrealF	CGACCCACACTACAGGATGT
AslccBrealR	GCAGTGTTGTTGGTGGTGTCT
AslccCrealF	GGAGCGATGGTTACAGCATT
AslccCrealR	GAAGTGCCACTGCCATCTG
AslccDrealF	TCTTTGGAGGCATCATCATC
AslccDrealR	AGAGGTCTCGGCGGAGTG
AslccErealF	CGCCTAATGGCGGATACTAC
AslccErealR	CTCTTCGTGCTTTGGTCCAT
AslccFrealF	ACACGATAGAGCCTGGGAGA
AslccFrealR	GAAGTCGGCGTTGGTATTG
AslccGrealF	GGACACGGCGAGTGTAGAAG
AslccGrealR	CGCAAACATCATCATCAAGC
Asmr1-5F	CTGATCCGACGTGTGTGC
Asmr1-5R	CAAAATAGGCATTGATGTGTTGACCTCCACAACAGTGGTTGAGCGAGA
Asmr1-3F	CTCGTCCGAGGGCAAAGGAATAGAGTAGGTTTCTCCATCCGCATTGTC
Asmr1-3R	GACGTGCCTCGATACTGCTT
Asmr1-nestF	TCACTCTCAGCACGAACCTC
Asmr1-nestR	GCCCTCGTGTCCTCTCATAT
Asmr1-CF	ACTCACTATAGGGCGAATTGGGTACTCAAATTGGTTGGTACTCGGTTGGCGTAGAG
Asmr1-CR	CACCACCCCGGTGAACAGCTCCTCGCCTTGCTCACACCACGCGCAAGTTTCTC
ID-R	GTCAGCTTGCCGTAGGTGGCA
HphCR	GCACCAAGCAGCAGATGATA
HphCF	ACTGTCGGGCGTACACAAAT
H850F	TTCCTCCCTTTATTTCAGATTCAA
H852R	ATGTTGGCGACCTCGTATTGG
H855R	GCTGATCTGACCAGTTGC
H856F	GTCGATGCGACGCAATCGT
hph1349F	GGAGGTCAACACATCAATGCCTATT
hph1349R	CTACTCTATTCCTTTGCCCT
Asmr1-InF	GCAAATAGCCGTGTCGAAAT
Asmr1-InR	CTTGTGCATCCACAGCATCT

Supplemental Table S3 List of information about 44 *Alternaria* spp.

Species name	*Alternaria* section	Strain/Isolate	Isolated host	Isolated location	Additional information	Source[a]
Alternaria alstroemeriae	NA	CBS 118809	SpX12/RR9 Inside cage Flight	USA	NA	GenBank
Alternaria alternata	*Alternata*	ATCC 66891; EGS 34-016; CBS 916.96; BMP 0269	*Arachis hypogaea*	India	Leaf spot, rots of plants	JGI
Alternaria angustiovoidea	*Alternata*	FKII-L2-CM-DRAB1	Cleanroom at space-craft assembly facility	Pasadena, CA, USA	NA	GenBank
Alternaria arborescens	*Alternata*	FERA675	*Malus domestica*	NA	NA	GenBank
Alternaria arbusti	*Infectoriae*	BMP 1465	*Pyrus pyrifolia*	CA, USA	NA	GenBank
Alternaria atra	*Ulocladioides*	CS162	*Solanum chilense*	Chile	Leaf	GenBank
Alternaria brassicicola	*Brassicicola*	ATCC 96836; EGS 42-002; CBS 118699; BMP1950	*Brassica oleracea*	USA	Blackspot of brassica	GenBank
Alternaria brassicae	NA	J3	*Brassica juncea*	India	NA	GenBank
Alternaria burnsii	*Alternata*	CBS 107.38	*Cuminum cyminum*	Maharashtra, India	NA	GenBank
Alternaria capsici	*Porri*	ATCC MYA-998; EGS 45–075; BMP 0180	*Capsicum annuum*	Australia	Leaf spot of solanaceae (pepper)	JGI
Alternaria carthami	*Porri*	BMP 1963; CBS 635.80	*Carthamus tinctorius*	Perugia, Italy	Leaf spot and blight of safflower	JGI
Alternaria citriarbusti	*Alternata*	EGS 46-140; BMP 2343; SH-MIL-8 s	Citrus	NA	Brown/black spot of citrus	JGI
Alternaria conjuncta	*Infectoriae*	BMP 0040	*Pastinaca sativa*	Switzerland	NA	GenBank
Alternaria consortialis	*Ulocladioides*	JCM1940	Goat dung	Hisper Valley, Karakorum	NA	GenBank
Alternaria crassa	*Porri*	BM P0172; ACR1	Solanaceae	NA	Leaf spot of solanaceae	JGI
Alternaria dauci	*Porri*	ATCC 36613; BMP 0167	Carrot	USA	Leaf blight of carrots	JGI
Alternaria destruens	*Alternata*	ATCC 204363; EGS 46-069; BMP 0317	*Cuscuta gronovii*	Rochester, MA, USA	Infecting and suppressing dodder (weed)	AGD
Alternaria ethzedia	*Infectoriae*	BMP 0044	*Brassica napus*	Switzerland	NA	GenBank
Alternaria fragariae	NA	BMP 3062; NAF-8	*Fragaria* x *ananassa*	NA	Black spot disease of strawberry	JGI
Alternaria gansuensis	*Undifilum*	LYZ1412	*Astragalus adsurgens* Pall.	Loess Plateau, China	Swainsonine-producing fungi	GenBank
Alternaira gaisen	*Alternata*	CBS 632.93; EGS 90-0512; BMP 2338	*Pyrus pyrifolia* cv. Nijiseiki	Tottori, Japan	Black spot, ring spot disease of pear	JGI
Alternaria hordeiaustralica	*Infectoriae*	BMP 2776	*Hordeum vulgare*	Australia	NA	GenBank
Alternaria incomplexa	*Infectoriae*	BMP 0042	NA	Idaho, USA	NA	GenBank
Alternaria infectoria	*Infectoriae*	BMP 0036	*Triticum* sp.	United Kingdom	NA	GenBank
Alternaria limoniasperae	*Alternata*	EGS 44–159; BMP 2335	Citrus	NA	Leaf spot of citrus	JGI
Alternaria longipes	*Alternata*	CBS 540.94; EGS 30-033; BMP 0313	*Nicotiana tabacum*	North Carolina, USA	Tobacco Brown spot/Black/browen leaf spot of tobacco	JGI
Alternaria macrospora	*Porri*	BMP 1949; CH3	*Gossypium hirsutum*	NA	Leaf spot of cotton	JGI
Alternaria mali	*Alternaria*	BMP 3063; M-71	*Malus domestica*	NA	Leaf ring spot of apple	JGI
Alternaria metachromatica	*Infectoriae*	BMP 0045	*Triticum aestivum*	Australia	NA	GenBank
Alternaria novae-zelandiae	*Infectoriae*	BMP 2774	*Daucus carota*	New Zealand	NA	GenBank
Alternaria panax	*Panax*	BNCC115425	*Panax quinquefolium*	Beijing, China	American ginseng black spot	GenBank
Alternaria porri	*Porri*	BMP 0178; Z6B	*Allium cepa*	NA	Purple blotch, leaf blight and bulb rot of Allium (onion)	JGI
Alternaria postmessia	*Alternata*	BMP 2775	*Citrus* x *tangelo*	Florida, USA	NA	GenBank
Alternaria rosae	*Pseudoalternaria*	MPI-PUGE-AT-0040	*Arabidopsis thaliana*	Pulheim, Germany	*Arabidopsis thaliana* root mycobiota	GenBank
Alternaria solani	*Porri*	BMP 0185	*Solanum tuberosum/Solanum lycopersicum*	NA	Early blight of potatoes and tomatoes	AGD
Alternaria tagetica	*Porri*	EGS 44–044; BMP 0179	*Tagetes lemmonii*	NA	Leaf spot of marigold	AGD
Alternaria tangelonis	*Alternata*	EGS 45–080; BMP 2327; BC2-RLR-1 s	Citrus	NA	Leaf spot of citrus	JGI
Alternaria tenuissima	*Alternata*	ATCC 96828; EGS 34-015; CBS 918.96; BMP 0304	*Dianthus* sp.	UK	Leaf spot of plants	JGI
Alternaria tomatophila	*Porri*	BMP 2032; CBS 109156	*Lycopersicon esculentum*	Indiana, USA	Leaf spot of tomato	JGI
Alternaria triticina	*Infectoriae*	CBS 763.84	*Triticum aestivum*	New Delhi, India	NA	GenBank
Alternaria triticimaculans	*Infectoriae*	BMP 0046	*Triticum aestivum*	Argentina	NA	GenBank
Alternaria turkisafria	*Alternata*	BMP 3436; SH-MIL-20s	Citrus	USA	Leaf spot of citrus	AGD
Alternaria ventricosa	*Infectoriae*	BMP 2768	*Pyrus* x *bretschneideri*	China	NA	GenBank
Alternaria viburni	*Infectoriae*	BMP 2772	*Viburnum* sp.	Netherlands	NA	GenBank

[a]GenBank, National Center for Biotechnology Information (http://www.ncbi.nih.gov); JGI, Joint Genome Institute, US Department of Energy (http://www.jgi.doe.gov); AGD, *Alternaria* Genome Database (http://alternaria.vbi.vt.edu). NA=not applicable

Supplemental Table S4 Information about 44 Cmr1 homologs in the genus *Alternaria*

Species name	Gene accession	Gene sequence length	Intron location	Protein accession	Protein sequence length
Alternaria alstroemeriae	NA	3273	71-170; 226-309; 2161-2216	NA	1010
Alternaria alternata	KJ939356[a]	3273	71-170; 226-309; 2161-2216	AIG15468[a]	1010
Alternaria angustiovoidea	NA	3273	71-170; 226-309; 2161-2216	NA	1010
Alternaria arborescens	XM_028649146[a]	3273	71-170; 226-309; 2161-2216	XP_028511872[a]	1010
Alternaria arbusti	NA	3263	71-169; 225-301; 2150-2206	NA	1009
Alternaria atra	NA	3267	71-169; 225-300; 2152-2207	NA	1011
Alternaria brassicicola	NA	3282	71-170; 226-311; 2163-2222	NA	1011
Alternaria brassicae	NA	3254	71-146; 202-287; 2139-2194	NA	1011
Alternaria burnsii	XM_038925615[a]	3273	71-170; 226-309; 2161-2216	XP_038791471[a]	1010
Alternaria capsici	NA	3277	71-170; 226-310; 2162-2217	NA	1011
Alternaria carthami	ACM_PT10841[b]	3283	71-170; 226-310; 2162-2217	ACM_PP10841[b]	992
Alternaria citriarbusti	NA	3273	71-170; 226-309; 2161-2216	NA	1010
Alternaria conjuncta	NA	3263	71-169; 225-301; 2150-2206	NA	1009
Alternaria consortialis	NA	3267	71-169; 225-300; 2152-2207	NA	1011
Alternaria crassa	ACM_PT08031[b]	3277	71-170; 226-310; 2162-2217	ACM_PP08031[b]	1011
Alternaria dauci	ADC_PT01443[c]	3277	71-170; 226-310; 2162-2217	ADC_PP01443[c]	1011
Alternaria destruens	ADT_PT05662[b]	3344	71-170; 226-309; 2204-2259	ADT_PP05662[b]	790
Alternaria ethzedia	NA	3263	71-169; 225-301; 2150-2206	NA	1009
Alternaria fragariae	AFG_PT01606[c]	3273	71-170; 226-309; 2161-2216	AFG_PP01606[c]	1010
Alternaria gansuensis	NA	3277	71-170; 226-310; 2162-2217	NA	1009
Alternaira gaisen	AGS_PT02526[c]	3273	71-170; 226-309; 2161-2216	AGS_PP02526[c]	1010
Alternaria hordeiaustralica	NA	3263	71-169; 225-301; 2150-2206	NA	1009
Alternaria incomplexa	NA	3263	71-169; 225-301; 2150-2206	NA	1009
Alternaria infectoria	J4E92_008460[a]	3263	71-169; 225-301; 2150-2206	KAI4920335[a]	1009
Alternaria limoniasperae	NA	3273	71-170; 226-309; 2161-2216	NA	1010
Alternaria longipes	NA	3273	71-170; 226-309; 2161-2216	NA	1010
Alternaria macrospora	NA	3277	71-170; 226-310; 2162-2217	NA	1011
Alternaria mali	AML_PT04777[c]	3273	71-170; 226-309; 2161-2216	AML_PP04777[c]	1010
Alternaria metachromatica	J4E83_001950[a]	3263	71-169; 225-301; 2150-2206	KAI4634631[a]	1009
Alternaria novae-zelandiae	J4E88_001357[a]	3263	71-169; 225-301; 2150-2206	KAI4692986[a]	1009
Alternaria panax	G6011_11168[a]	3282	71-170; 226-311; 2163-2222	KAG9192434[a]	1011
Alternaria porri	APR_PT04911[c]	3277	71-170; 226-310; 2162-2217	APR_PP04911[c]	1011
Alternaria postmessia	NA	3273	71-170; 226-309; 2161-2216	NA	1010
Alternaria rosae	XM_046164914[a]	3271	71-168; 224-309; 2158-2214	XP_046026581[a]	1009
Alternaria solani	ASL_PT00349[b]	3277	71-170; 226-310; 2162-2217	ASL_PP00349[b]	1011
Alternaria tagetica	ATT_PT04326[b]	3277	71-170; 226-310; 2162-2217	ATT_PP04326[b]	1011
Alternaria tangelonis	NA	3273	71-170; 226-309; 2161-2216	NA	1010
Alternaria tenuissima	NA	3273	71-170; 226-309; 2161-2216	NA	1010
Alternaria tomatophila	ATM_PT05196[b]	3277	71-170; 226-310; 2162-2217	ATM_PP05196[b]	1011
Alternaria triticina	NA	3263	71-169; 225-301; 2150-2206	NA	1009
Alternaria triticimaculans	NA	3263	71-169; 225-301; 2150-2206	NA	1009
Alternaria turkisafria	NA	3273	71-170; 226-309; 2161-2216	NA	1010
Alternaria ventricosa	NA	3263	71-169; 225-301; 2150-2206	NA	1009
Alternaria viburni	J4E79_003358[a]	3263	71-169; 225-301; 2150-2206	KAI4665059[a]	1009

[a]*GenBank*, National Center for Biotechnology Information (http://www.ncbi.nih.gov)

[b]*AGD, Alternaria* Genome Database (http://alternaria.vbi.vt.edu)

[c]*JGI*, Joint Genome Institute, US Department of Energy (http://www.jgi.doe.gov)

NA=not applicable
